# The effect of paclitaxel on apoptosis, autophagy and mitotic catastrophe in AGS cells

**DOI:** 10.1038/s41598-021-02503-9

**Published:** 2021-12-06

**Authors:** Tin Myo Khing, Won Seok Choi, Dong Min Kim, Wah Wah Po, Wynn Thein, Chang Yell Shin, Uy Dong Sohn

**Affiliations:** Laboratory of Signaling and Pharmacological Activity, Department of Pharmacology, College of Pharmacy, Chung-Ang University, Seoul, 06974 Republic of Korea

**Keywords:** Cancer, Cell biology, Molecular biology

## Abstract

Paclitaxel is an anti-microtubule agent that has been shown to induce cell death in gastric cancer. However, the detailed mechanism of action is unclear. In this study, we reveal that the paclitaxel-induced cell death mechanism involves mitotic catastrophe, autophagy and apoptosis in AGS cells. Paclitaxel induced intrinsic apoptosis by activating caspase-3, caspase-9 and PARP. In addition, the significant increase in autophagy marker LC3B-II, together with Atg5, class III PI3K and Beclin-1, and the down-regulation of p62 following paclitaxel treatment verified that paclitaxel induced autophagy. Further experiments showed that paclitaxel caused mitotic catastrophe, cell cycle arrest of the accumulated multinucleated giant cells at the G2/M phase and induction of cell death in 24 h. Within 48 h, the arrested multinucleated cells escaped mitosis by decreasing cell division regulatory proteins and triggered cell death. Cells treated with paclitaxel for 48 h were grown in fresh medium for 24 h and checked for CDC2, CDC25C and lamin B1 protein expressions. These proteins had decreased significantly, indicating that the remaining cells became senescent. In conclusion, it is suggested that paclitaxel-induced mitotic catastrophe is an integral part of the cell death mechanism, in addition to apoptosis and autophagy, in AGS cells.

## Introduction

Cancer is the leading cause of death in Korea^1^ and the second leading cause of death globally^2^. Gastric cancer is one of the most common types of cancer in men and is responsible for a third of cancer deaths^2^. Unlike in normal tissue, cancerous tumours are produced by uncontrollable cell division. Cell division is part of the cell cycle, whether the cells are normal or cancerous. Chemotherapy is most effective at killing cells that are rapidly dividing^3^. Paclitaxel is a microtubule-stabilising drug that is approved as an anti-cancer agent in monotherapy or combination therapy for the treatment of several types of cancer, including AIDS-related Kaposi sarcoma, breast cancer, non-small cell lung cancer and advanced ovarian cancer^4^. Furthermore, several phase-I/II clinical trials have highlighted paclitaxel for its effectiveness in treating advanced gastric cancers^5,6^. However, resistance to paclitaxel chemotherapy limits its success. Therefore, elucidating the details underlying the paclitaxel-induced cell death mechanism on gastric cancer cells is required.

Among the various mechanisms of anti-tumour drugs, programmed cell death (apoptosis) is the most common. There are two common types of apoptotic pathways; intrinsic and extrinsic. In the early phase of apoptosis, DNA damage causes permeabilisation of the mitochondrial outer membrane due to insertion of the pro-apoptotic protein, B-cell CLL/lymphoma 2 (Bcl-2)-associated X protein (Bax) and leads to inhibition of Bcl-2 in the intrinsic apoptotic pathway. Activation of the extrinsic pathway induces the formation of the death-inducing signal complex (DISC), which leads to caspase-8 activation. In the late phase, activation of caspase-3/7, cleavage of poly (ADP-ribose) polymerase (PARP), DNA fragmentation and cell membrane disruption/blebbing are enhanced^7^. Despite many studies on the apoptotic effect of paclitaxel in different cell lines^8,9^, those on gastric cancer cell lines are limited.

Another cell death mechanism is autophagy. Autophagy, also called macro-autophagy, is a cellular catabolic degradation process induced by nutrient starvation or metabolic stress^10,11^. In this process, the cytosolic form of microtubule-associated protein 1 light chain 3B (LC3B-I) is transformed into the lapidated LC3B-II and forms an integral part of the autophagosome membrane. Class III phosphoinositide-3 kinase (class III PI3K), Beclin-1 (also known as autophagy-related gene 6 [Atg 6]), Atg 5 and Atg 16L, together with lapidated LC3B-II, stimulate the elongation of the phagophore for autophagosome formation. Once p62/sequestosome 1 (SQSTM1) interacts with LC3B, it becomes a substrate for autophagosomes^12,13^. Cellular components or whole organelles are sequestered and enclosed in double-membrane vesicles, autophagosomes. By fusing these autophagosomes with lysosomes, auto-phagolysosomes are formed and degraded by proteases^13^. Data suggests that autophagy induction, specifically in the senescence transition phase, correlates with inhibition of the mechanistic (formerly mammalian) target of rapamycin (mTOR) activity^14^. Conversely, some studies reported that cell cycle inhibitors caused chemo-resistance and delayed cell death by activating autophagy^15–17^. Therefore, if autophagy is involved in the paclitaxel-induced cell death mechanism, it is required to define whether the involvement of autophagy triggers cell survival or cell death.

Mitotic catastrophe is a cell death mechanism triggered by aberrant or dysregulated mitosis. The morphological markers of mitotic catastrophe are multinucleation or micronucleation. The giant multinucleated cells are formed from the clusters of mis-segregated uncondensed chromosomes^18^. During mitotic arrest, cell fate is controlled by two competing pathways that mediate mitotic cell death (activation of the pro-death signals) and mitotic slippage (protection against cyclin B degradation). The activity thresholds and stochastic rivalry between these two pathways determine the cell fate during a prolonged mitotic arrest^19,20^. Cell cycle progression is governed by cyclin-dependent kinases (CDKs). CDKs bind to their essential regulatory subunits called cyclins and drive cell cycle progression^21–23^. Cell division cycle 25C (CDC25C) enhances mitotic cell G2/M transition by dephosphorylation of CDK1 to activate the cyclin B1/CDK1 complex^24^. To our best knowledge, the detailed mechanism of paclitaxel-induced mitotic catastrophe remains elusive.

Cellular senescence is a potent tumour-suppressive mechanism that alters nuclear morphology, causing an enlarged and often irregular nuclear envelope^25,26^. The nuclear lamina is a protein network lining the inner surface of the nuclear envelope, and it controls the size, shape and stability of the nucleus. The nuclear lamins are the major structural proteins of the lamina. Lamin B1 degradation is an indicator of senescence in culture and in vivo^27^. Autophagy is thought to mediate the degradation of the nuclear lamina components in mammals^28^.

In this study, we used the AGS cell line to investigate the detailed mechanism of paclitaxel-induced cell death in gastric cancer cells. The involvement of apoptosis, autophagy and mitotic catastrophe were evaluated. Autophagy is a potential therapeutic target of anti-cancer drugs because it confers drug resistance. This study aims to support the current clinical trials of paclitaxel treatment in advanced gastric cancers by revealing the molecular mechanism of paclitaxel-induced cell death.

## Results

### Determination of cytotoxic effect of paclitaxel in AGS cells

**Figure 1:**
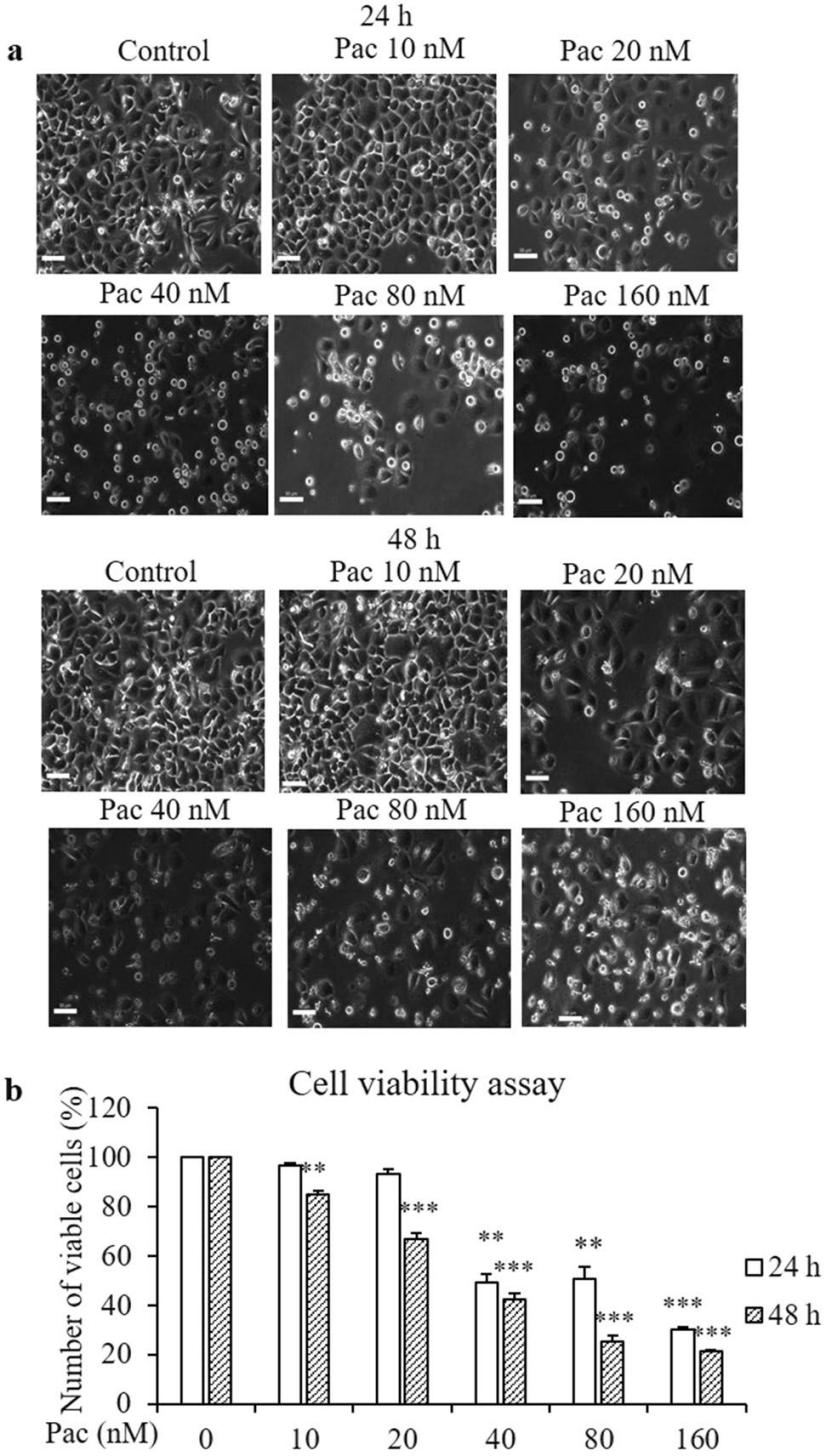
Paclitaxel suppresses the viability of AGS cells. The cells were treated with the indicated concentrations of paclitaxel for 24 and 48 h, and the MTT assay was conducted to determine cell viability. (**a**) Cell morphology was observed by optical microscopy (× 20 magnification). (**b**) The graph shows the percentage of viable cells compared with the non-treated group. All data are presented as mean ± SEM, *n* = 5. (*P < 0.05, **P < 0.01 and ***P < 0.001 compared to the non-treated group).

To determine the cytotoxicity of paclitaxel, AGS cells were treated with 10–160 nM paclitaxel for 24 and 48 h. The non-treated AGS cells were polygonal in shape, with a homogeneous size distribution. In comparison, the paclitaxel-treated cells were larger, and there were more detached, round-shaped cells that showed a concentration-dependent increase in abundance (Fig. 1a). To identify the half inhibitory concentration (IC_50_) of paclitaxel in AGS cells, cell viability was evaluated by the 3-[4,5-dimethylthiazol-2-yl]-2,5-diphenyl-tetrazolium bromide (MTT) assay. The growth of AGS cells was suppressed by paclitaxel in a concentration- and time-dependent manner. Paclitaxel was cytotoxic at 20 nM, and the cell viability decreased to 50% following treatment with 40 nM paclitaxel for 24 h compared to the non-treated group (Fig. 1b). Therefore, paclitaxel concentrations of 20, 40 and 80 nM were targeted for further study.

### Paclitaxel induced apoptosis through cleavage of caspase-3 and PARP and led to the formation of multinucleated cells after 24- and 48-h treatments

**Figure 2:**
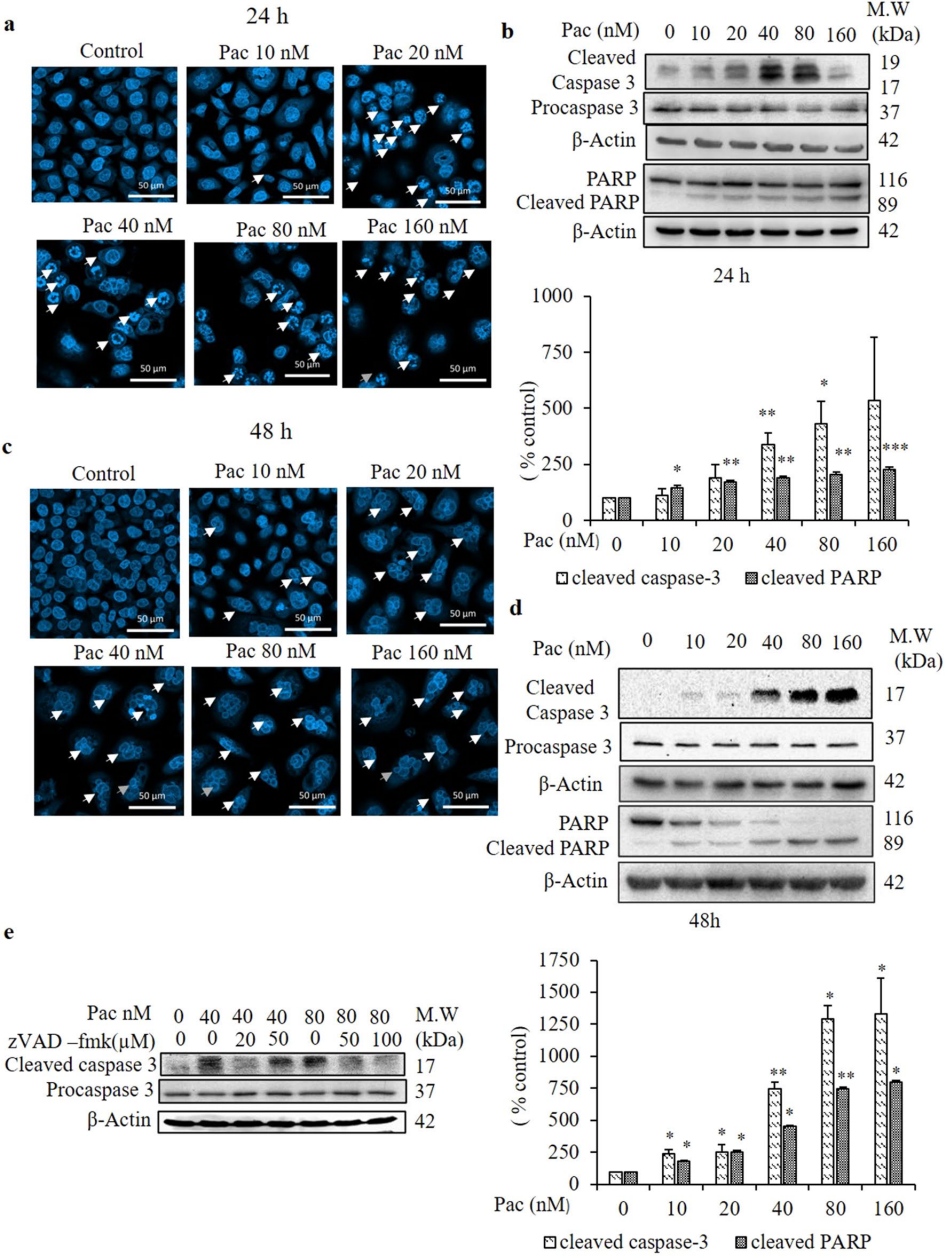
Evaluation of nuclear morphology by DAPI staining and cleaved caspase-3 and PARP protein expressions by western blot analysis after 24- and 48-h treatments. (**a**,**c**) The nuclear morphologies of AGS cells were assessed by DAPI staining combined with confocal microscopy (× 40) after treatment with the indicated concentrations of paclitaxel for 24 and 48 h and compared with the non-treated cells. White arrow indicates the fragmented nuclei (**a**) and multinucleated cells (**c**). (**b**,**d**) Protein expressions of procaspase-3 and cleaved caspase-3 and PARP of AGS cells were determined by western blot analysis after 24- and 48-h treatments. The full-length gel blots were described in Supplementary Fig. A. (**e**) zVAD-fmk was used as a caspase inhibitor in the protein expression of cleaved caspase-3. β-actin was used as the loading control. The blots were quantified by ImageJ software. All data are presented as the mean ± SEM, *n* = 3. (*P < 0.05, **P < 0.01 and ***P < 0.01 compared to the non-treated group).

**Figure 3:**
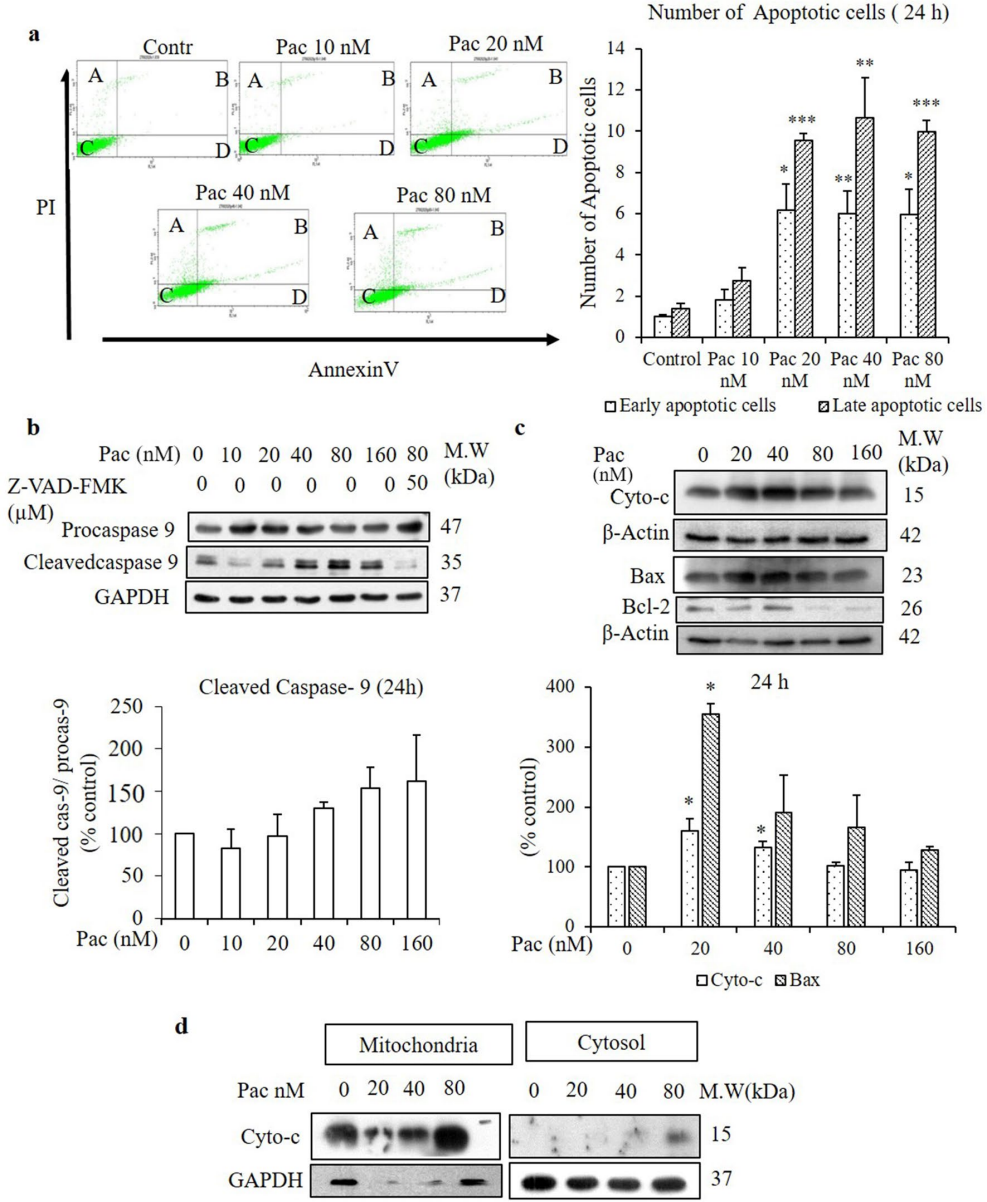

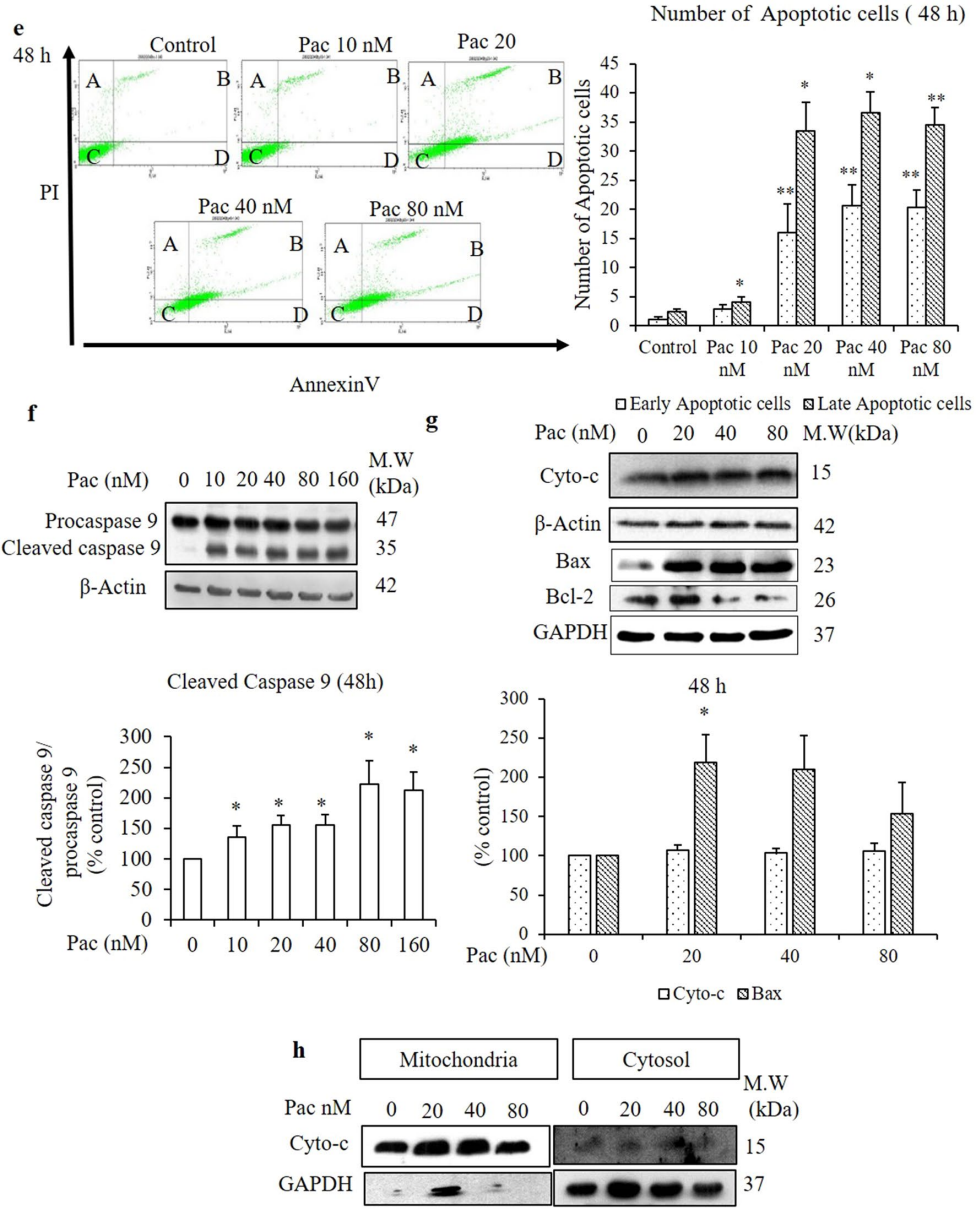
Paclitaxel induced apoptosis without release of cytochrome c from mitochondria. Apoptotic cells were determined by flow cytometry following Annexin V/PI staining and western blot analysis after 24- and 48-h treatments with paclitaxel at the indicated concentrations in which A = necrotic cells, B = late apoptotic cells, C = live cells and D = early apoptotic cells. (**a**,**e**) Cytograph and quantitative analysis of early and late apoptotic cells for the 24- and 48-h treatments. After 24- and 48-h treatments with the indicated paclitaxel concentrations, the protein expressions of (**b,f**) cleaved caspase-9 and (**c**,**g**) Bax, Bcl2 and cytochrome *c* of whole-cell lysates were determined by western blot analysis. β-Actin and GAPDH were used as the loading controls. (**d**,**h**) After mitochondrial fractionation, the release of cytochrome *c* from mitochondria to cytosol was determined by analysing the protein expression of cytochrome *c* in mitochondria and cytosol, separately, by western blot analysis. The blots were quantified by ImageJ software. The full-length gel blots were described in Supplementary Fig. B. All data are presented as the mean ± SEM, *n* = 3. (*P < 0.05, ** *P* < 0.01 and ***P < 0.001 compared to the non-treated group).

To identify whether the paclitaxel-induced cell death mechanism was related to apoptosis in AGS cells, both the non-treated and paclitaxel-treated groups, respectively, were stained with DAPI, a fluorescent DNA-binding dye, and their nuclear morphologies were observed under a confocal microscope. In the cells treated for 24 h, the number of fragmented nuclei increased in a dose-dependent manner compared to the non-treated group (indicated by white arrows in Fig. 2a). To confirm the apoptotic pathway of paclitaxel in AGS cells, the number of early and late apoptotic cells were evaluated by flow cytometry using the Annexin V/propidium iodide (PI) staining assay. Annexin V stains apoptotic cells by binding to phosphatidylserine, a marker of apoptosis. PI stains necrotic or late apoptotic cells because it is internalized by cells that have lost plasma membrane and nuclear membrane integrity. Figure 3a,e reveals a significant and time-dependent increase in the percentage of early and late apoptotic cells following treatment with 20 nM paclitaxel for 24 and 48 h.

To confirm the involvement of the apoptotic mechanism in paclitaxel-induced cell death, western blot analysis was performed to detect the protein expression of the critical executioner apoptotic proteins procaspase-3 and cleaved caspase-3. Cells exposed to paclitaxel (40 and 80 nM) showed a significant increase (3.5- and 4.5-fold, respectively) in the protein expression of cleaved caspase-3 compared to the non-treated group (Fig. 2b). Additionally, the total and cleaved PARP protein expressions were evaluated by western blot analysis after paclitaxel treatment for 24 and 48 h. PARP is a well-known substrate of caspases, and its cleavage form is one of the hallmarks of apoptosis. Figure 2b,d shows a significant decrease in total PARP and induction of PARP cleavage by 1.7-, 2.0- and 2.3-fold at 24 h, and 1.7-, 2.0- and 2.3-fold at 48 h in cells treated with 40, 80 and 160 nM paclitaxel, respectively, compared to the non-treated group.

In the cells treated for 48 h, instead of the fragmented nuclei produced after 24-h treatment, the number of multinucleated cells increased (indicated by white arrows in Fig. 2c) in DAPI staining. To determine the involvement of the apoptotic mechanism in these multinucleated cells, the protein expressions of procaspase-3 and cleaved caspase-3 were detected by western blot analysis. In the cells treated with 40, 80 and 160 nM paclitaxel for 48 h, the protein expression of cleaved caspase-3 increased significantly by 7.0-, 13- and 40-fold, respectively, compared to the non-treated group (Fig. 2d). zVAD-fmk was used as a caspase inhibitor to determine the protein expression of cleaved caspase-3. Figure 2e shows the protein expression of cleaved caspase-3 in 40-nM paclitaxel-treated cells was inhibited by pretreatment with 20 µM zVAD-fmk and in 80-nM paclitaxel-treated cells by 50 and 100 µM zVAD-fmk but it was not significantly different from procaspase-3. These results indicate that the paclitaxel-induced formation of multinucleated giant cells involves an apoptotic mechanism.

### Paclitaxel induces apoptosis independent of cytochrome c release from mitochondria in AGS cells

To identify the paclitaxel-induced apoptotic mechanism in AGS cells, the apoptotic-related proteins were determined by western blot analysis. When AGS cells were treated with the indicated concentrations of paclitaxel, caspase-9 was cleaved significantly into an active dimer form following the 48-h treatment but showed no significant change after the 24-h treatment (Fig. 3b,f). zVAD-fmk (50 µM) was used as a caspase inhibitor. It inhibited the induction of caspase-9 cleavage in the cells treated with 80 nM paclitaxel. Involvement of mitochondrial permeabilisation in activation of caspase-9 was checked by detecting the pro-apoptotic protein Bax and the anti-apoptotic protein Bcl-2. Compared to the corresponding protein expressions in the non-treated group, Bax increased and Bcl-2 decreased after the 24- and 48-h treatments (Fig. 3c,g).

To evaluate the involvement of mitochondria in caspase-9 activation, the release of cytochrome *c* from the mitochondria to cytosol was evaluated by western blot analysis after mitochondrial fractionation and subsequent detection of cytochrome *c* protein expression in the mitochondria and cytoplasm, separately. Although cytochrome c protein expression in whole-cell lysates increased significantly following the 24-h treatment (Fig. 3c,g), it did not change significantly after the 48-h treatment compared to the non-treated group. After mitochondrial fractionation, only a small amount of cytochrome *c* was detected in the cytosol, despite large amounts detected in the mitochondria (Fig. 3d,h).

### Paclitaxel induced apoptosis without activation of caspase-8

**Figure 4:**
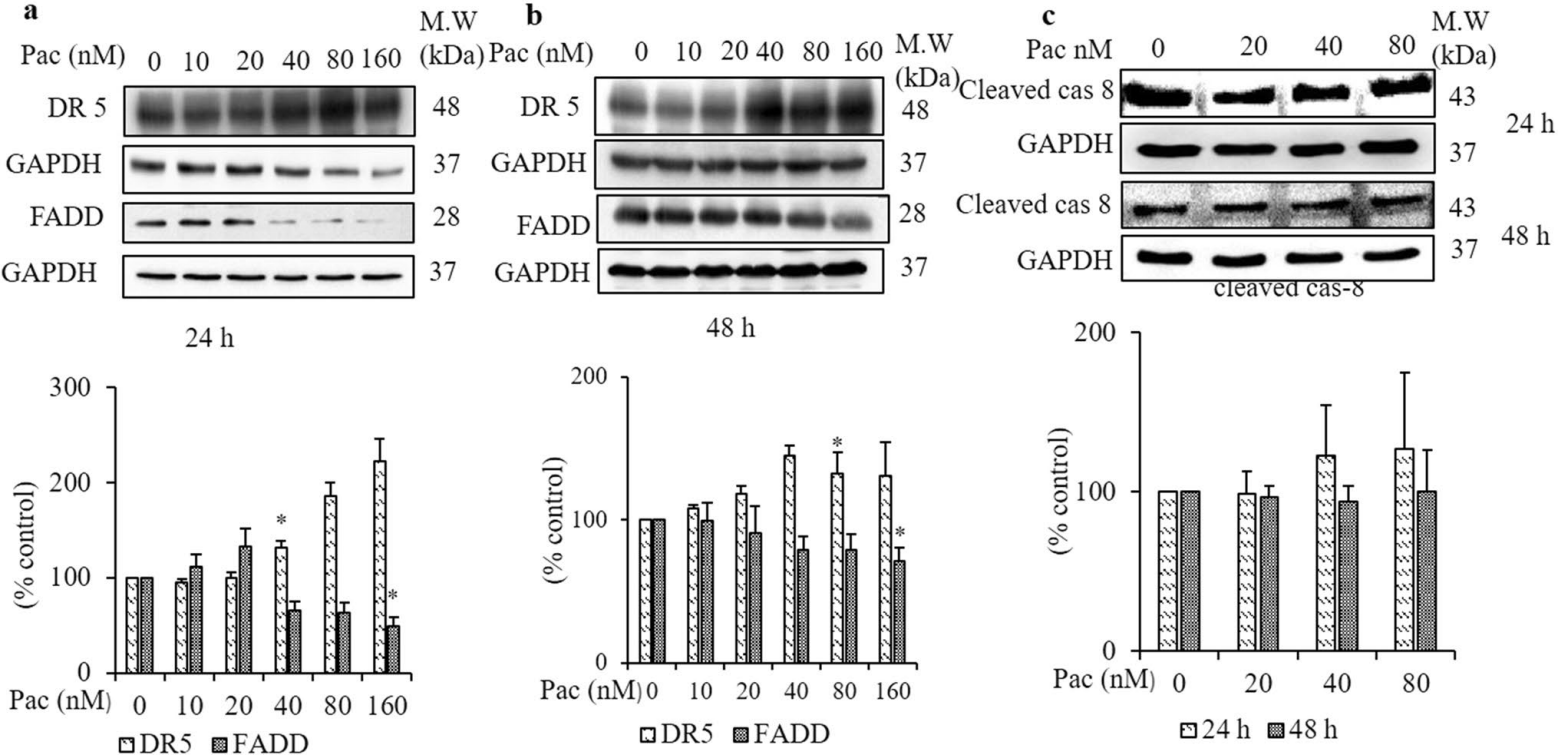
The effect of paclitaxel on the TRAIL receptor DR5, FADD and caspase-8 on AGS cells after 24- and 48-h treatments. AGS cells were treated with paclitaxel at the indicated concentrations for 24 and 48 h. The protein expressions of DR5 and FADD (**a**) 24 h, (**b**) 48 h and that of (**c**) cleaved caspase-8 were determined by western blot analysis. GAPDH was used as the loading control. The blots were quantified by ImageJ software. The full-length gel blots were described in Supplementary Fig. C. Data are presented as the mean ± SEM, *n* = 3. (*P < 0.05 compared to the non-treated group).

After noting that mitochondrial permeabilisation was partially involved in caspase-9 activation caused by paclitaxel, the role of death receptors was checked by detecting death receptor 5 (DR5) and the Fas-associated death domain (FADD) in western blot analysis. DR5 protein expression increased significantly in AGS cells treated with 40 nM paclitaxel for 24 h and 80 nM paclitaxel for 48 h (Fig. 4a,b). By contrast, FADD protein expression decreased significantly in both the 24- and 48-h treatments compared to the non-treated group. Continuously, caspase-8 activation was evaluated by western blot analysis. In paclitaxel-treated cells, cleaved caspase-8 protein expression did not change compared to the non-treated group (Fig. 4c).

### Involvement of autophagy in paclitaxel-induced cell death mechanism after 24- and 48-h treatments

**Figure 5:**
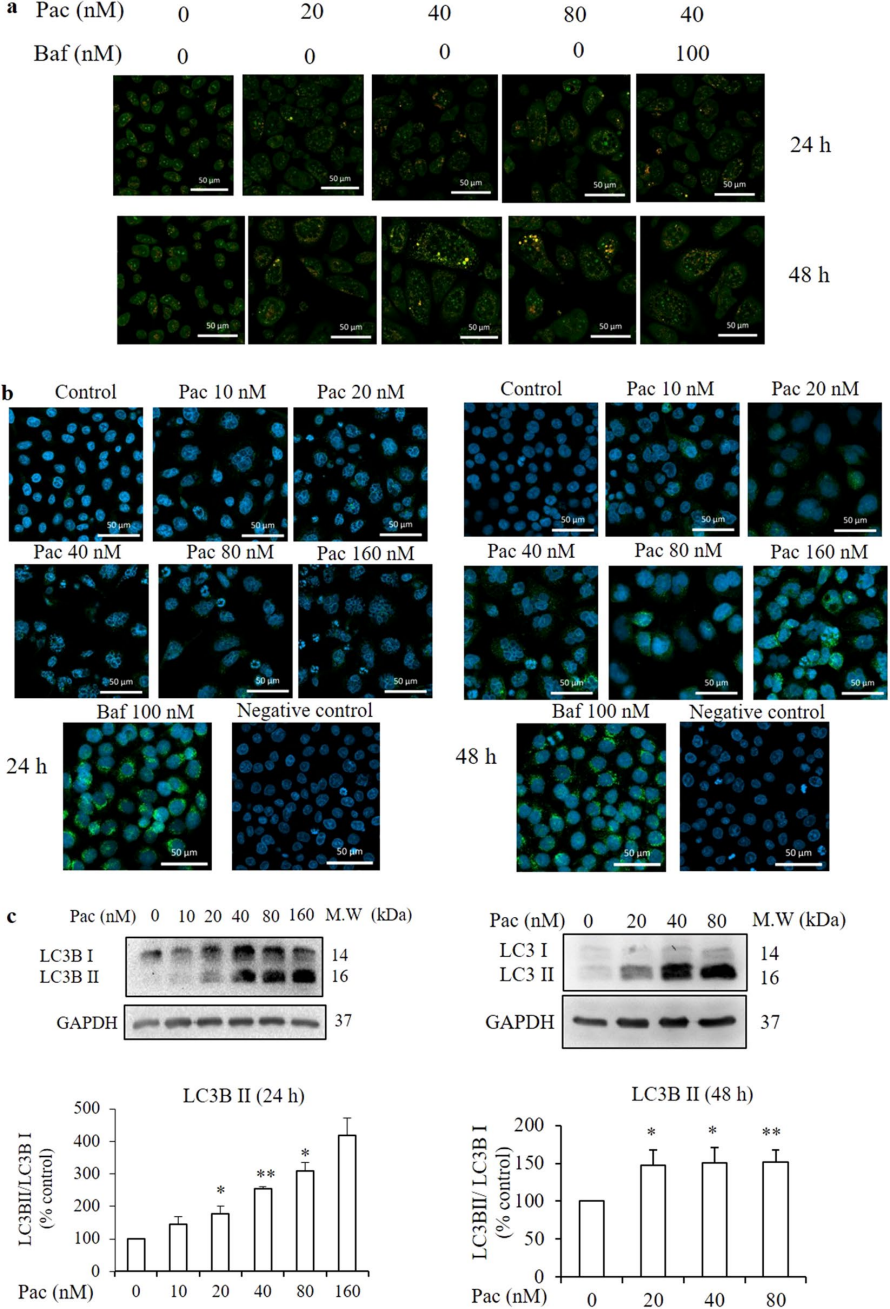

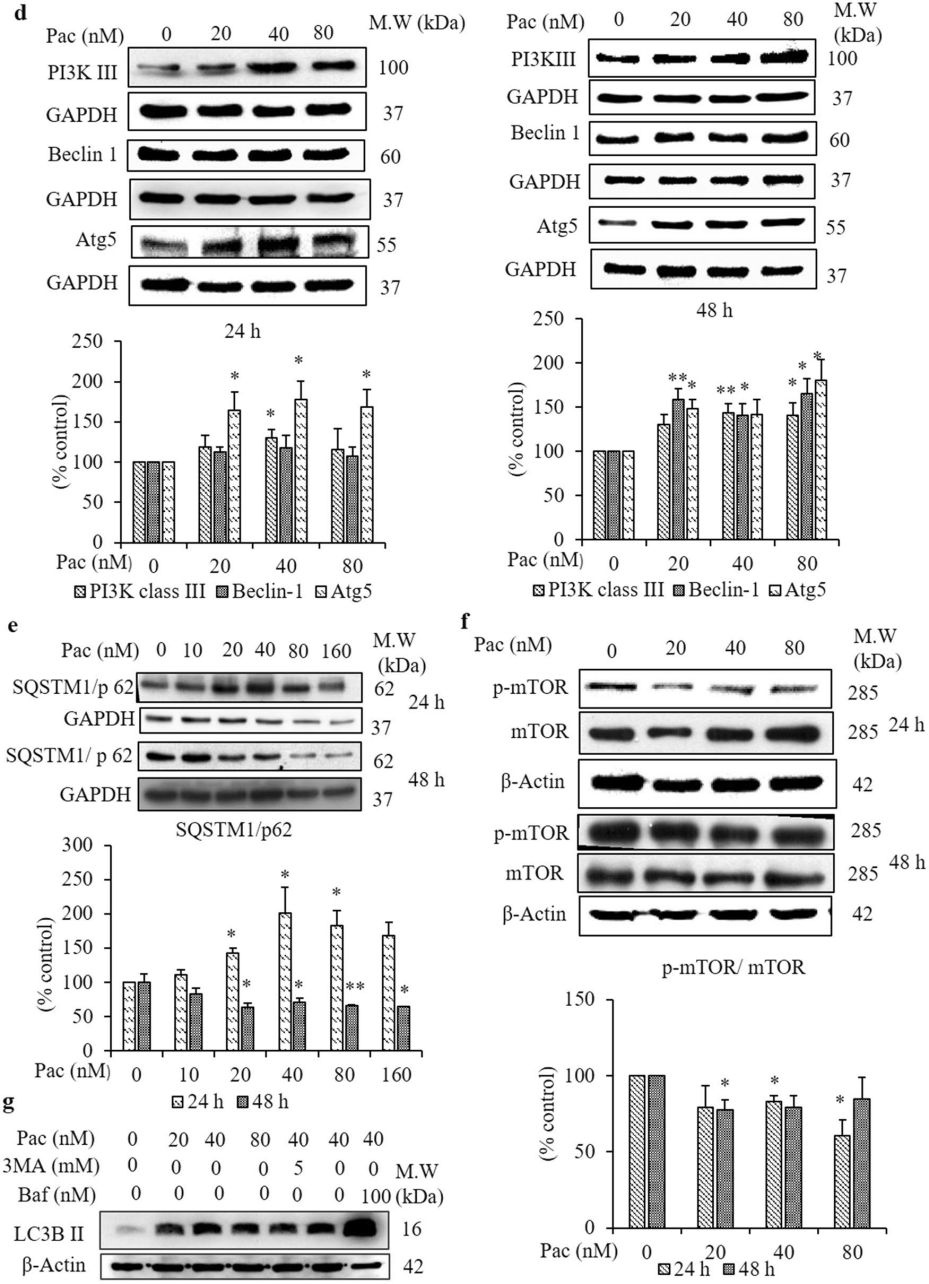
Involvement of autophagy process in the paclitaxel-induced cell death mechanism after 24- and 48-h treatments. (**a**) AGS cells were treated with paclitaxel for 24 and 48 h and then stained with acridine orange. The acidic compartments—such as autolysosomes (orange fluorescence)—and the cytoplasm and nucleus (green fluorescence) were evaluated by confocal microscopy (× 40). (**b**) LC3B protein expression level was determined by confocal microscopy (× 40) after immunofluorescence staining with LC3 primary antibody (green) and nuclear staining with DAPI (violet). (**c**) Protein expressions of LC3B and (**d**) autophagy-related proteins: class III PI3K, Atg5 and Beclin-1, (**e**) SQSTM1/p62 and (**f**) p-mTOR and mTOR were determined by western blot analysis. (**g**) Both the inhibition of LC3B-II by 3-MA (5 mM) and inhibition of lysosomal degradation by bafilomycin (Baf, 100 nM) were determined by western blot analysis. GAPDH was used as the loading control. The full-length gel blots were described in Supplementary Fig. D. The blots were quantified by ImageJ software. Data are presented as the mean ± SEM, *n* = 3. (*P < 0.05 and **P < 0.01 compared to the non-treated group).

Next, the involvement of autophagy in the paclitaxel-induced cell death mechanism was studied. In autophagy, the cells develop double-membraned, acidic vesicular organelles (AVOs, autophagosomes). First, acridine orange staining was performed to visualise the AVOs in paclitaxel-treated cells by confocal microscopy. The amount and intensity of orange fluorescence representing AVOs increased markedly in the paclitaxel-treated (40 and 80 nM for 24 and 48 h) cells compared to the non-treated group (Fig. 5a). Cells cultured in serum-starved medium and paclitaxel 40 nM treated cells which were pretreated with 100 nM bafilomycin, an inhibitor of autophagosome–lysosome fusion, served as the control to check the fluorescence intensity. Then, the protein expression of the autophagy marker, LC3B, in paclitaxel-treated AGS cells was determined by confocal microscopy with immunofluorescence staining. Figure 5b displays the increased green fluorescence, representing LC3B protein expression, in the paclitaxel-treated group compared to the non-treated one. In western blot analysis, LC3B-II protein expression increased significantly in cells treated with 20, 40 and 80 nM paclitaxel for 24 h (Fig. 5c). Continuously, autophagy-regulated proteins, including the PI3K-classIII, SQSTM1/p62, Atg5 and Beclin-1 protein expressions, were evaluated by western blot analysis. SQSTM1/p62 protein expression increased significantly in the 24 h-treated cells compared to the non-treated group, and the PI3K classIII and Atg5 protein expressions also increased significantly, but Beclin-1 expression was unaffected (Fig. 5d,e).

After the 48-h treatment, green fluorescence representing LC3B protein expression in giant multinucleated cells increased in the paclitaxel-treated group compared to the non-treated one (Fig. 5b). Figure 5c illustrates the significant increase in LC3B-II protein expression in the cells treated with 40 and 80 nM paclitaxel for 48 h. Figure 5d,e shows a significant decrease in SQSTM1/p62 protein expression after the 48-h treatment compared to the non-treated group. Beclin-1 expression and PI3K-III and Atg5 protein expressions also increased significantly. In addition, pretreatment with 5 mM of the class III PI3K inhibitor, 3-methyladenine (3-MA), decreased paclitaxel-induced LC3B-II protein expression. In the bafilomycin-pretreated cells, LC3B-II protein expression was increased compared to paclitaxel treatment alone or the non-treated group (Fig. 5g). To confirm paclitaxel-induced cell death in AGS cells, western blot analysis was performed to detect the cell proliferation protein marker—mTOR—and its phosphorylated form, p-mTOR. The protein expression of p-mTOR related to mTOR decreased significantly in cells treated with 40 and 80 nM paclitaxel for 24 h and 20 nM paclitaxel for 48 h when compared to the non-treated group (Fig. 5f).

### Paclitaxel induced mitotic catastrophe in AGS cells

**Figure 6:**
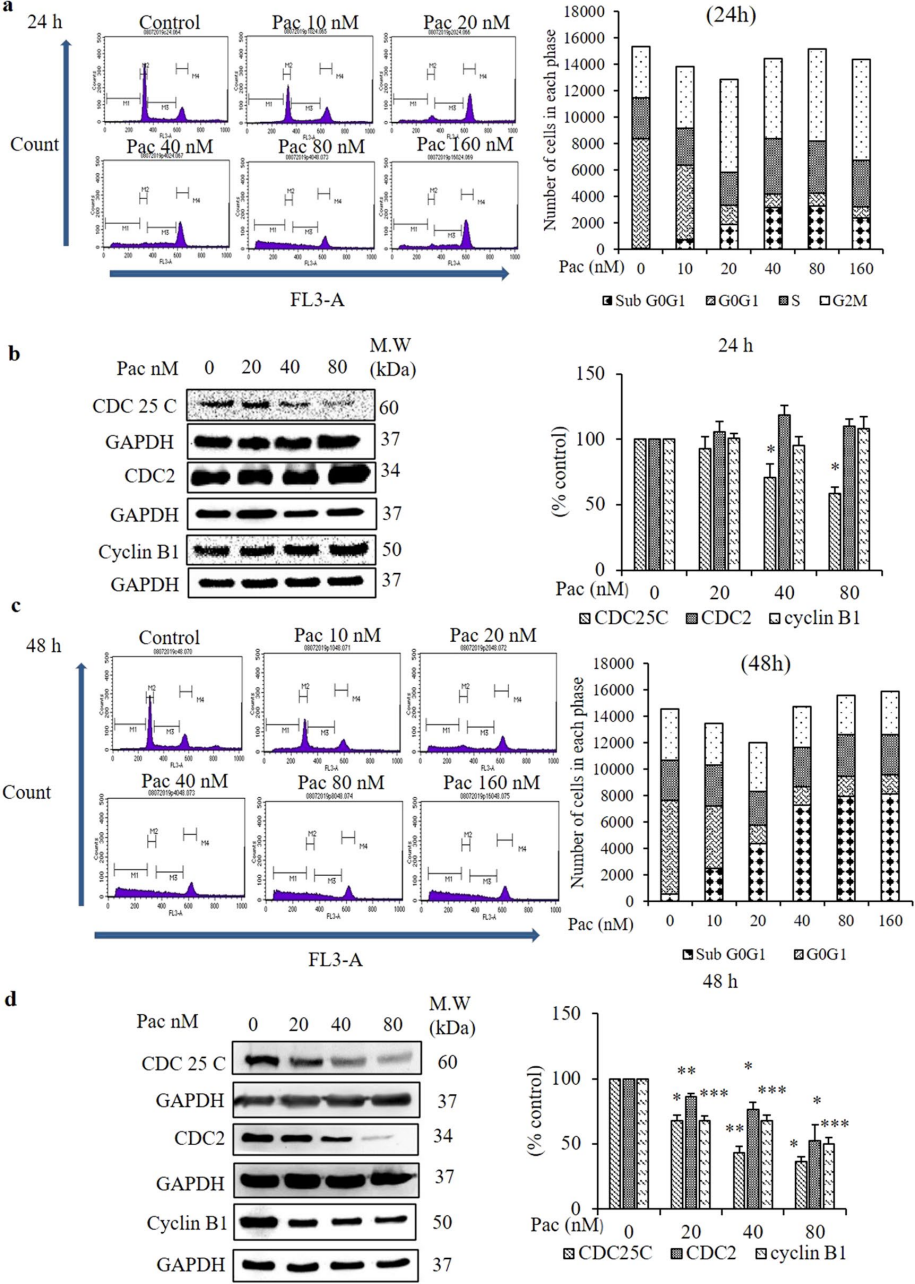
Effect of paclitaxel on cell cycle arrest in G2/M and cell division regulatory proteins after 24- and 48-h treatments. After treated with the indicated concentrations of paclitaxel for 24 and 48 h, (**a**,**c**) the cell cycle distribution of AGS cells was detected by PI staining and flow cytometry. (**b**,**d**) The protein expressions of cell division regulatory proteins (CDC25C, CDC2 and cyclin B1) were determined by western blot analysis. GAPDH was used as the loading control. The full-length gel blots were described in Supplementary Fig. E. The blots were quantified by ImageJ software. Data are presented as the mean ± SEM, *n* = 3. (*P < 0. 05 and **P < 0. 01 compared to the non-treated group).

Paclitaxel induces cell cycle arrest at G2/M, disturbs cell division and causes cell death. However, most of the paclitaxel-treated AGS cells became multinucleated (Fig. 2a,c). When multinucleated cells escape mitotic arrest, they may re-enter the cell cycle, and these cells become abundantly proliferated. To check the involvement of multinucleated cells in the cell division process, the number of cells in each cell cycle phase was evaluated by a cell cycle analysis using flow cytometry. After the 24-h treatment, the number of cells in G2/M increased compared to the non-treated group (Fig. 6a). By contrast, after the 48-h treatment, the cell populations increased in sub-G0/G1 (Fig. 6c).

To determine whether these cells in G2/M continue the cell division process, cell division regulatory proteins were checked by western blot analysis. Despite a significant decrease in CDC25C protein expression, the cyclin B1 and CDC2 protein expressions did not change significantly after the 24-h treatment relative to the non-treated group (Fig. 6b). However, all three protein expression levels decreased significantly after the 48-h treatment (Fig. 6d).

### Determination of the viability and cellular senescence of multinucleated cells cultured in fresh media for 1 day after 48-h paclitaxel treatment

**Figure 7:**
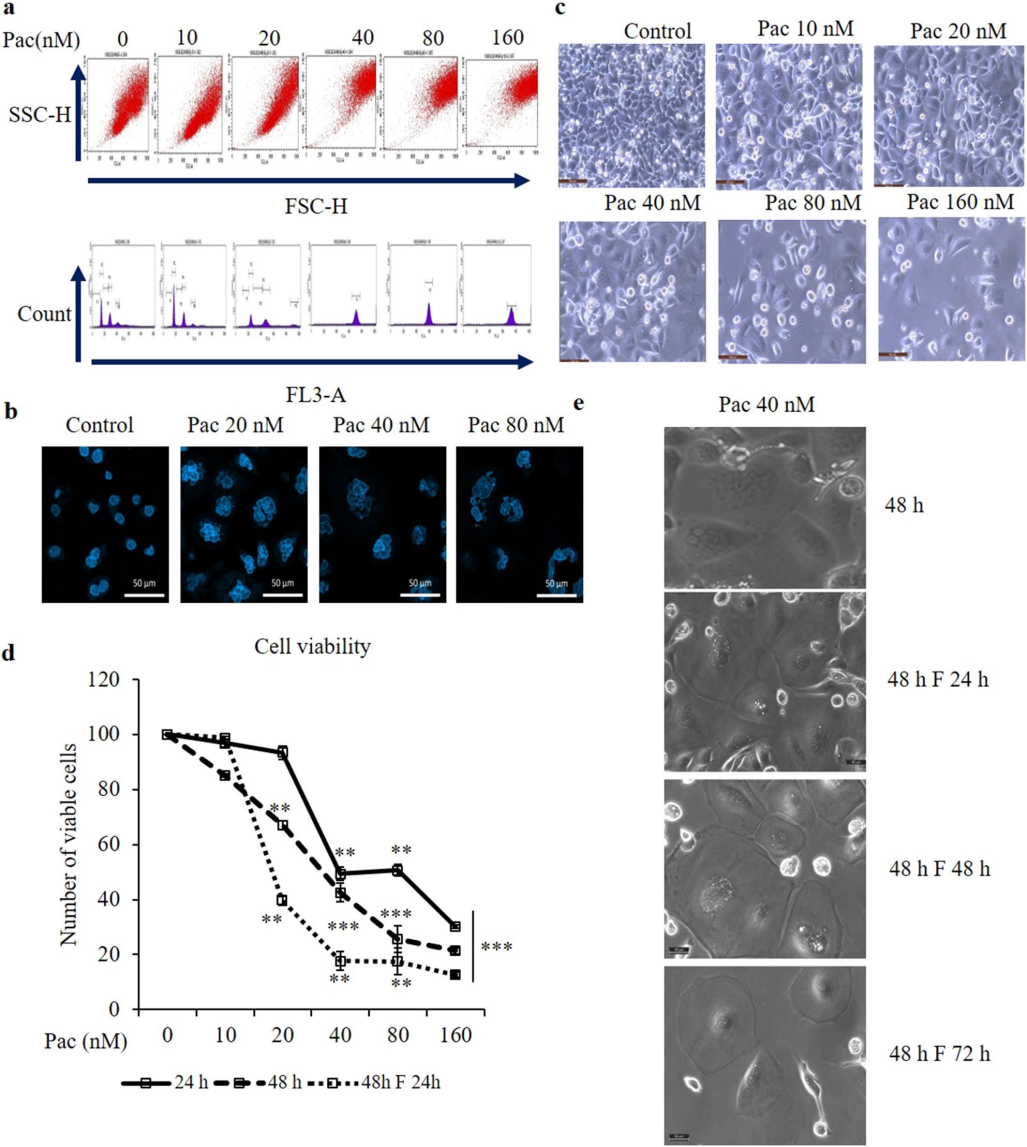

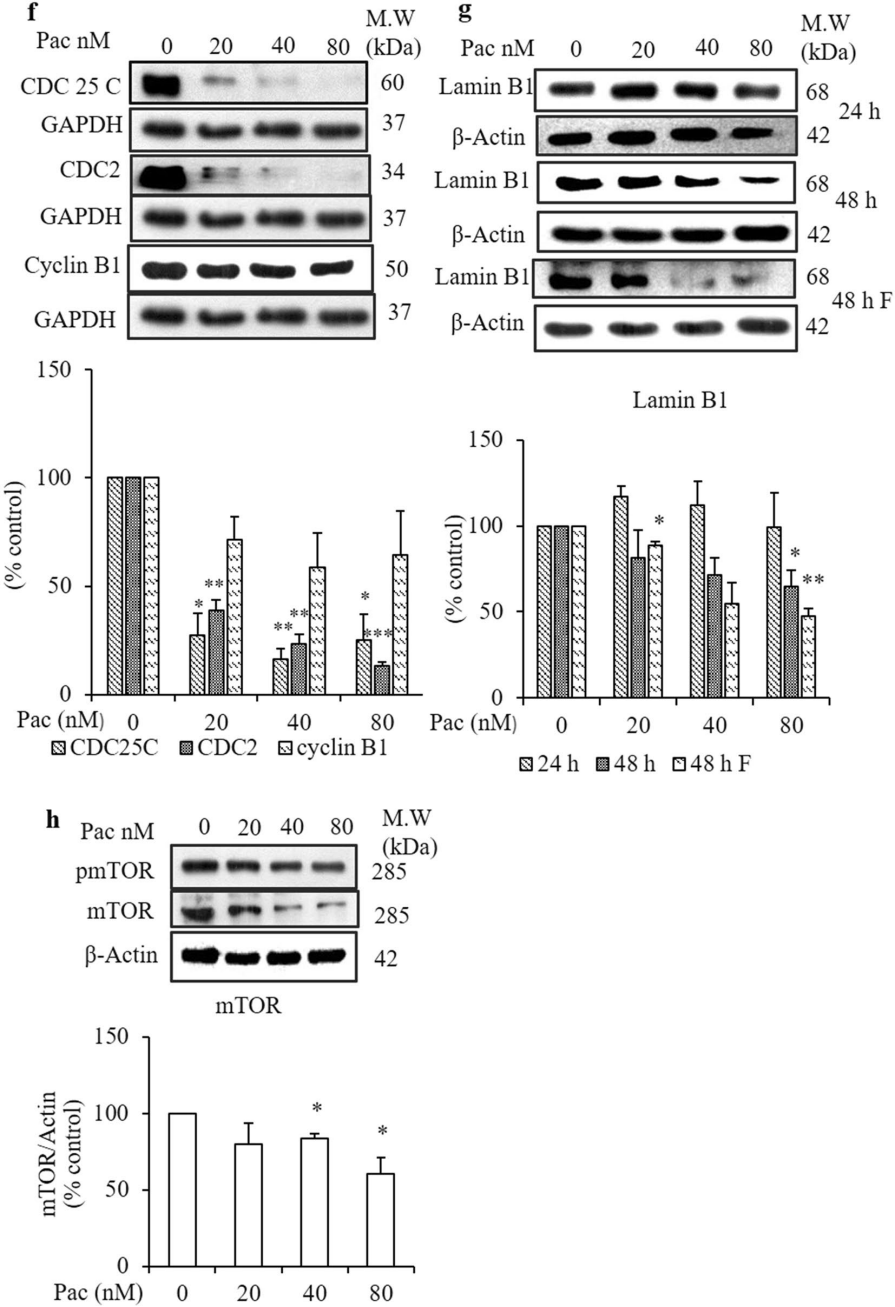
Determination of cell division process of multinucleated cells cultured in fresh media for 24 h after 48-h treatment with paclitaxel. After AGS cells were treated with paclitaxel for 48 h, the culture media was replaced with fresh media, and the cells were cultured for 24 h (48 F 24 h). (**a**) Cell cycle distribution of AGS cells was analysed by PI staining and flow cytometry. (**b**) Nuclear morphology was examined by confocal microscopy after DAPI staining. (**c**) Cell morphology was observed under an optical microscope (magnification × 20). (**d**) The graph compares the MTT assay of the percentage of viable cells in the non-treated group to the 24-h- and 48-h-treated cells and the 24-h-cultured cells after 48-h treatment. (**e**) The cellular senescence morphology of cells treated with 40 nM paclitaxel in time-dependent manner (**f**) Cell division regulatory proteins, CDC25C, CDC2 and cyclin B1 and (**g**) the biomarker protein of cellular senescence, lamin B1, and (**h**) p-mTOR and mTOR protein expressions were determined by western blot analysis. GAPDH and β-actin were used as the loading controls. The full-length gel blots were described in Supplementary Fig. F. Data are presented as the mean ± SEM, *n* = 3. (*P < 0.05, **P < 0.01 and ***P < 0.001 compared to the non-treated group).

To determine the cell cycle re-entry of multinucleated cells after a 48-h treatment with paclitaxel, the remaining attached cells were cultured in fresh media for 24 h and then stained with PI for analysis of the cell cycle distribution by flow cytometry. The increasing intensity of the forward scatter and side scatter observed in the cytograph and histogram indicated that the size and granularity of the remaining cells increased compared to the non-treated group and accumulated in G2/M (Fig. 7a). Moreover, confocal microscopy after DAPI staining showed the accumulated cells were multinucleated (Fig. 7b). When the protein expressions of cell division regulatory proteins in these multinucleated cells were analysed by western blot analysis, CDC2 and CDC25C protein expressions had decreased significantly, and cyclin B1 protein expression was not significantly different from the non-treated group (Fig. 7f). To confirm the cellular senescence mechanism of paclitaxel-treated multinucleated cells, first, AGS cells were cultured for 24 h in fresh media after 48-h paclitaxel treatment, and cell viability was evaluated by visualising by a light microscope and the MTT assay (Fig. 7c,d). Cell viability decreased significantly compared to the non-treated group and the 24- and 48-h treatments without the additional cell culture (Fig. 7d). To confirm the cellular senescence mechanism, after the 48 h treated cells were cultured in fresh media for next 24 h, 48 h and 72 h, their morphological characteristics were checked on light microscope (Fig. 7e). As described in Fig. 7e, the giant multinucleated cells are getting smaller and shrink. Next, western blot analysis was performed to detect the protein marker of cellular senescence, lamin B1. Lamin B1 protein expression decreased significantly after the 48-h treatment and in the 24-h-cultured cells after a 48-h paclitaxel treatment compared to the non-treated group (Fig. 7g). Despite no significant change in the protein expression of p-mTOR, the mTOR protein expression was reduced significantly compared to the non-treated group (Fig. 7h).

### Summary of paclitaxel-induced cell death mechanism in AGS cells

**Figure 8:**
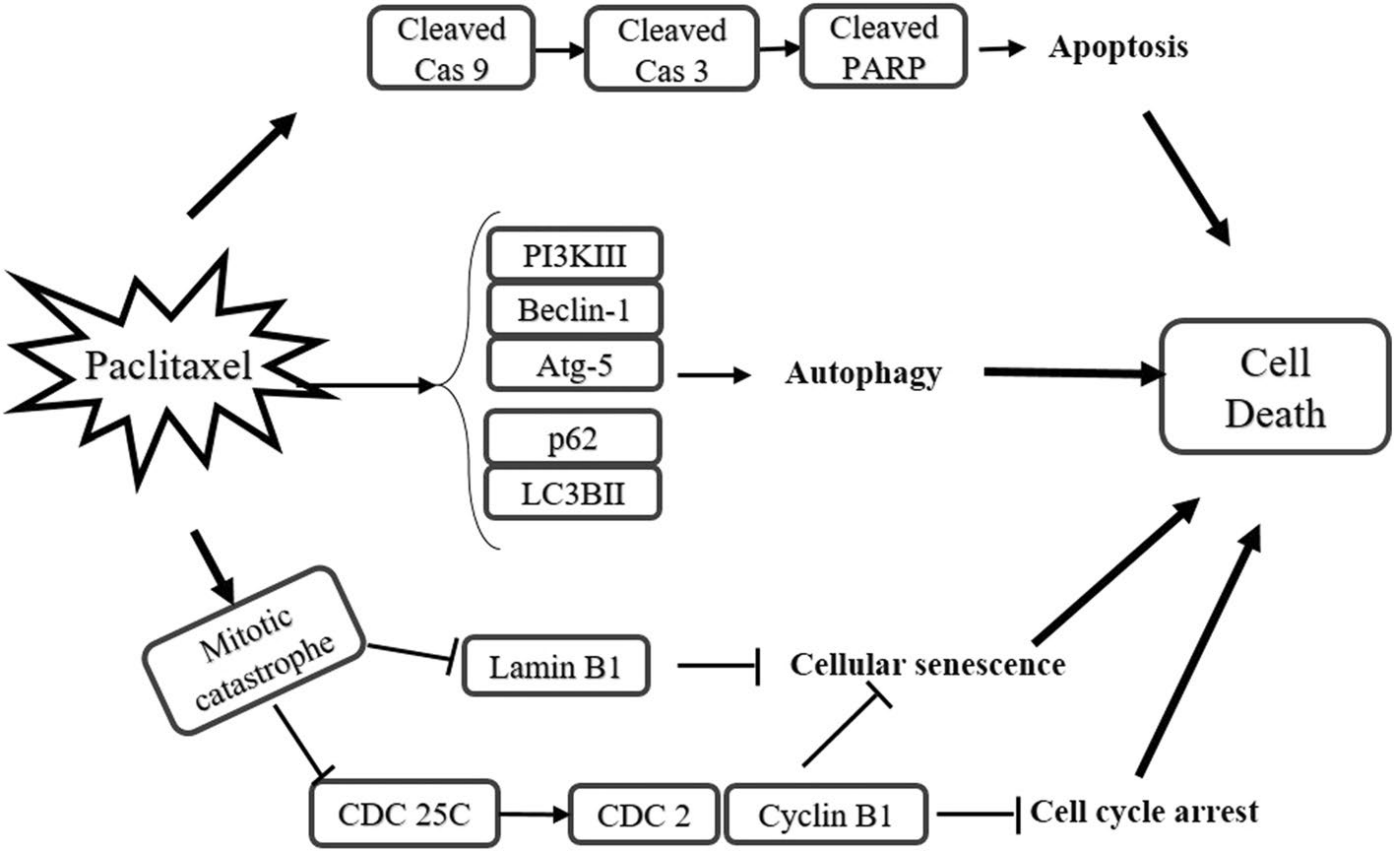
Summary of paclitaxel-induced cell death mechanism in AGS cells.

Paclitaxel induced apoptosis, autophagy and mitotic catastrophe in AGS cells (Fig. 8). The western blot result showed that the cleaved forms of caspase-3, caspase-9 and PARP were induced significantly, indicating apoptosis. This observation was further supported by the increase in the early and late apoptotic cells, as demonstrated by Annexin V/PI flow cytometry. Furthermore, immunofluorescence staining and western blotting revealed a significant increase in the protein expression of LC3B—an autophagy marker—and induction of Atg5, class III PI3K and Beclin-1, demonstrating the involvement of autophagy in paclitaxel-treated AGS cells. In confocal microscopy, DAPI staining showed that paclitaxel induced mitotic catastrophe, resulting in the formation of multinucleated cells after a 48-h treatment. According to the cell cycle and western blot results, the accumulated multinucleated giant cells were arrested in G2/M, and there was a significant decrease in cell division regulatory proteins—CDC2, CDC25C and cyclin b1—verifying the induction of cell death. After 48-h paclitaxel treatment, the remaining cells were cultured in fresh medium for 24 h, and CDC2 and CDC25C protein expressions were determined. The observed decrease in these cell cycle division proteins and the cellular senescence biomarker lamin B1 indicated that the accumulated cells in G2/M were senescent. In conclusion, the paclitaxel-induced cell death mechanism includes apoptosis, autophagy and mitotic catastrophe, which trigger cell death by the cellular senescence mechanism in AGS cells.

## Discussion

The present study suggests that paclitaxel induces cell death through apoptosis, autophagy and mitotic catastrophe in AGS cells. First, the paclitaxel-induced apoptotic pathway was characterised through a series of functional measurements. Second, the involvement of autophagy was analysed in paclitaxel-treated AGS cells. Third, the present study found that paclitaxel induced mitotic arrest and cellular senescence through mitotic catastrophe.

The cytotoxicity of paclitaxel is dependent on the cell type. In the present study, the IC_50_ of paclitaxel was 40 nM in AGS cells after 24 h. It has been reported that mTOR activation induces cell survival and leads to drug resistance^29^. In the present study, paclitaxel decreased the phosphorylation of mTOR significantly. From this result, it was suggested that the inhibition of mTOR activation was involved in the anti-proliferative effect of paclitaxel in AGS cells.

Reports show that the nature of the paclitaxel-induced apoptotic pathway varies among cancer cells in vitro. It was mediated by down-expression of Bax and up-expression of Bcl-2 in SGC-7901 human gastric carcinoma cells^9^, via caspase-independent routes in NCI-H460 non-small cell lung cancer cells^8^ and through the mitogen-activated protein kinase pathway^30^. In the present study, paclitaxel increased the expression of cleaved caspase-3—the critical executioner apoptotic protein—and induced PARP cleavage. Paclitaxel was found to induce apoptosis through PARP cleavage by caspase-3 activation in ovarian cancer, non-small lung cancer and leukaemia cells^31–34^. These findings are consistent with the present result. Therefore, it is suggested that the paclitaxel-induced apoptotic mechanism involves PARP cleavage and caspase-3 activation in AGS cells.

As mentioned above, there are two apoptotic pathways: intrinsic and extrinsic pathways. In the present study, the Bax/Bcl-2 protein expression ratio and cleaved caspase-9 were induced significantly by paclitaxel. Although cytochrome *c* protein expression of whole-cell lysates also increased significantly, only small amounts of cytochrome *c* were detected in the cytosol. Paclitaxel has been shown to induce apoptosis through the intrinsic pathway in breast cancer cell lines, the coronary artery smooth muscle cell line and leukaemia cell lines^14,35–38^. In the present study, it was suggested that the paclitaxel induced apoptosis in AGS cells was independent of cytochrome c release form mitochondria.

Paclitaxel increased DR4 and DR5 protein levels and induced apoptosis of human prostate cancer cells^39^. Yet, when a small amount of FADD was recruited to DISC, it was insufficient to activate caspase-8 to induce apoptosis^40,41^. Likewise, the present finding demonstrated that although DR5 protein expression increased significantly following paclitaxel treatment, the expression of FADD decreased significantly, while that of cleaved caspase-8 did not change. Therefore, it was suggested that caspase-8 was not involved in caspase-3 activation of paclitaxel-induced apoptosis. In one study, the researchers revealed that the apoptotic mechanism induced by etoposide in Cell Line P39 was independent of cytochrome c release and caspase-3 was activated by lysosomal enzymes. In our study, there is limitation to detect lysosomal enzymes for caspase-3 activation and it will be examined in future study.

Autophagy plays an important regulatory role in cell survival and cell death, depending on the cell environment and cell type. In the present study, paclitaxel induced LC3B activation, as evidenced by the enhanced intensity of green fluorescence when the cells were exposed to immunofluorescence staining using anti-LC3B antibody, and expression of the LC3B-II/LC3B-I ratio was increased in western blot analysis. In addition, pretreatment with 5 mM 3-MA, the class III PI3K inhibitor^42^, decreased paclitaxel-induced LC3B-II protein expression. From these results, it was suggested that paclitaxel induced autophagy in AGS cells.

In the bafilomycin pretreated cells, LC3II protein expression was increased compared to paclitaxel treatment alone and the non-treated group. It was assumed that when bafilomycin blocked the fusion of autophagosomes with lysosomes, LC3B-II proteins accumulated instead of degraded^43^. The degradation of p62 is a meaningful approach to monitor autophagy because p62 is selectively degraded by autophagy after binding to LC3B^44^. In the present study, the increased expressions of autophagy-related proteins—class III PI3K, Atg5 and Beclin-1—and the decreased expression of membrane protein SQSTM1/p62 confirmed that 48-h paclitaxel treatment induced the autophagy process. Apoptosis and autophagy induction can occur in the same cell, and there is cross-talk between these processes^45^. The present findings supported such phenomena, and it was assumed that paclitaxel induced autophagy, in addition to apoptosis, in AGS cells. Autophagy has been described as a potential cancer suppressor or cancer promoter, depending on the type of cancer cell. However, modulating autophagy remains challenging in the context of clinical translatability for improved therapeutic approaches^46–48^ Prolonged mitotic arrest caused by abrogation of cyclin B1 degradation promotes cell death by autophagy^49^. Based on these findings, the decreased expressions of mTOR and cyclin B1 in the present study indicated that the paclitaxel-induced cell death mechanism involved the autophagy pathway.

The morphological markers of mitotic catastrophe are multinucleation and/or micronucleation. The giant multinucleated cells are formed from clusters of mis-segregated uncondensed chromosomes^18^. Mitotic block is induced in HeLa cells at low concentrations of paclitaxel, but at higher concentrations, some cells exit mitotic arrest and become abnormal multinucleated cells^50^. Similarly, most of the AGS cells treated with paclitaxel for 48 h were found by DAPI staining to form multinucleated giant cells in the current study. Paclitaxel has been shown to induce multinucleation in several cancer cell lines, such as ovarian, breast, leukaemia^51–53^. Following activation of mitotic catastrophe, cells arrested in mitosis have three fates; mitotic death in the presence of cyclin B; or cyclin B levels will gradually fall, allowing the cells to undergo slippage and exit mitosis where they subsequently undergo death in G1 or cells undergo senescence following slippage^20^.

Paclitaxel inhibits microtubule depolymerisation, causes cell cycle arrest at G2/M and suppresses tumour growth^54^. The current study observed an increase in the number of cells in G2/M at paclitaxel concentrations ≥ 20 nM. For the 24-h treatment, even though paclitaxel decreased CDC25C protein expression, the cyclin B1 and CDC2 protein expressions did not change. It was suggested that the 24 h-treated cells were arrested in mitosis and then underwent mitotic catastrophe-induced death in the presence of cyclin B1. Treatment with paclitaxel for 48-h decreased the protein expressions of CDC2, CDC25C and cyclin B1 significantly, as revealed by western blot analysis, and increased the number of cells in sub-G0/G1. It was assumed that some of the accumulated cells in G2/M escaped from mitosis and were dead in G1. To confirm whether these 48 h-treated long-arrested cells trigger cell death, these cells were grown in fresh media for another 24 h. Cell cycle analysis showed that giant multinucleated cells were accumulated in G2/M. Furthermore, the expressions of cell division regulatory proteins CDC2 and CDC25C were diminished significantly, along with the protein expression of lamin B1, the biomarker of cellular senescence. It was assumed that the cellular senescence mechanism was involved in the paclitaxel-induced mitotic catastrophe. It was concluded that paclitaxel induced cell death and cellular senescence by mitotic catastrophe in AGS cells. Moreover, the number of viable cells was decreased significantly compared to the non-treated group and the 24- and 48-h treatments without their additional 24-h culture. From these results, it was suggested that paclitaxel induced cell death by cellular senescence. Some reports mention that autophagy is involved in the execution of cell cycle-exit programs, particularly senescence^55–57^. In the present study, there is a limitation to suggest the relationship between senescence and autophagy.

In conclusion, the current study proved the involvement of mitotic catastrophe in the paclitaxel-induced cell death mechanism. The resulting multinucleated cells became senescent through the down-regulation of cell division regulatory proteins—CDC2 and CDC25C—and the biomarker of cellular senescence, lamin B1. Moreover, it is suggested that the senescence cells are finally dead by degradation of mTOR and decreasing the cell viability. Paclitaxel is reported to trigger drug resistance and aneuploidy or polyploidy because of aberrant mitosis if its cell death mechanism is dependent only on apoptosis^58–60^. The paclitaxel-induced cell death mechanism in the present study involved not only apoptosis but also the autophagy pathway. Although the paclitaxel-induced apoptotic mechanism in AGS cells involves the cleavage of PARP and caspase-3 by the activation of cleaved caspase-9, it is not related to the release of cytochrome c from mitochondria, and caspase-8 is not involved in the apoptotic mechanism. However, due to a lack of study on the inter-relationship among apoptosis, autophagy and mitotic catastrophe, it is difficult to evaluate whether multinucleation precedes apoptosis and autophagy. Despite its limitations, the present study supports that paclitaxel-induced mitotic catastrophe is an integral part of the cell death mechanism, in addition to apoptosis and autophagy, in AGS cells.

## Materials and methods

### Materials

Paclitaxel was purchased from Sigma-Aldrich (St. Louis, MO, USA). The human gastric cancer cell line AGS (gastric adenocarcinoma, KCLB 21739) was obtained from the Korean Cell Line Bank (Seoul, Korea). Materials used in the cell culture process, such as Roswell Park Memorial Institute (RPMI) 1640 medium, Dulbecco’s phosphate-buffered saline (DPBS) and foetal bovine serum (FBS), were purchased from Welgene, Inc. (Gyeongsan-si, Gyeongsangbuk-do, South Korea). Antibiotic solutions and trypsin–EDTA 0.25% were purchased from Thermo Fisher Scientific (Waltham, MA, USA). For the cell viability process, MTT and dimethyl sulphoxide (DMSO) were purchased from Tokyo Industry Chemical Co., Ltd. (Tokyo, Japan).

For western blot analysis, the primary antibodies, including anti-mTOR, anti-p-mTOR, anti-Bcl2, anti-DR5, anti-PARP, anti-caspase-3, anti-cleaved caspase-3, anti-caspase-8, anti-cleaved caspase-8, anti-caspase-9, anti-cleaved caspase-9, anti-LC3B, anti-SQSTM1/p62, anti-PI3K-III complex, anti-Beclin 1, anti-CDC2, anti-CDC25C, anti-cyclin B1 and lamin B1, were purchased from Cell Signaling Technology (Danvers, MA, USA). Anti-actin, anti-GAPDH and cytochrome *c* antibodies were purchased from Santa Cruz Biotechnology, Inc. (Santa Cruz, CA, USA). The goat anti-rabbit IgG-horse radish peroxidase (HRP) and goat anti-mouse IgG-HRP secondary antibodies were purchased from Bethyl Laboratories (Montgomery, TX, USA). zVAD-fmk, a pan-caspase inhibitor, was purchased from Selleckchem, Munich, Germany. Enhanced chemiluminescence (ECL) solutions and acrylamide were purchased from Elpis Biotechnology (Daejeon, Korea). The protein marker was purchased from Bio-Rad (Hercules, CA, USA). For the apoptosis assay, the PI and Annexin V staining kits were purchased from Abcam (Cambridge, UK). For the cell cycle analysis, PI and the RNase solutions were purchased from BD Biosciences (San Jose, CA, USA).

### Cell culture process

The AGS cells were cultured in RPMI 1640 medium containing 10% FBS, 1% penicillin–streptomycin and 0.1% amphotericin B at 37 °C in a humidified atmosphere of 5% CO_2_ and 95% air.

### Cell viability assay

The effect of various paclitaxel concentrations (10, 20, 40, 80 and 160 nM) on cell growth was analysed by the MTT assay. In each well of a 24-well plate, 1 × 10^5^ cells were seeded and incubated at 37 °C for 24 h to reach 80% confluency. After 24-and 48-h treatments of the cells with the indicated paclitaxel concentrations, the medium was aspirated and washed with DPBS. Afterwards, 200 µl of MTT solution (0.5 mg/ml in PBS) was added to each well and incubated at 37 °C for an additional 3 h in a 5% CO_2_ incubator. Then, the MTT solution was removed, DMSO (200 μl) was added to each well, and the plate was shaken for 15 min to solubilise the MTT metabolic product, the formazan crystals. Continuously, 100 µl of the solution was added to the 96-well microplate. Next, the optical density (OD) was measured at 570 nm in a FlexStation 3 multimode microplate reader (Molecular Devices, San Jose, CA, USA), followed by IC_50_ calculation. The cell growth inhibitory rate was calculated as follows: (OD of control group − [OD of experimental group/OD of control group] − OD of blank group) × 100%.

### Western blot analysis

Twenty to fifty micrograms of protein from each sample was loaded onto a 5–15% SDS-PAGE gel. After electrophoresis, the proteins were electroblotted onto nitrocellulose membranes. Then the membranes were stained with Ponceau solution and cut to get the desired protein lanes by checking with molecular weight standard marker. The Ponceau solution was washed with distilled water and TBS/0.2% Tween-20 solution and then blocked with 5% non-fat dry milk or bovine serum albumin (BSA) in TBST solution for 1 h. After washing off the blocking solution, the membranes were incubated with the primary antibody (1:1000 dilution) at 4 °C overnight. Subsequently, the membranes were washed with TBST and incubated with a secondary antibody—HRP-conjugated goat anti-mouse or goat anti-rabbit—for 90 min. After washing the membrane with TBST, the protein expression was detected by the Bio-Rad Universal Hood II Gel doc system (Bio-Rad) or X-ray film method (Bio-Rad). ImageJ software (NIH, Maryland, USA) was used to quantify the protein expressions^61^. β-actin antibody or GAPDH antibody was used as the loading control.

### DAPI staining

To check the nuclear morphology, AGS cells were treated with the indicated concentrations of paclitaxel in a confocal disc and then both the non-treated and treated cells were washed with PBS and fixed with 4% paraformaldehyde in PBS for 30 min at room temperature. The fixed cells were washed again with PBS and stained with DAPI (Roche Diagnostics GmbH, Mannheim, Germany) solution at room temperature for 10 min. The cells were washed twice with PBS and detected under a confocal microscope.

### Cell cycle analysis

The AGS cells were treated with the indicated concentrations of paclitaxel in a 6-well plate for 24 and 48 h. After treatment, the cells were collected from the culture plates by trypsinisation. The collected cells were centrifuged, and the pellets were washed with cold PBS. The cells were subsequently fixed with ice-cold 70% ethanol and incubated at − 20 °C overnight. After washing the cells in cold PBS, they were incubated with 1 ml of PI/RNase staining solution at 20–25 °C for 25 min in the dark. The staining solution was stored on ice, and the distribution of cells in each cell cycle phase was measured by flow cytometry (BD FACS Calibur, BD Biosciences) using CellQuest Pro software.

### Annexin V/PI apoptotic assay

To identify the apoptotic cells and necrotic cells, the Annexin V-FITC/PI double-staining kit was used. After treatment with the indicated concentrations of paclitaxel in a 6-well plate for 24 and 48 h, the AGS cells were harvested and suspended in 500 μl of Annexin V binding buffer. To 100 µl of the cell suspensions, 5 μl of Annexin V-FITC and 5 μl PI were added and incubated for 10 min in the dark. Subsequently, the staining solutions were resuspended in 400 µl of Annexin V binding buffer and kept on ice during flow cytometry measurement. The apoptotic and necrotic cells were detected using the FL1 and FL2 detectors by flow cytometry. The cells were classified as healthy cells if double-negative (Annexin V-/PI-), necrotic cells if double-positive (Annexin V+/PI+), late apoptotic cells if Annexin V-/PI+ and early apoptotic cells if Annexin V+/PI−. The non-treated group was used as a negative control, and camptothecin (0.5 µM) served as a positive control to adjust the flow cytometry settings.

### Immunofluorescence staining

AGS cells were treated with the indicated concentrations of paclitaxel for 24 and 48 h on a confocal disc. The medium was aspirated and washed with 1× PBS. The attached cells were then fixed with ice-cold 100% methanol at − 20 °C for 15 min. The fixative was aspirated and rinsed three times with 1× PBS for 5 min each. Afterwards, the specimen was blocked in blocking buffer (1× PBS/5% normal serum/0.3% Triton X-100) for 60 min. The blocking solution was aspirated and washed three times with PBS. Then, the diluted primary antibody was applied and incubated overnight at 4 °C. The specimen was rinsed thrice with 1× PBS for 5 min each. The specimen was incubated in fluorochrome-conjugated secondary antibody diluted in antibody dilution buffer (1× PBS/1% BSA/0.3% Triton X-100) at room temperature for 1–2 h in the dark. Then, it was rinsed in 1× PBS and stained with DAPI for 10 min. After washing with PBS, the cells were detected under a confocal microscope.

### Mitochondrial fractionation

The cells were trypsinised and collected (5 × 10^7^) by centrifugation at 600×*g*, 4 °C for 5 min. The collected cells were washed with 10 ml of ice-cold PBS and centrifuged again under the same conditions. The supernatant was removed, and the cells were resuspended with 1.0 ml of 1× cytosol extraction buffer mix containing dithiothreitol (DTT) and protease inhibitors and incubated on ice for 10 min. The cells were homogenised by mixing with 22-gauge syringes on ice. The homogenate was transferred to a 1.5-ml microcentrifuge tube and centrifuged at 700×*g* (~ 3000 rpm) in a microcentrifuge at 4 °C for 10 min. The supernatant was carefully collected as the cytosolic fraction, and the pellet was discarded. The supernatant was transferred to a fresh 1.5-ml tube and centrifuged at 10,000×*g* (~ 13,000 rpm) in a microcentrifuge at 4 °C for 30 min. The supernatant was collected as the cytosolic fraction, and the pellet was resuspended in 100 µl of the mitochondrial extraction buffer mix containing DTT and protease inhibitors and vortexed for 10 s. It was defined as the mitochondrial fraction and stored at − 80 °C.

### Acridine orange staining for AVOs

Acridine orange staining was used to detect the formation of AVOs, such as autolysosomes, a morphological characteristic of autophagy. Cells were seeded and cultured in confocal dishes to 70% confluency, then treated with the indicated concentrations of paclitaxel and bafilomycin for 24 and 48 h. Acridine orange staining solution was prepared with the growth medium to a final concentration of 2 µg/ml and used to stain the non-treated and treated cells at 37 °C for 20 min. The cells were washed twice with PBS containing 3% FBS. Then, 1 ml of PBS solution containing 3% FBS was added to the disc and visualised under a confocal microscope (LSM 800, Carl Zeiss, Oberkochen, Germany). Zen system software (Image Software, Carl Zeiss) was used to capture the images.

### Statistical analysis

All data are expressed as mean ± standard error of the mean of 3–6 experiments. Statistical differences among the groups were analysed by One-way ANOVA and the Student’s *t*-test. Data were considered significant at *P* < 0.05.

## Supplementary Information

Supplementary Information 1.

Supplementary Information 2.

Supplementary Information 3.

## Data Availability

Western blot data are available in the Supplementary Information file.
