# Supplementary material for: The effect of paclitaxel on apoptosis, autophagy and mitotic catastrophe in AGS cells
Source: Sci Rep. 2021 Dec 6;11:23490. doi: 10.1038/s41598-021-02503-9 (PMC8648765; doi:10.1038/s41598-021-02503-9)

The effect of paclitaxel on apoptosis, autophagy and mitotic catastrophe in AGS cells

Tin Myo KHING, Won Seok CHOI, Dong Min KIM, Wah Wah PO, Wynn THEIN, Chang Yell SHIN and Uy Dong SOHN*

Laboratory of Signaling and Pharmacological Activity, Department of Pharmacology, College of Pharmacy, Chung-Ang University, Seoul, Republic of Korea

***Corresponding author:** Uy Dong Sohn, Professor, Ph.D., Department of Pharmacology, College of Pharmacy, Chung-Ang University, Seoul, 06974, Republic of Korea. Tel.: +82 28205614, Fax: 011 +8228268752, e-mail: udsohn@cau.ac.kr

Supplementary Fig. A

Fig. A Photographs of the full-length blots described in Figure 2

Fig. 2b

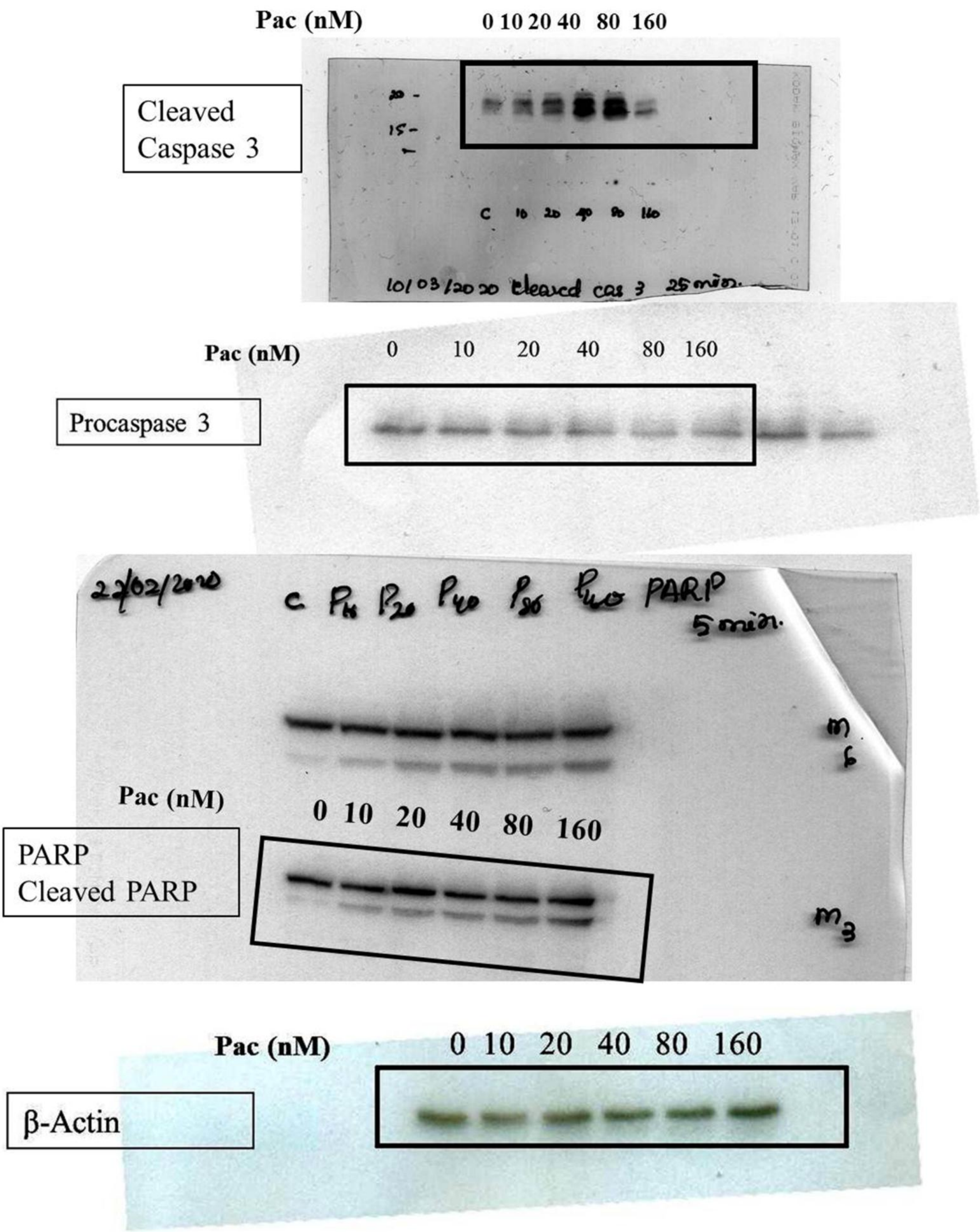

Fig.2d

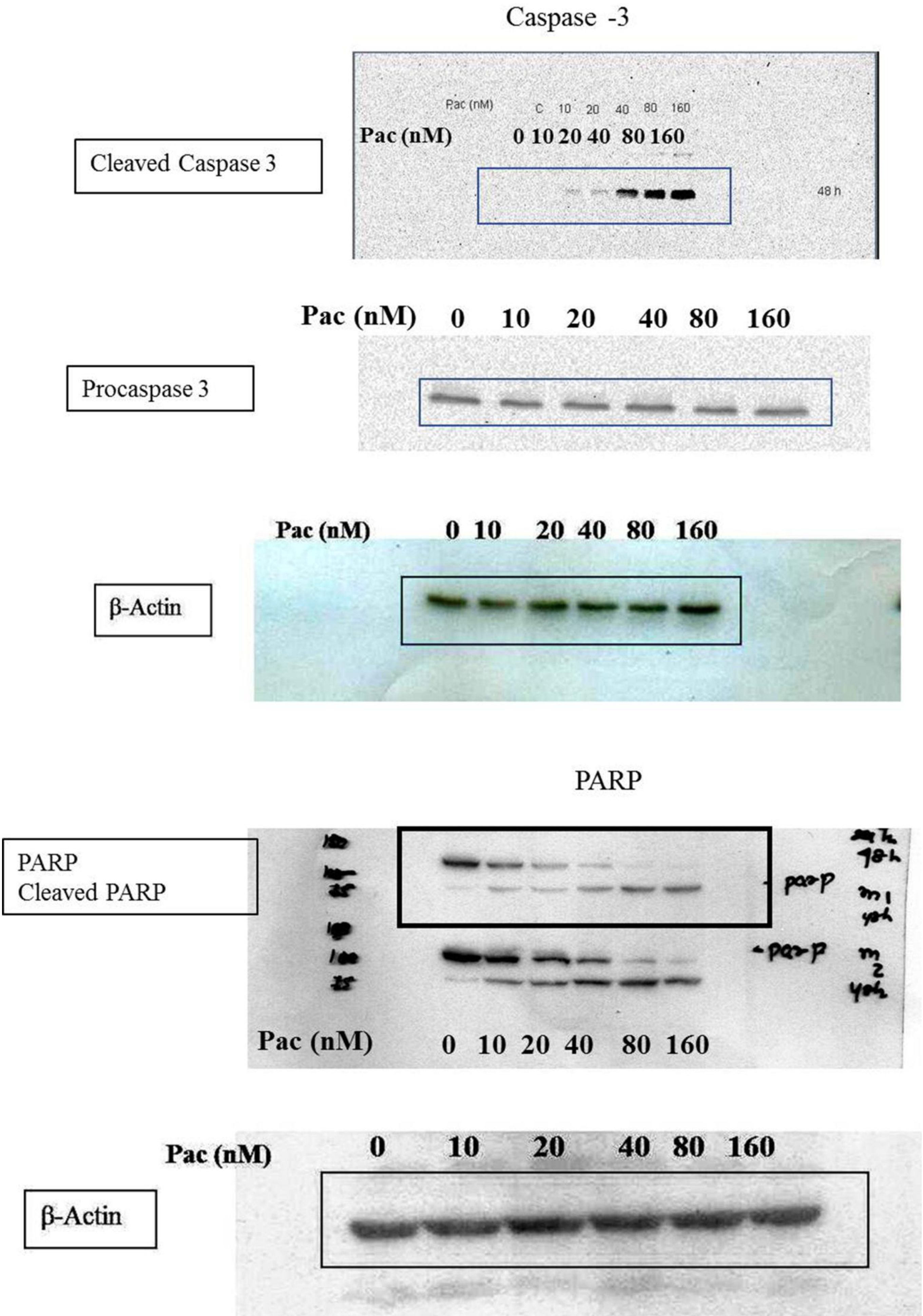
Caspase -3
Cleaved Caspase 3
Pac (nM) C 10 20 40 80 160
Pac (nM) 0 10 20 40 80 160
48 h
Pac (nM) 0 10 20 40 80 160
Procaspase 3
Pac (nM) 0 10 20 40 80 160
β-Actin
PARP
PARP
Cleaved PARP
Pac (nM) 0 10 20 40 80 160
Pac (nM) 0 10 20 40 80 160
β-Actin

Supplementary Fig. B

Fig. B Photographs of the full-length blots described in Figure 3

Fig. 3b

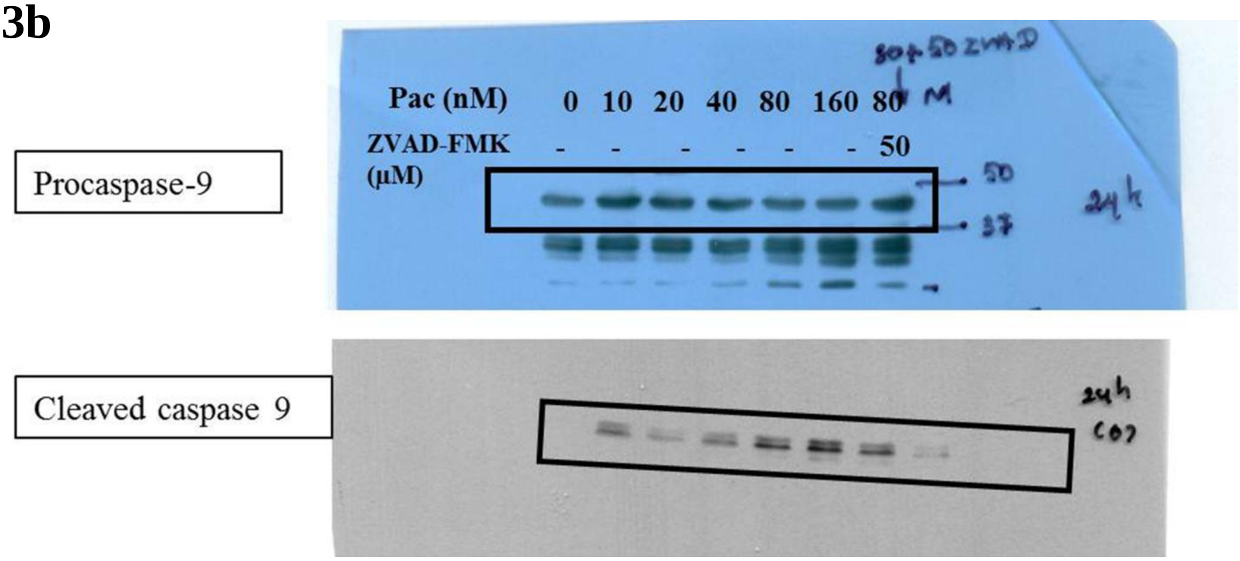

Fig. 3c

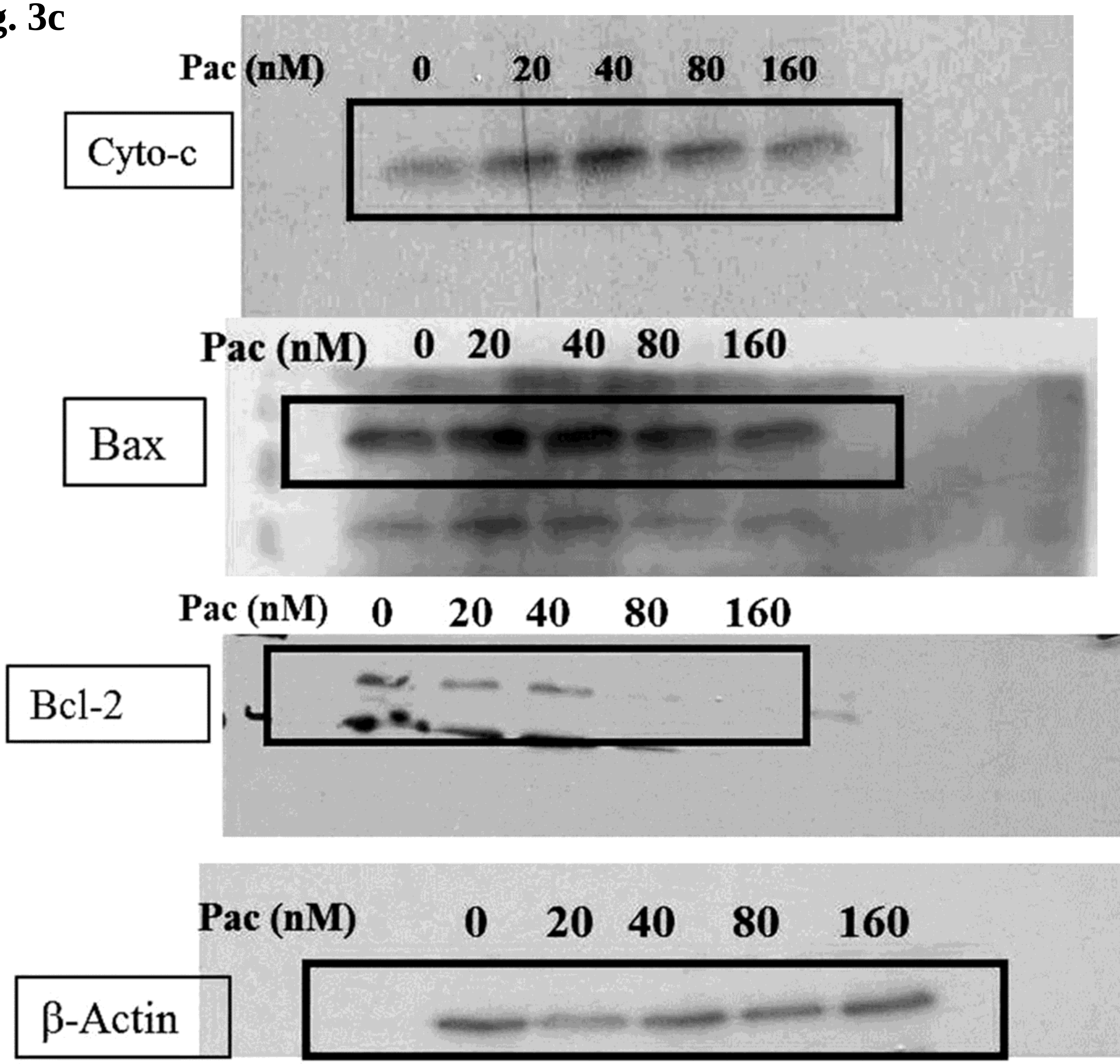

Fig.3d

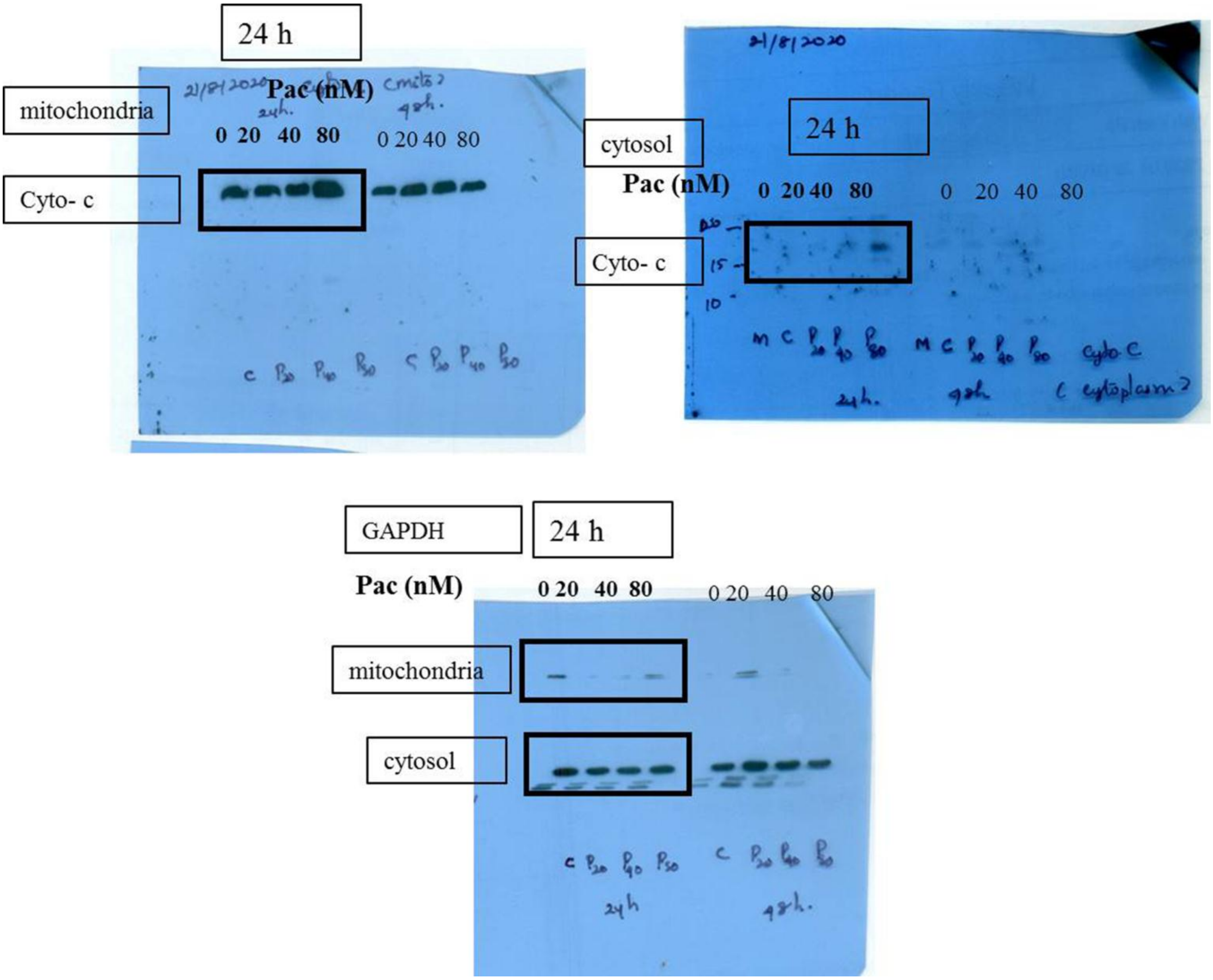

24 h
mitochondria
Pac (nM)
0 20 40 80
0 20 40 80
Cyto- c
21/8/2020
cytosol
24 h
Pac (nM)
0 20 40 80
0 20 40 80
Cyto- c
GAPDH
24 h
Pac (nM)
0 20 40 80
0 20 40 80
mitochondria
cytosol

Fig. 3f

Cleaved caspase 9

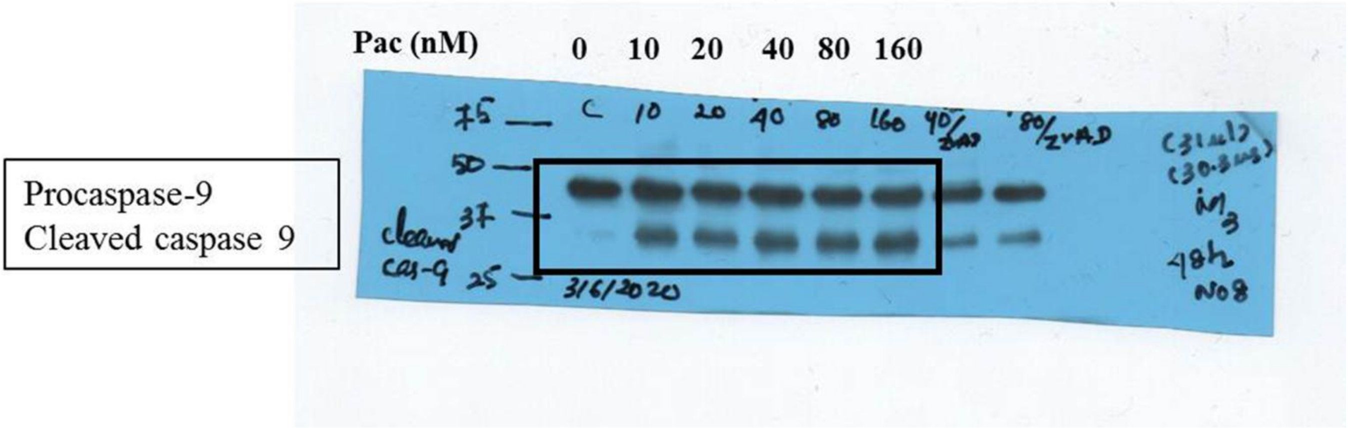

Fig. 3g

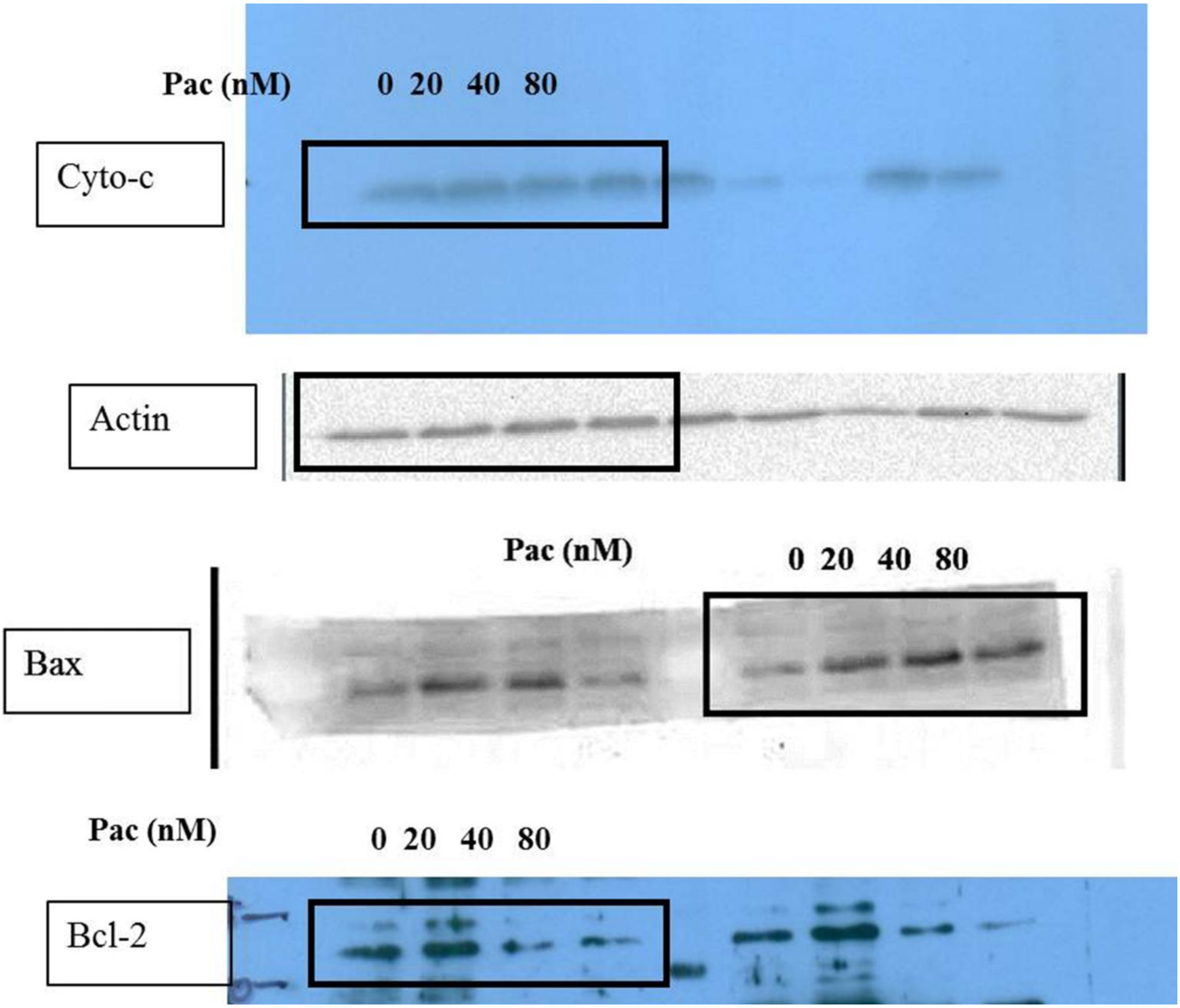

Fig. 3h

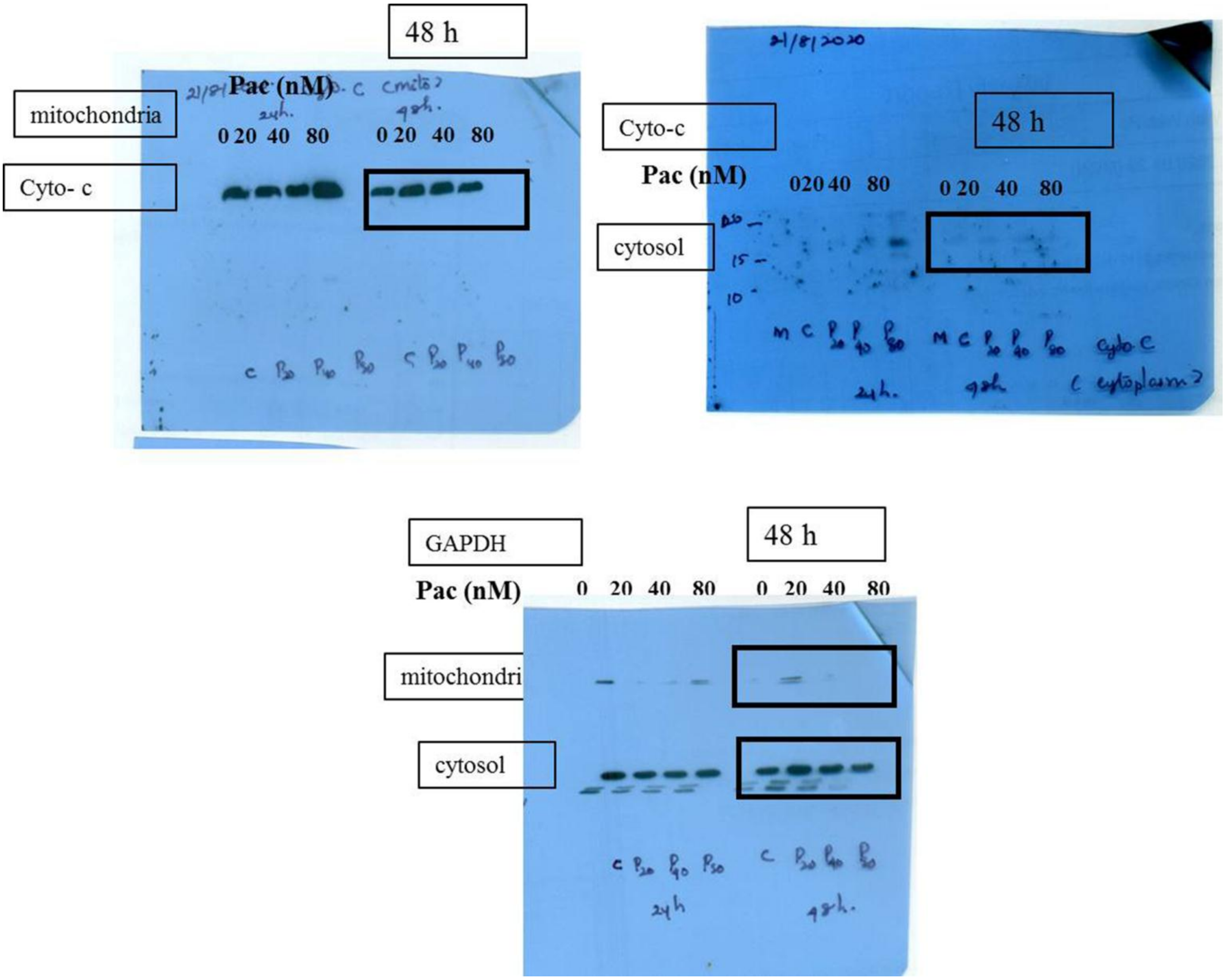

48 h
mitochondria
Pac (nM)
0 20 40 80 0 20 40 80
Cyto- c
Cyto-c
48 h
Pac (nM)
0 20 40 80 0 20 40 80
cytosol
GAPDH
48 h
Pac (nM)
0 20 40 80 0 20 40 80
mitochondri
cytosol

Supplementary Fig. C

Fig. C Photographs of the full-length blots described in Figure 4

Fig. 4a

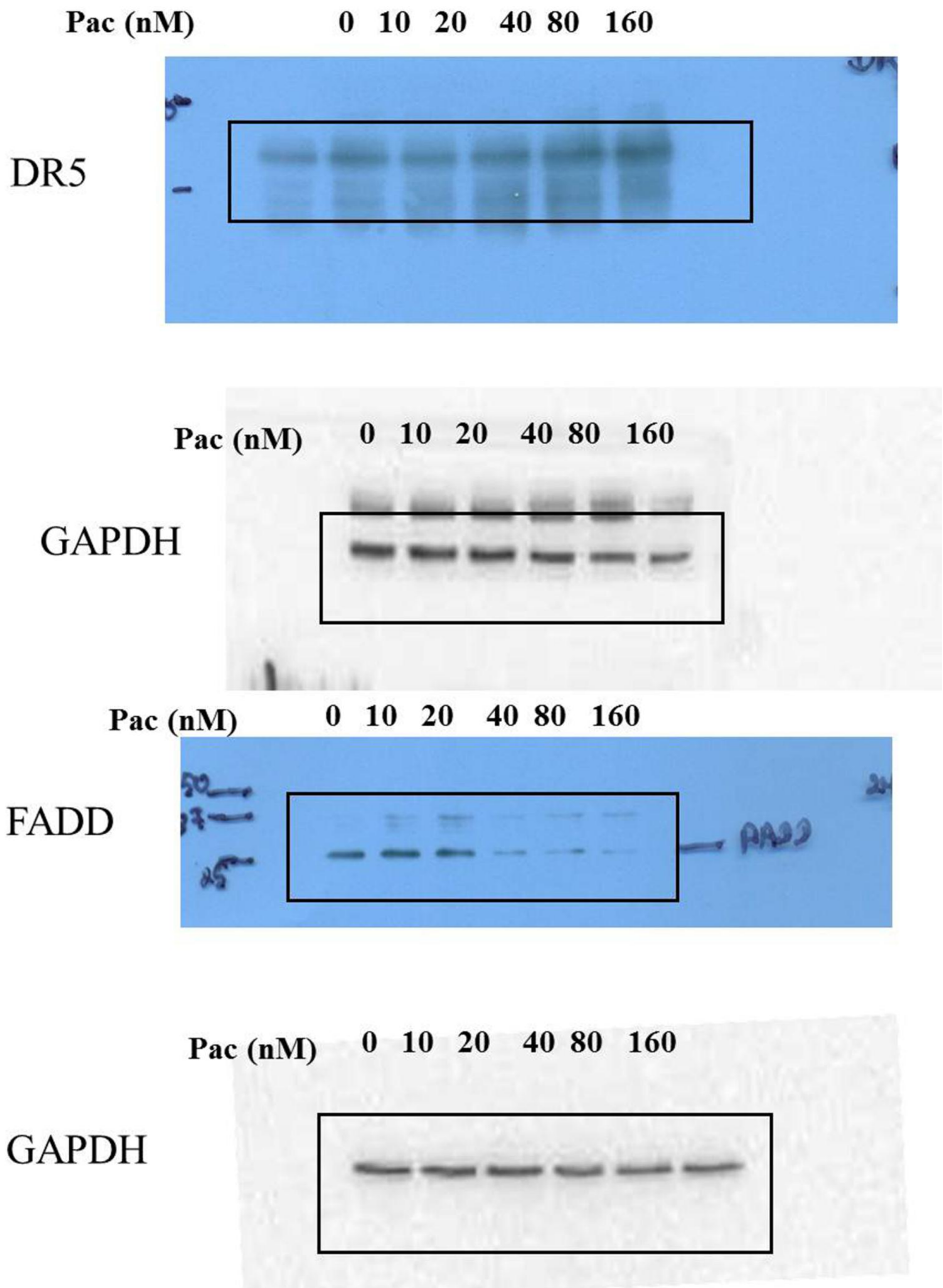

Fig.4b

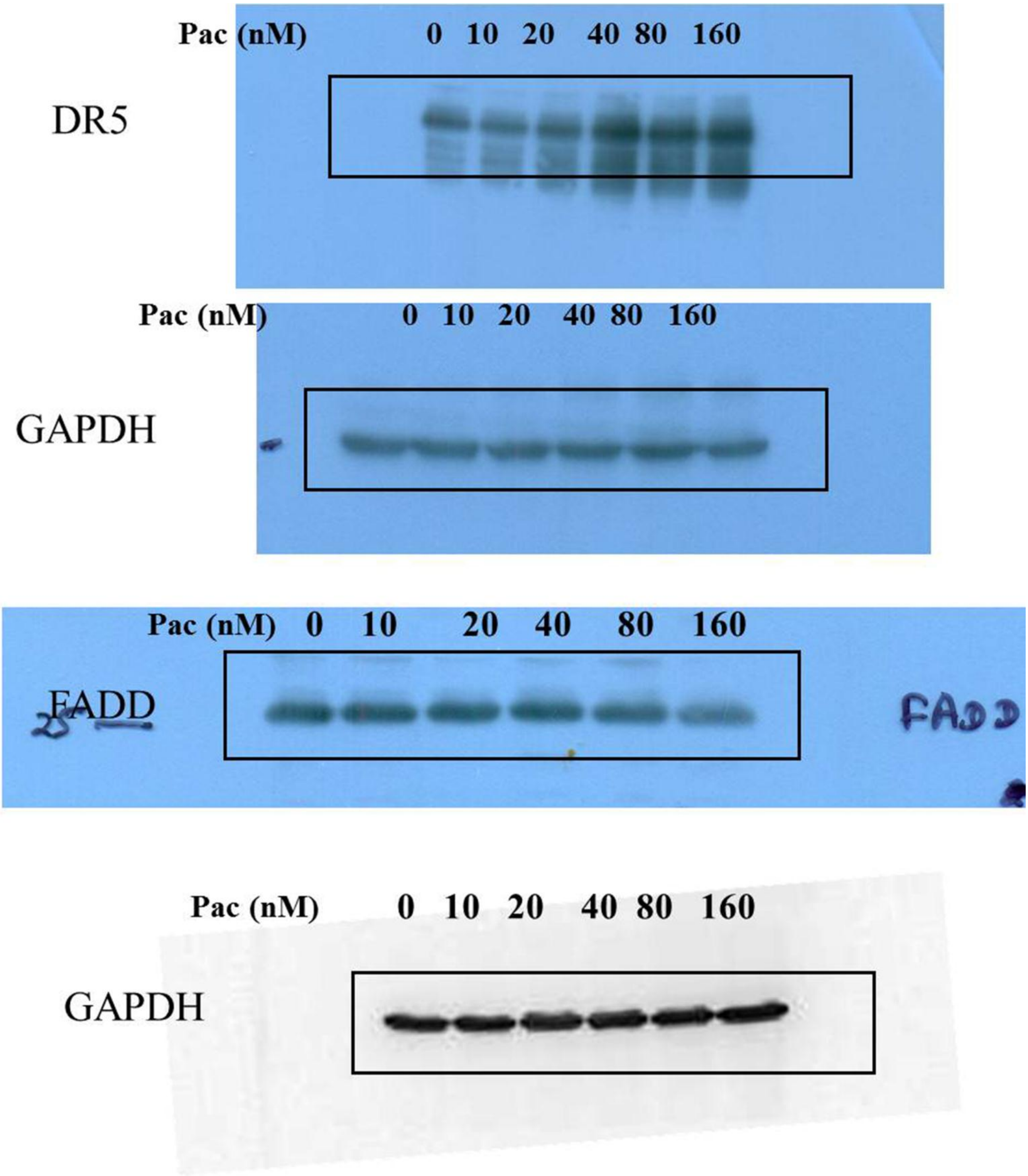
Pac (nM) 0 10 20 40 80 160
DR5
Pac (nM) 0 10 20 40 80 160
GAPDH
Pac (nM) 0 10 20 40 80 160
FADD
FADD
Pac (nM) 0 10 20 40 80 160
GAPDH

Fig. 4c

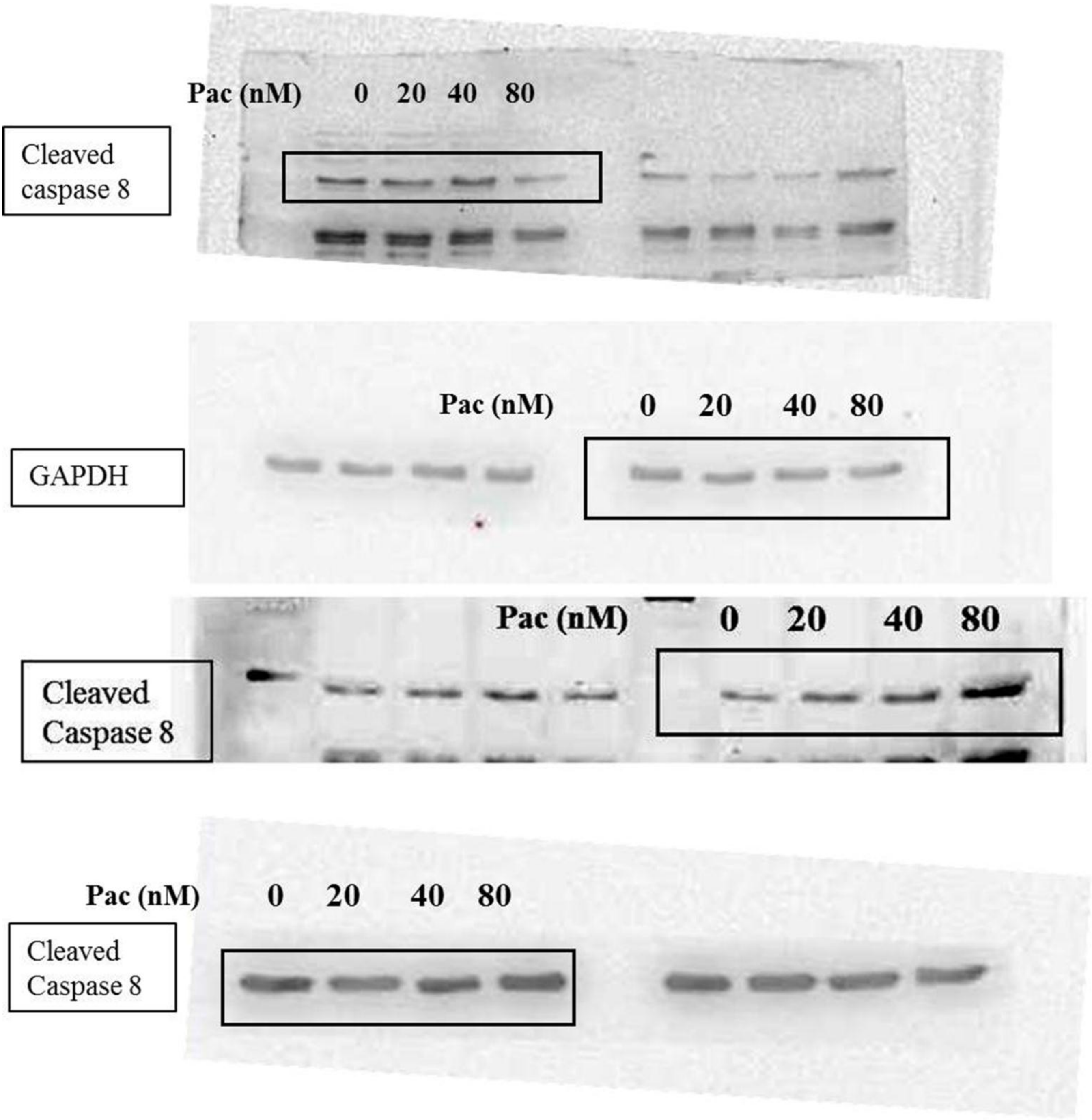

Pac (nM) 0 20 40 80
Cleaved caspase 8
Pac (nM) 0 20 40 80
GAPDH
Pac (nM) 0 20 40 80
Cleaved Caspase 8
Pac (nM) 0 20 40 80
Cleaved Caspase 8

Supplementary Fig. D

Fig.D Photographs of the full-length blots described in Figure 5

Fig. 5c

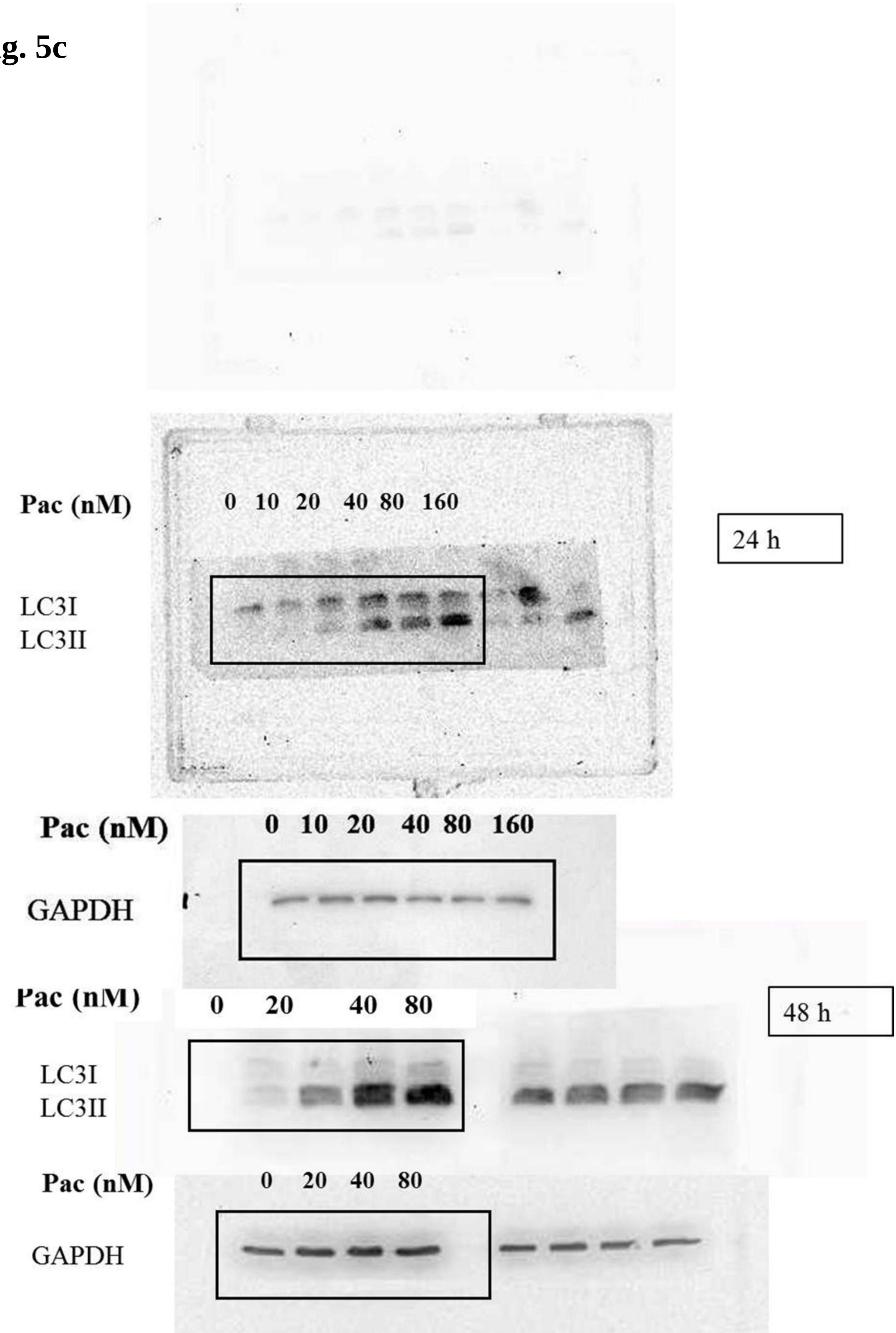

Fig. 5d

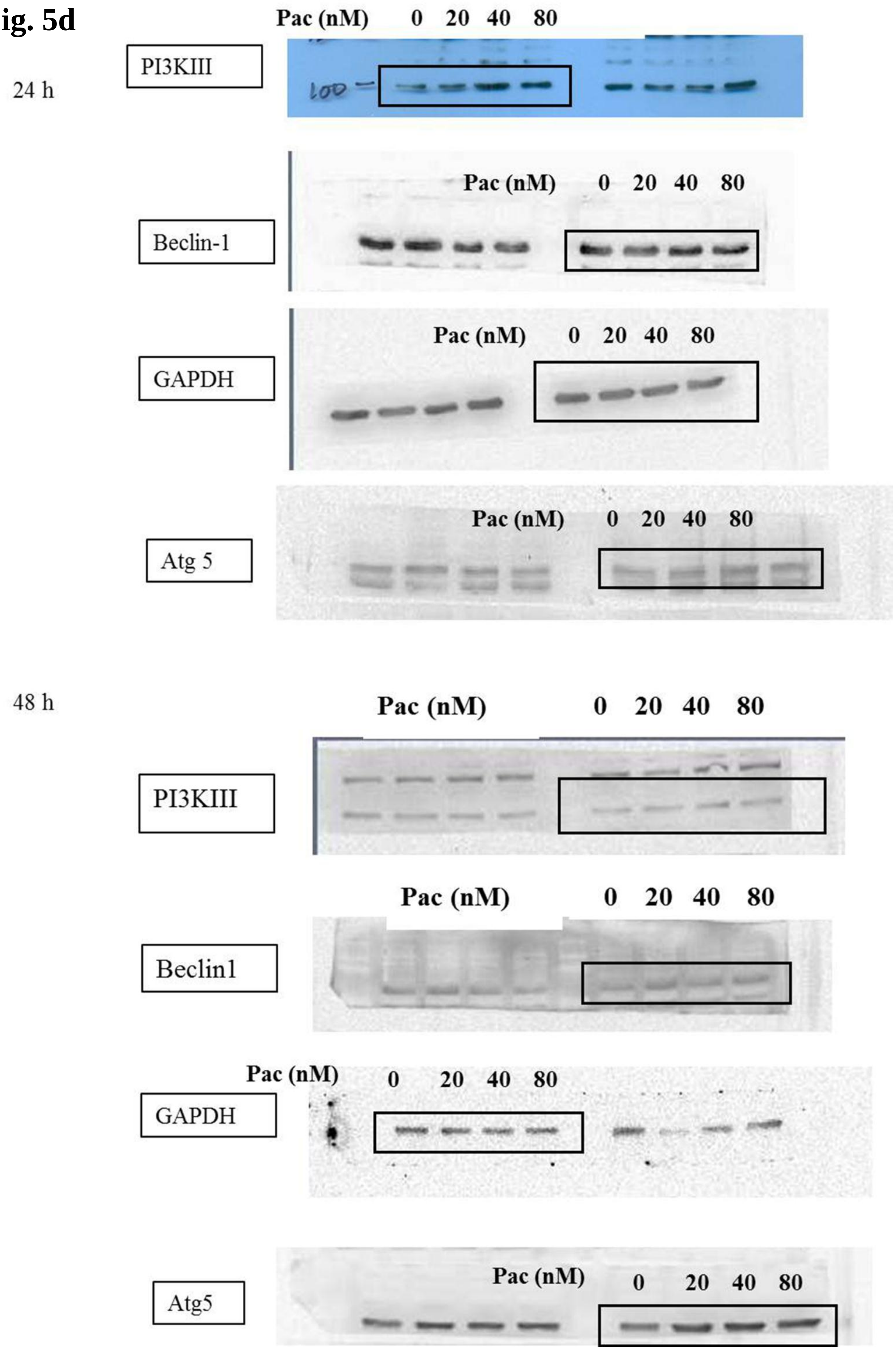

24 h
Pac (nM) 0 20 40 80
PI3KIII
100
Beclin-1
GAPDH
Atg 5
48 h
PI3KIII
Beclin1
GAPDH
Atg5

Fig. 5e

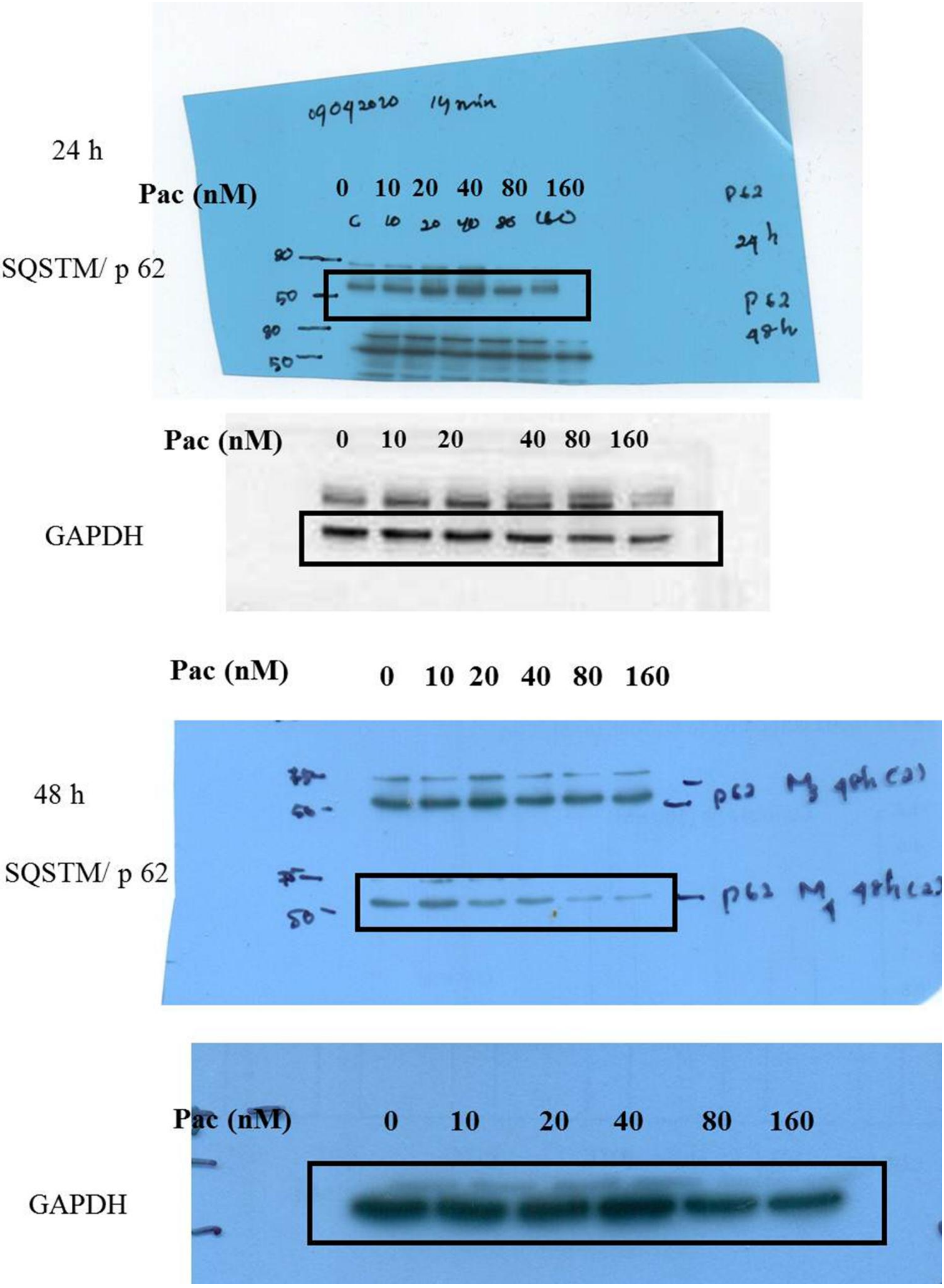
24 h
Pac (nM) 0 10 20 40 80 160
SQSTM/ p 62
Pac (nM) 0 10 20 40 80 160
GAPDH
Pac (nM) 0 10 20 40 80 160
48 h
SQSTM/ p 62
Pac (nM) 0 10 20 40 80 160
GAPDH

Fig. 5f

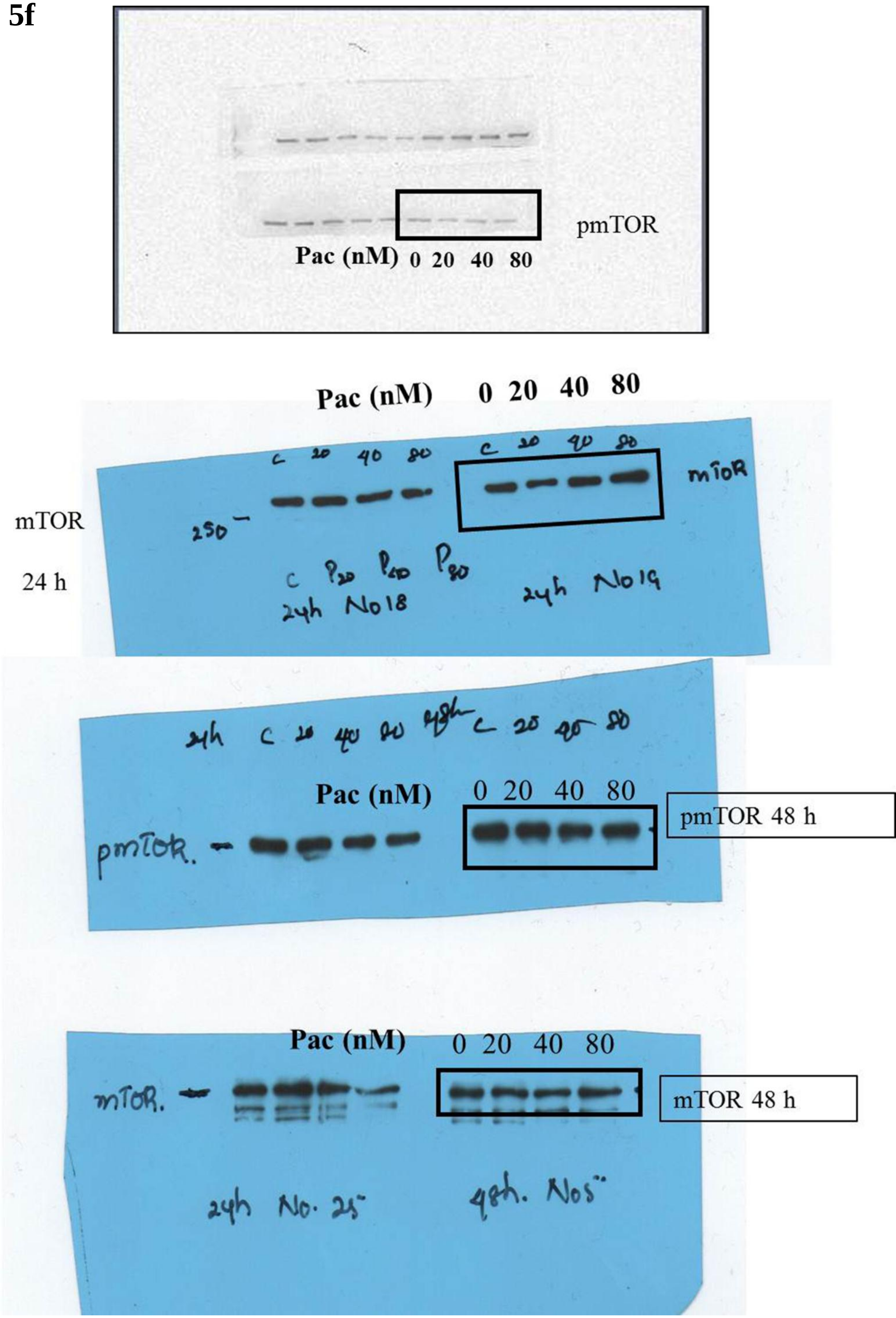
pmTOR
Pac (nM) 0 20 40 80
Pac (nM) 0 20 40 80
mTOR
24 h
Pac (nM) 0 20 40 80
pmTOR 48 h
Pac (nM) 0 20 40 80
mTOR 48 h

Fig. 5g

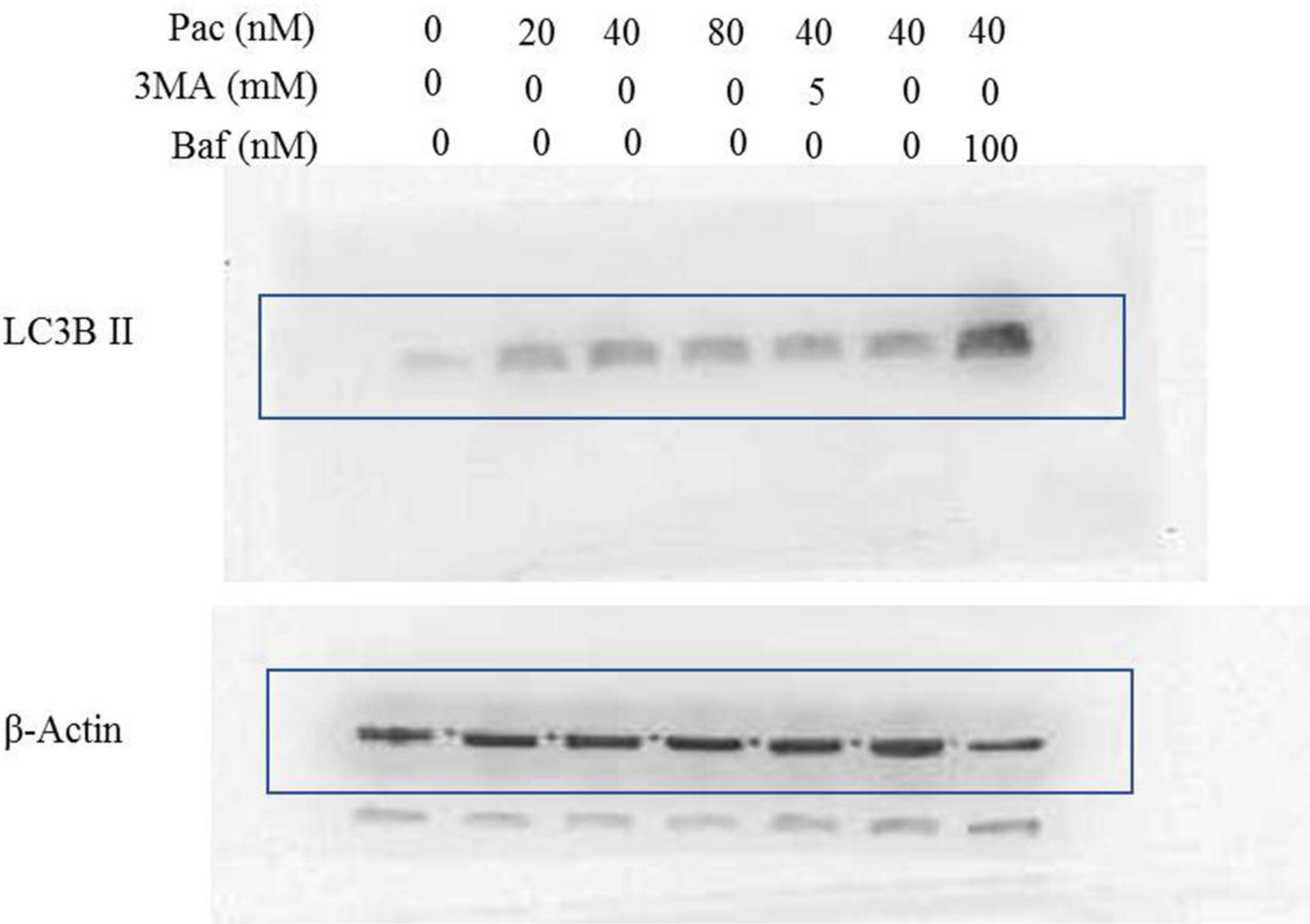
Pac (nM) 0 20 40 80 40 40 40
3MA (mM) 0 0 0 0 5 0 0
Baf (nM) 0 0 0 0 0 0 100
LC3B II
β-Actin

Supplementary Fig. E

Fig. E Photographs of the full-length blots described in Figure 6

Fig. 6b

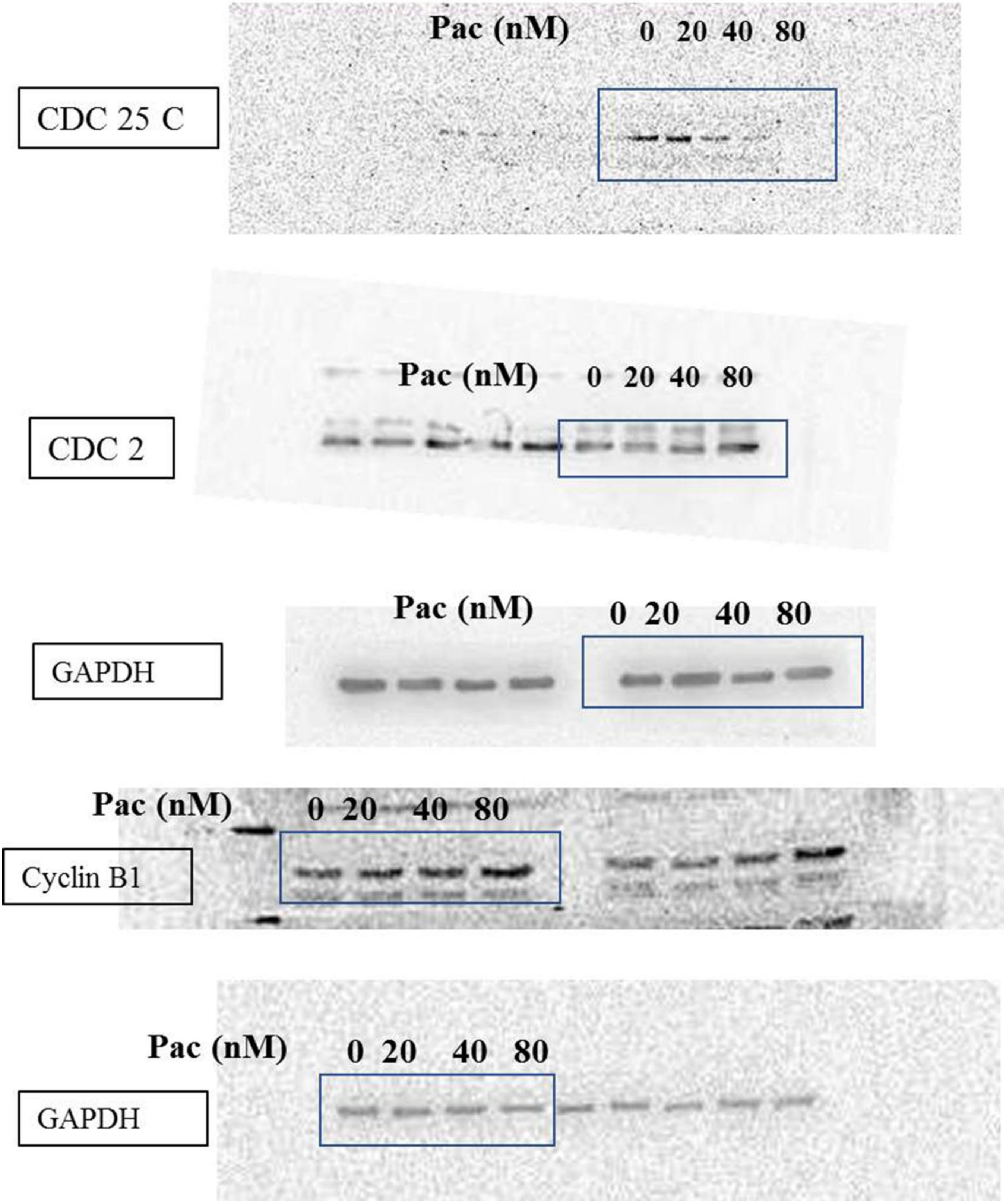

Fig. 6d

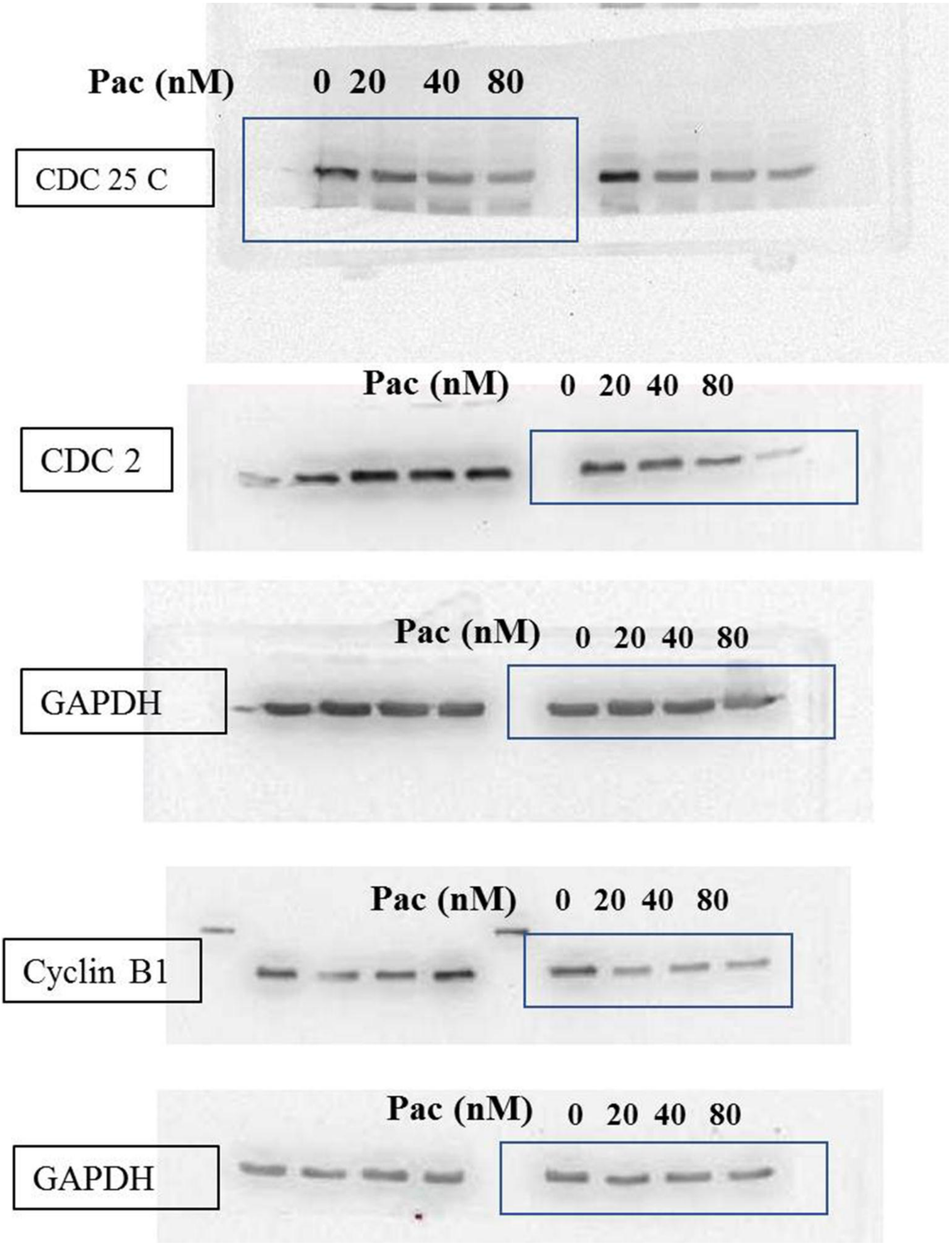

Pac (nM) 0 20 40 80
CDC 25 C
Pac (nM) 0 20 40 80
CDC 2
Pac (nM) 0 20 40 80
GAPDH
Pac (nM) 0 20 40 80
Cyclin B1
Pac (nM) 0 20 40 80
GAPDH

Supplementary Fig. F

Fig. F Photographs of the full-length blots described in Figure 7

Fig. 7f

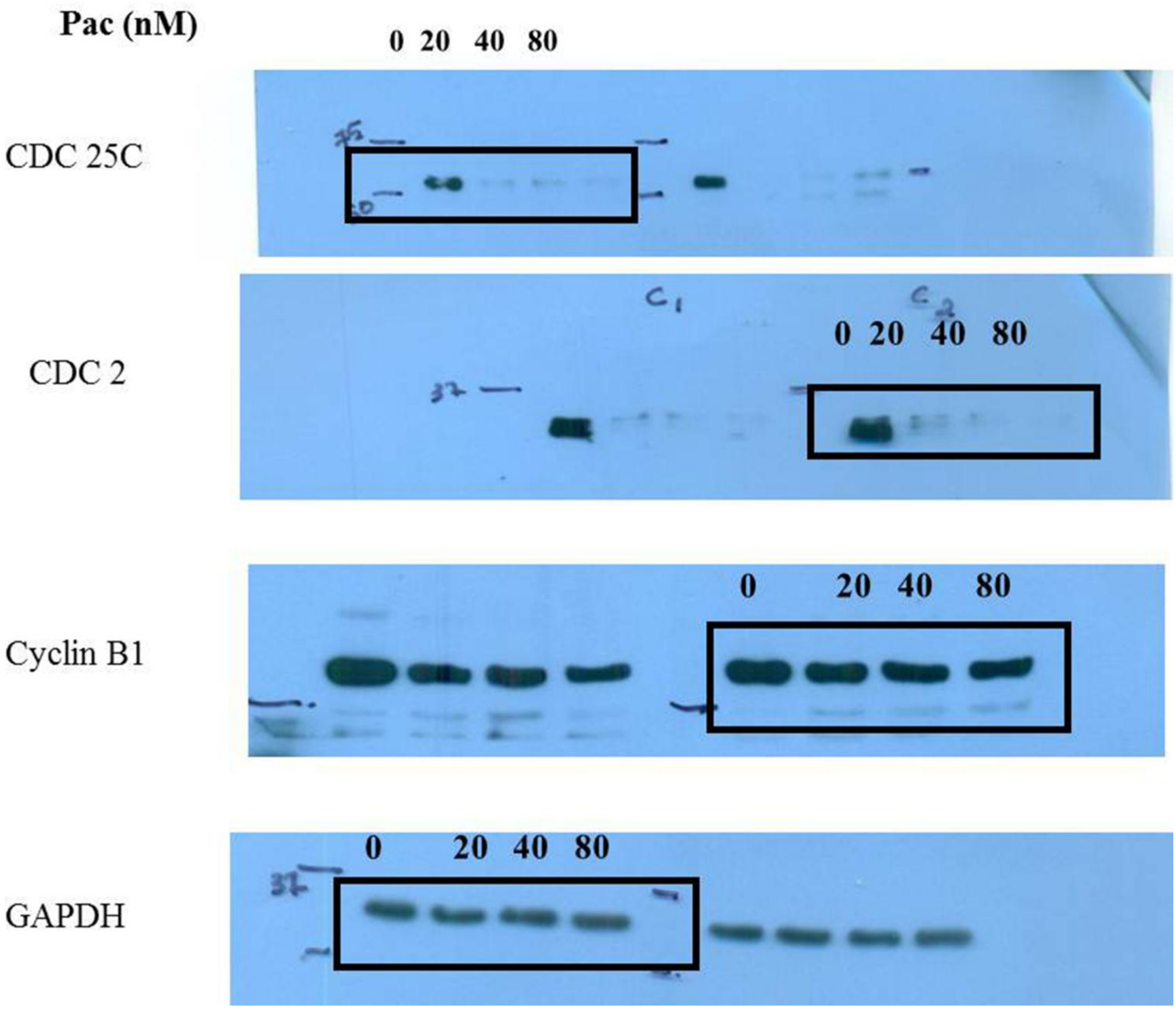

Fig. 7g

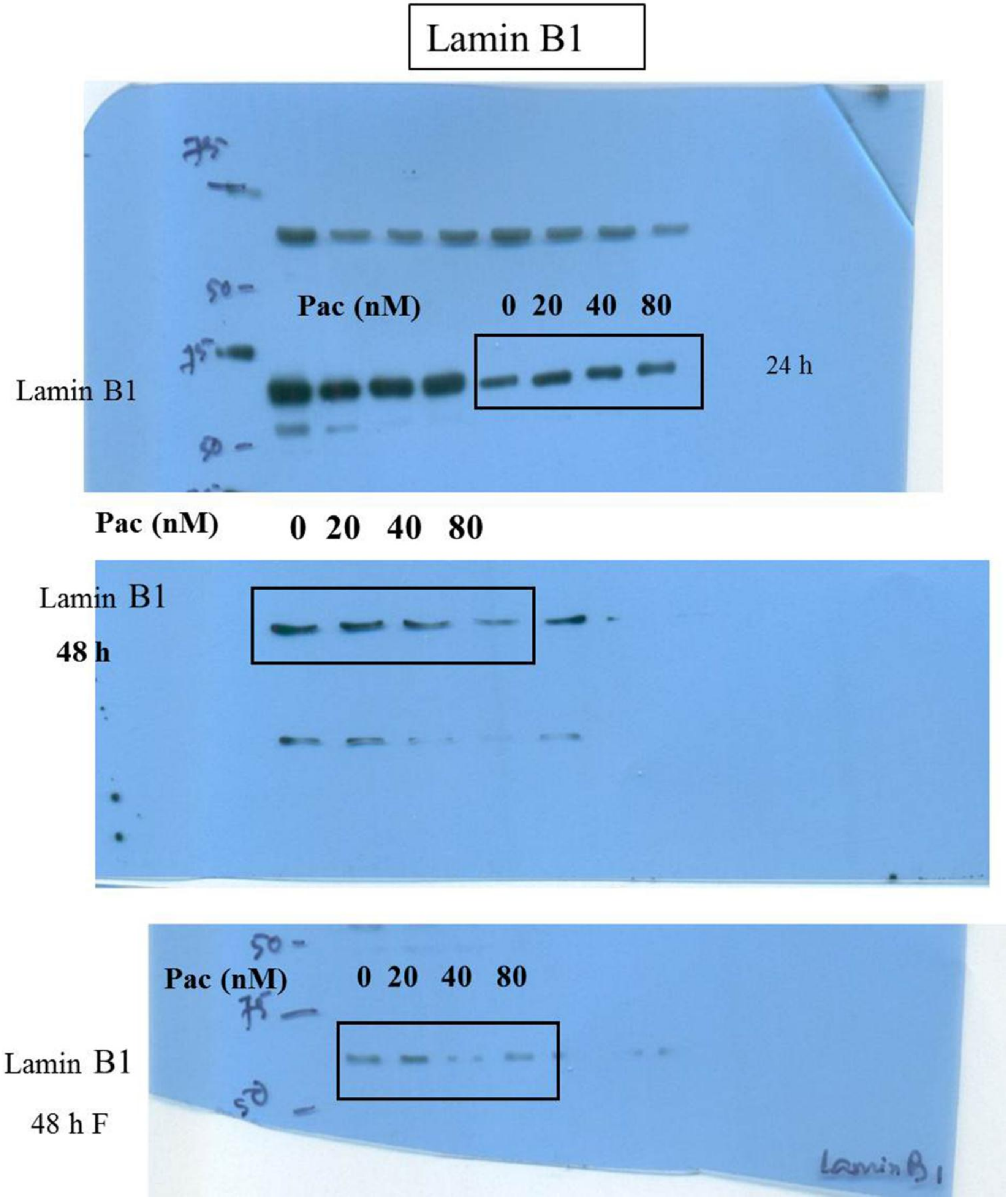
Lamin B1
Pac (nM)
0 20 40 80
24 h
Lamin B1
Pac (nM)
0 20 40 80
Lamin B1
48 h
Pac (nM)
0 20 40 80
Lamin B1
48 h F

Fig. 7h

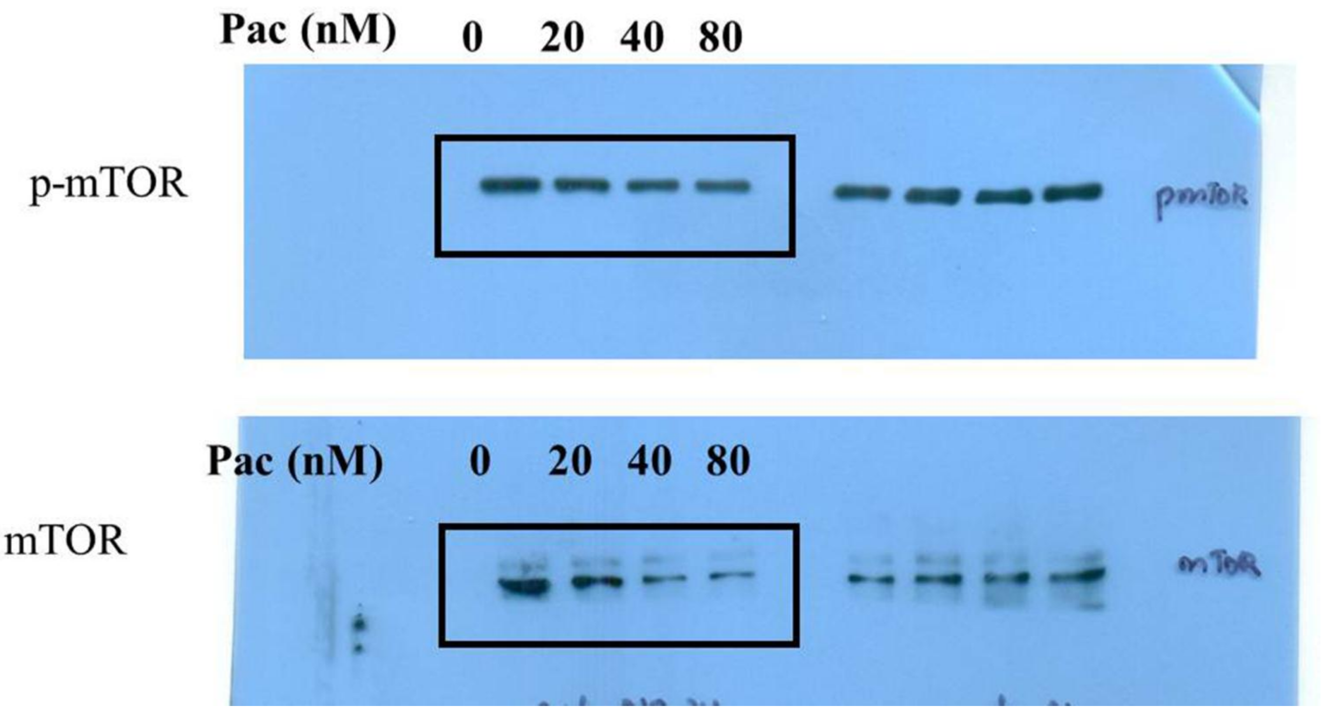

Supplement: Supplementary file 1 — Supplementary Information 1. [file 41598_2021_2503_MOESM1_ESM.pdf]
