# Supplementary material for: The effect of paclitaxel on apoptosis, autophagy and mitotic catastrophe in AGS cells
Source: Sci Rep. 2021 Dec 6;11:23490. doi: 10.1038/s41598-021-02503-9 (PMC8648765; doi:10.1038/s41598-021-02503-9)
Supplement: Supplementary file 2 — Supplementary Information 2. [file 41598_2021_2503_MOESM2_ESM.pdf]

The effect of paclitaxel on apoptosis, autophagy and mitotic catastrophe in AGS cells

Tin Myo KHING, Won Seok CHOI, Dong Min KIM, Wah Wah PO, Wynn THEIN, Chang Yell SHIN and Uy Dong SOHN*

Laboratory of Signaling and Pharmacological Activity, Department of Pharmacology, College of Pharmacy, Chung-Ang University, Seoul, Republic of Korea

***Corresponding author:** Uy Dong Sohn, Professor, Ph.D., Department of Pharmacology, College of Pharmacy, Chung-Ang University, Seoul, 06974, Republic of Korea. Tel.: +82 28205614, Fax: 011 +8228268752, e-mail: udsohn@cau.ac.kr

Supplementary Fig. A

Fig. A Photographs of the full-length blots described in Figure 2

Fig. 2b

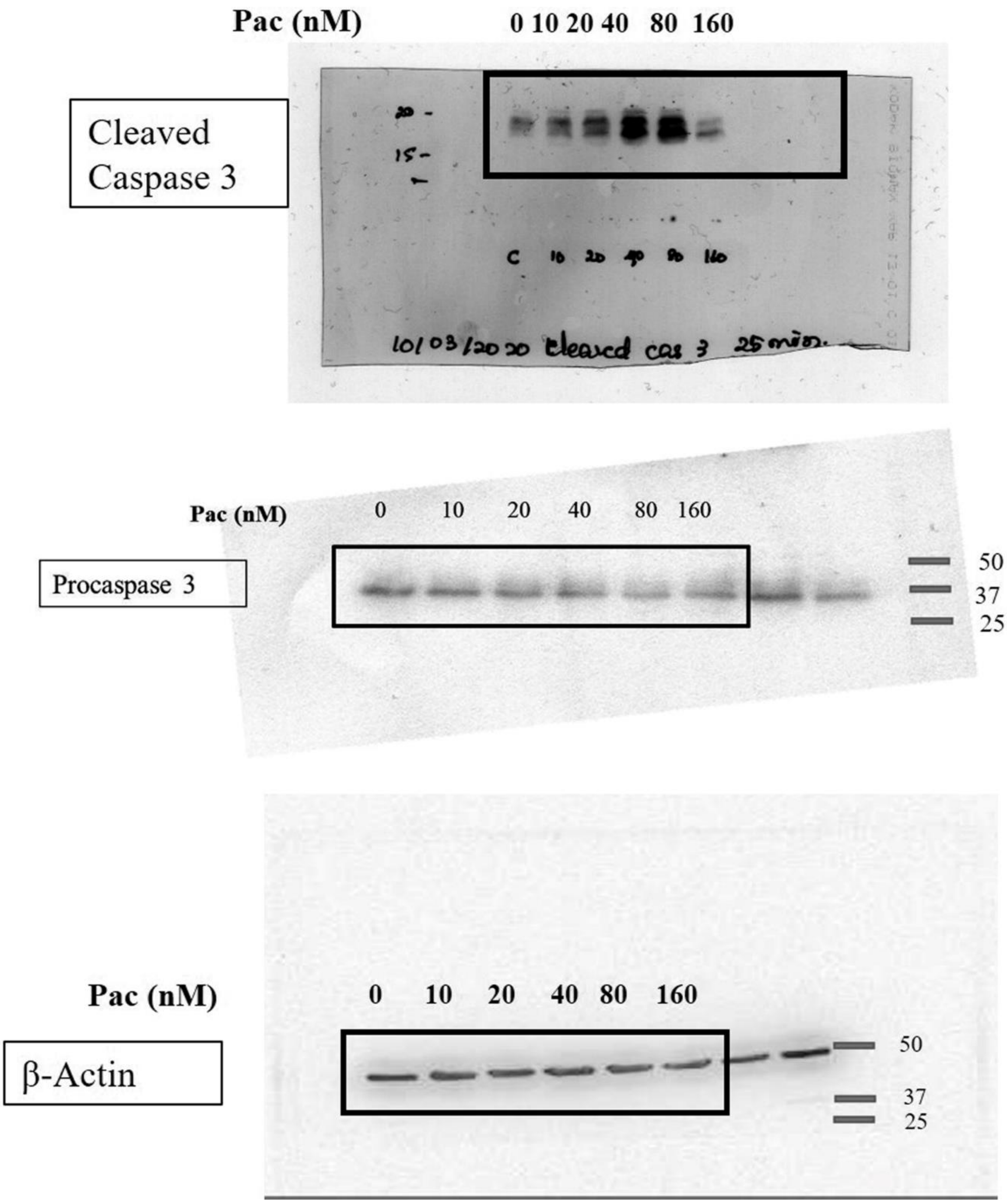

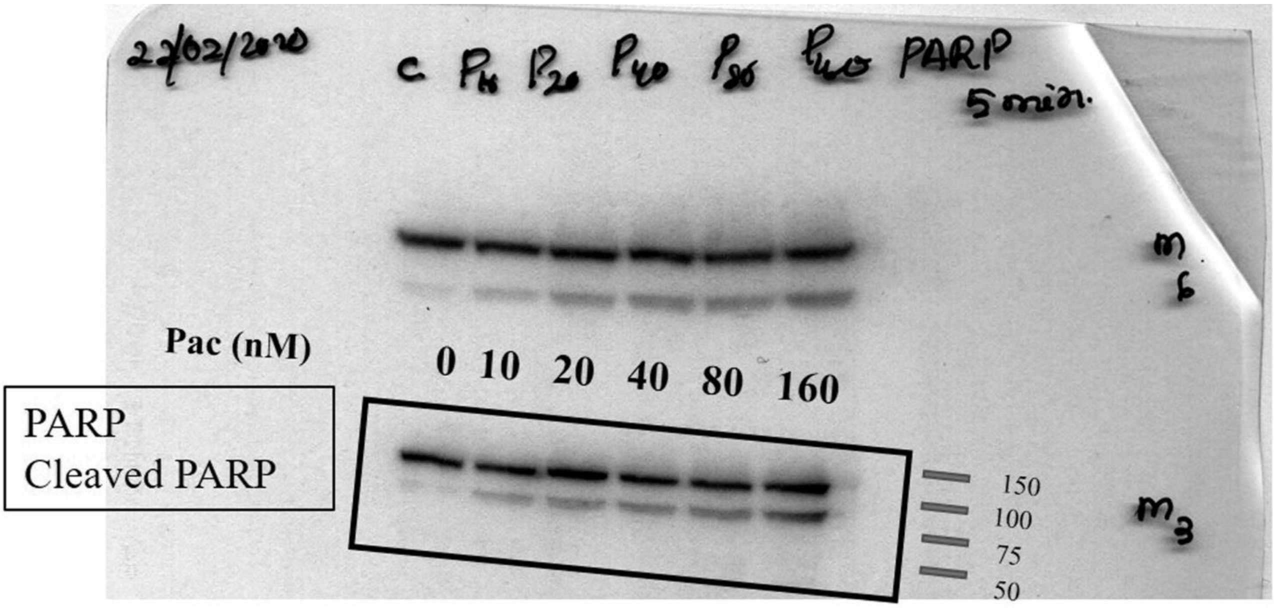
Pac (nM)
0 10 20 40 80 160
PARP
Cleaved PARP
150
100
75
50

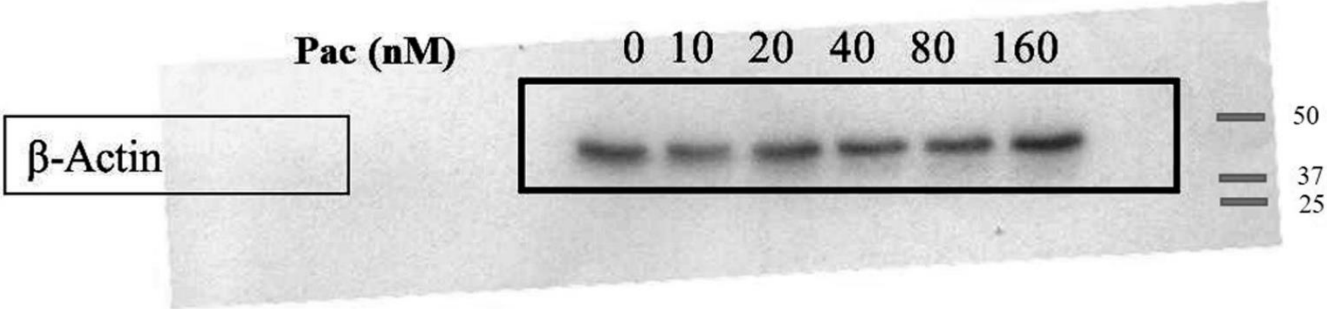
Pac (nM)
0 10 20 40 80 160
β-Actin
50
37
25

Fig.2d

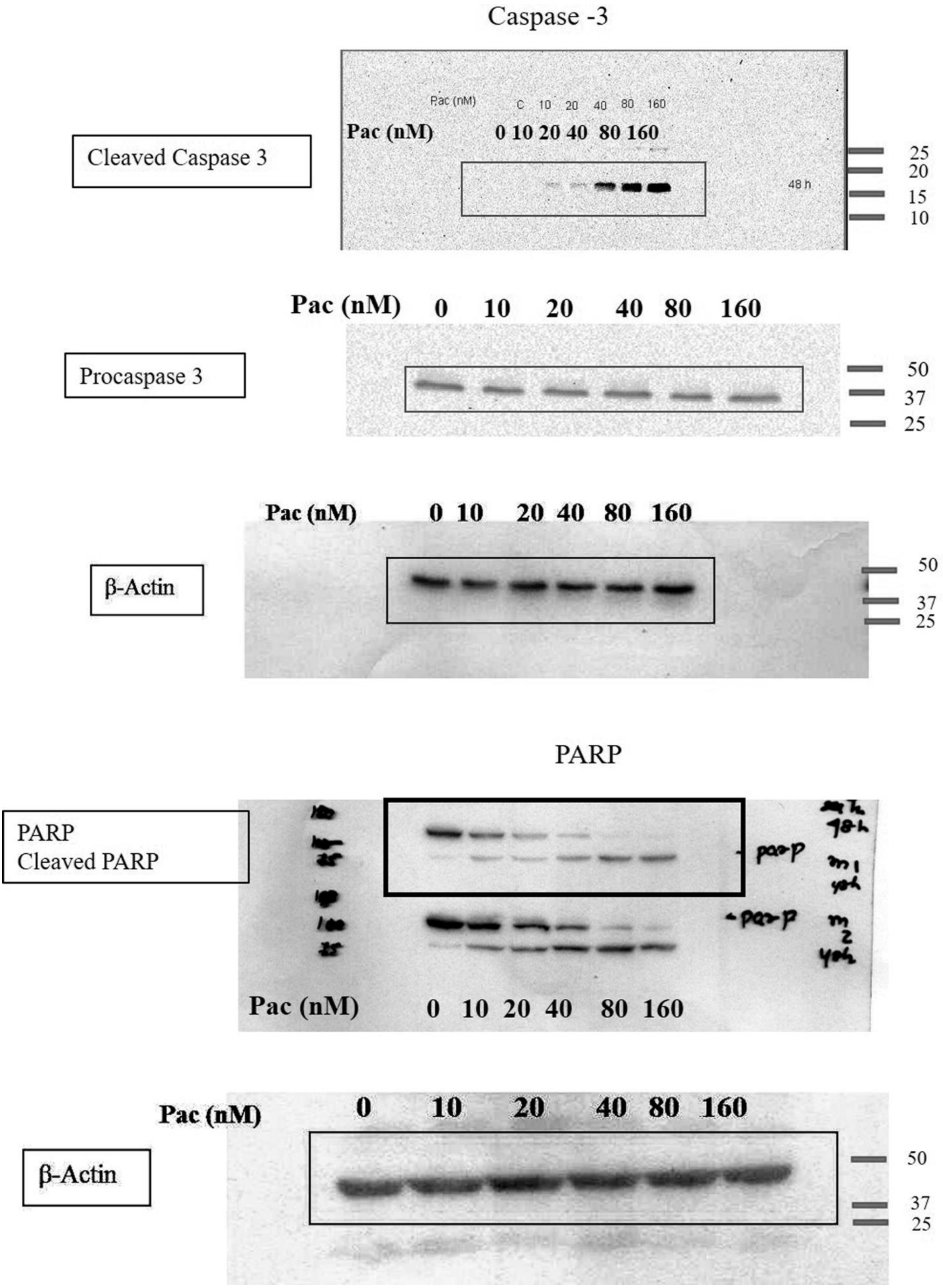
Caspase -3
Cleaved Caspase 3
Pac (nM) C 10 20 40 80 160
Pac (nM) 0 10 20 40 80 160
48 h
25
20
15
10
Pac (nM) 0 10 20 40 80 160
Procaspase 3
50
37
25
Pac (nM) 0 10 20 40 80 160
β-Actin
50
37
25
PARP
PARP
Cleaved PARP
Pac (nM) 0 10 20 40 80 160
Pac (nM) 0 10 20 40 80 160
β-Actin
50
37
25

Fig. 2e

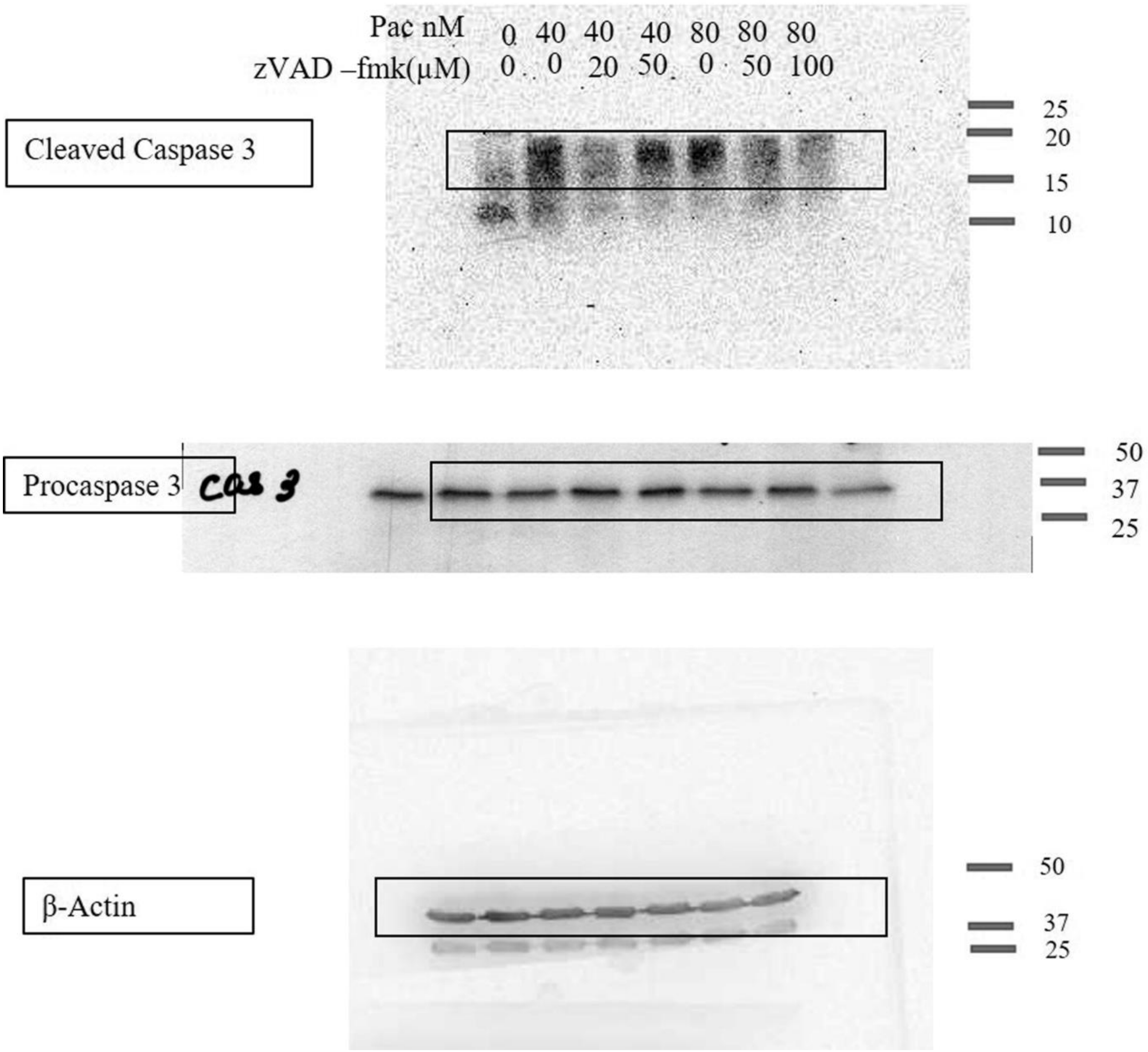
Pac nM 0 40 40 40 80 80 80
zVAD –fmk(μM) 0 0 20 50 0 50 100
Cleaved Caspase 3
25
20
15
10
Procaspase 3
cas 3
50
37
25
β-Actin
50
37
25

Supplementary Fig. B

Fig. B Photographs of the full-length blots described in Figure 3

Fig. 3b

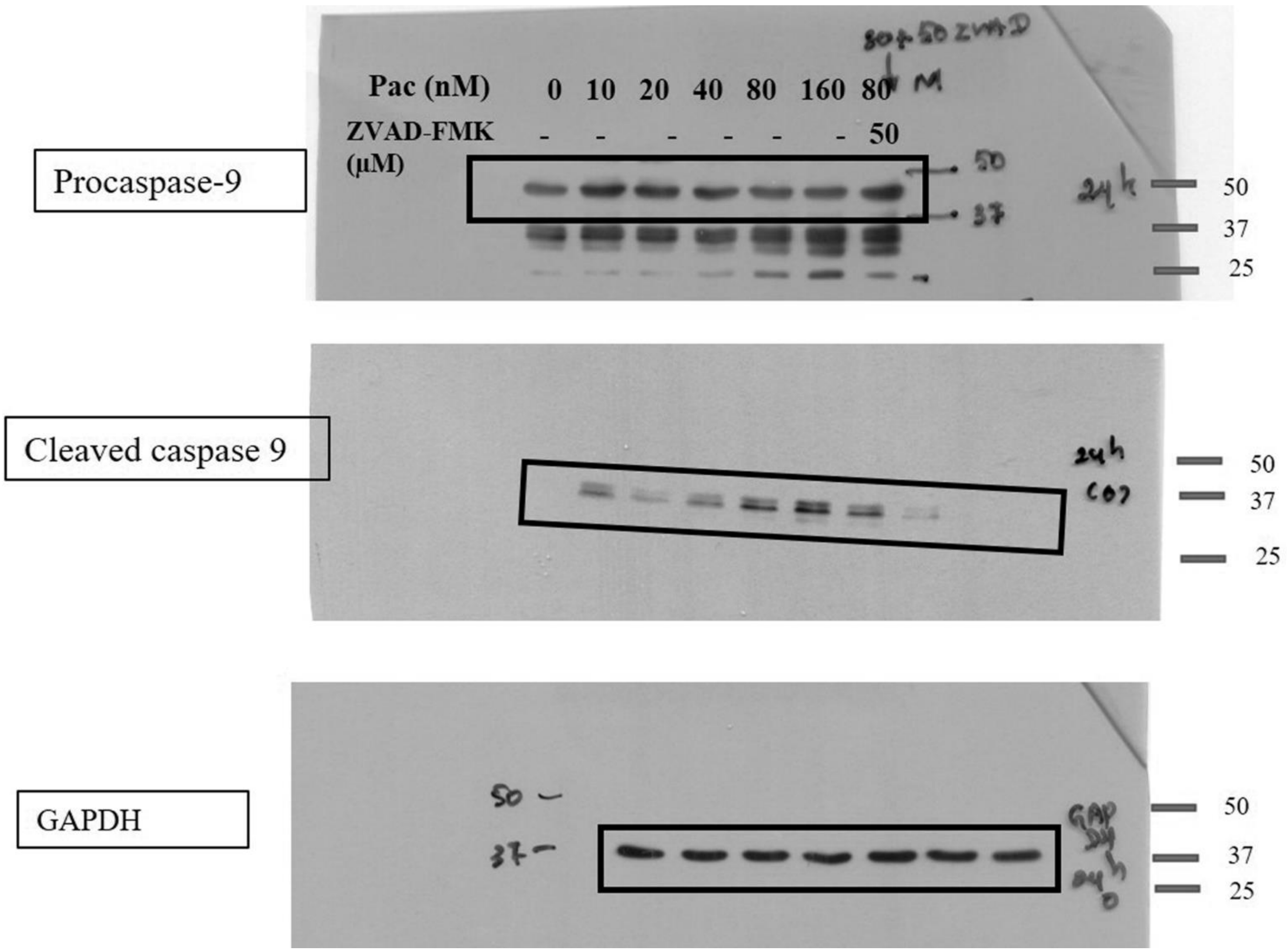

Fig. 3c

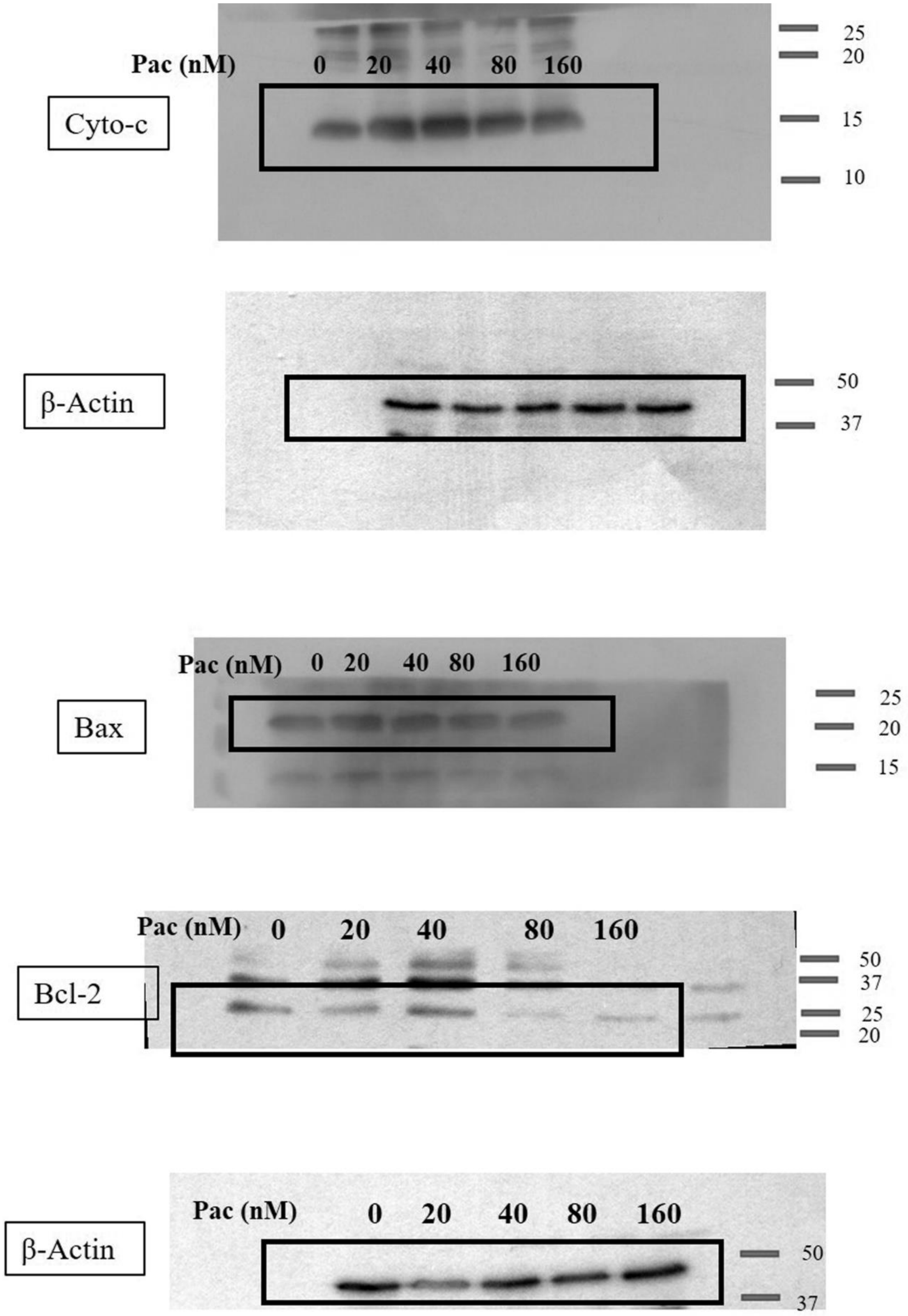
Pac (nM)
0
20
40
80
160
Cyto-c
25
20
15
10
β-Actin
50
37
Pac (nM)
0
20
40
80
160
Bax
25
20
15
Pac (nM)
0
20
40
80
160
Bcl-2
50
37
25
20
Pac (nM)
0
20
40
80
160
β-Actin
50
37

Fig.3d

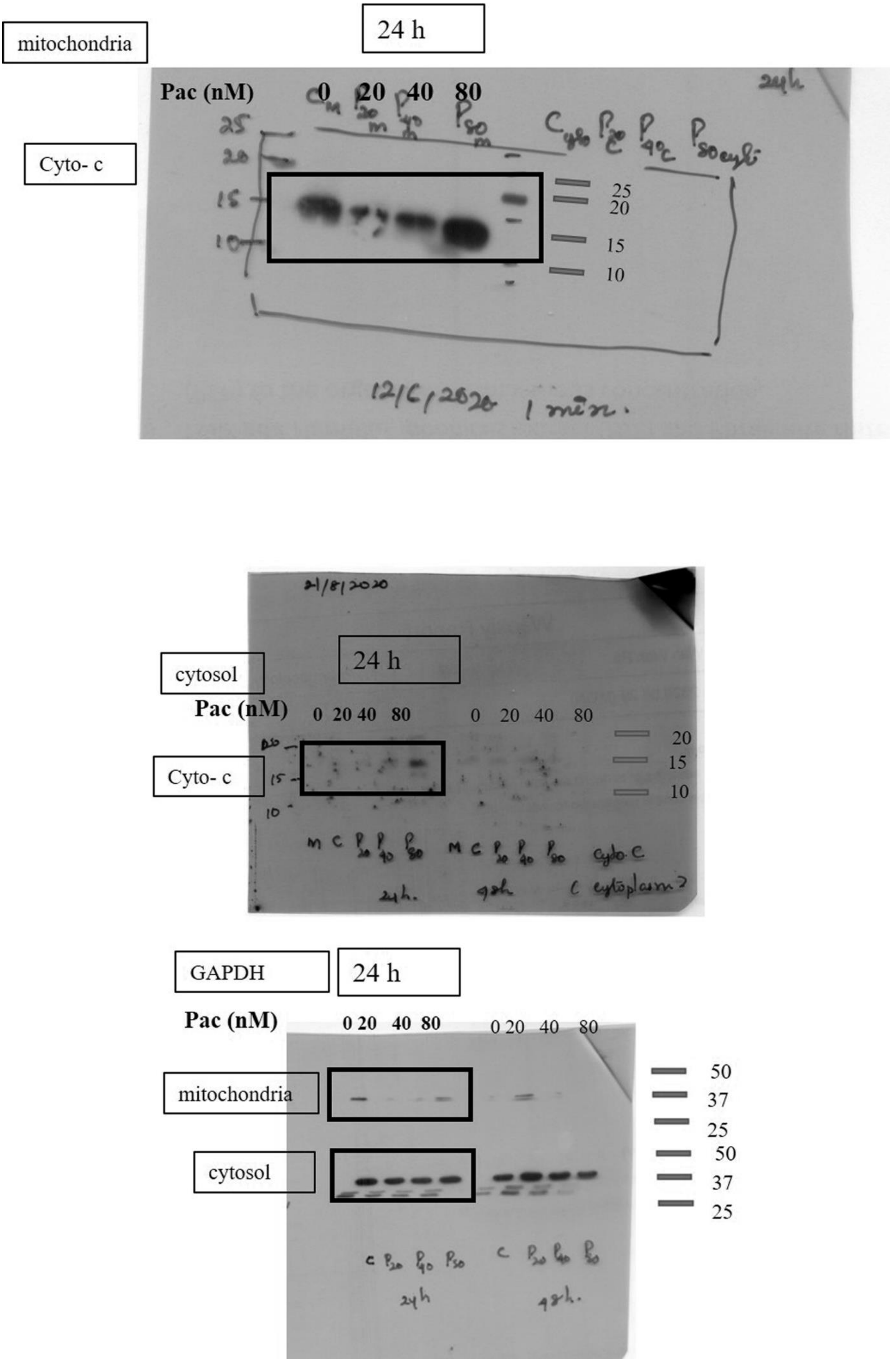
mitochondria
24 h
Pac (nM) 0 20 40 80
Cyto- c
25
20
15
10
cytosol
24 h
Pac (nM) 0 20 40 80 0 20 40 80
Cyto- c
20
15
10
GAPDH
24 h
Pac (nM) 0 20 40 80 0 20 40 80
mitochondria
cytosol
50
37
25
50
37
25

Fig. 3f

Cleaved caspase 9

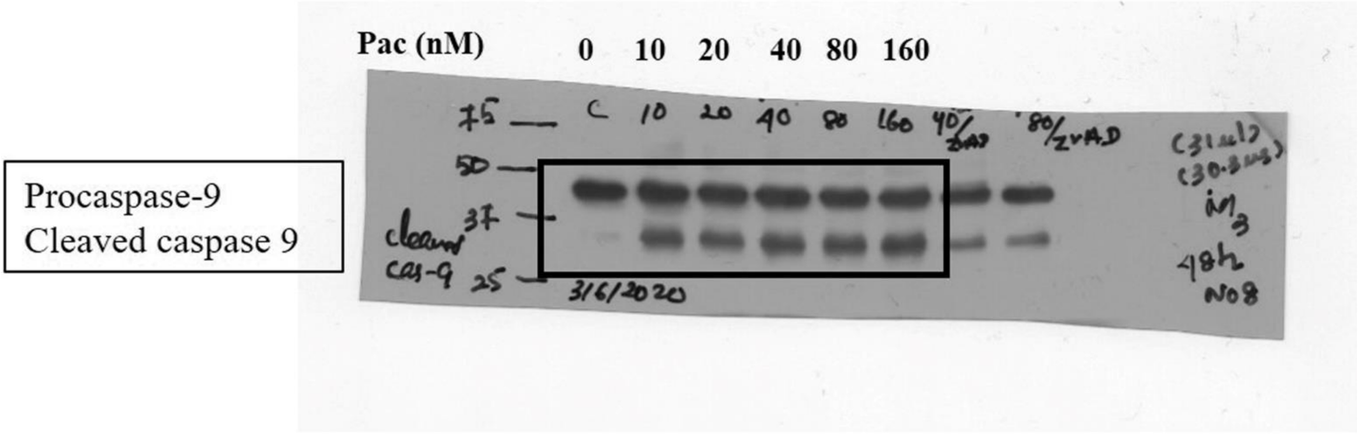

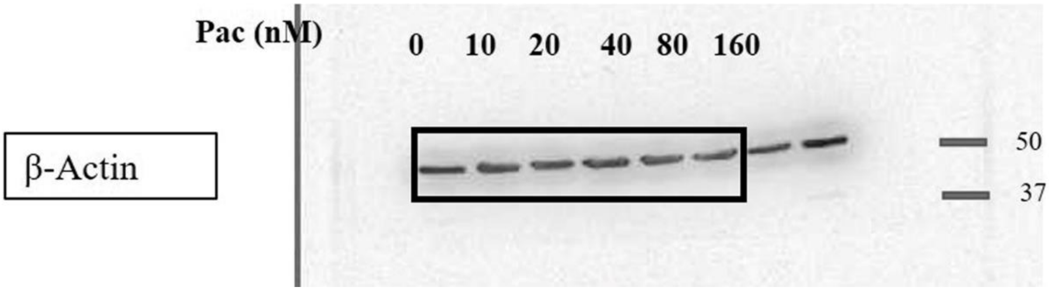

Fig. 3g

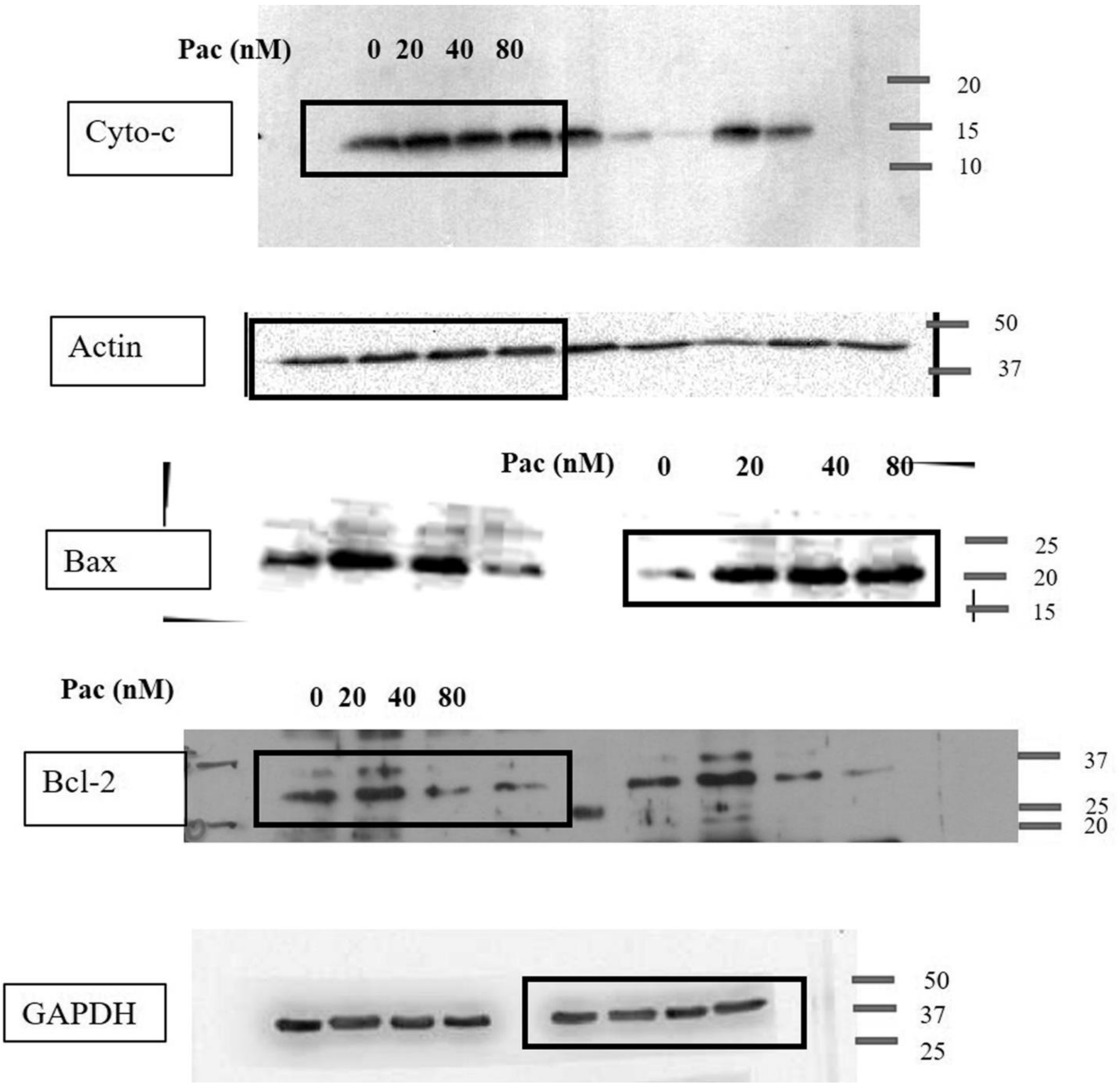
Pac (nM)
0 20 40 80
Cyto-c
20
15
10
Actin
50
37
Pac (nM)
0 20 40 80
Bax
25
20
15
Pac (nM)
0 20 40 80
Bcl-2
37
25
20
GAPDH
50
37
25

Fig. 3h

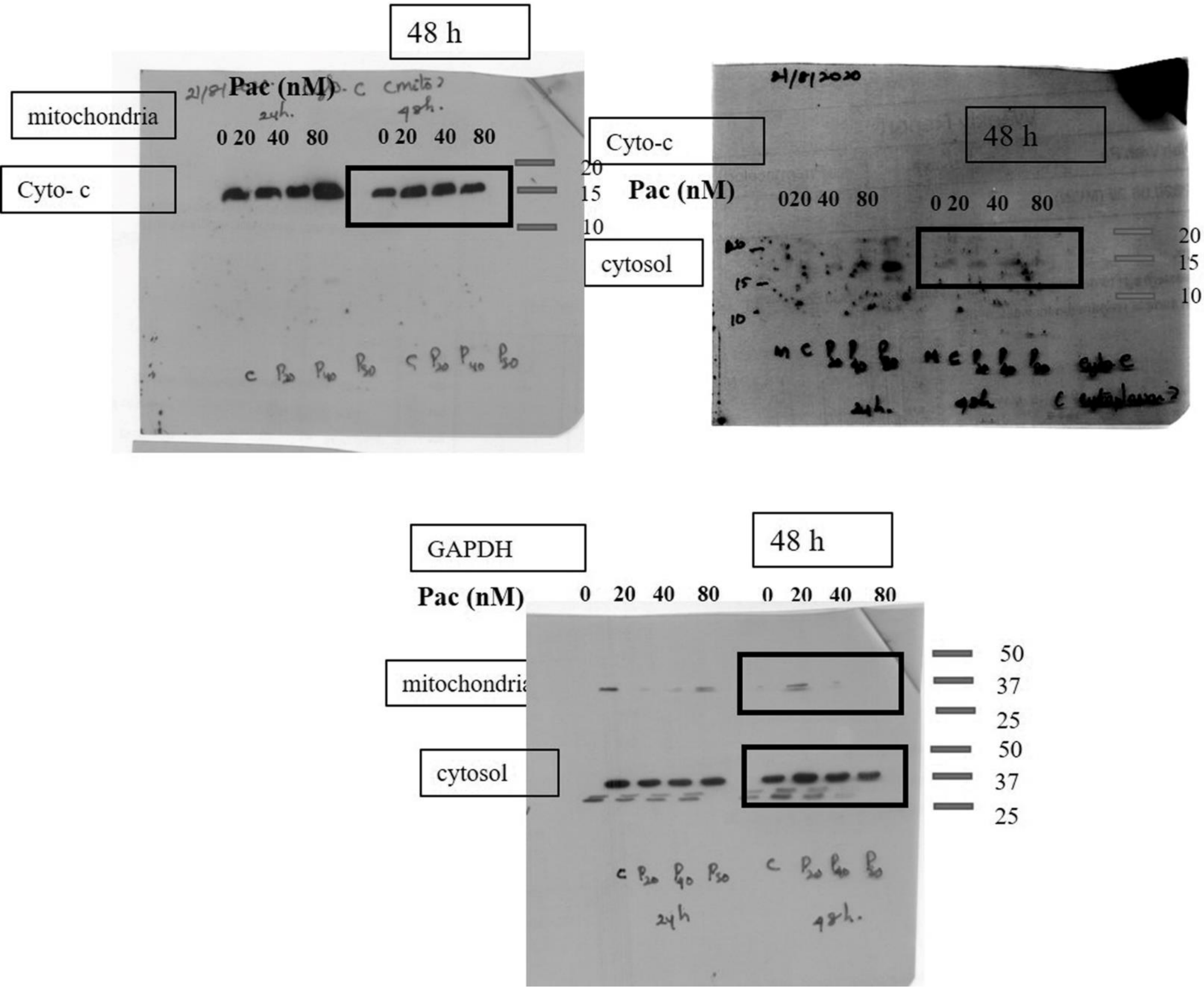
48 h
mitochondria
Pac (nM)
0 20 40 80 0 20 40 80
Cyto- c
20
15
10
Cyto-c
48 h
Pac (nM)
020 40 80 0 20 40 80
cytosol
20
15
10
GAPDH
48 h
Pac (nM) 0 20 40 80 0 20 40 80
mitochondria
50
37
25
cytosol
50
37
25

Supplementary Fig. D

Fig. D Photographs of the full-length blots utilized in Figure 4

Fig. 4a

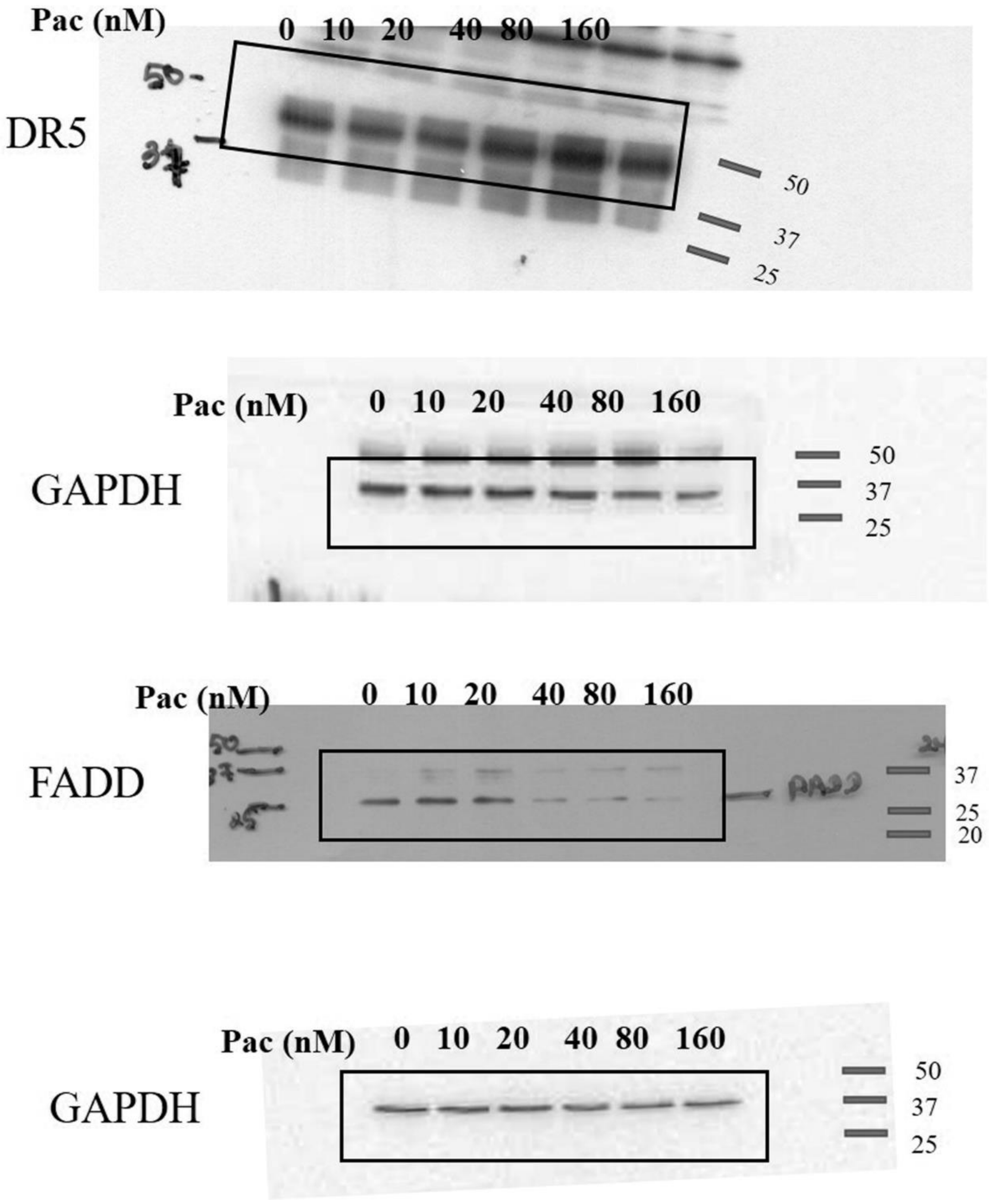

Fig.4b

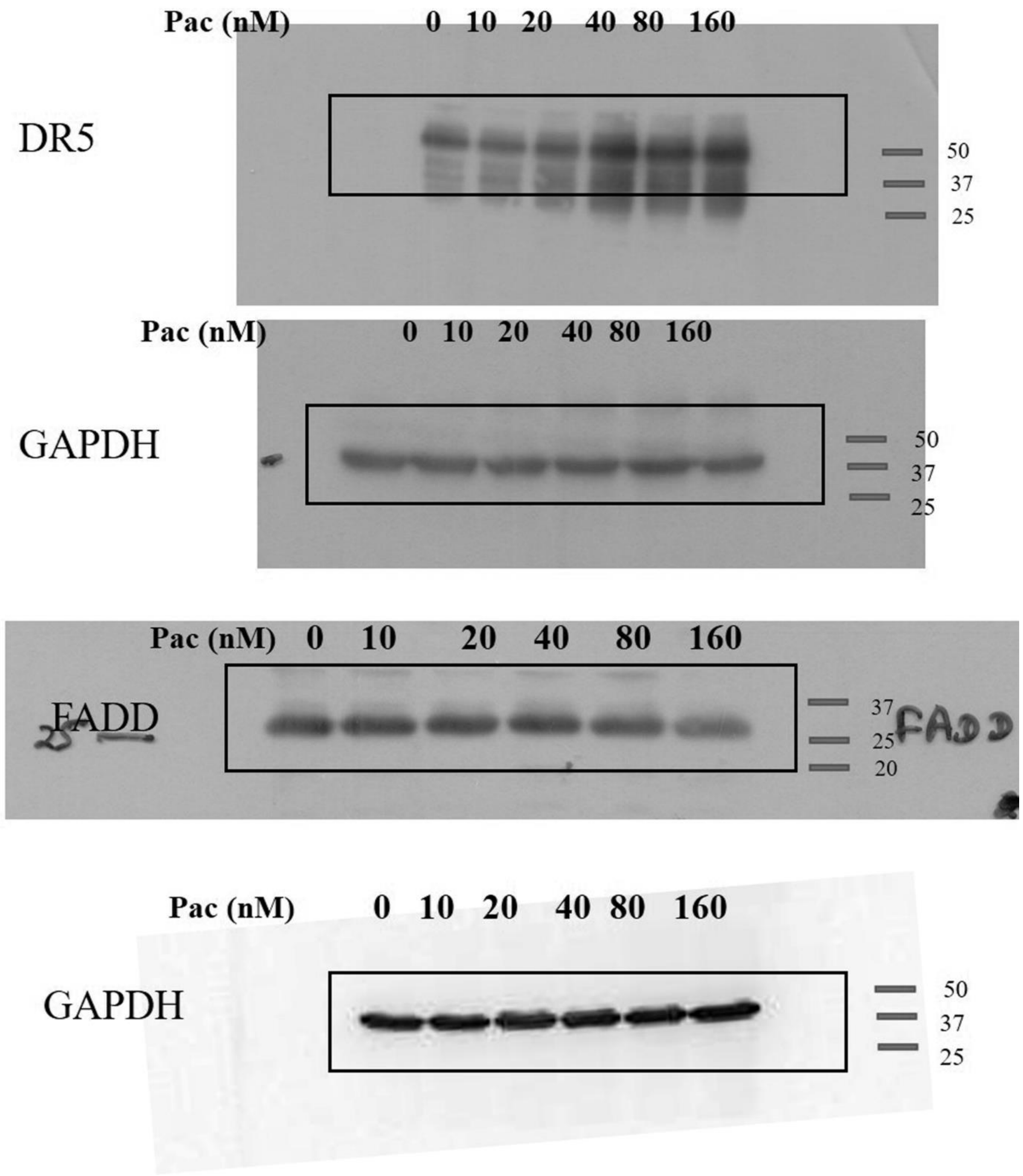
Pac (nM)
0 10 20 40 80 160
DR5
50
37
25
Pac (nM)
0 10 20 40 80 160
GAPDH
50
37
25
Pac (nM)
0 10 20 40 80 160
FADD
37
25
20
FADD
Pac (nM)
0 10 20 40 80 160
GAPDH
50
37
25

Fig. 4c

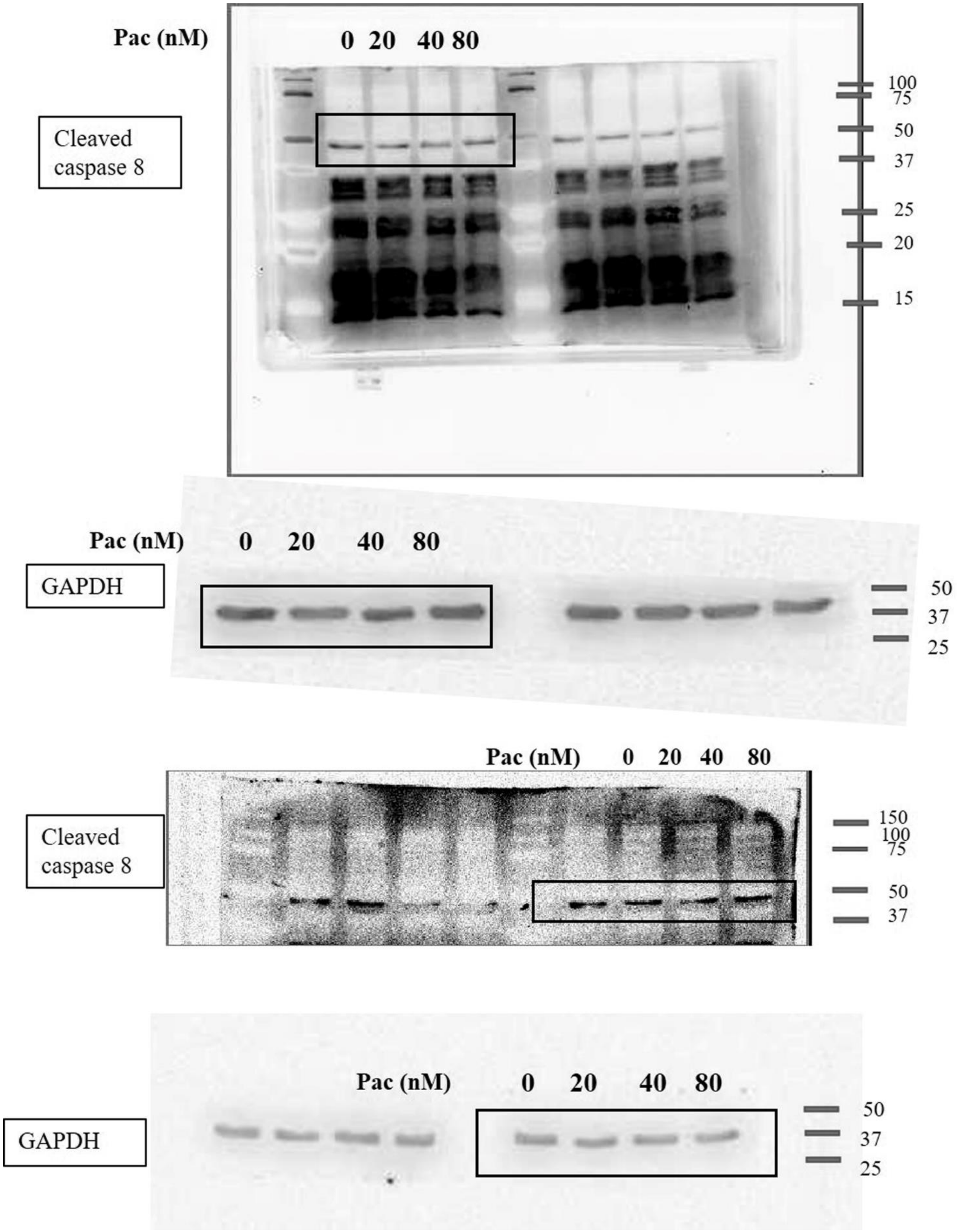

Pac (nM)
0 20 40 80
Cleaved caspase 8
100
75
50
37
25
20
15
Pac (nM)
0 20 40 80
GAPDH
50
37
25
Pac (nM)
0 20 40 80
Cleaved caspase 8
150
100
75
50
37
Pac (nM)
0 20 40 80
GAPDH
50
37
25

Supplementary Fig. E

Fig.E Photographs of the full-length blots described in Figure 5

Fig. 5c

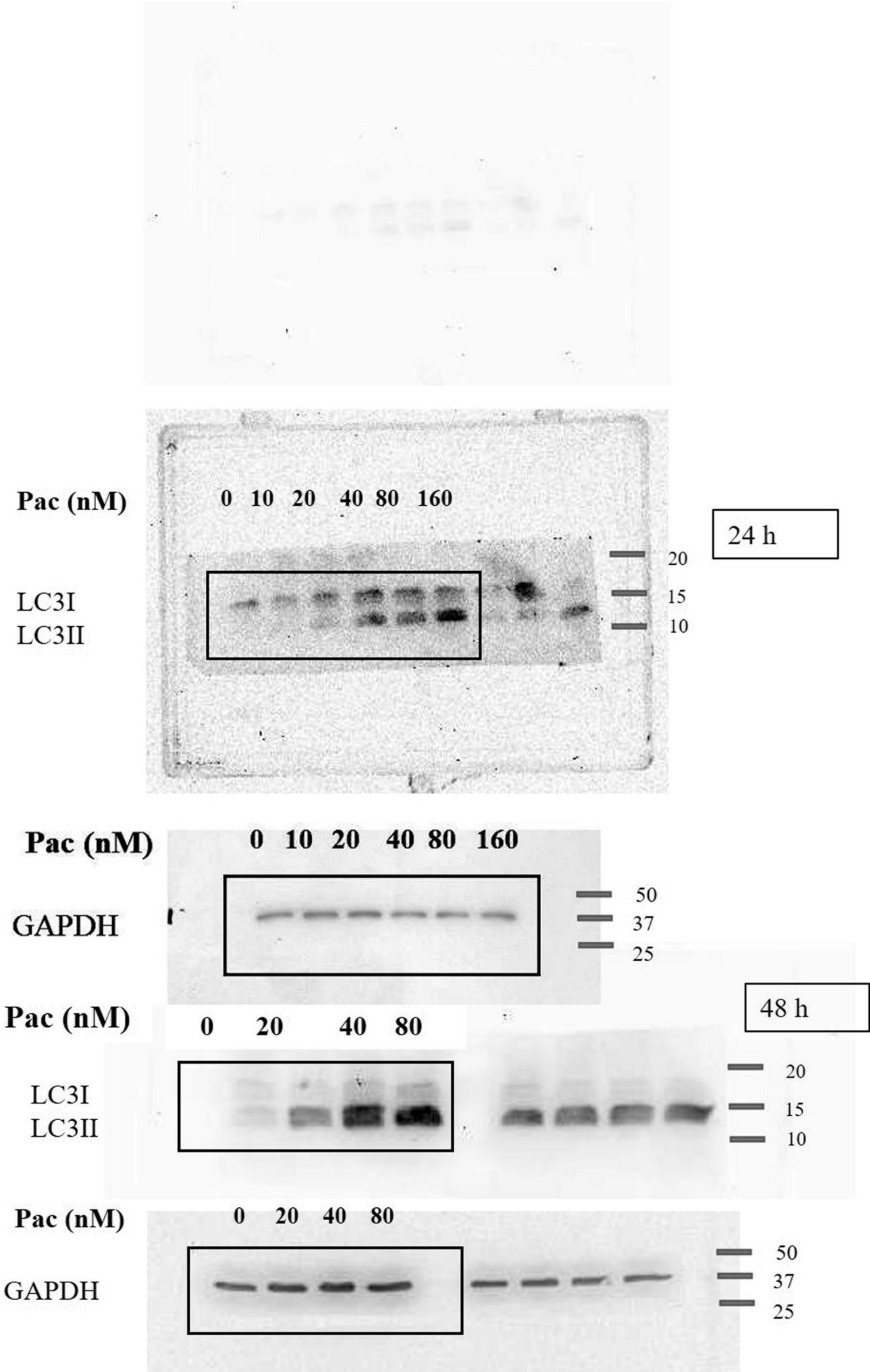

Fig. 5d

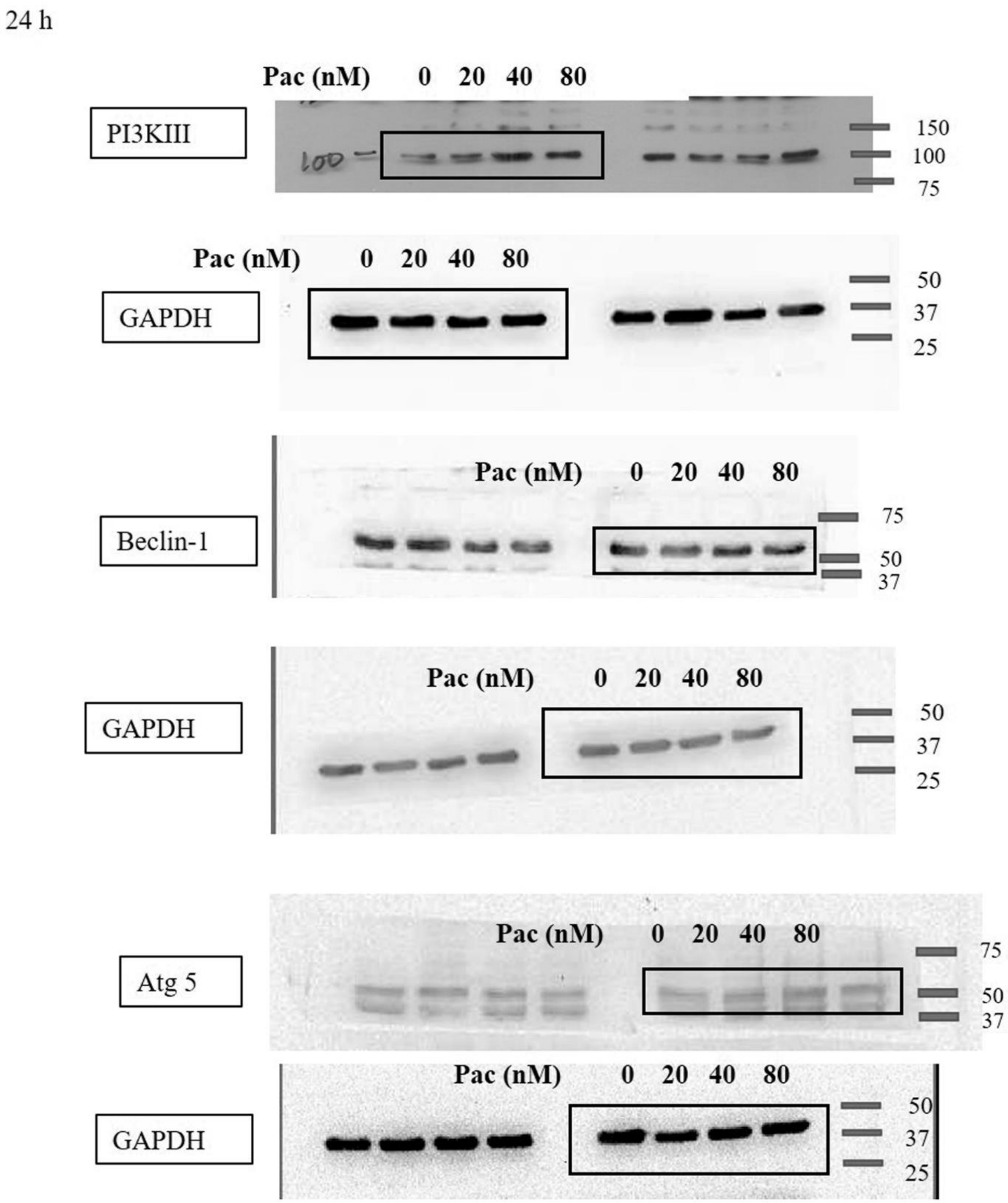
24 h
Pac (nM) 0 20 40 80
PI3KIII
150
100
75
Pac (nM) 0 20 40 80
GAPDH
50
37
25
Pac (nM) 0 20 40 80
Beclin-1
75
50
37
Pac (nM) 0 20 40 80
GAPDH
50
37
25
Pac (nM) 0 20 40 80
Atg 5
75
50
37
Pac (nM) 0 20 40 80
GAPDH
50
37
25

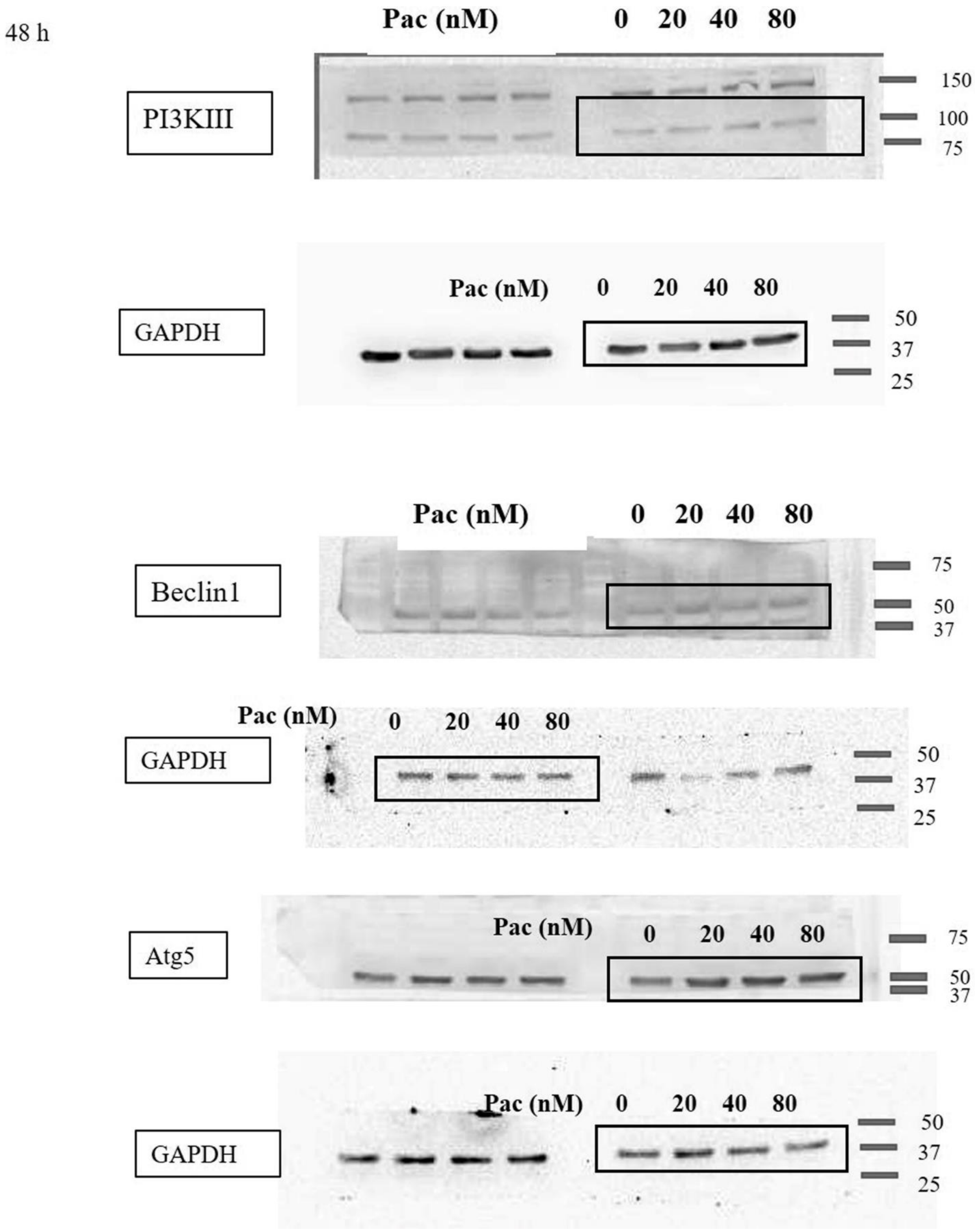
48 h
Pac (nM) 0 20 40 80
PI3KIII
150
100
75
Pac (nM) 0 20 40 80
GAPDH
50
37
25
Pac (nM) 0 20 40 80
Beclin1
75
50
37
Pac (nM) 0 20 40 80
GAPDH
50
37
25
Pac (nM) 0 20 40 80
Atg5
75
50
37
Pac (nM) 0 20 40 80
GAPDH
50
37
25

Fig. 5e

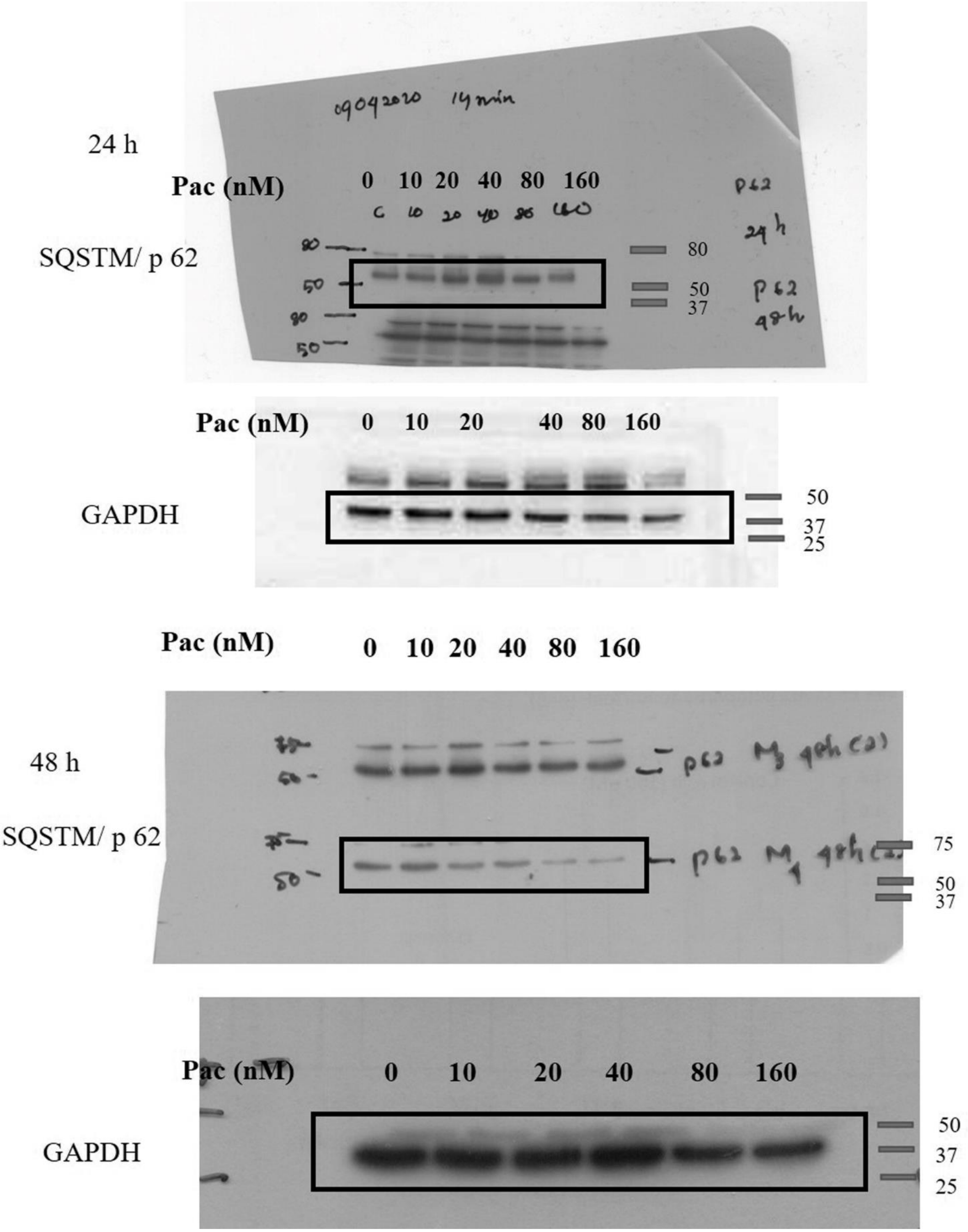
24 h
Pac (nM)
0 10 20 40 80 160
SQSTM/ p 62
80
50
37
Pac (nM)
0 10 20 40 80 160
GAPDH
50
37
25
Pac (nM)
0 10 20 40 80 160
48 h
SQSTM/ p 62
75
50
37
Pac (nM)
0 10 20 40 80 160
GAPDH
50
37
25

Fig. 5f

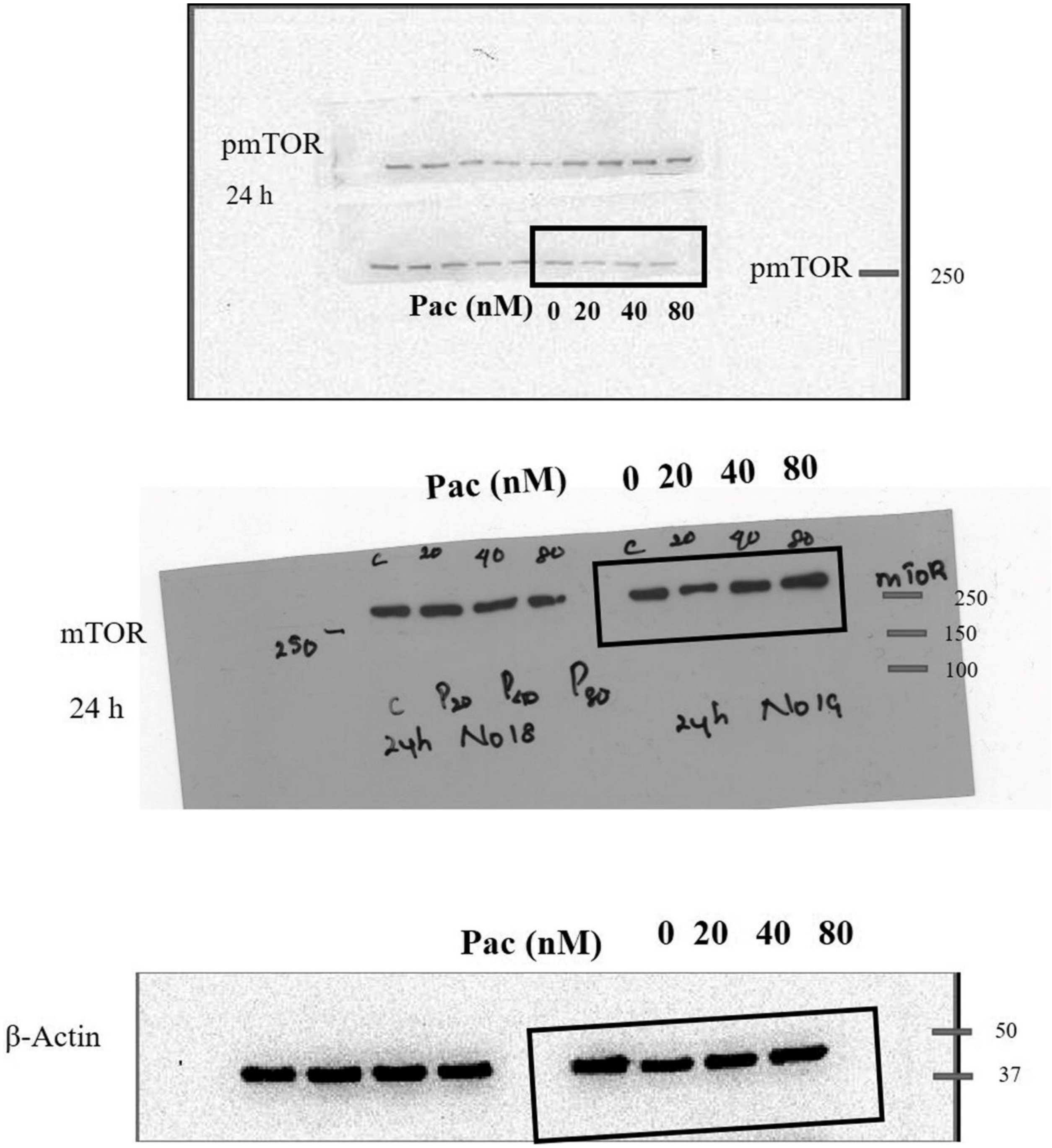
pmTOR
24 h
pmTOR
250
Pac (nM) 0 20 40 80
Pac (nM) 0 20 40 80
mTOR
24 h
250
150
100
24h No18
24h No19
Pac (nM) 0 20 40 80
β-Actin
50
37

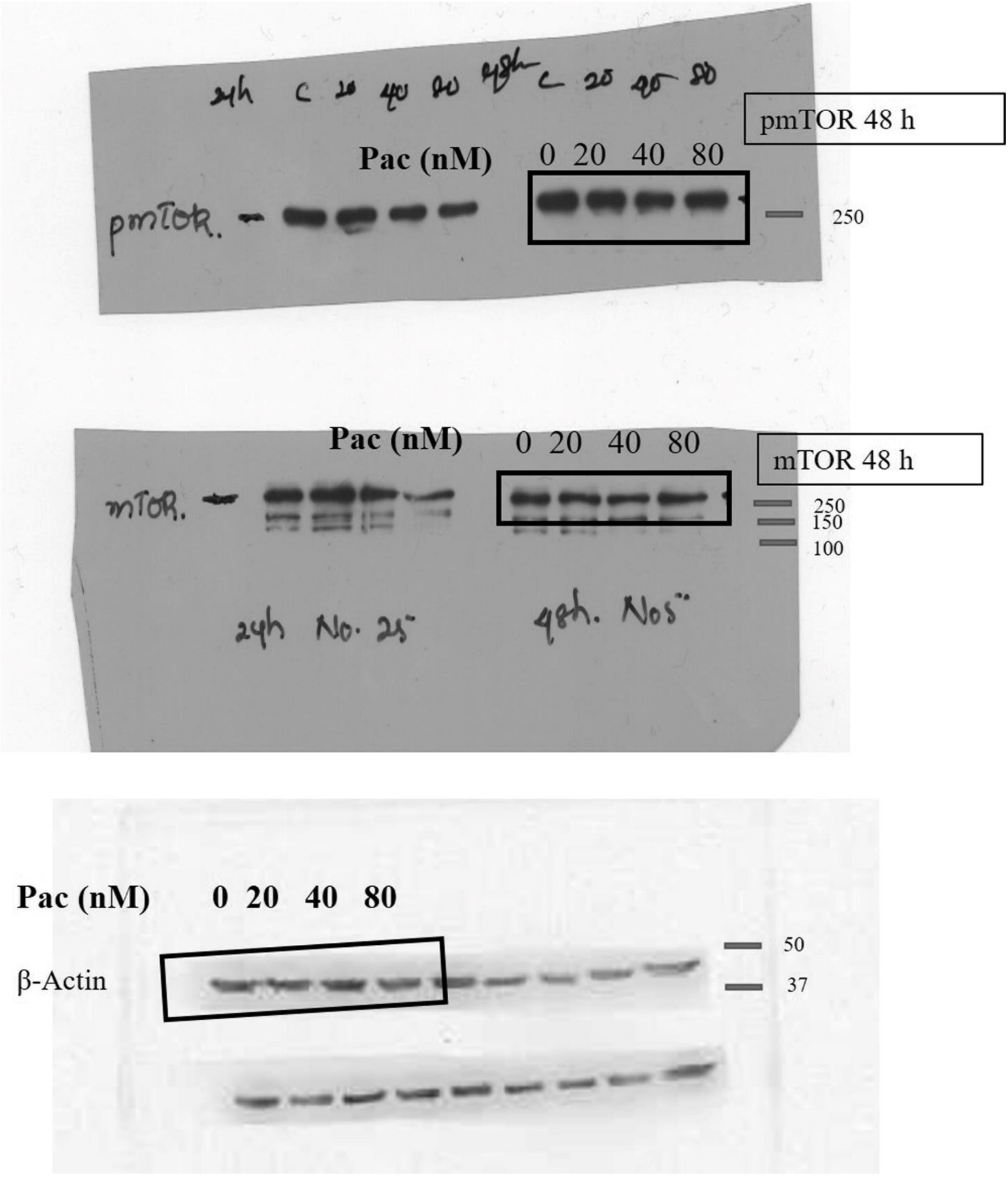
pmTOR 48 h
Pac (nM)
0 20 40 80
pmTOR.
250
Pac (nM)
0 20 40 80
mTOR 48 h
mTOR.
250
150
100
Pac (nM)
0 20 40 80
β-Actin
50
37

Fig. 5g

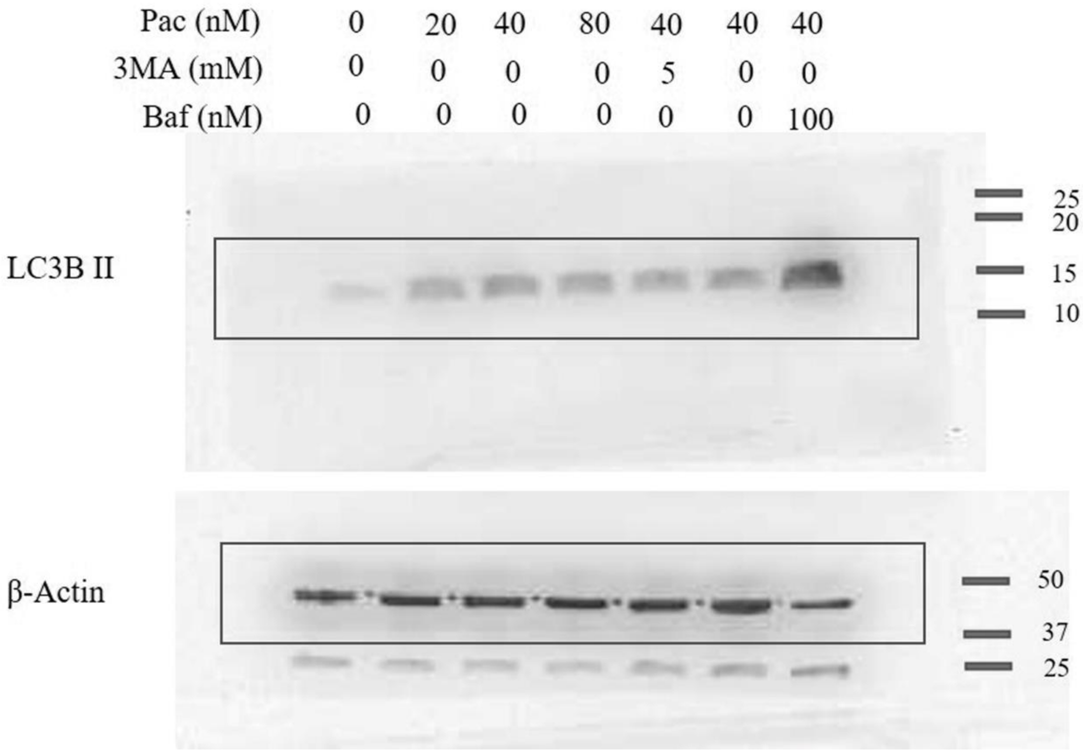
Pac (nM) 0 20 40 80 40 40 40
3MA (mM) 0 0 0 0 5 0 0
Baf (nM) 0 0 0 0 0 0 100
LC3B II
25
20
15
10
β-Actin
50
37
25

Supplementary Fig. F

Fig. F Photographs of the full-length blots described in Figure 6

Fig. 6b

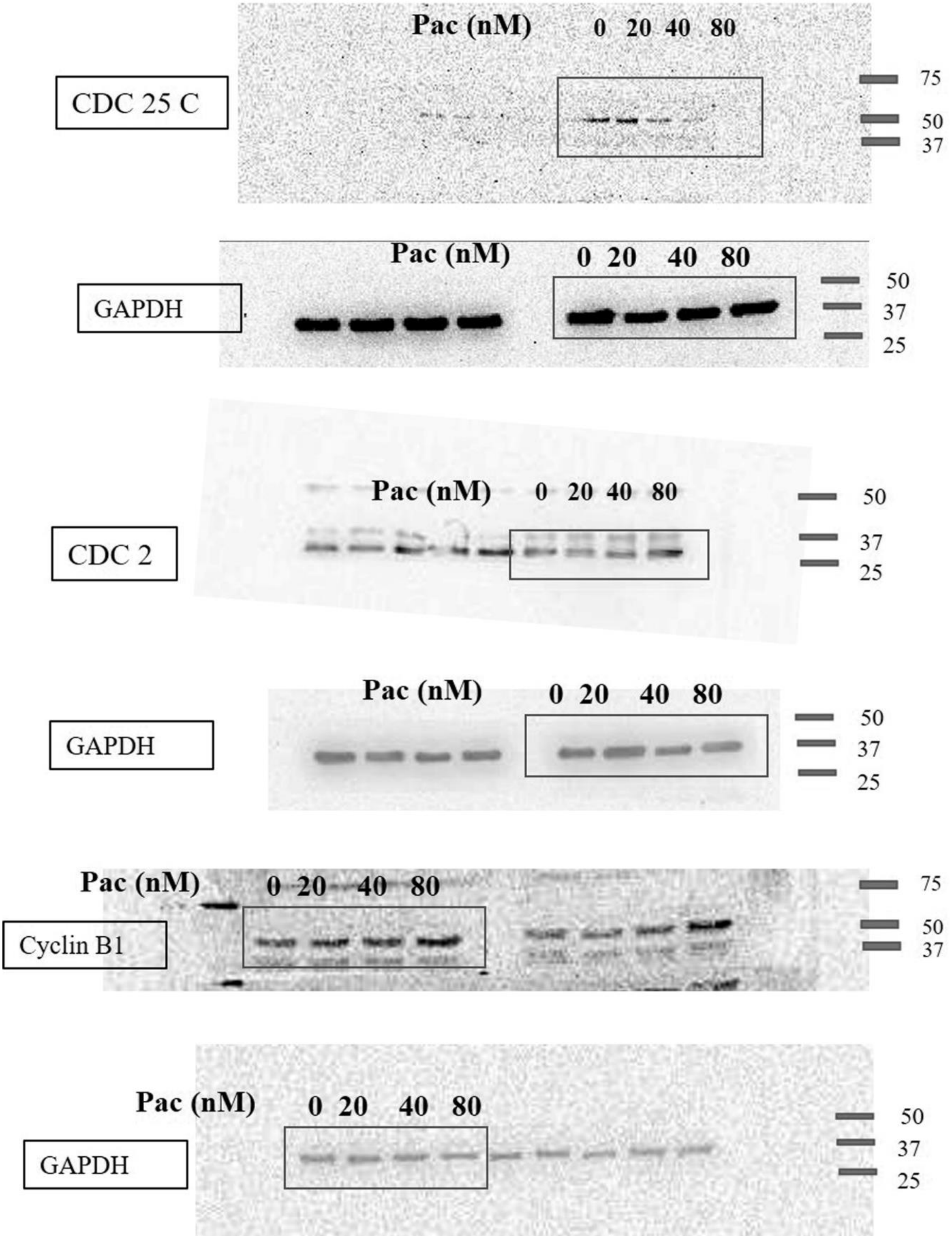

Fig. 6d

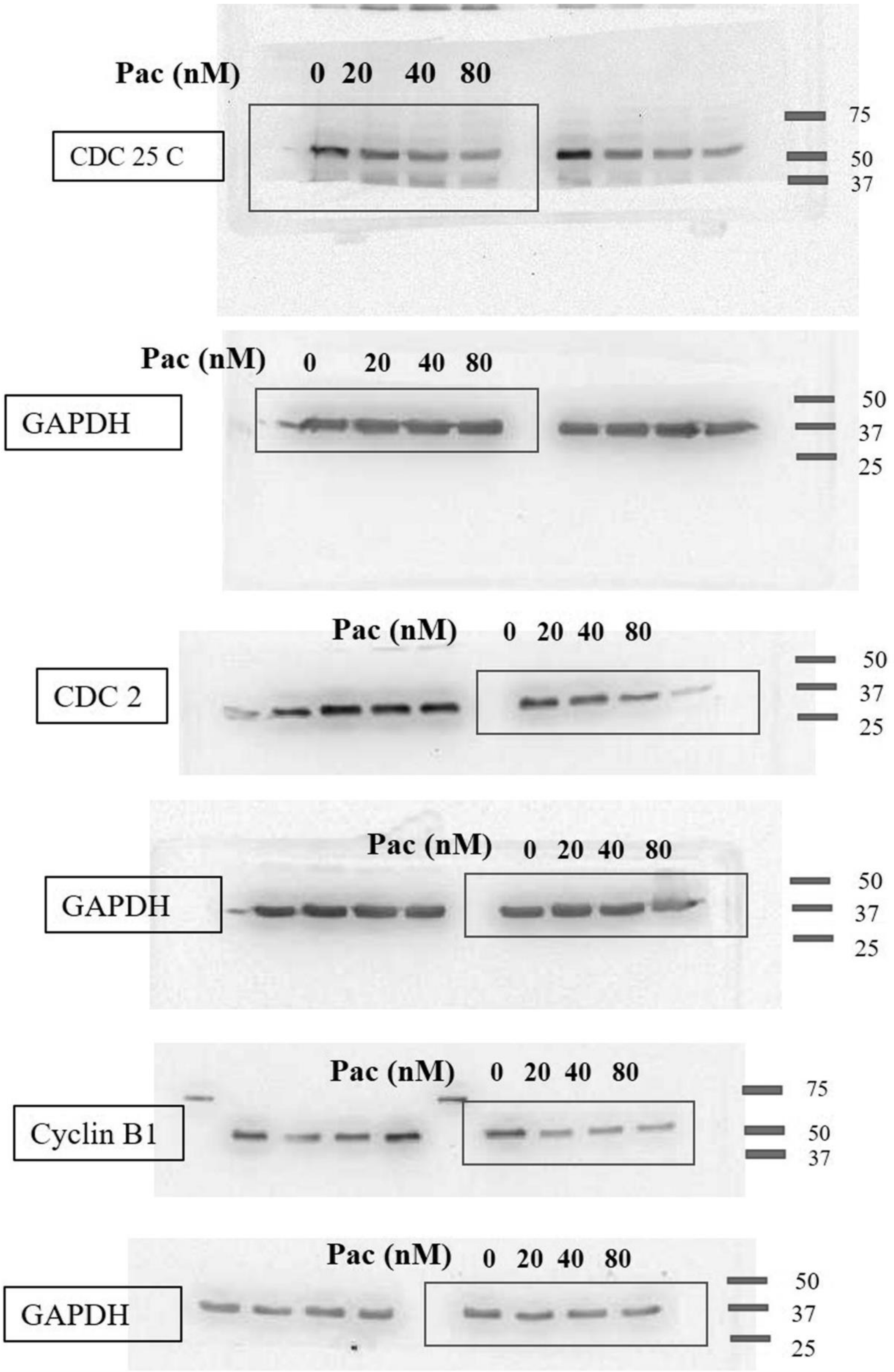
Pac (nM) 0 20 40 80
CDC 25 C
75
50
37
Pac (nM) 0 20 40 80
GAPDH
50
37
25
Pac (nM) 0 20 40 80
CDC 2
50
37
25
Pac (nM) 0 20 40 80
GAPDH
50
37
25
Pac (nM) 0 20 40 80
Cyclin B1
75
50
37
Pac (nM) 0 20 40 80
GAPDH
50
37
25

Fig. 7f

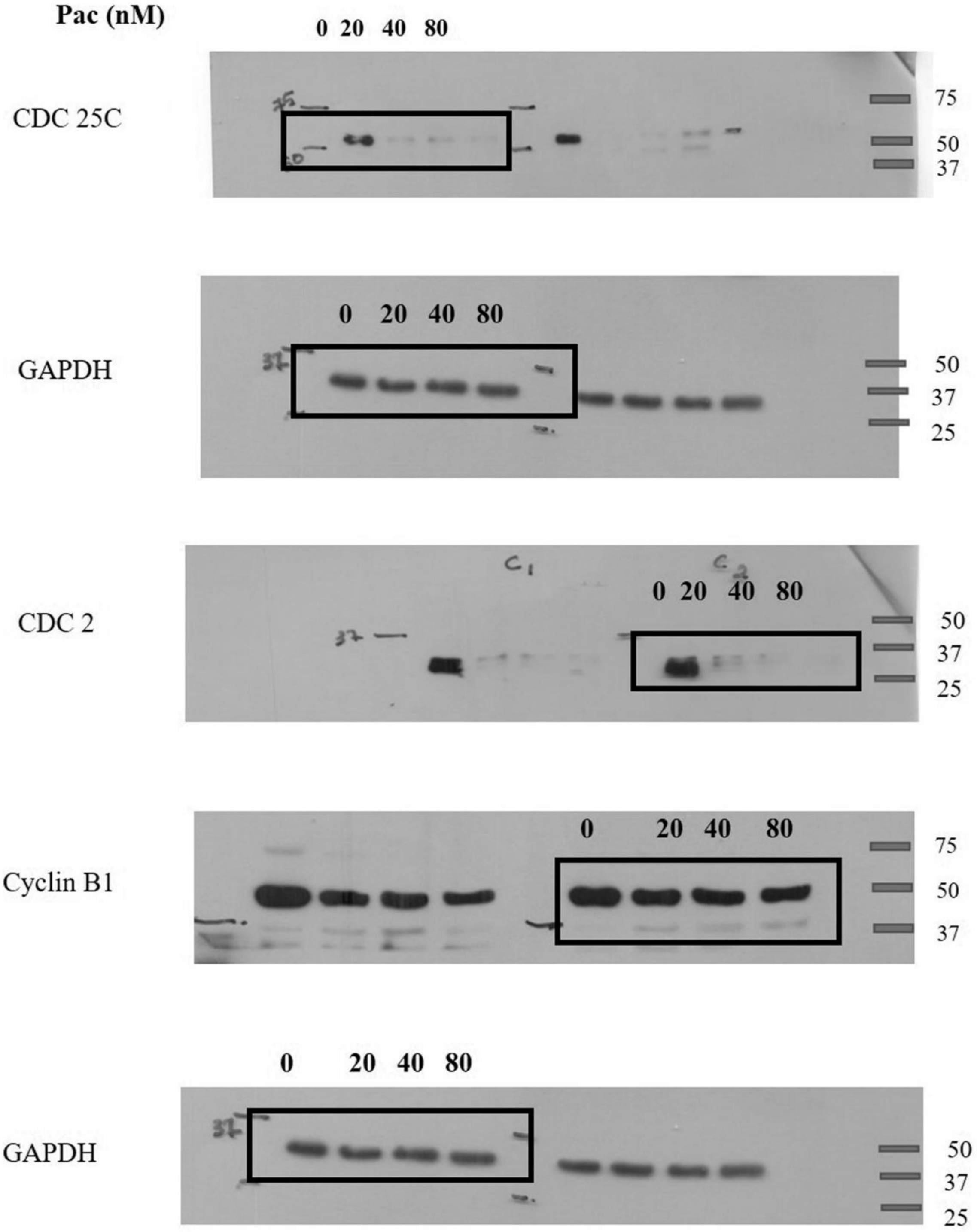
Pac (nM)
0 20 40 80
CDC 25C
75
50
37
0 20 40 80
GAPDH
50
37
25
CDC 2
0 20 40 80
50
37
25
Cyclin B1
0 20 40 80
75
50
37
0 20 40 80
GAPDH
50
37
25

Supplementary Fig. G

Fig. G Photographs of the full-length blots utilized in Figure 7

Fig. 7g

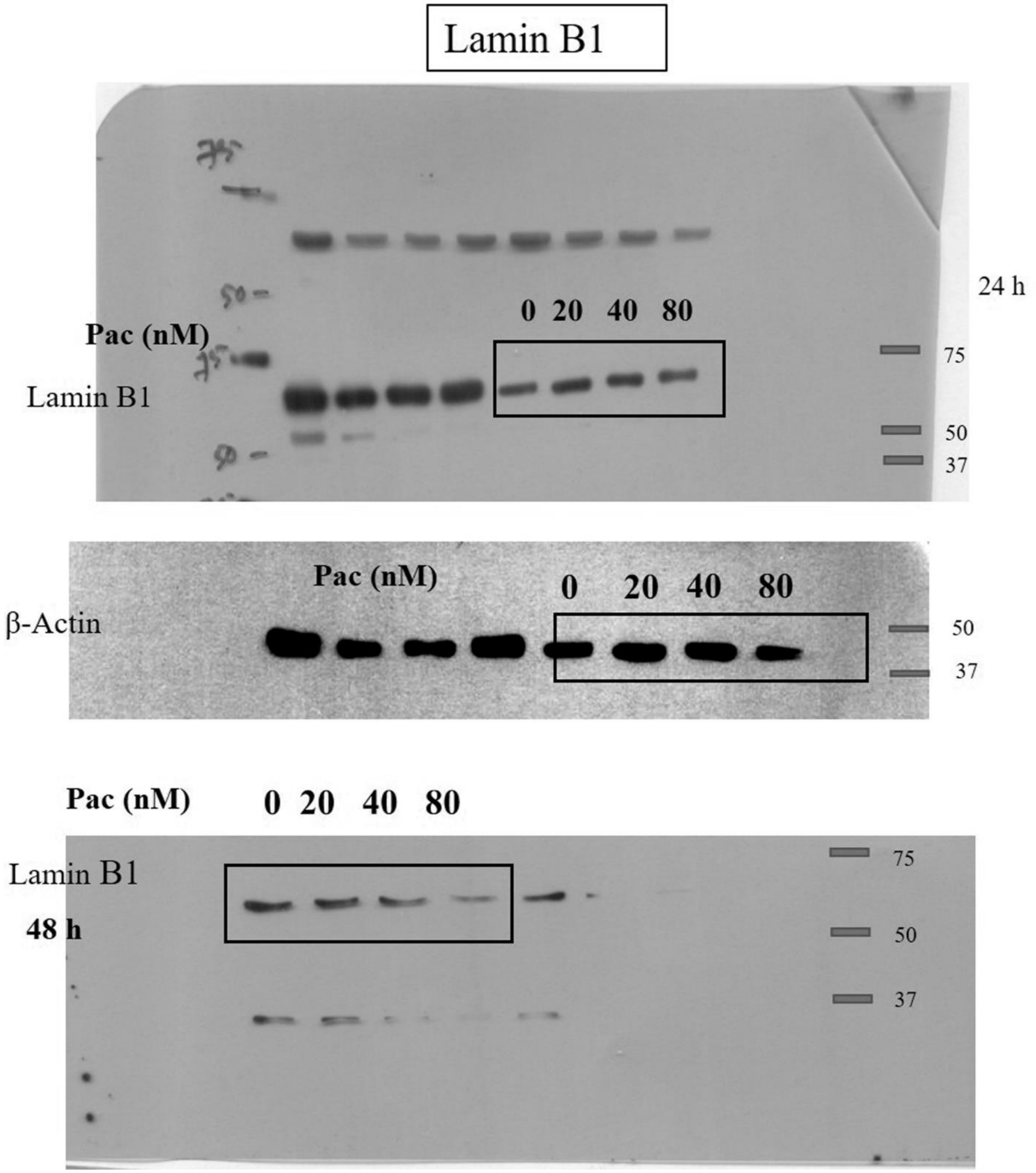

Lamin B1

48 h F

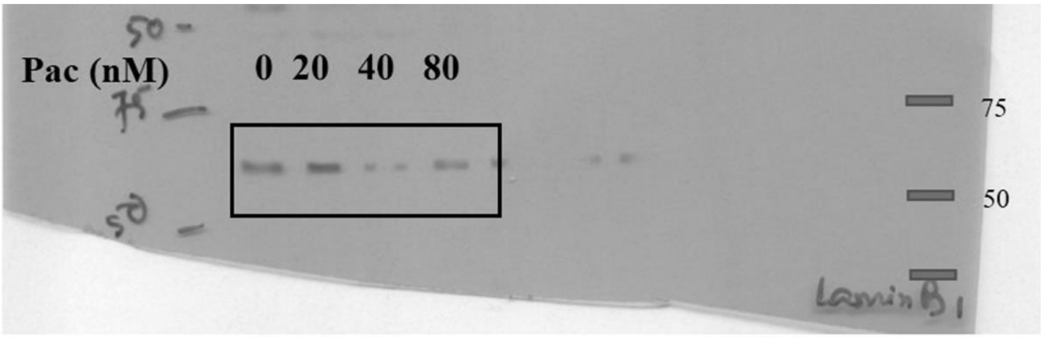

β-Actin

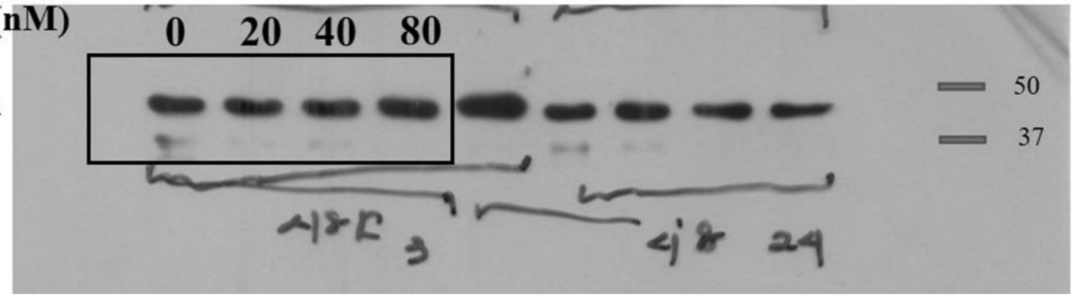

Fig. 7h

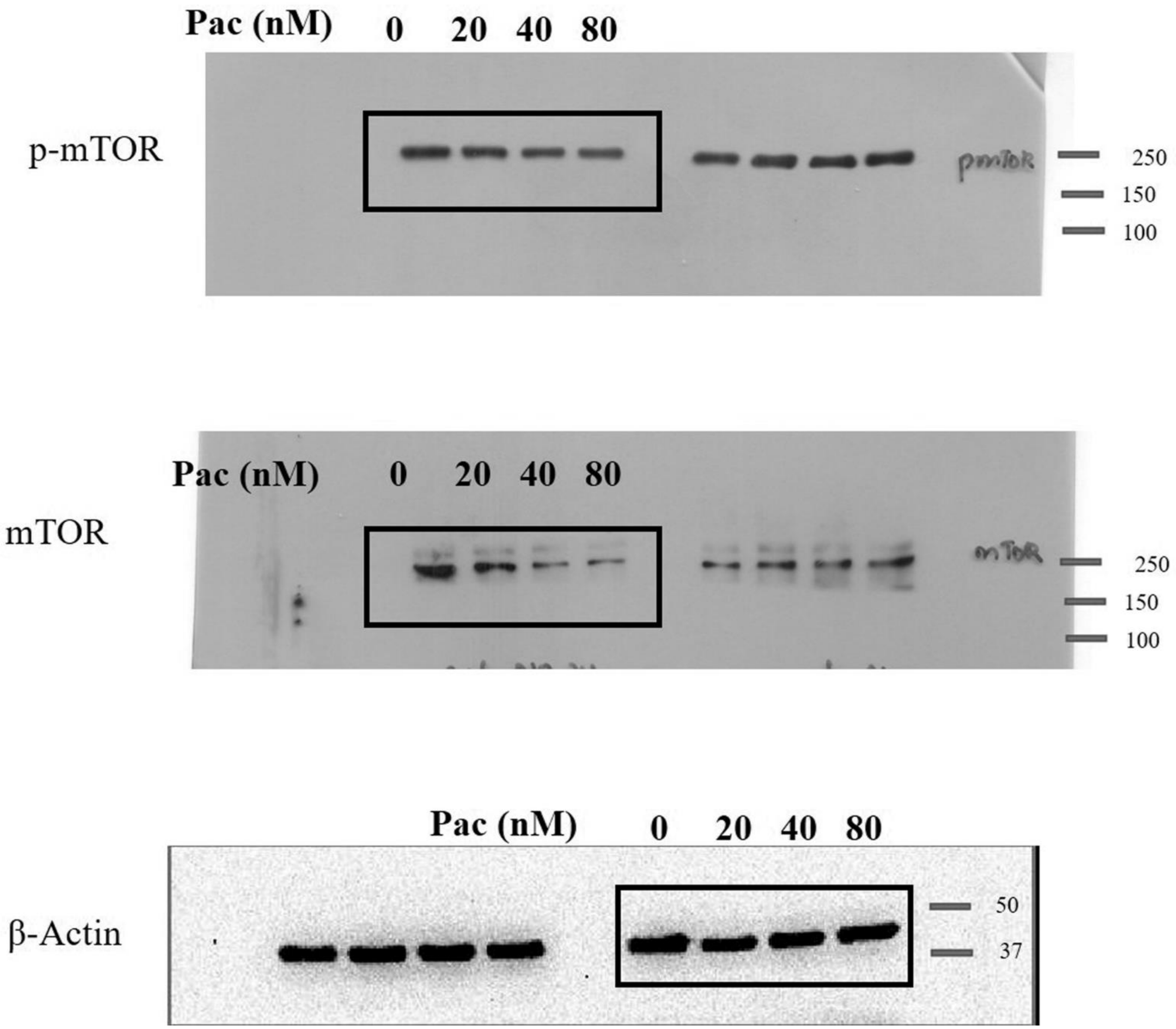
Pac (nM) 0 20 40 80
p-mTOR
pmTOR
250
150
100
Pac (nM) 0 20 40 80
mTOR
mTOR
250
150
100
Pac (nM) 0 20 40 80
β-Actin
50
37
