# Supplementary material for: The effect of paclitaxel on apoptosis, autophagy and mitotic catastrophe in AGS cells
Source: Sci Rep. 2021 Dec 6;11:23490. doi: 10.1038/s41598-021-02503-9 (PMC8648765; doi:10.1038/s41598-021-02503-9)

The effect of paclitaxel on apoptosis, autophagy and mitotic catastrophe in AGS cells

Tin Myo KHING, Won Seok CHOI, Dong Min KIM, Wah Wah PO, Wynn THEIN, Chang Yell SHIN and Uy Dong SOHN*

Laboratory of Signaling and Pharmacological Activity, Department of Pharmacology, College of Pharmacy, Chung-Ang University, Seoul, Republic of Korea

***Corresponding author:** Uy Dong Sohn, Professor, Ph.D., Department of Pharmacology, College of Pharmacy, Chung-Ang University, Seoul, 06974, Republic of Korea. Tel.: +82 28205614, Fax: 011 +8228268752, e-mail: udsohn@cau.ac.kr

Supporting Data

Fig. AA Photographs of the replicated full-length blots described in Figure 2

Fig.2b

Cleaved cas 3

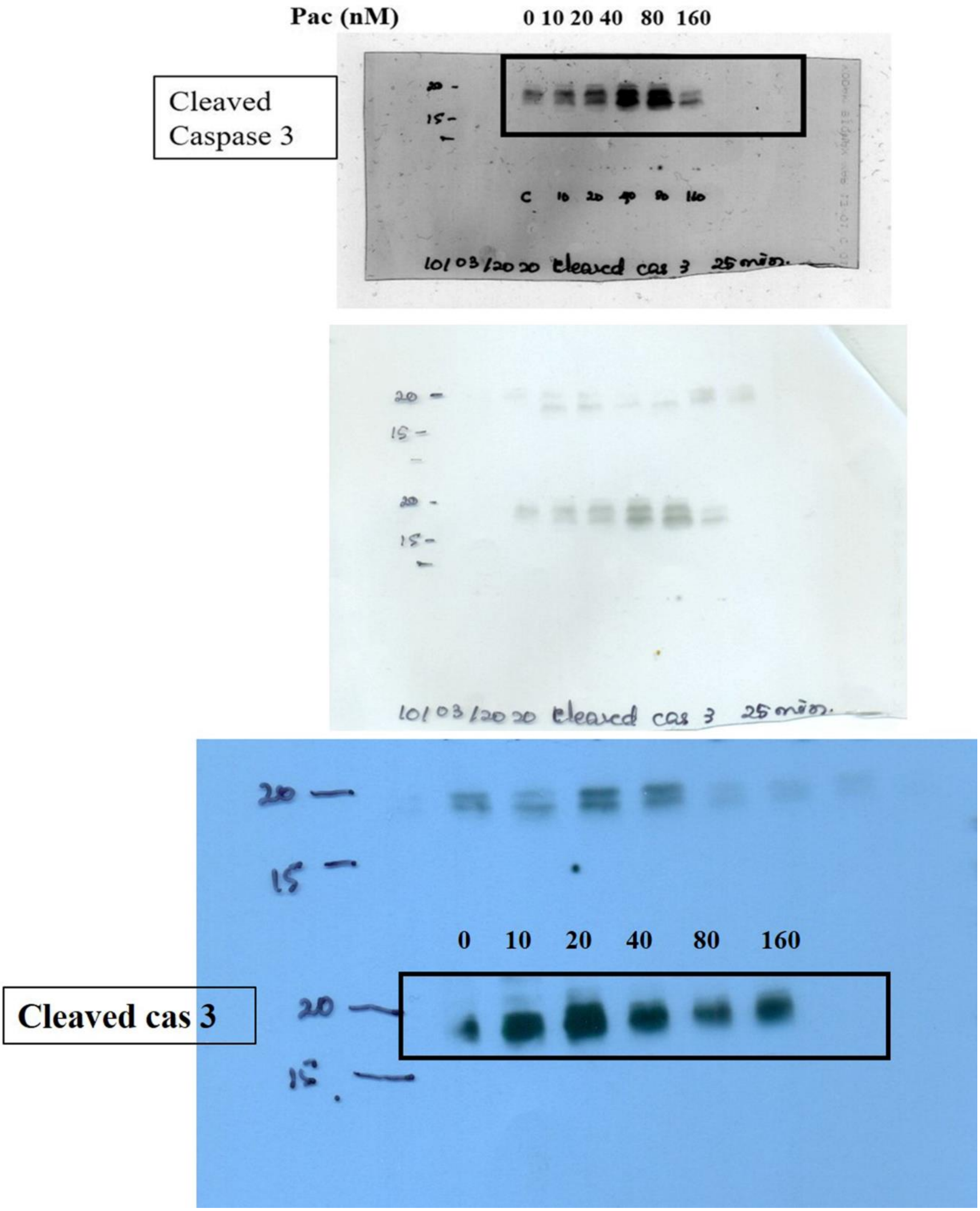

Cleaved cas 3

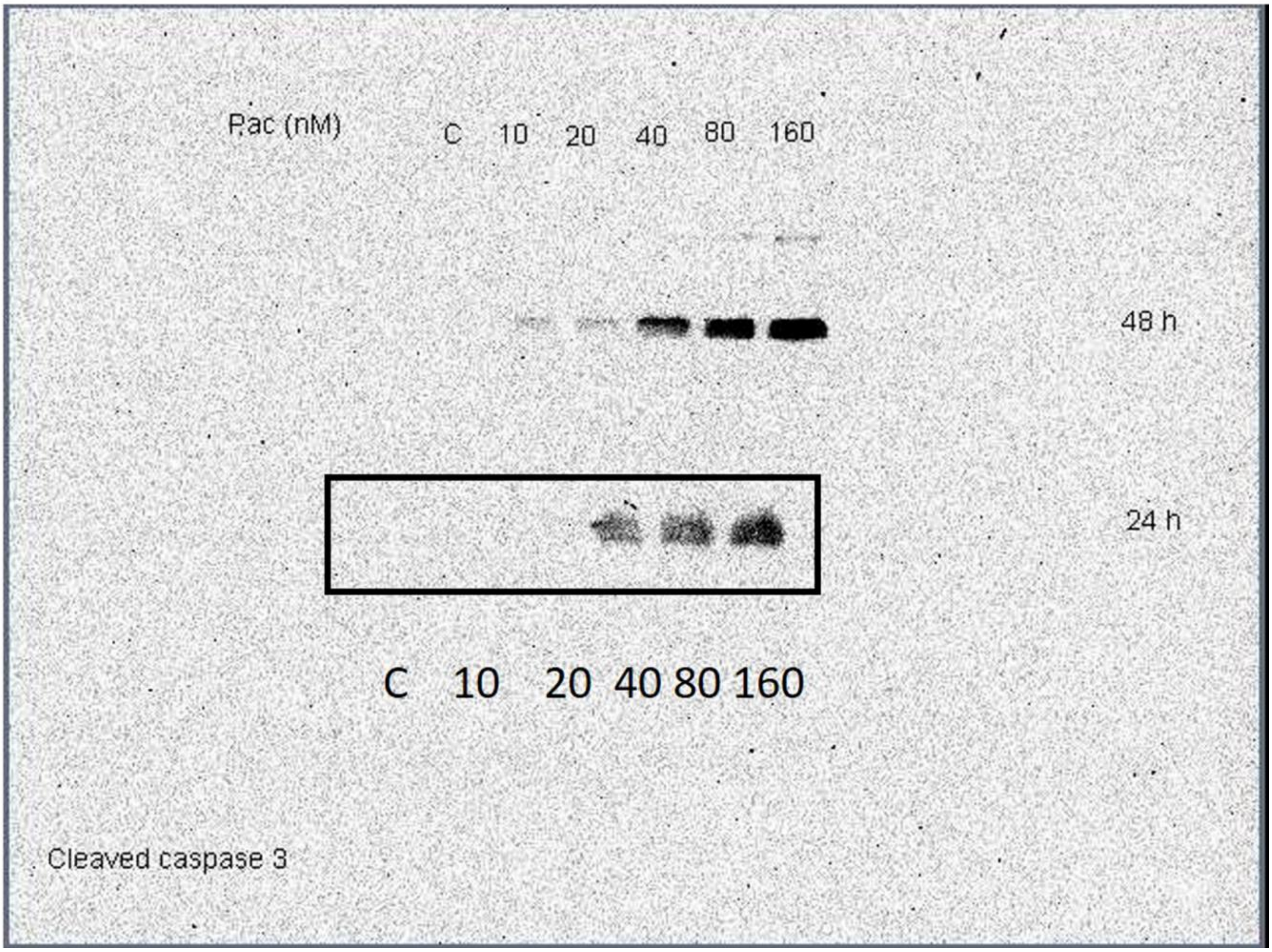

Procaspase-3

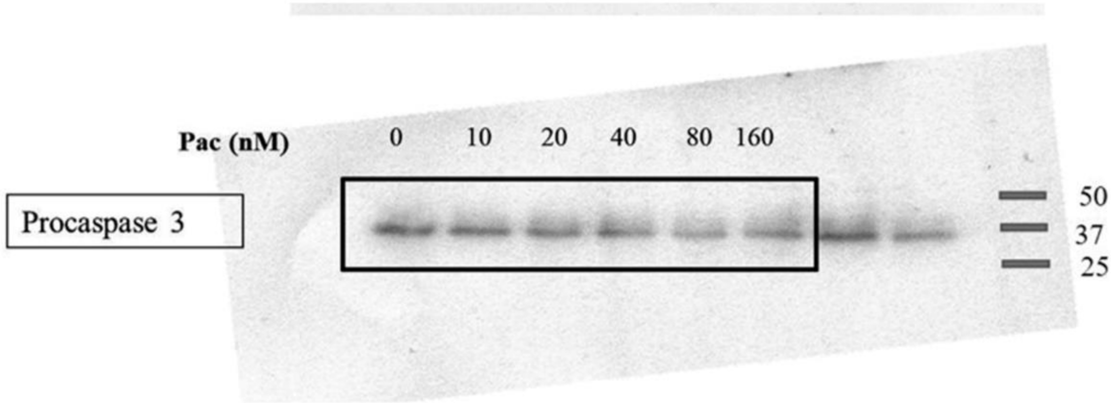

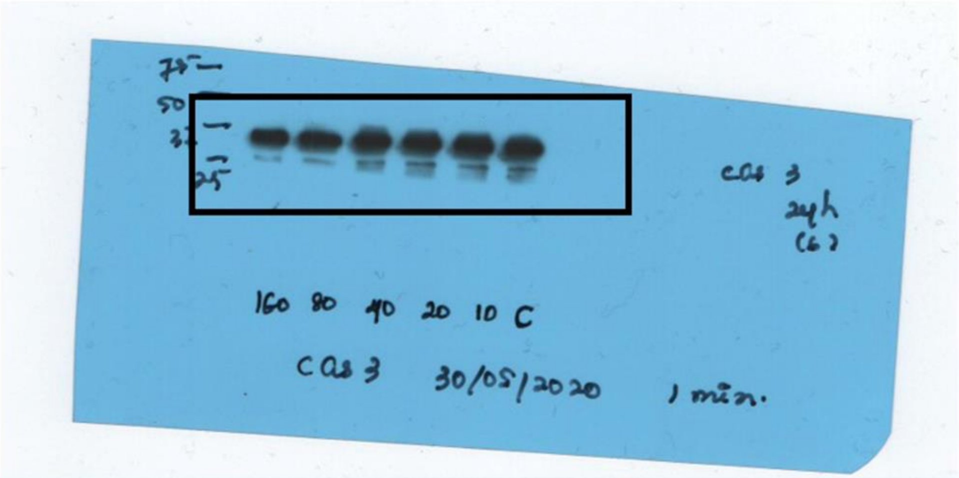

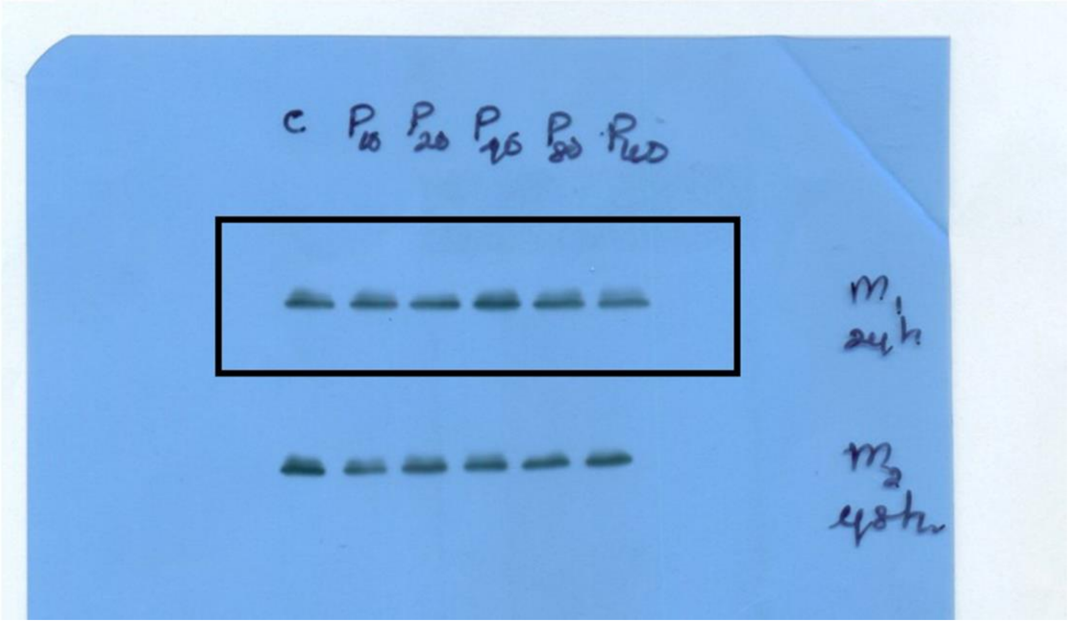

β-Actin

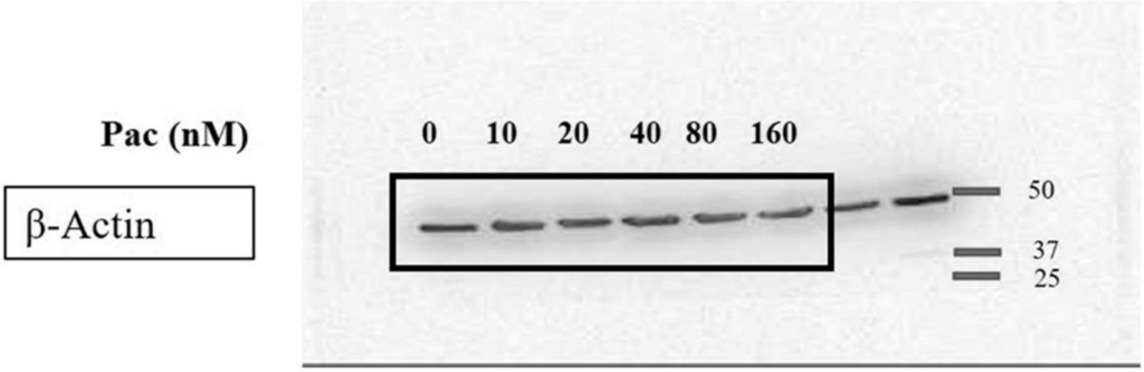
Pac (nM)
0 10 20 40 80 160
β-Actin
50
37
25

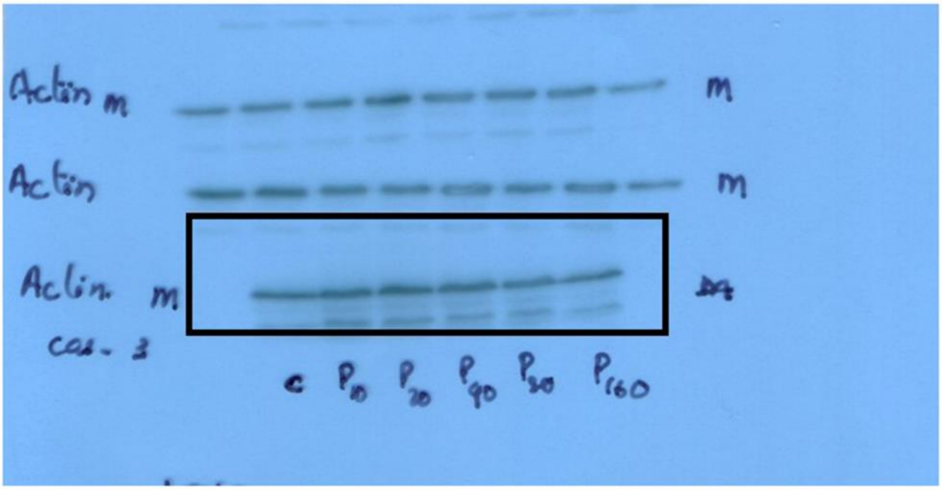
Actin m
m
Actin
m
Actin m
cas-3
c P10 P20 P40 P80 P160

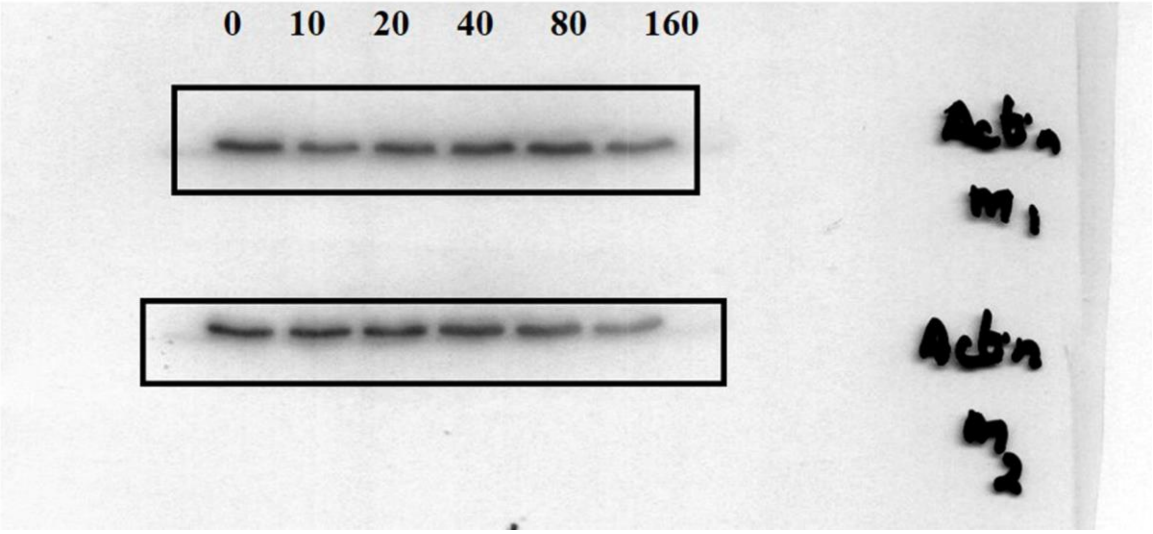
0 10 20 40 80 160
Actin m1
Actin m2

PARP

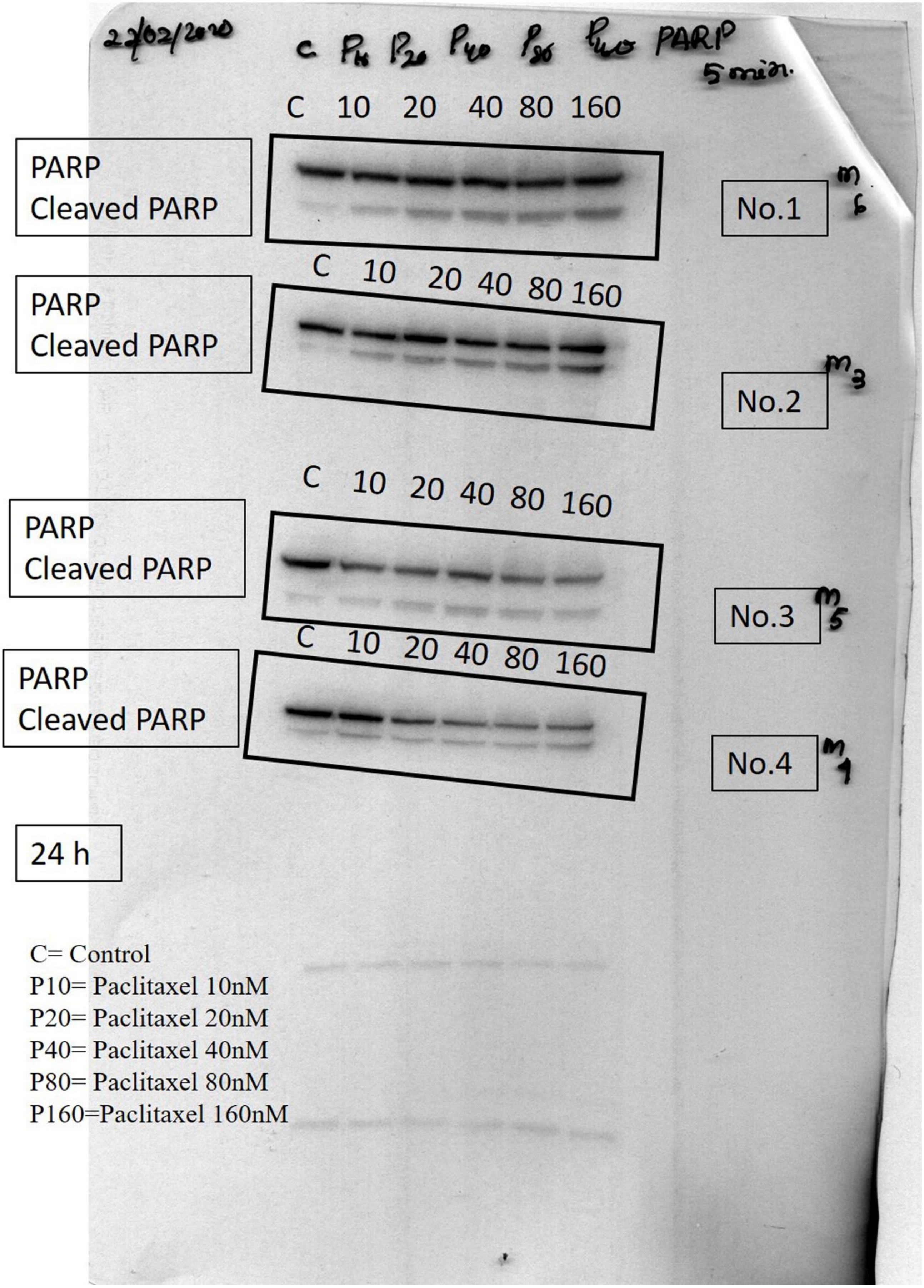

C= Control
P10= Paclitaxel 10nM
P20= Paclitaxel 20nM
P40= Paclitaxel 40nM
P80= Paclitaxel 80nM
P160=Paclitaxel 160nM

Fig.2d

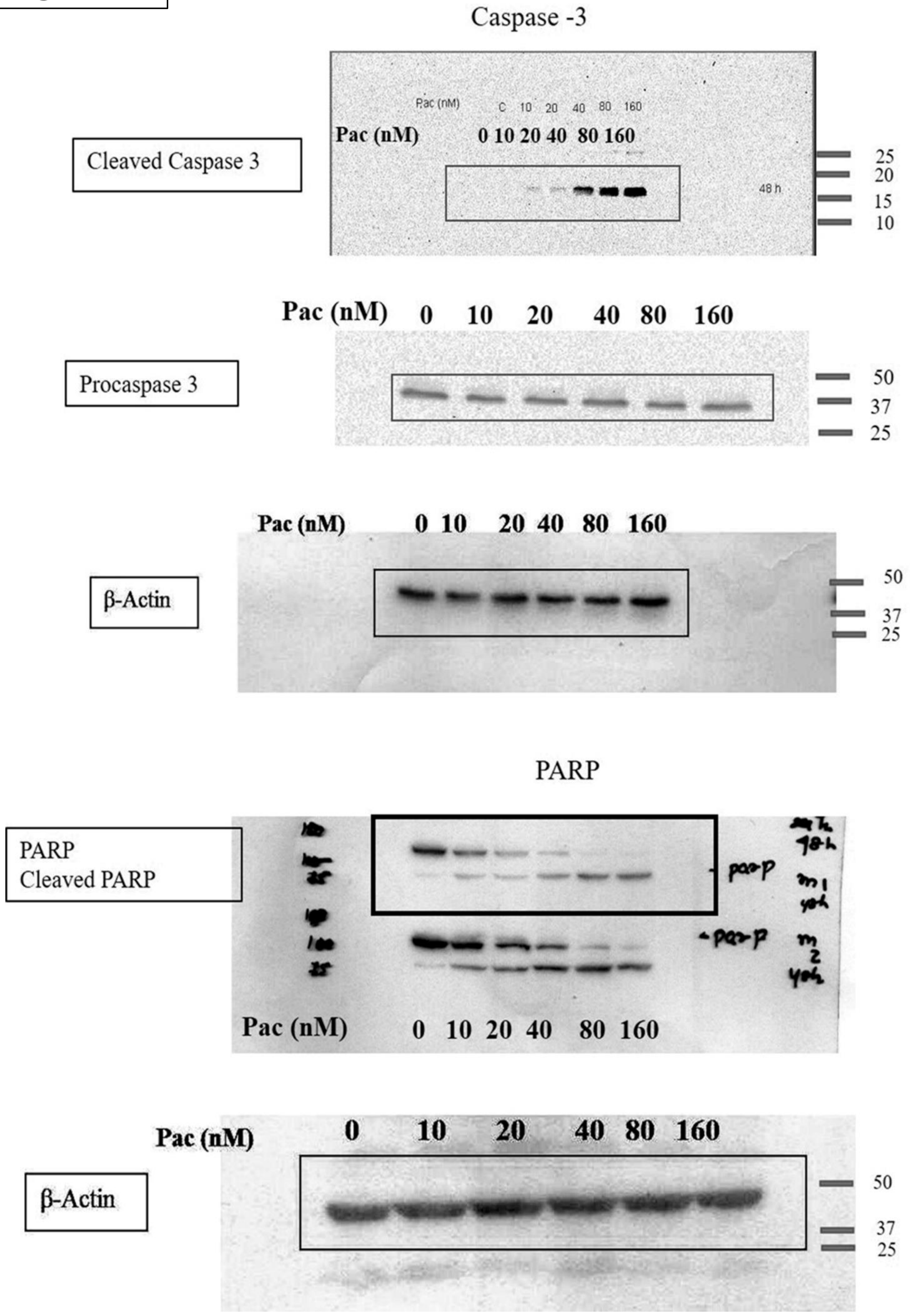

Caspase -3
Cleaved Caspase 3
Pac (nM) 0 10 20 40 80 160
25
20
15
10
Pac (nM) 0 10 20 40 80 160
Procaspase 3
50
37
25
Pac (nM) 0 10 20 40 80 160
β-Actin
50
37
25
PARP
PARP
Cleaved PARP
Pac (nM) 0 10 20 40 80 160
Pac (nM) 0 10 20 40 80 160
β-Actin
50
37
25

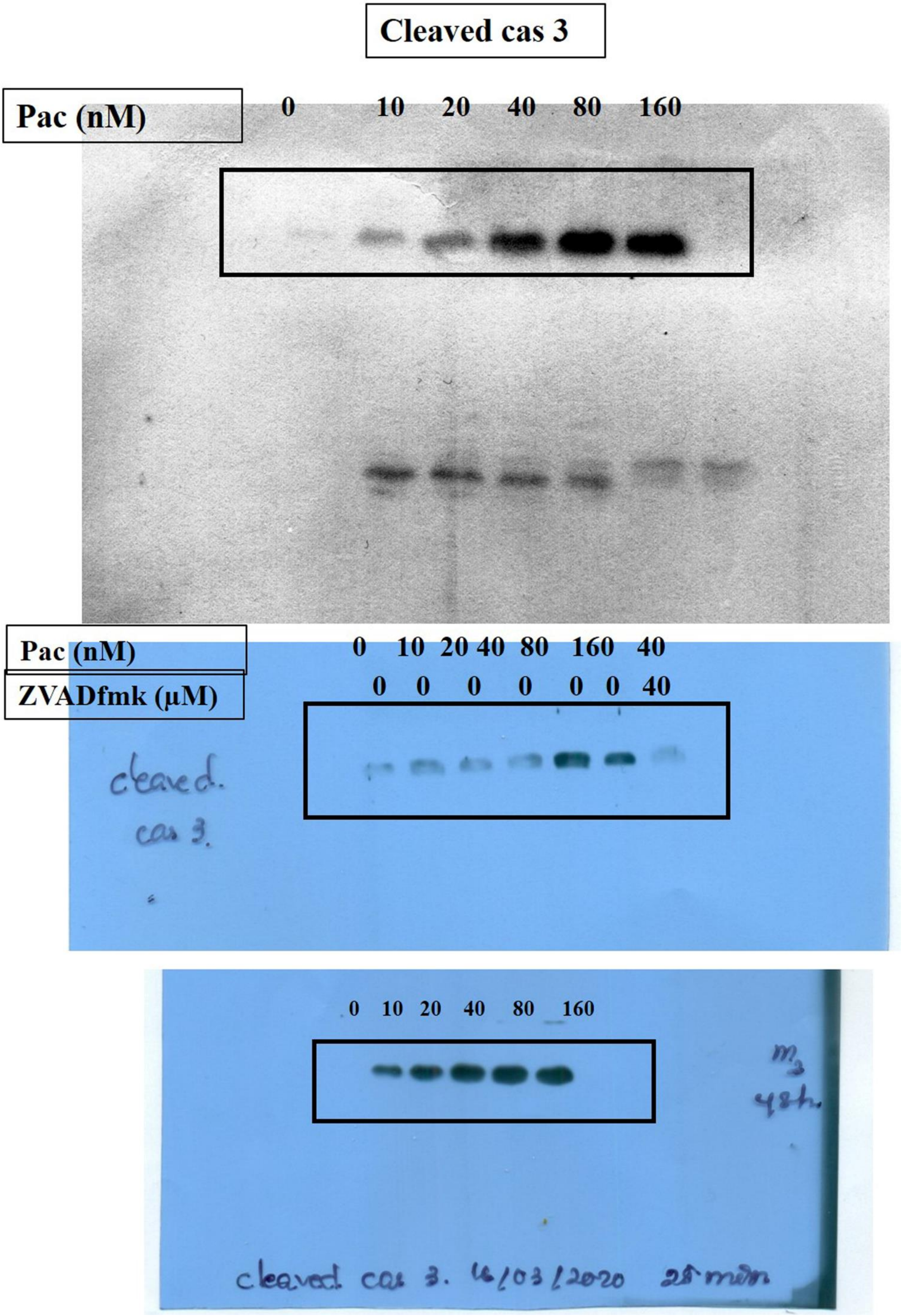

Cleaved cas 3
Pac (nM)
0 10 20 40 80 160
Pac (nM)
0 10 20 40 80 160 40
ZVADfmk (μM)
0 0 0 0 0 0 40
cleaved. cas 3.
0 10 20 40 80 160
m3 48h.
cleaved cas 3. 16/03/2020 25 min

Cleaved cas 3

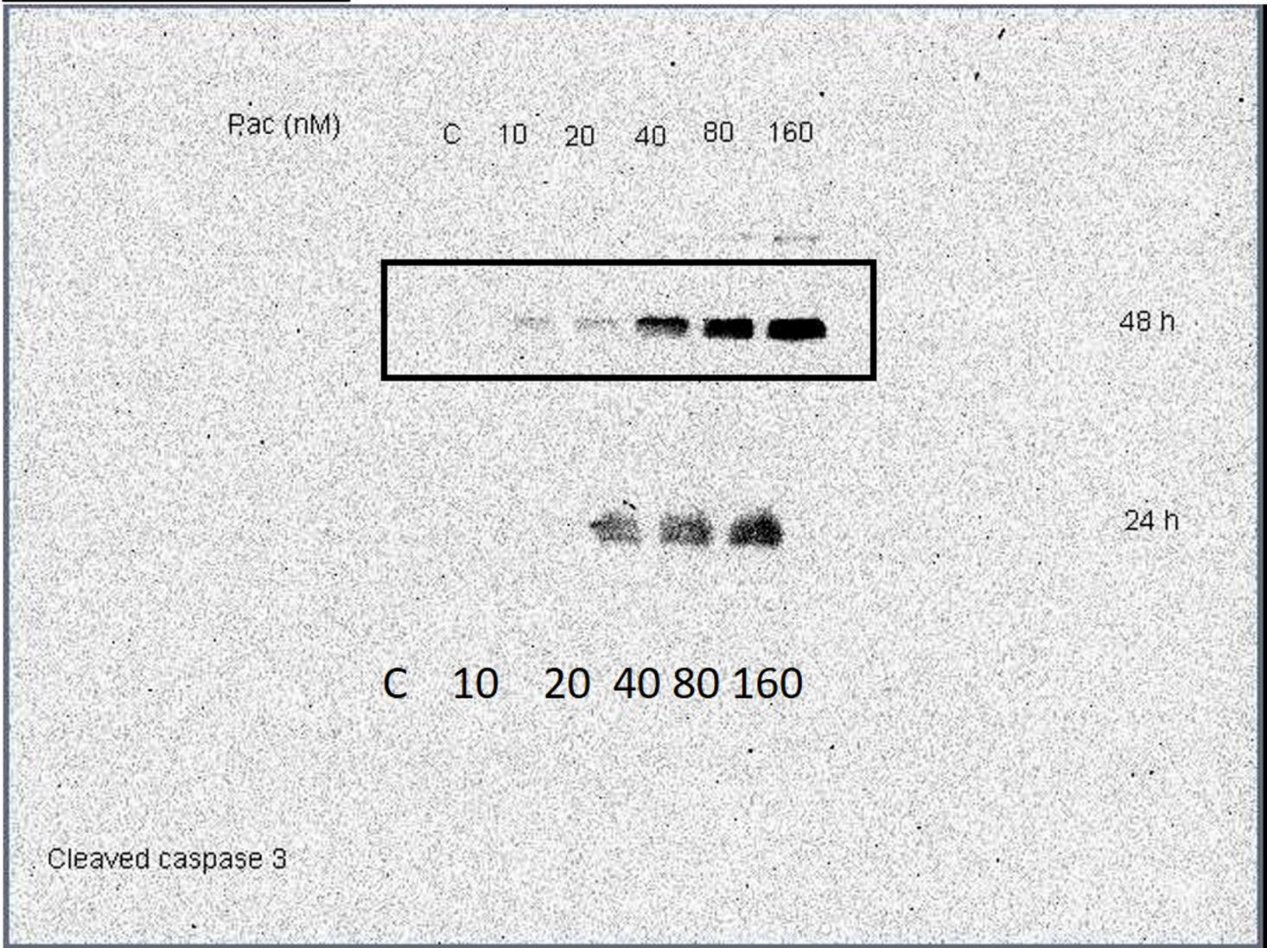

Procaspase-3

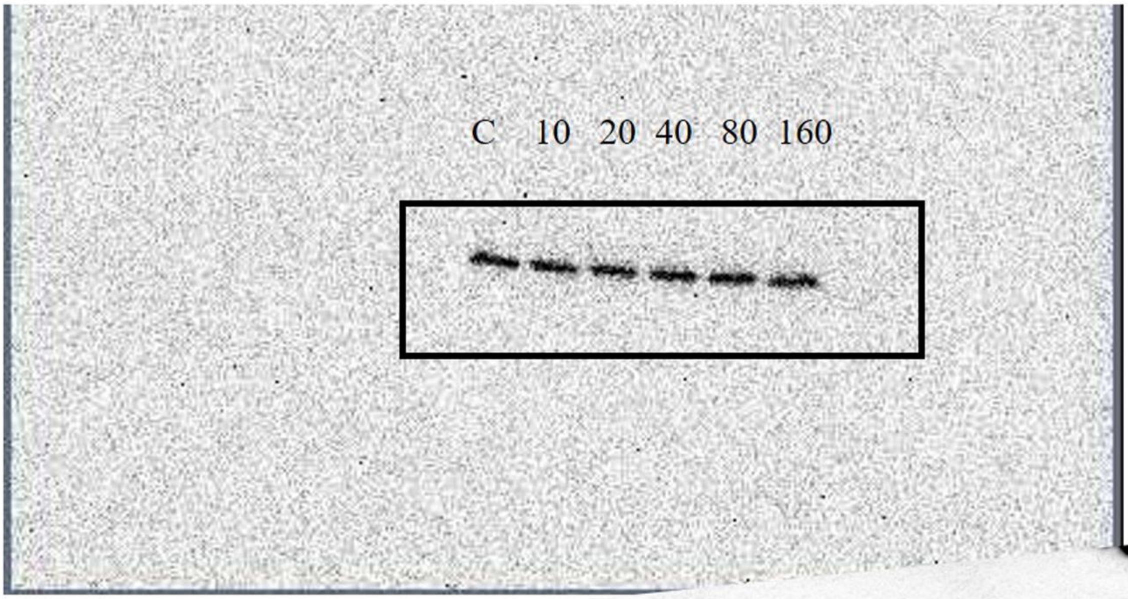

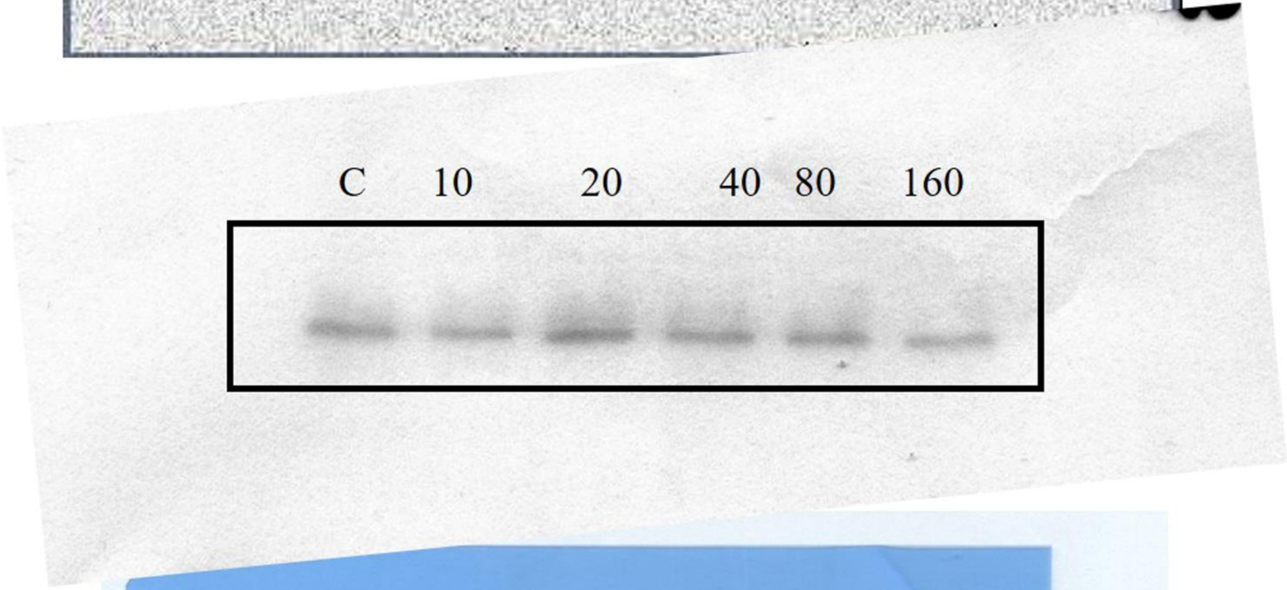

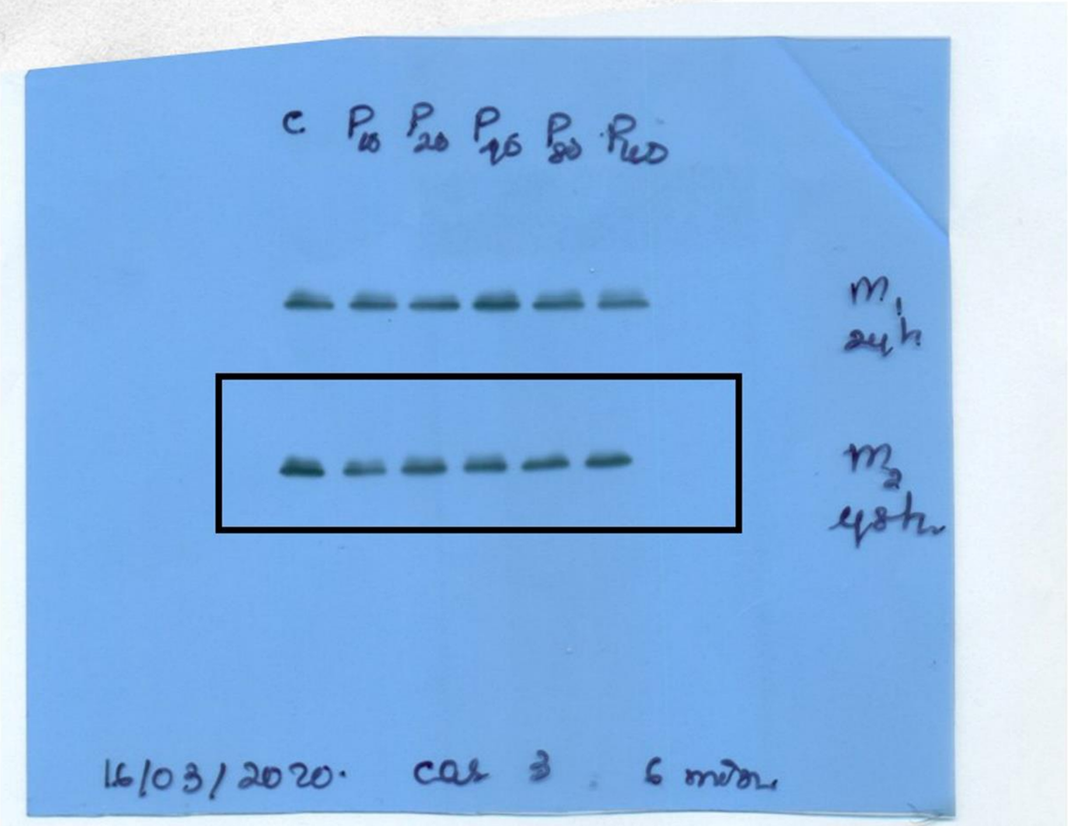

PARP

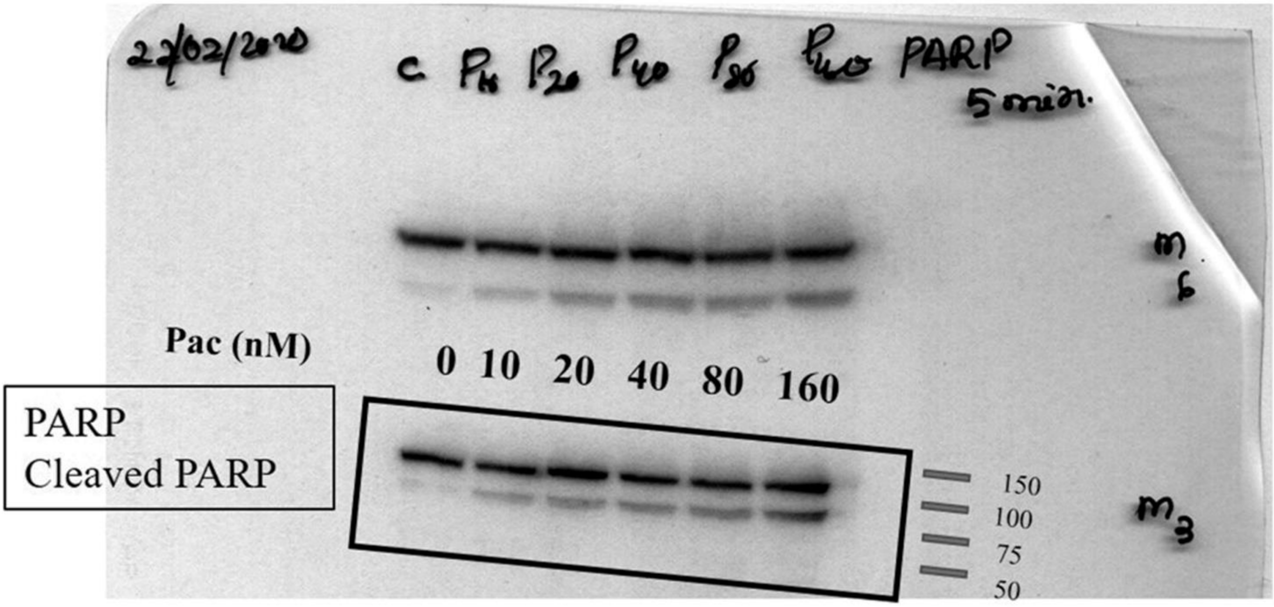
Pac (nM)
0 10 20 40 80 160
PARP
Cleaved PARP
150
100
75
50

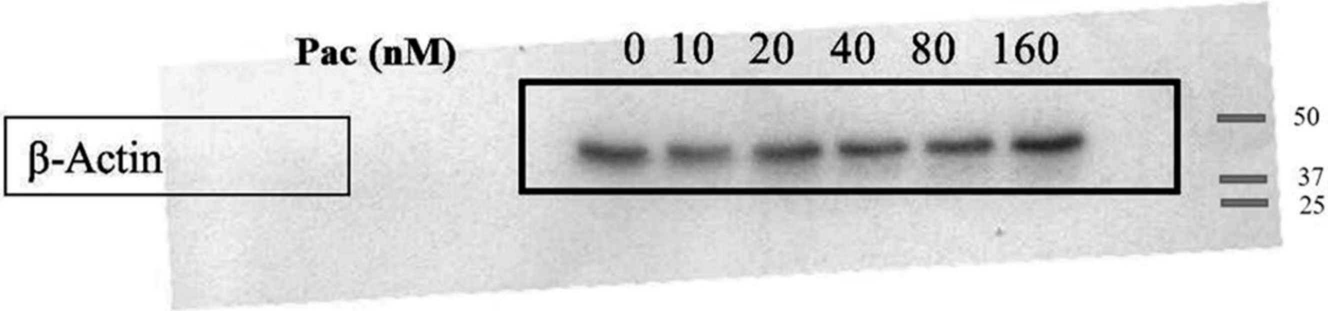
Pac (nM)
0 10 20 40 80 160
β-Actin
50
37
25

Figure. 2d

PARP

48 h

PARP
Cleaved PARP

PARP
Cleaved PARP

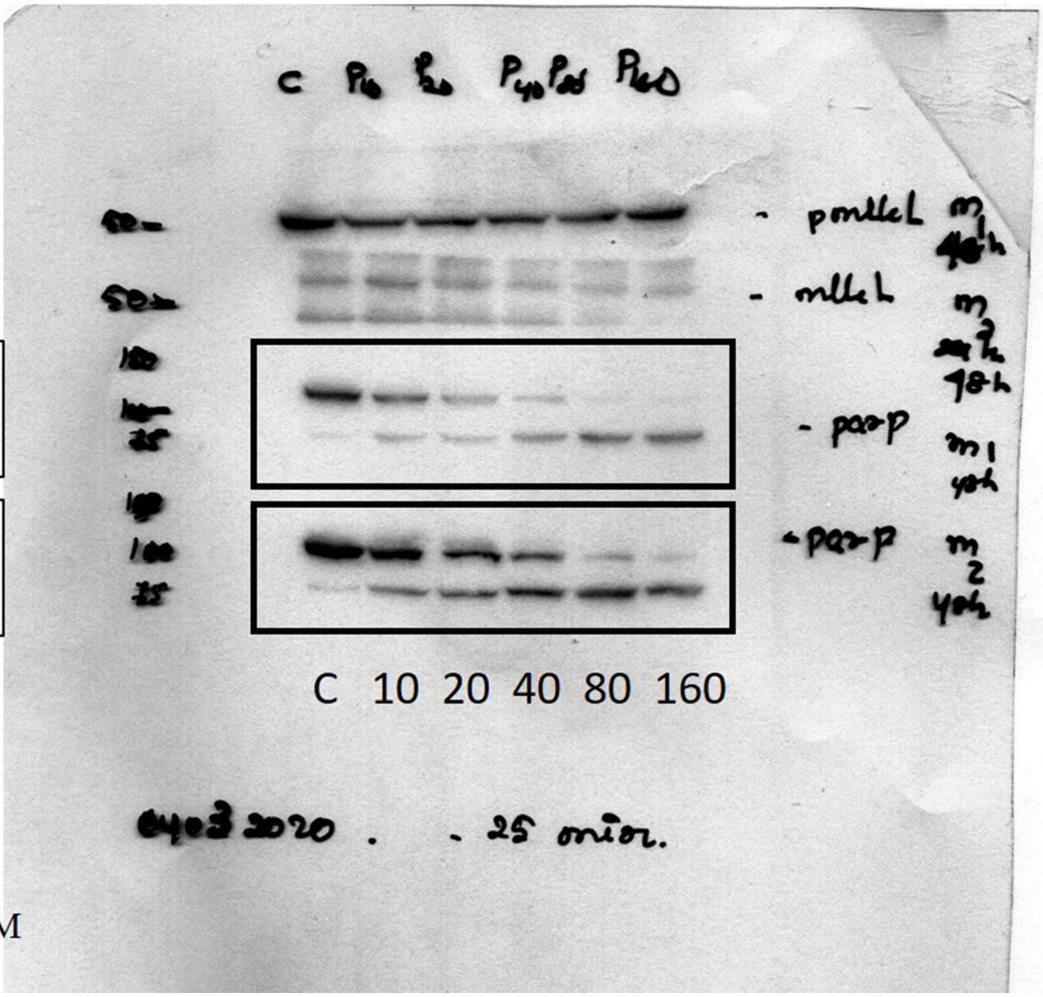

C= Control
10= Paclitaxel 10nM
20= Paclitaxel 20nM
40= Paclitaxel 40nM
80= Paclitaxel 80nM
160=Paclitaxel 160nM

Figure. 2d

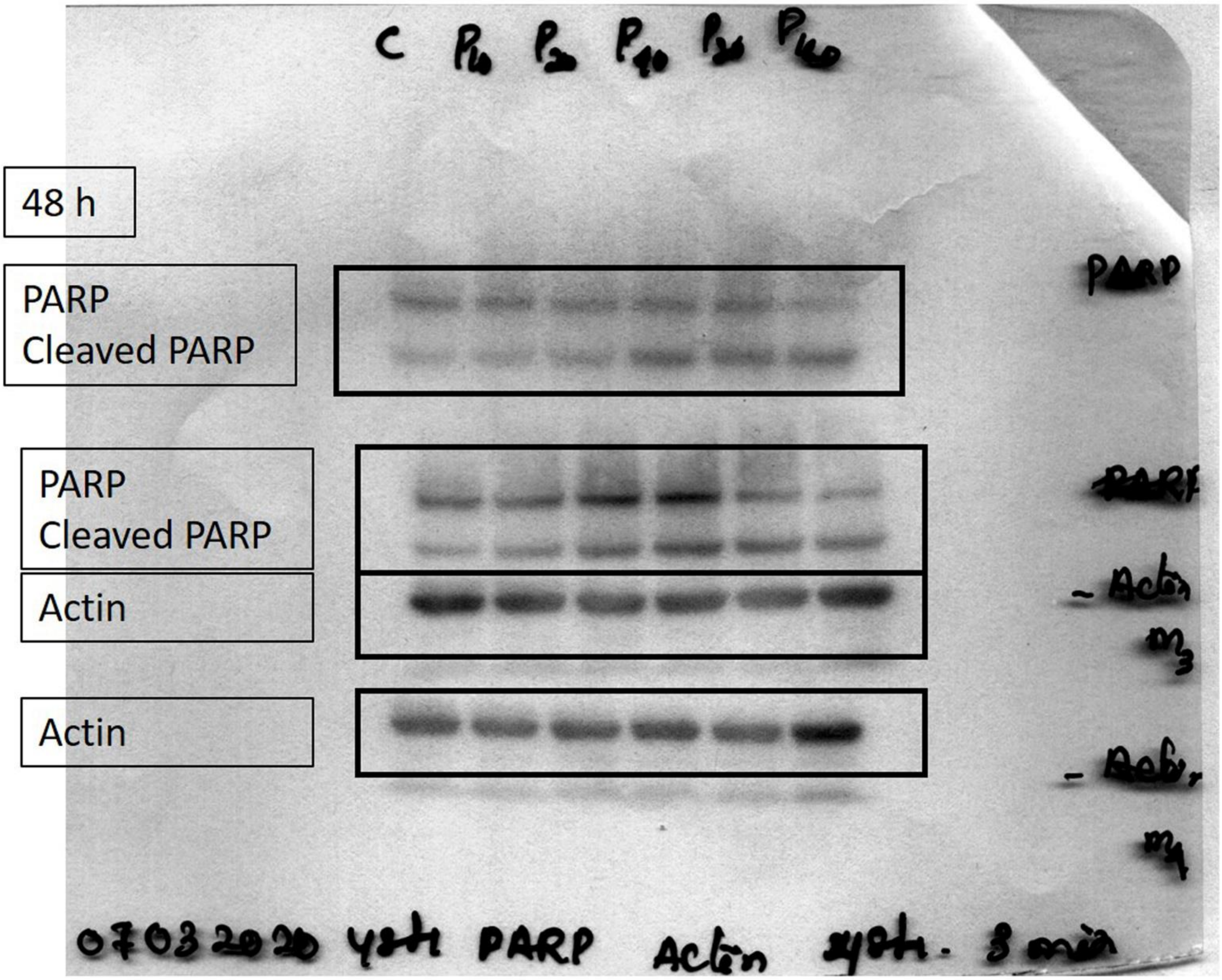

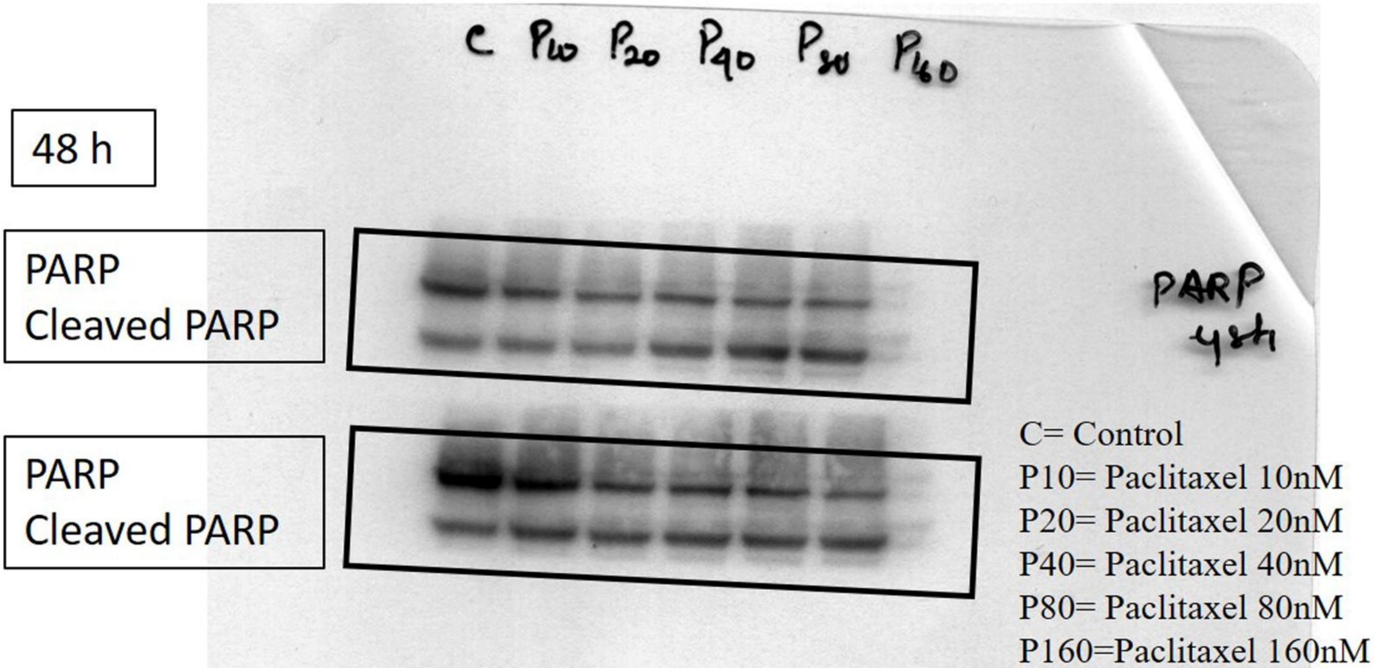

C= Control
P10= Paclitaxel 10nM
P20= Paclitaxel 20nM
P40= Paclitaxel 40nM
P80= Paclitaxel 80nM
P160=Paclitaxel 160nM

Fig. 2e

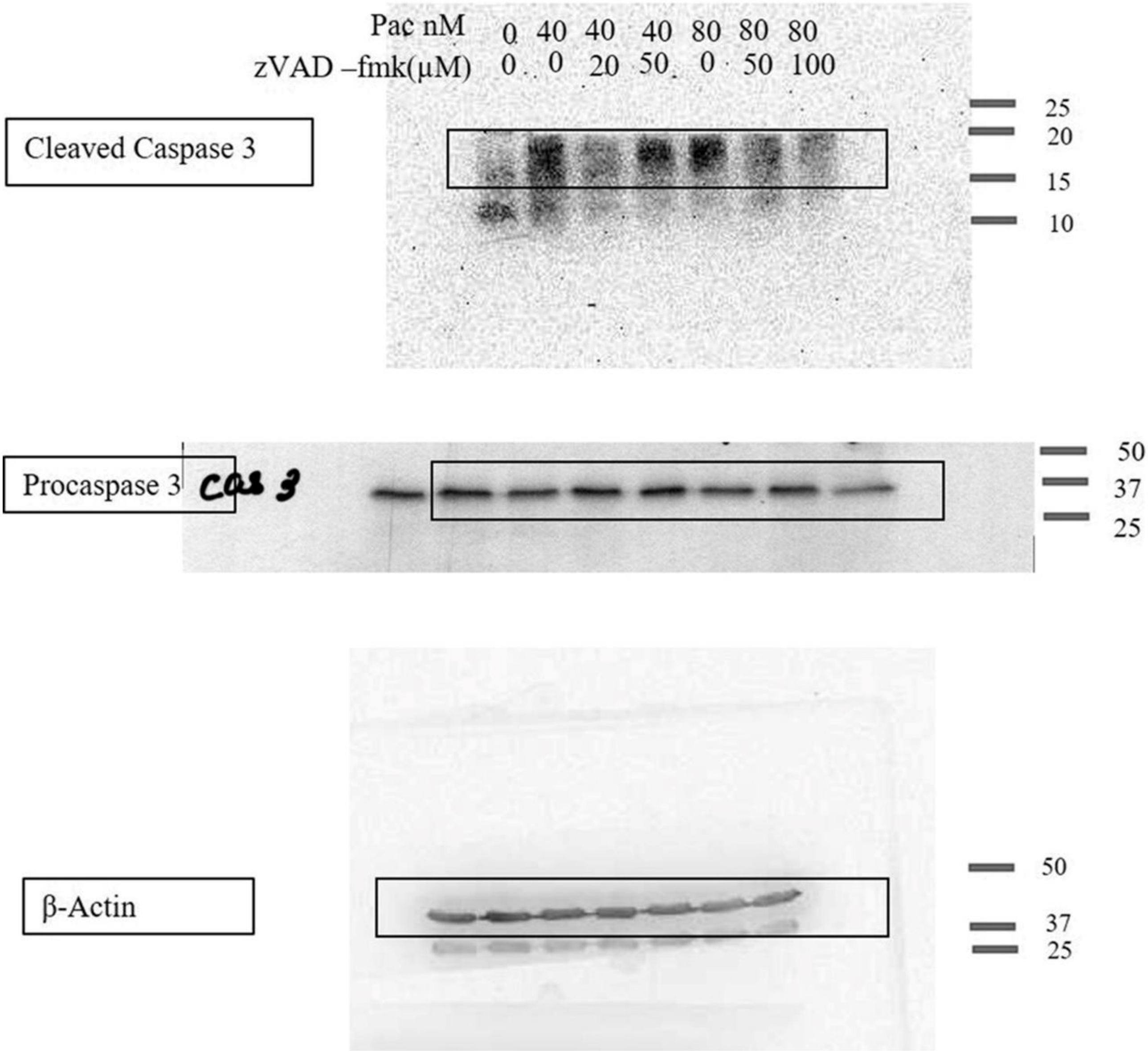
Pac nM 0 40 40 40 80 80 80
zVAD –fmk(μM) 0 0 20 50 0 50 100
Cleaved Caspase 3
25
20
15
10
Procaspase 3
cas 3
50
37
25
β-Actin
50
37
25

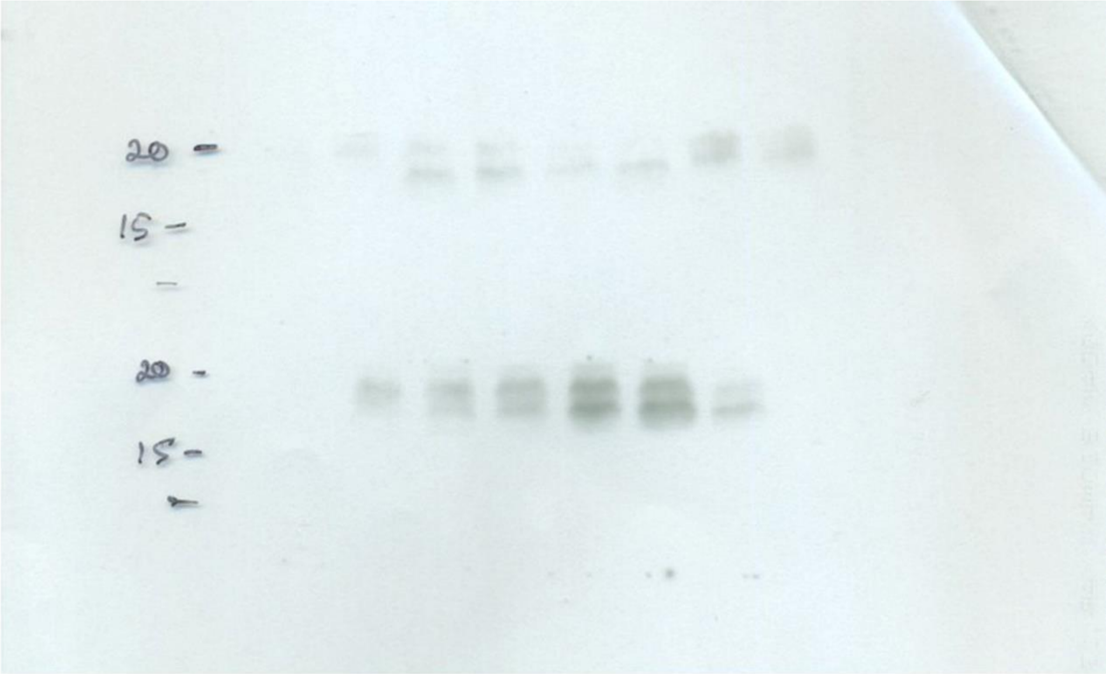
20
15
20
15

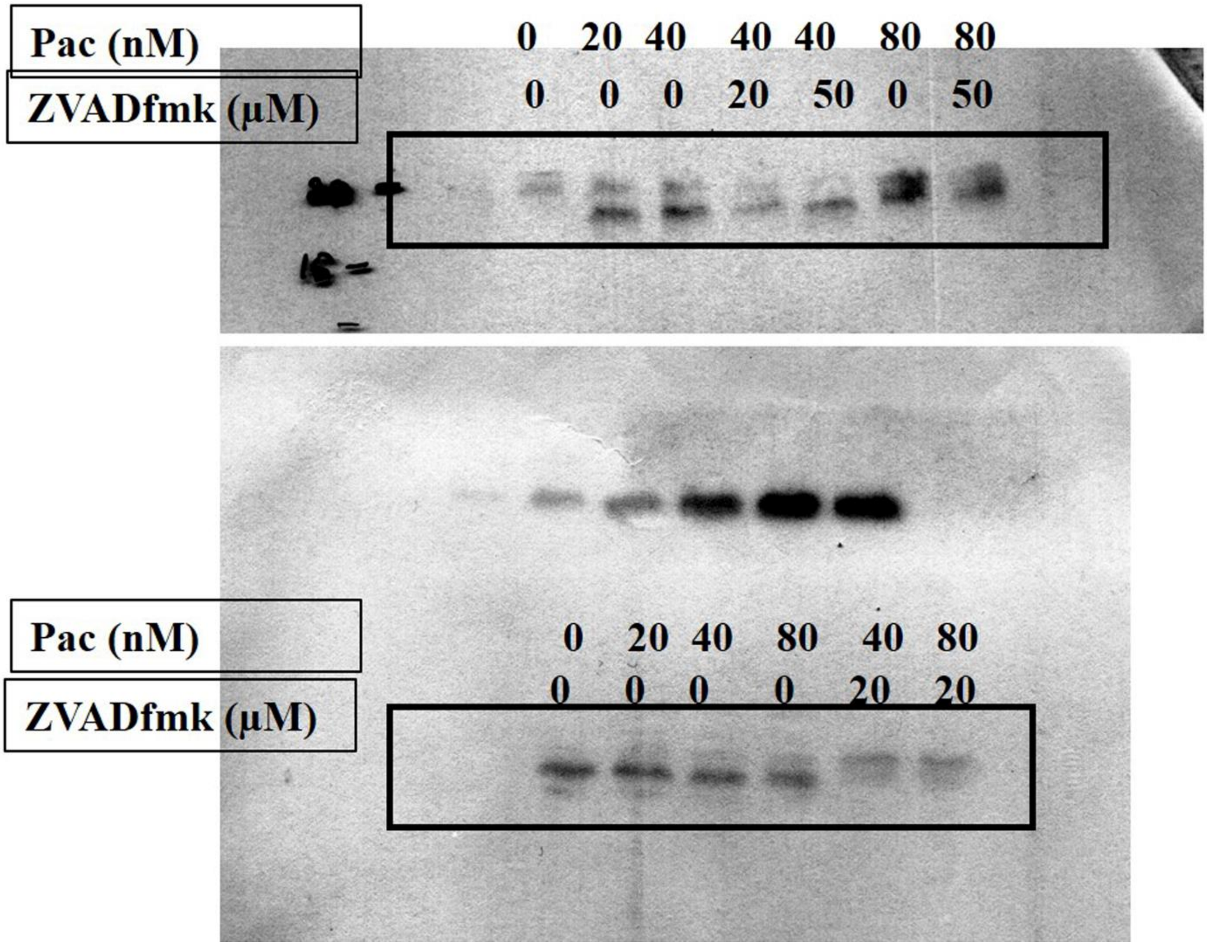
Pac (nM)
0 20 40 40 40 80 80
ZVADfmk (µM)
0 0 0 20 50 0 50
Pac (nM)
0 20 40 80 40 80
ZVADfmk (µM)
0 0 0 0 20 20

Procaspase-3

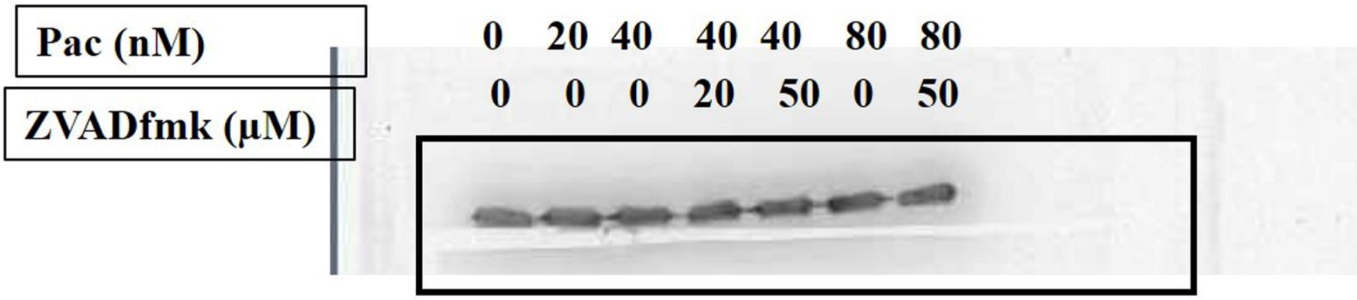

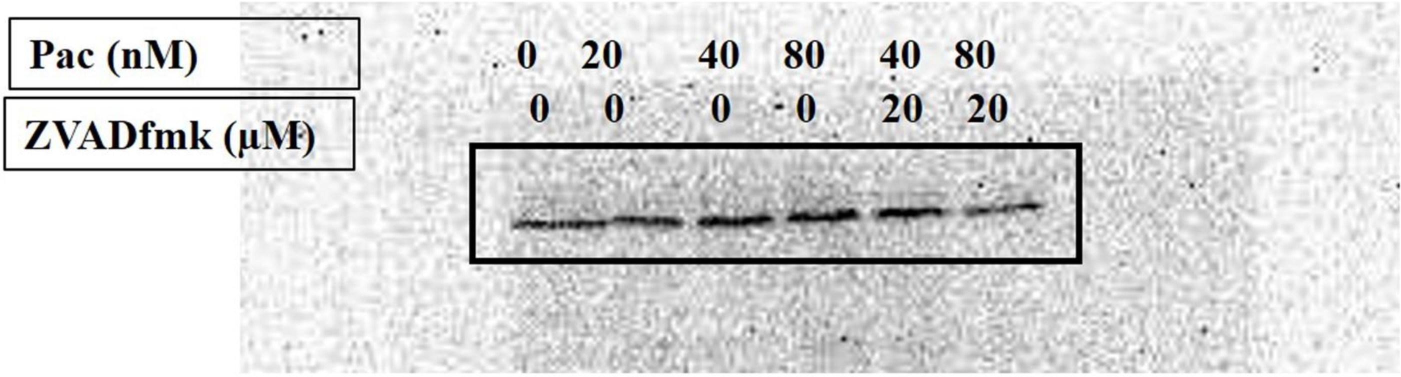

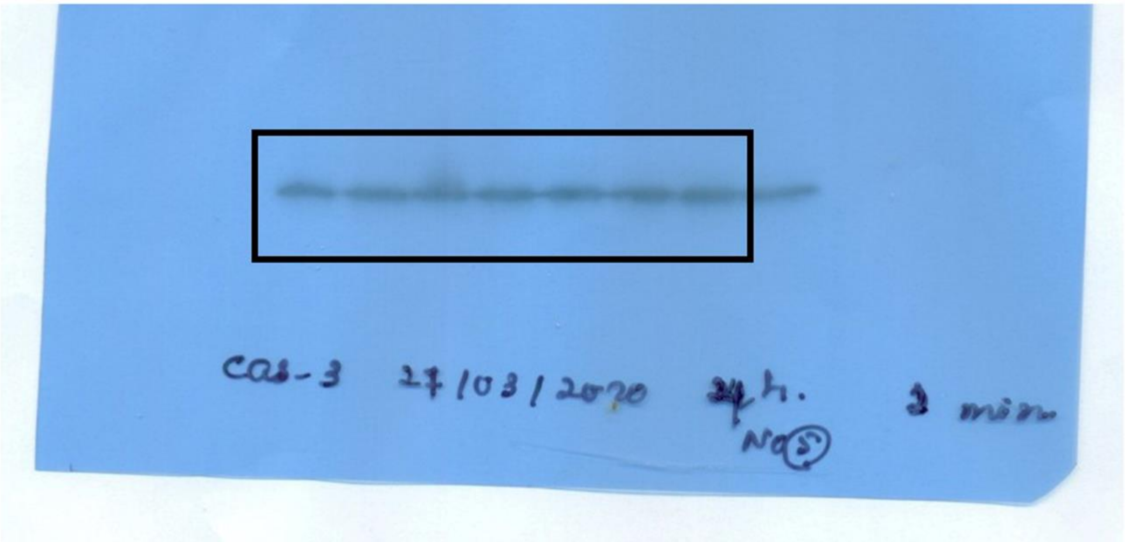

Supporting data

Fig. BB Photographs of the replicated full-length blots described in Figure 3

Fig. 3b

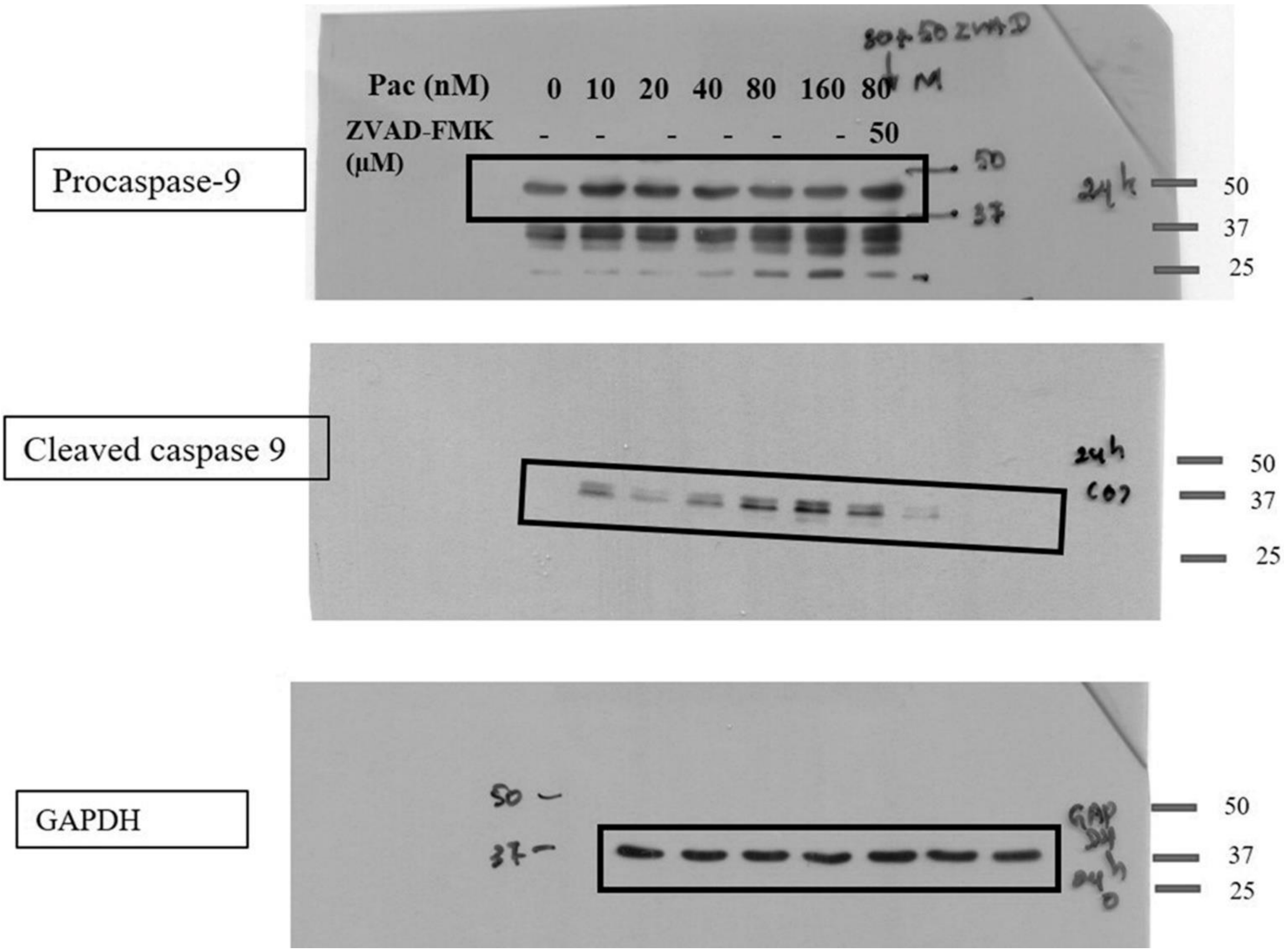

Cleaved cas 9

24 h

Cleaved caspase 9

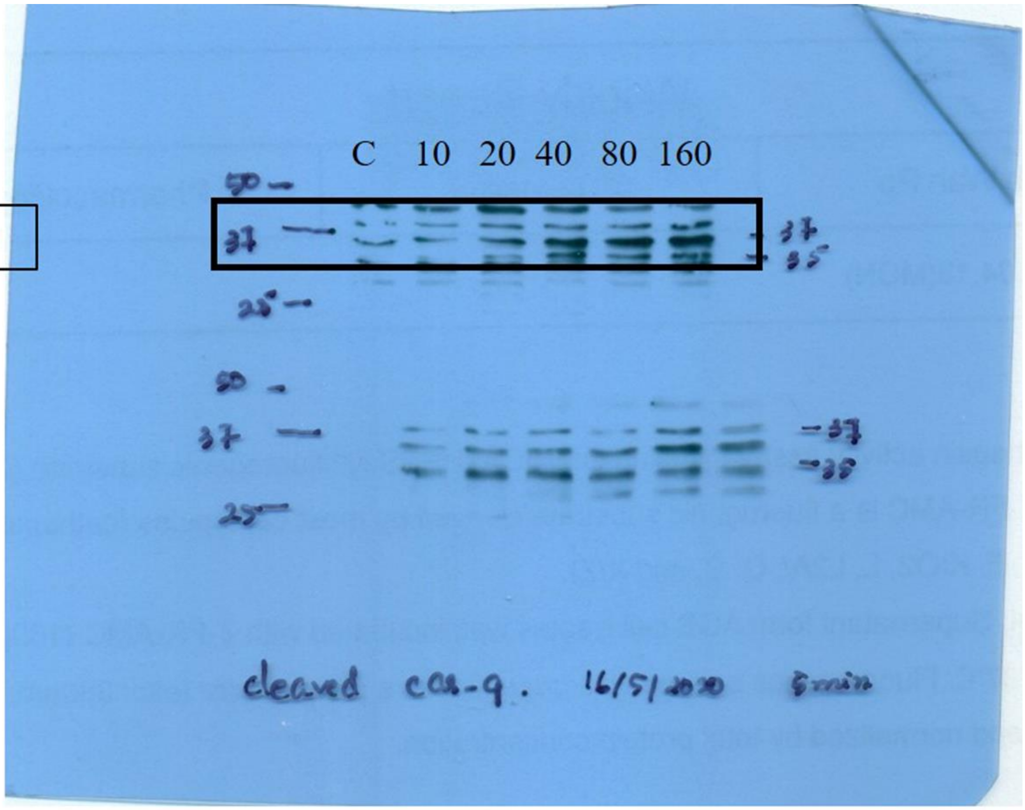

24 h

Cleaved caspase 9

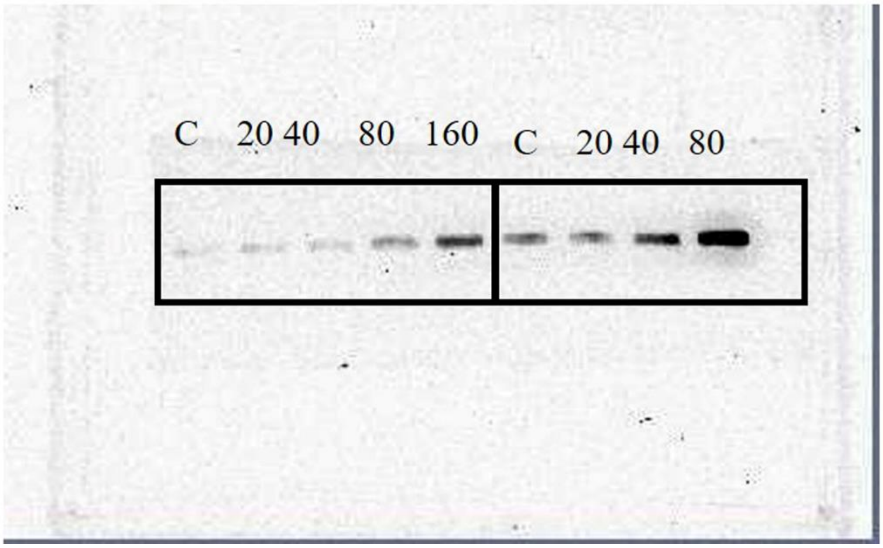

Fig. 3c

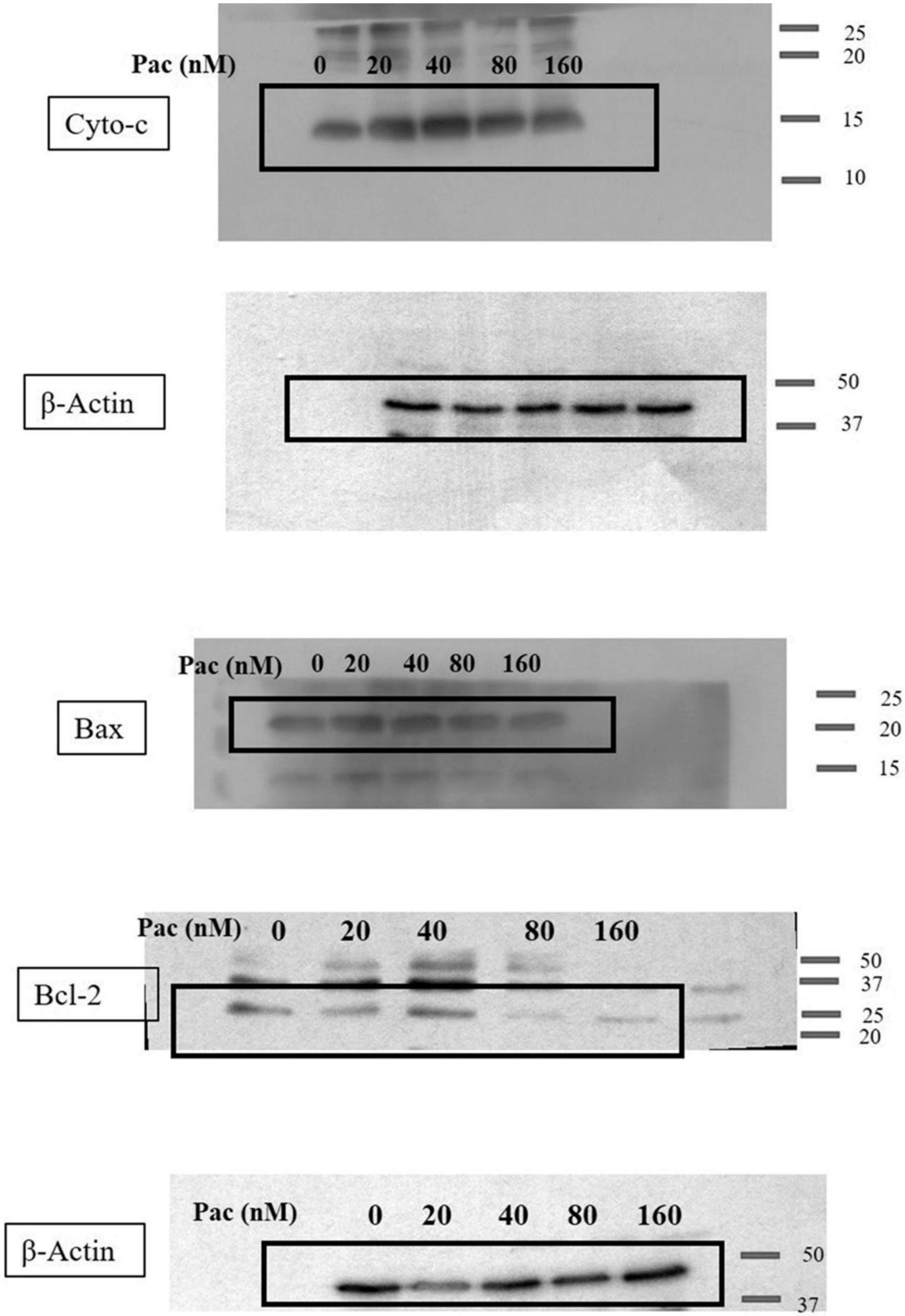
Pac (nM)
0 20 40 80 160
Cyto-c
25
20
15
10
β-Actin
50
37
Pac (nM)
0 20 40 80 160
Bax
25
20
15
Pac (nM)
0 20 40 80 160
Bcl-2
50
37
25
20
Pac (nM)
0 20 40 80 160
β-Actin
50
37

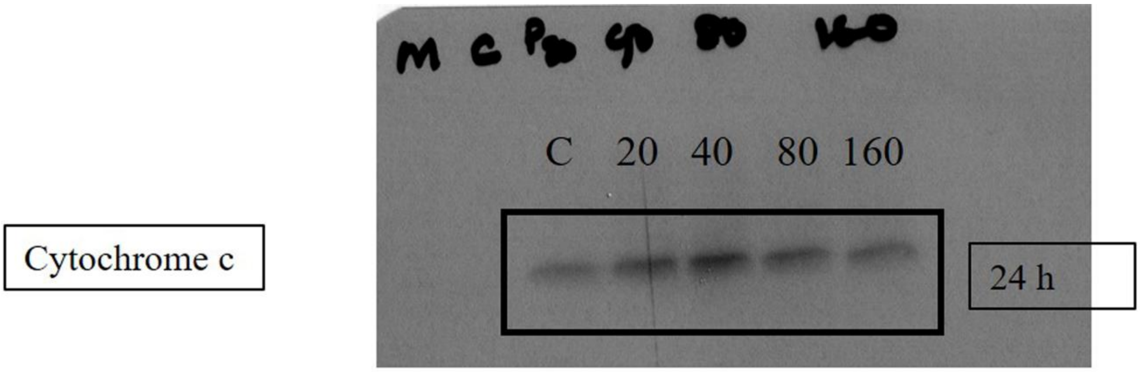
C 20 40 80 160
Cytochrome c
24 h

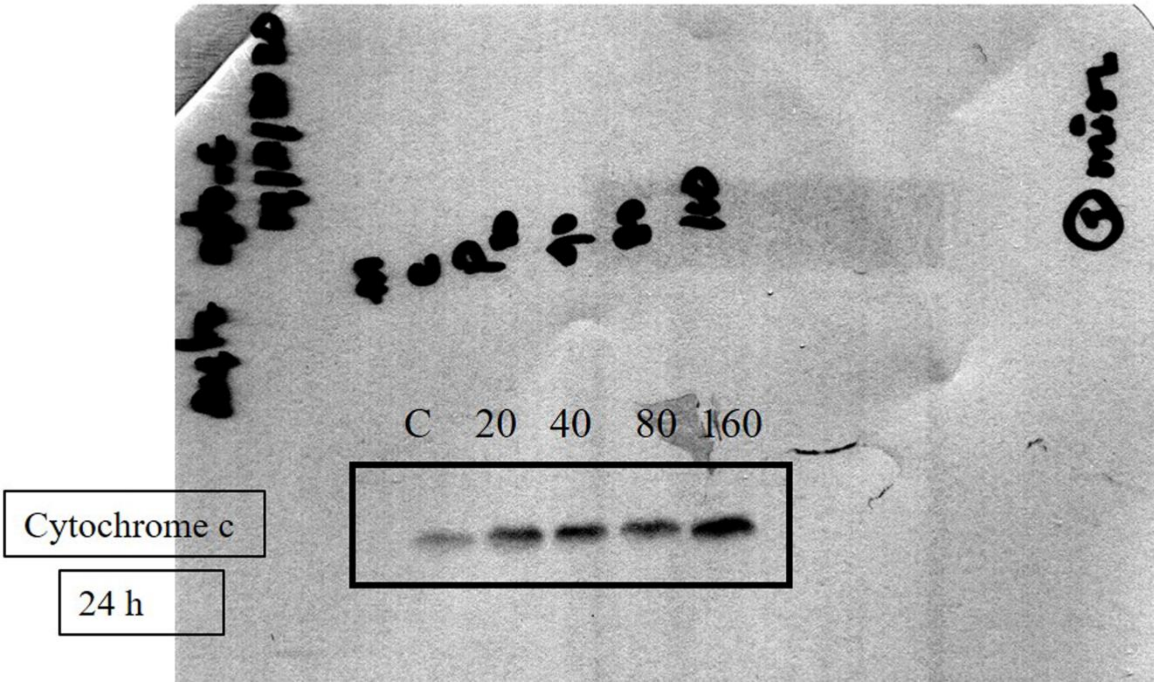
C 20 40 80 160
Cytochrome c
24 h

Bax

24 h

Bax

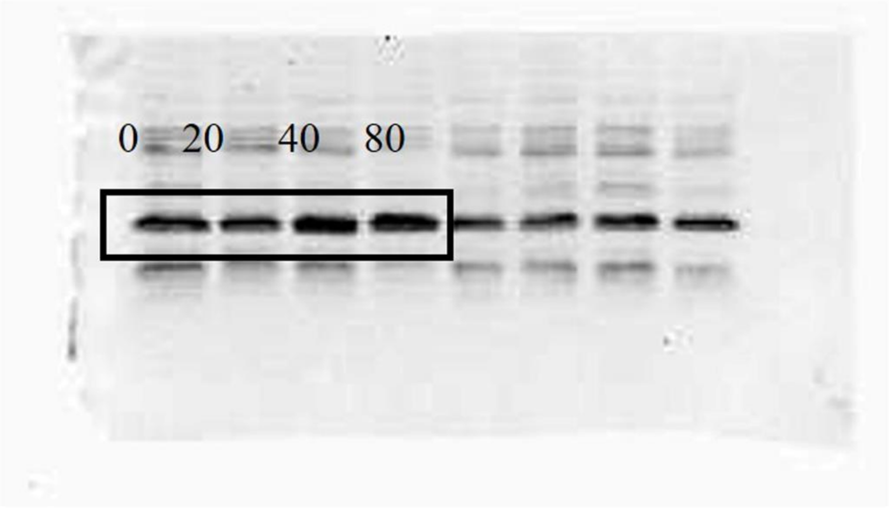

24 h

Bax

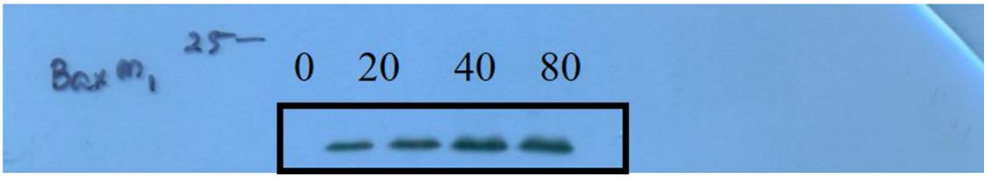

24 h

Bax

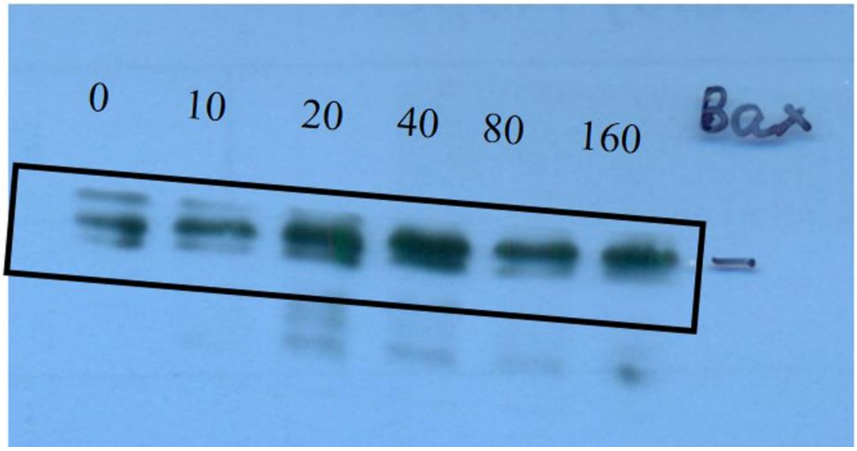

Bax

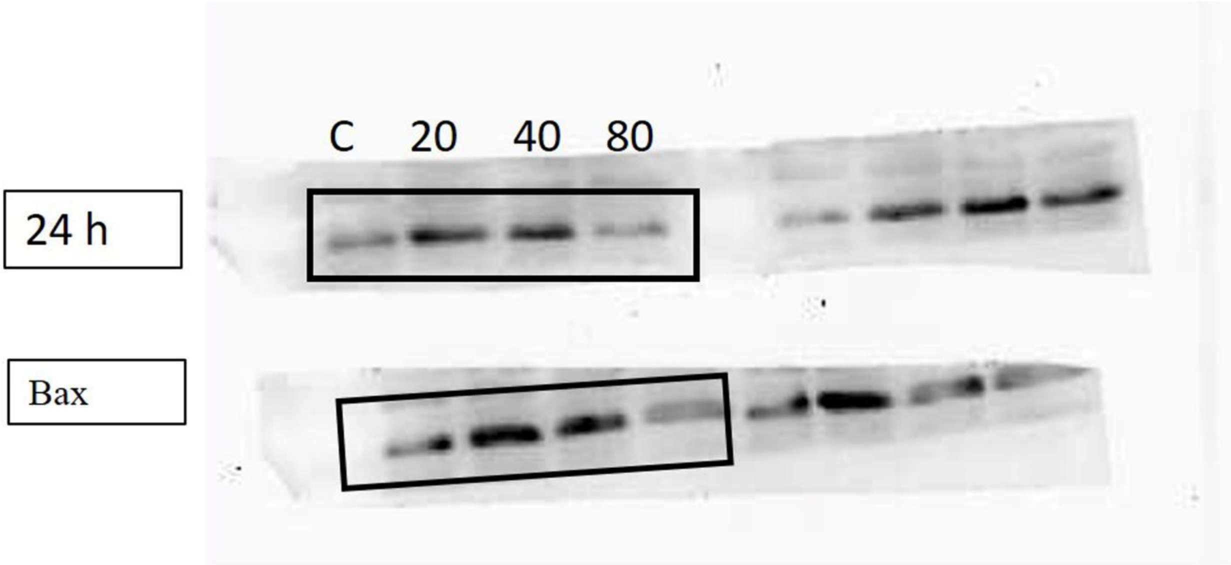
24 h
Bax
C 20 40 80

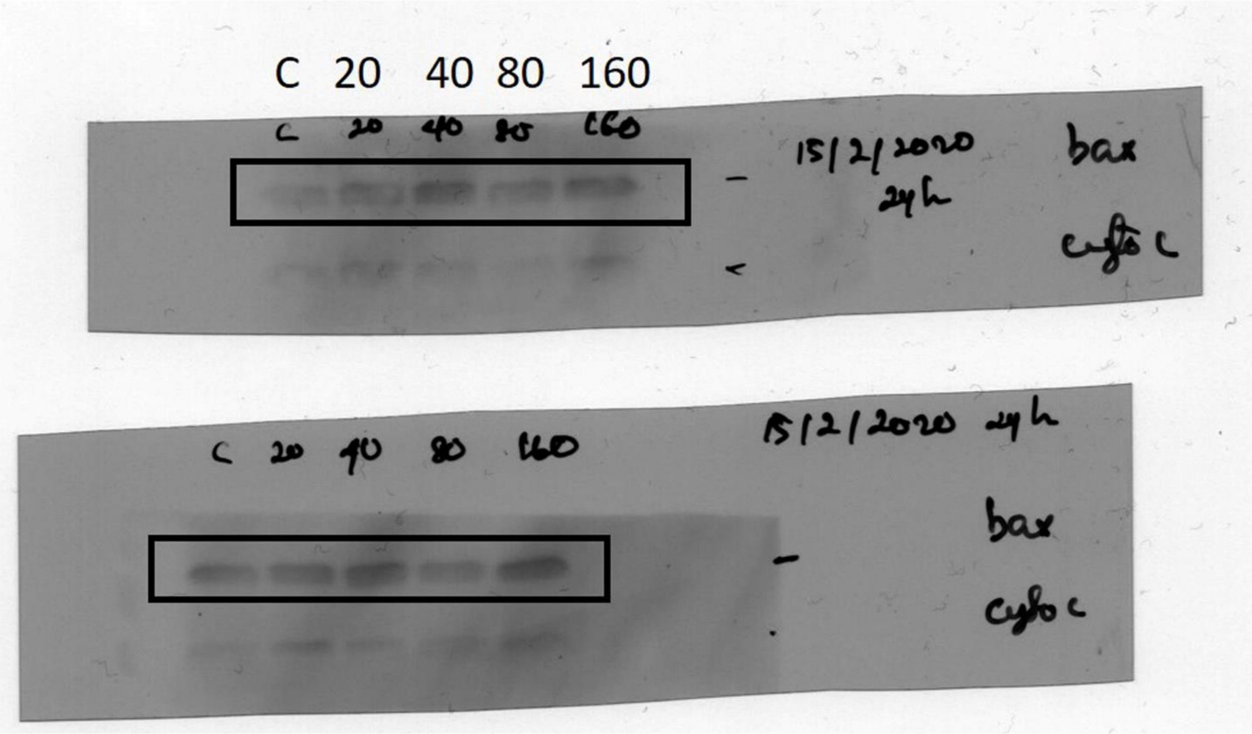
C 20 40 80 160
c 20 40 80 160
15/2/2020
24h
bax
cyto c
c 20 40 80 160
15/2/2020 24h
bax
cyto c

Bcl-2

24 h

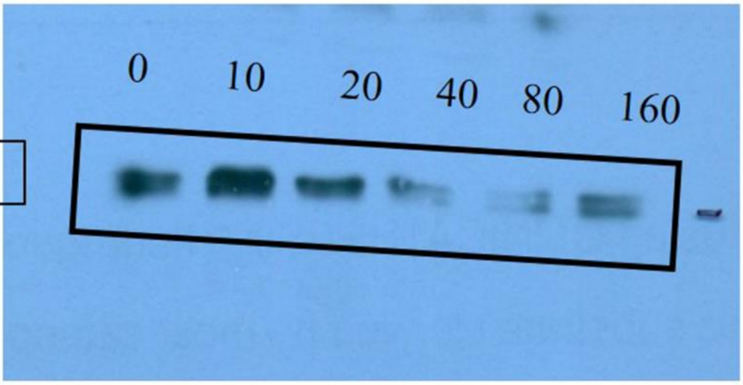

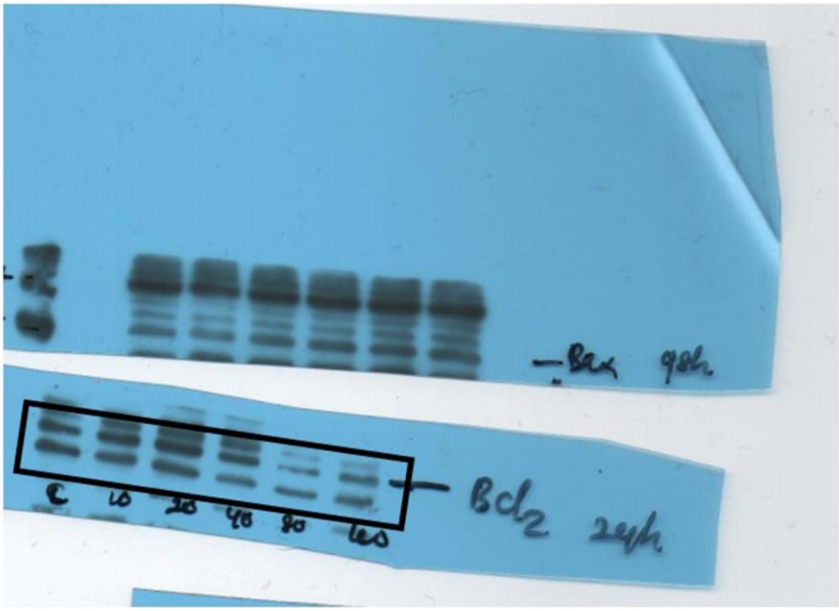

Bcl-2

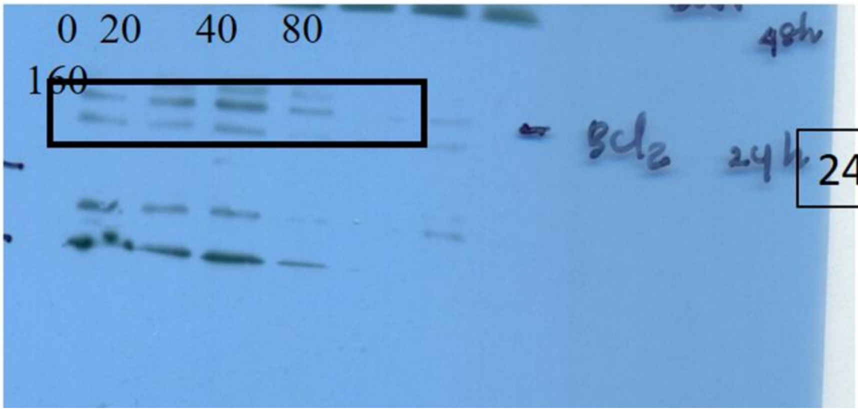

24 h

Bcl-2

24 h

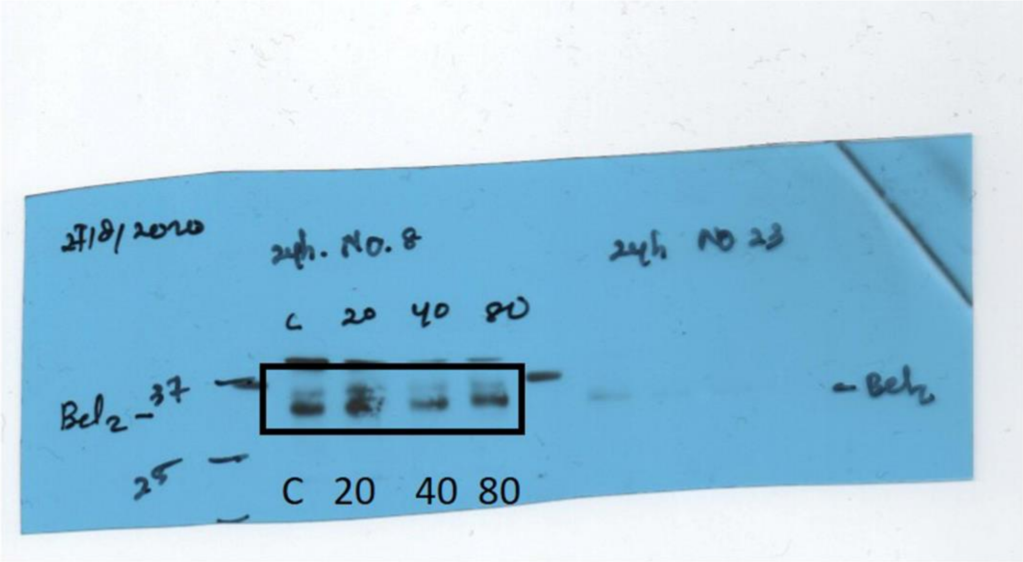
27/8/2020
24h. No. 8
c 20 40 80
24h NO 23
Bcl2 - 37
- Bcl2
25
C 20 40 80

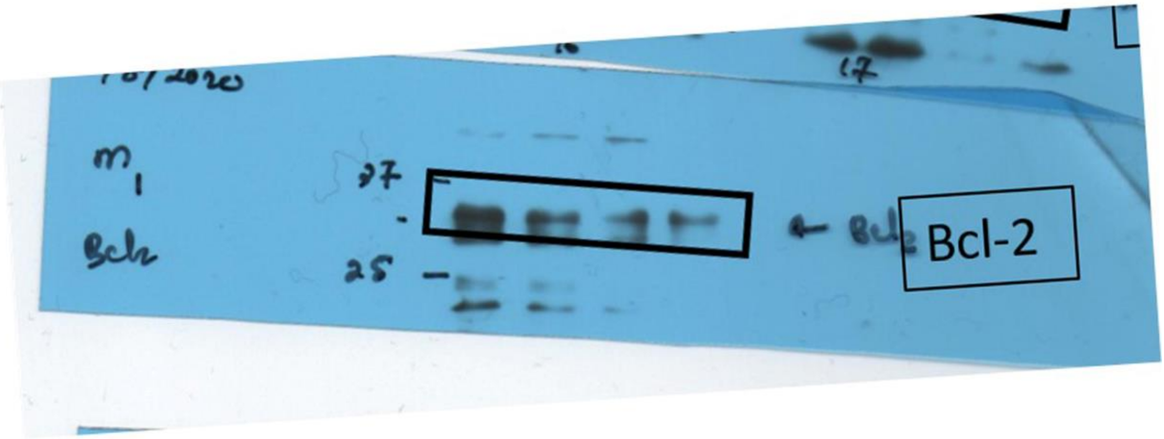
m1
Bcl2
37
25
← Bcl2
17
Bcl-2

Fig.3d

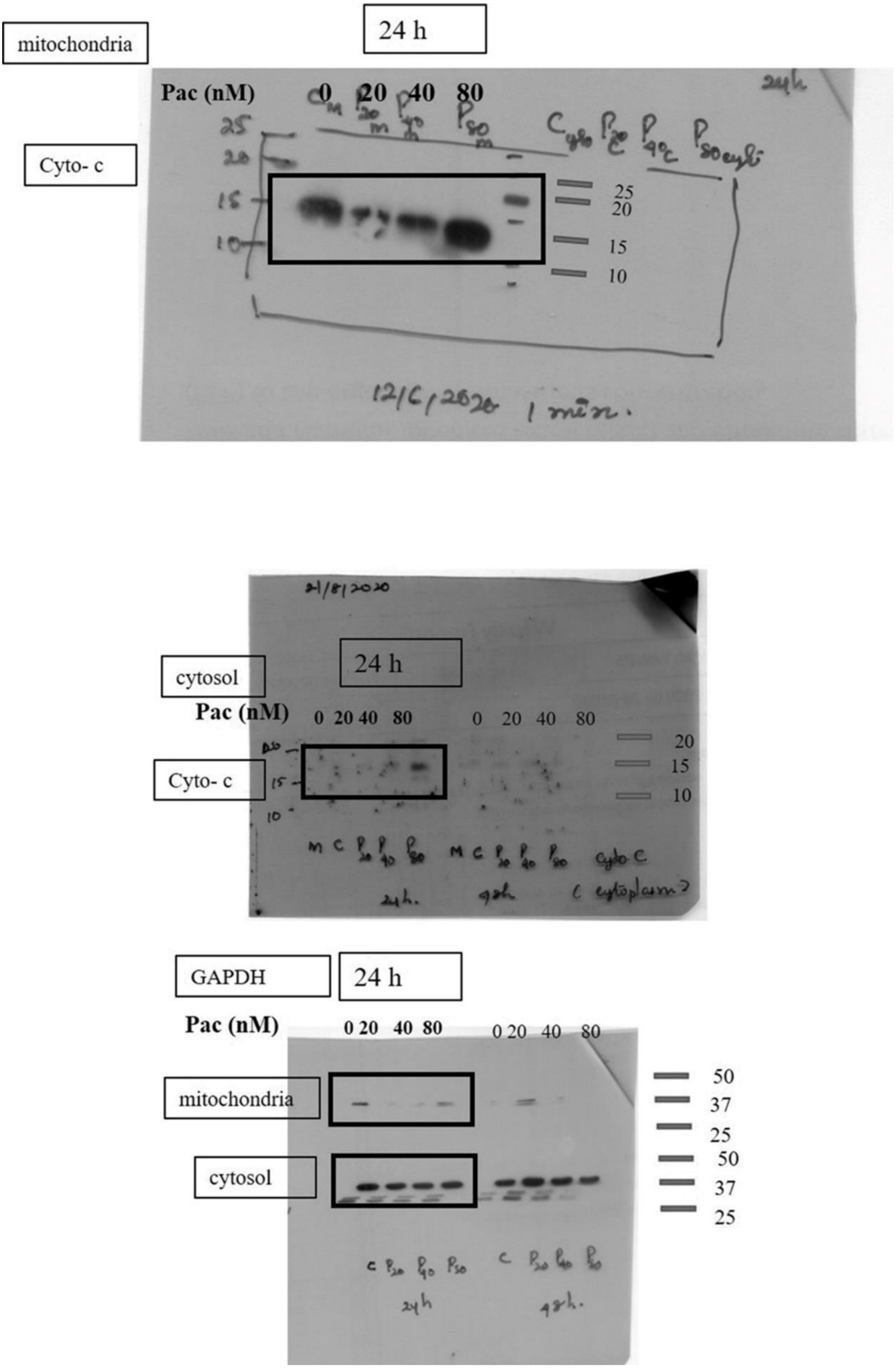

mitochondria
24 h
Pac (nM)
0 20 40 80
Cyto- c
25
20
15
10
cytosol
24 h
Pac (nM)
0 20 40 80
0 20 40 80
Cyto- c
20
15
10
GAPDH
24 h
Pac (nM)
0 20 40 80
0 20 40 80
mitochondria
cytosol
50
37
25
50
37
25

Fig. 3f

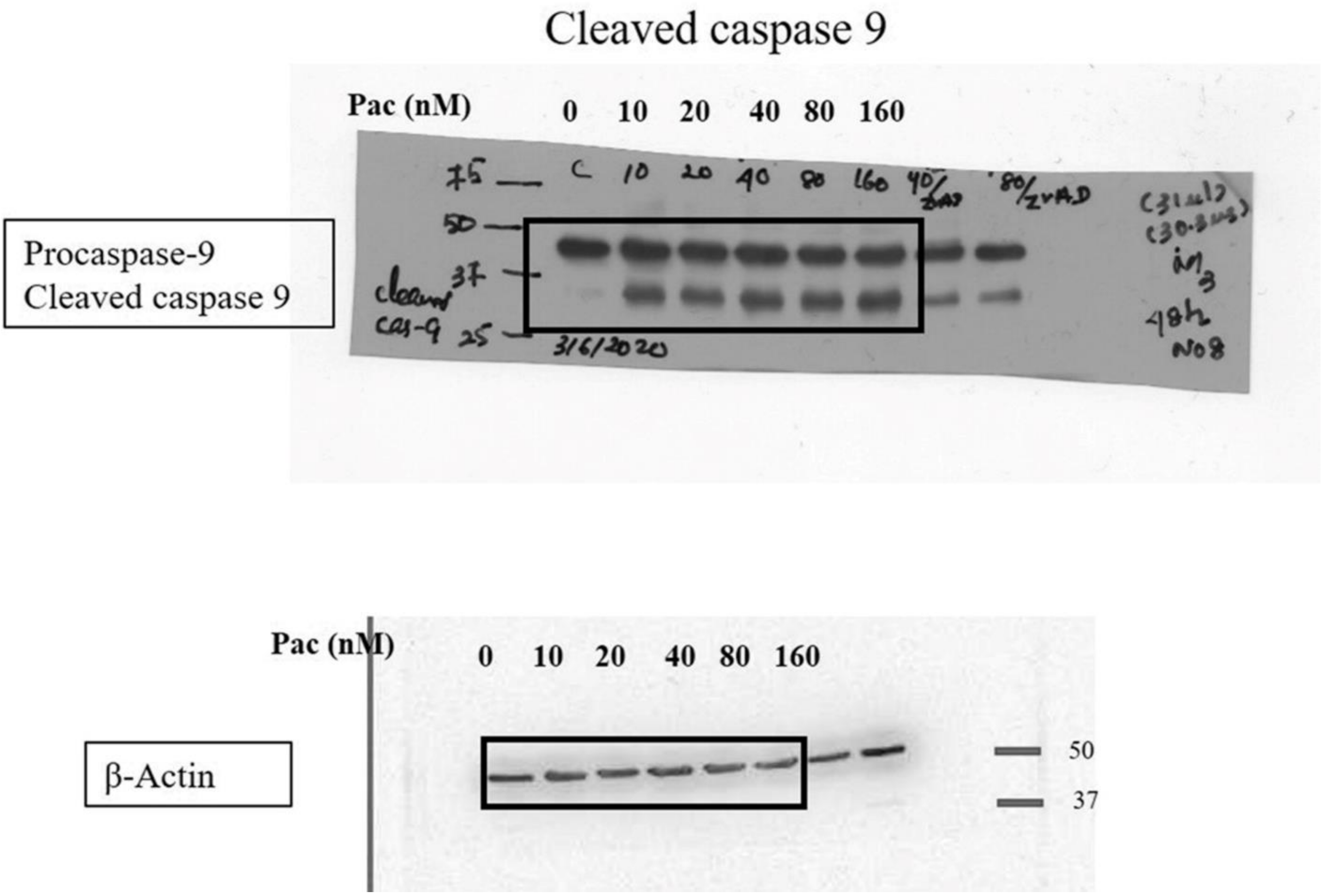
Cleaved caspase 9
Pac (nM)
0 10 20 40 80 160
Procaspase-9
Cleaved caspase 9
Pac (nM)
0 10 20 40 80 160
β-Actin
50
37

Cleaved caspase 9

48 h

Procaspase-9
Cleaved caspase 9

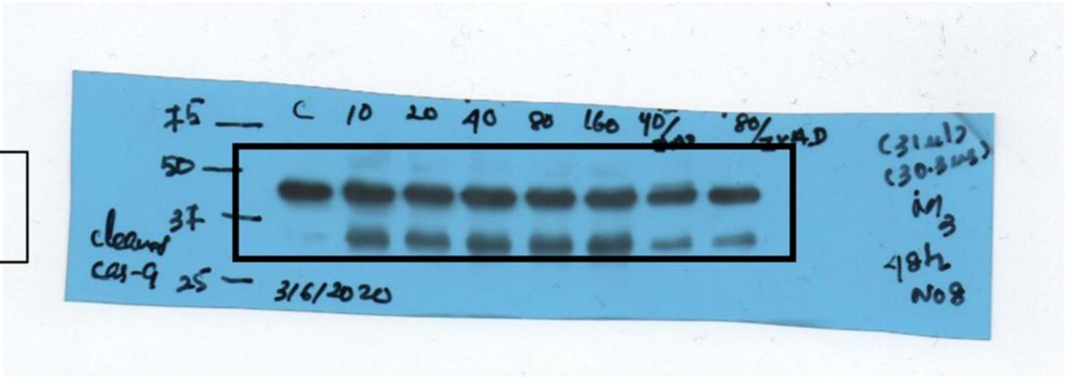

48 h

Cleaved caspase 9

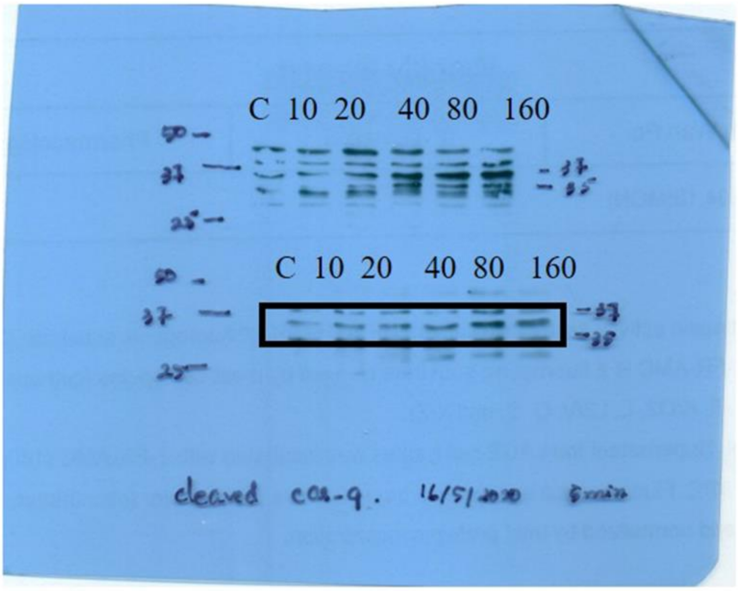

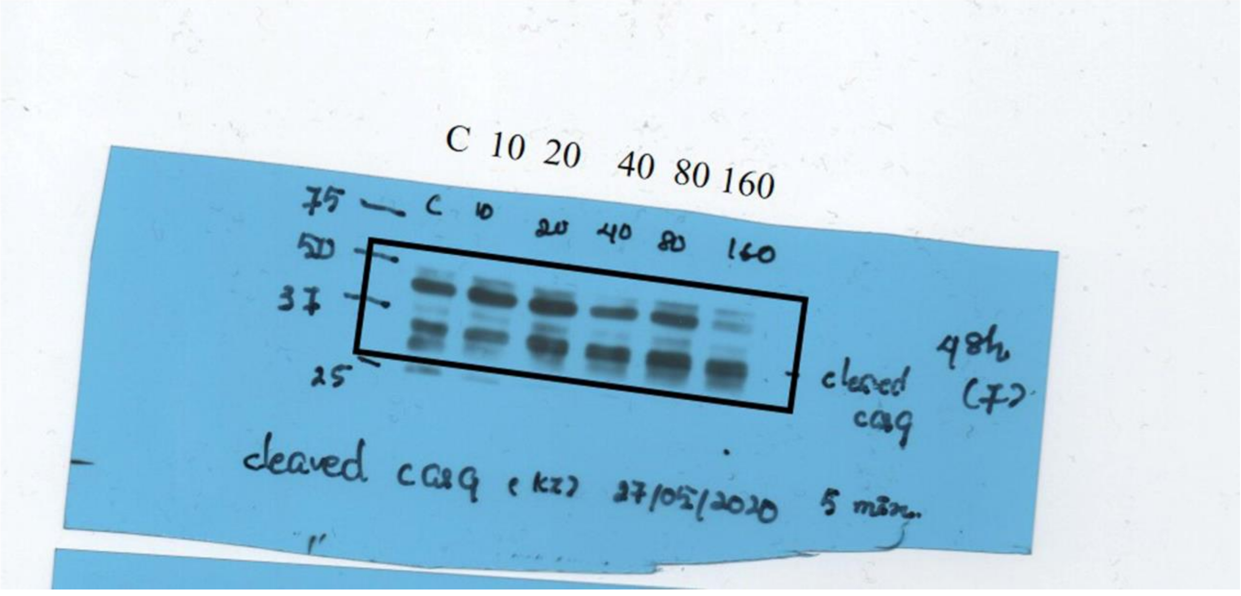

Fig. 3g

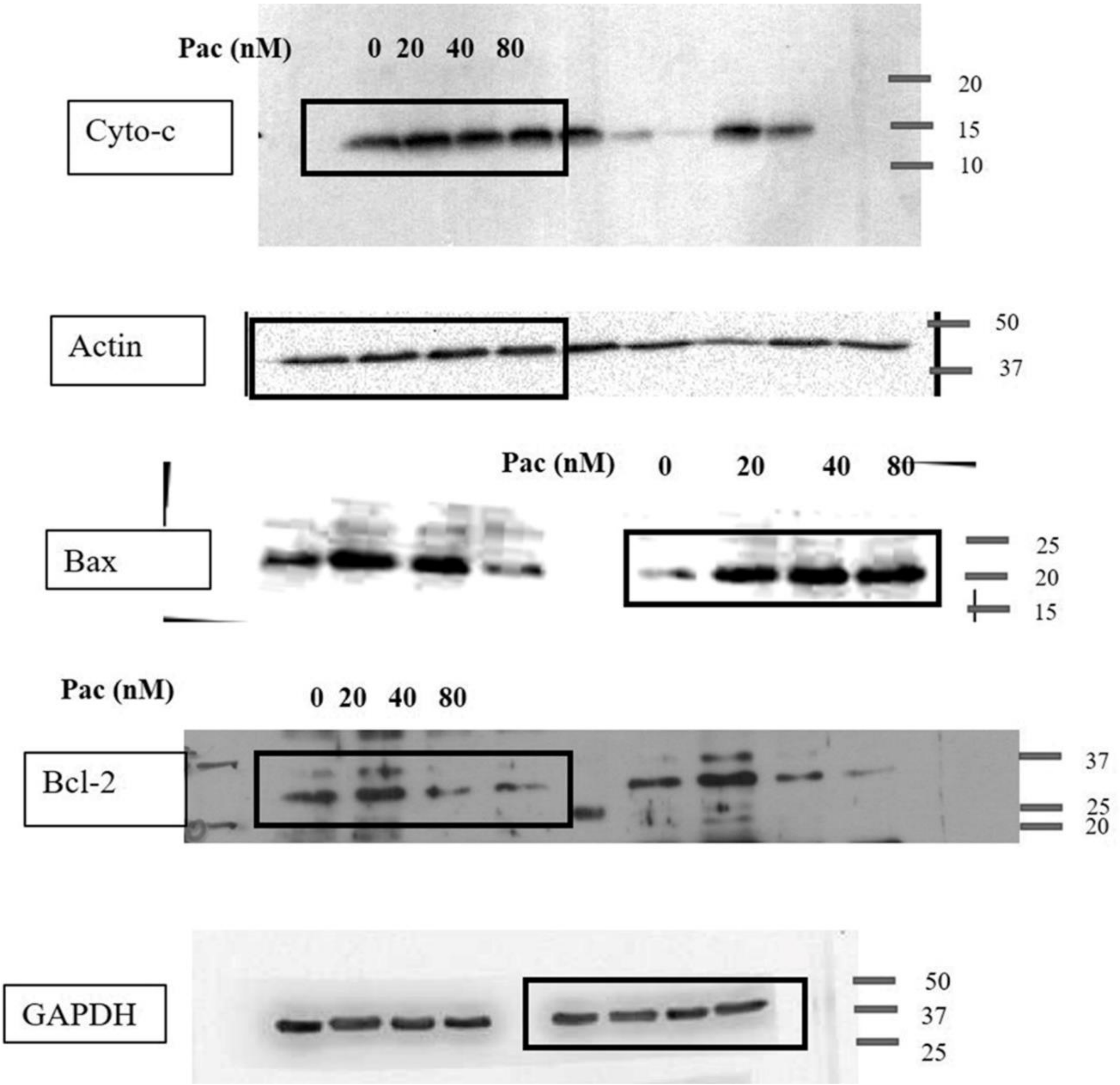

Pac (nM)
0 20 40 80
Cyto-c
20
15
10
Actin
50
37
Pac (nM)
0 20 40 80
Bax
25
20
15
Pac (nM)
0 20 40 80
Bcl-2
37
25
20
GAPDH
50
37
25

Cytochrome c

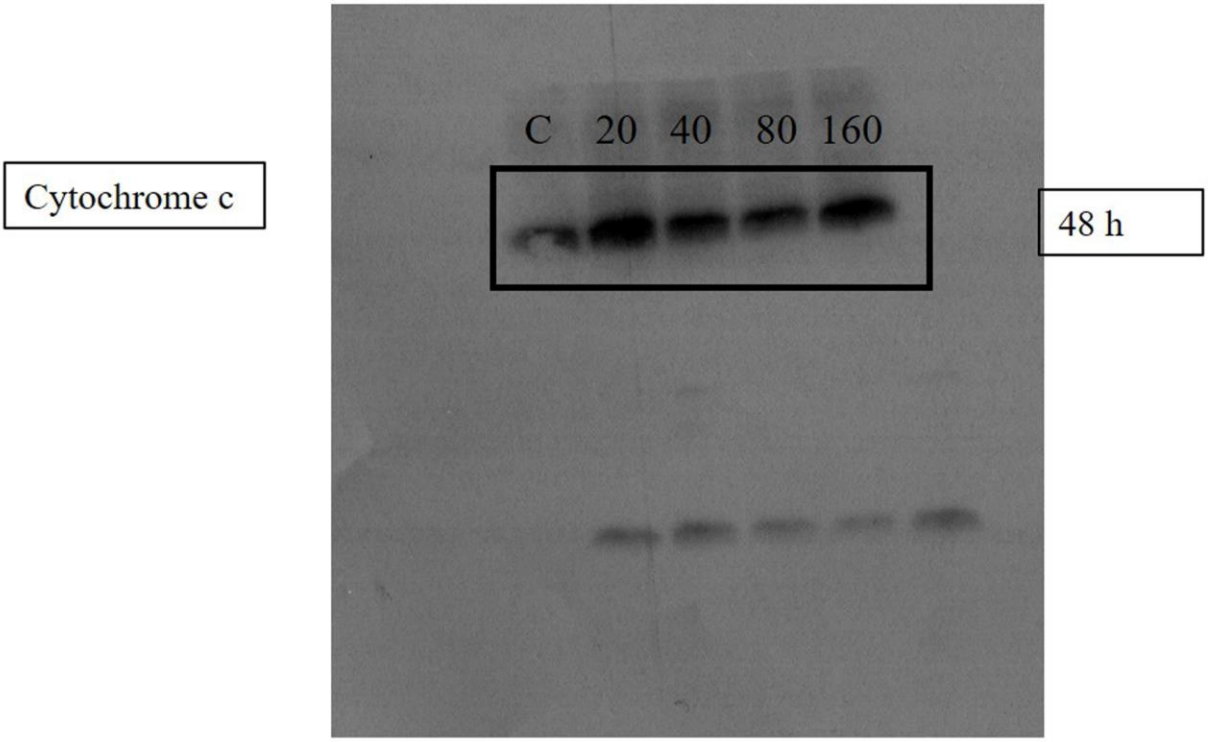

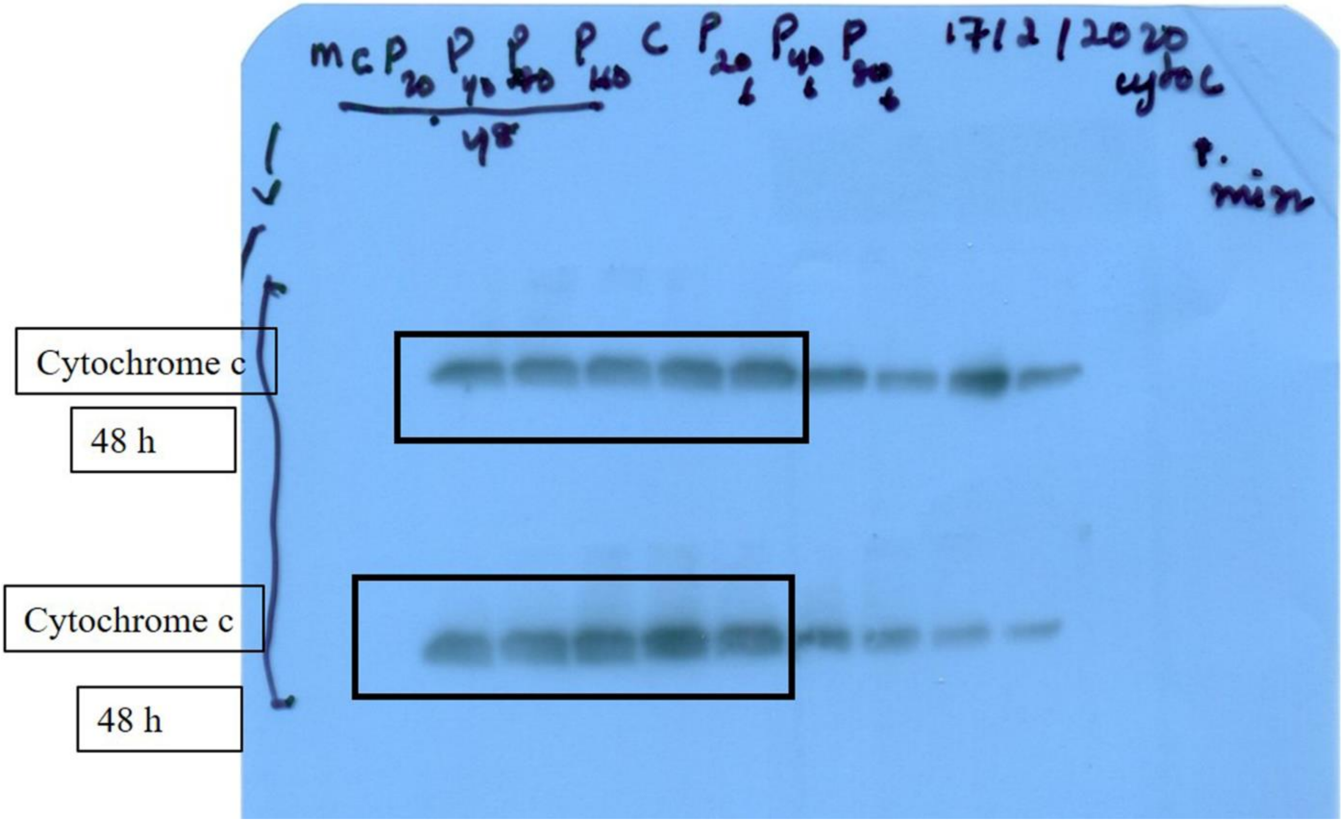

Bax

48 h

Bax

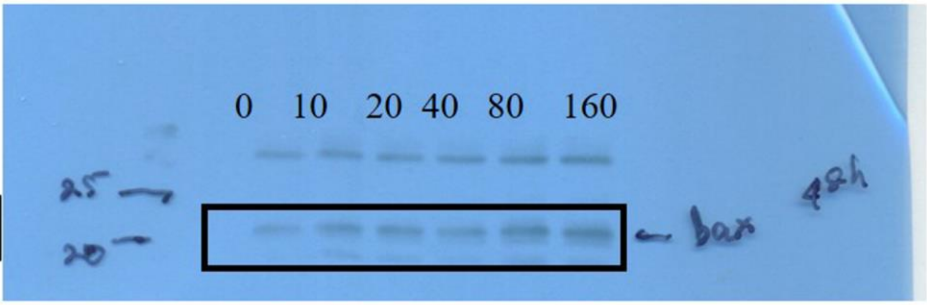

Bax

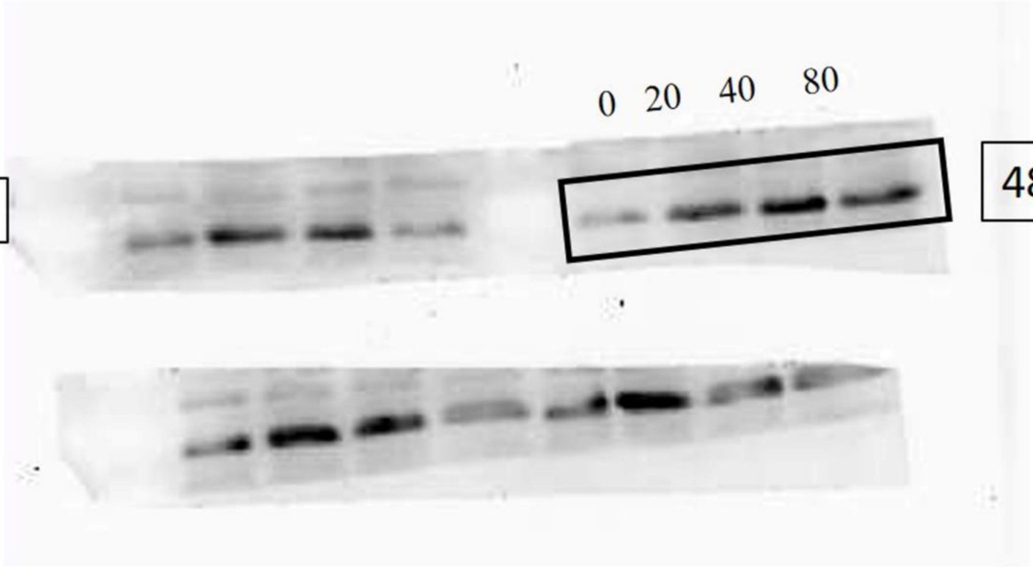

48 h

48 h

Bax

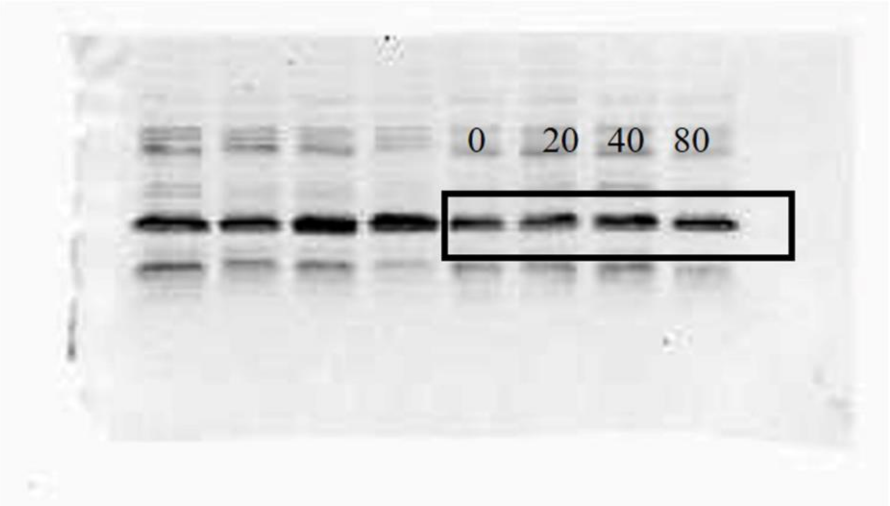

Bax
48 h

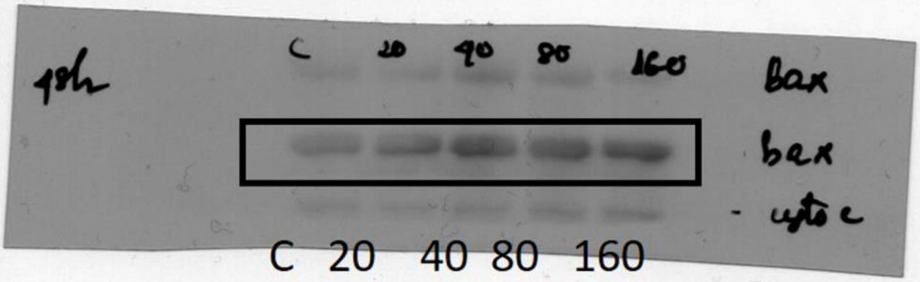

Bax
48 h

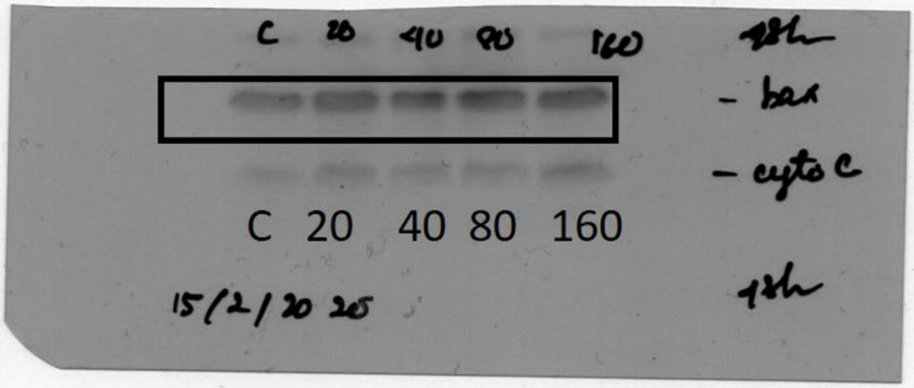

Bcl-2

Bcl-2

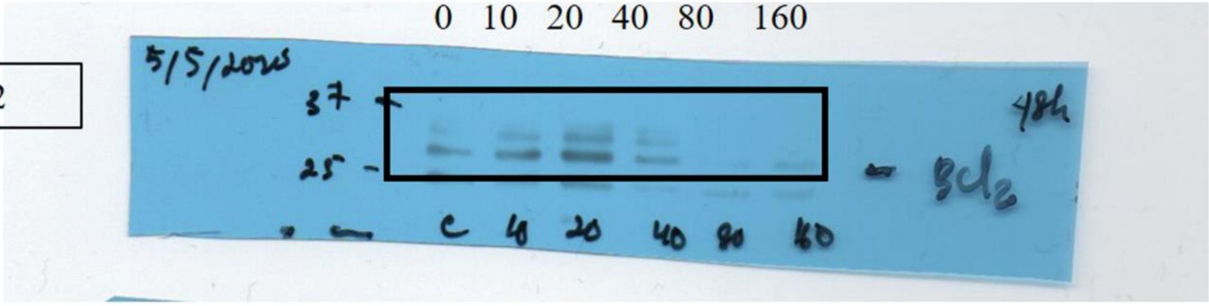

Bcl-2

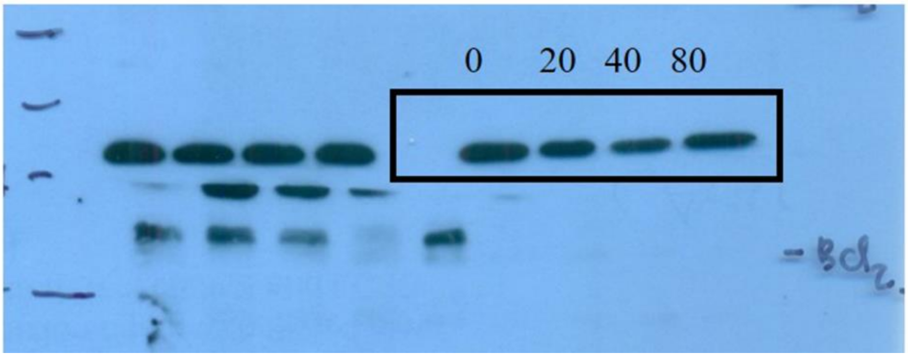

48 h

Fig. 3h

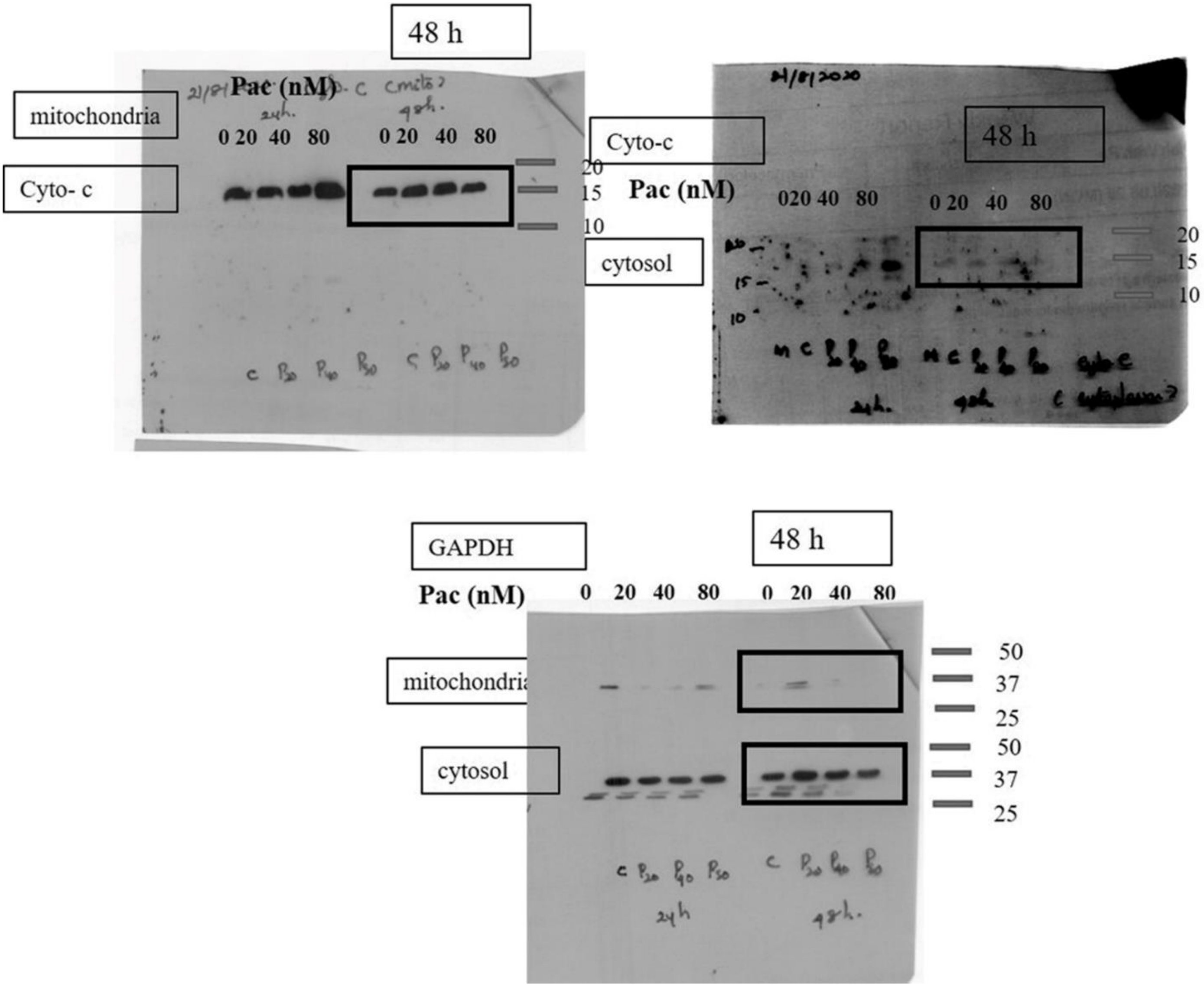
48 h
Pac (nM)
mitochondria
Cyto- c
0 20 40 80
0 20 40 80
20
15
10
Cyto-c
48 h
Pac (nM)
020 40 80
0 20 40 80
cytosol
20
15
10
GAPDH
48 h
Pac (nM)
0 20 40 80
0 20 40 80
mitochondria
cytosol
50
37
25
50
37
25

Supporting data

Fig. DD Photographs of the replicated full-length blots utilized in Figure 4

Fig. 4a

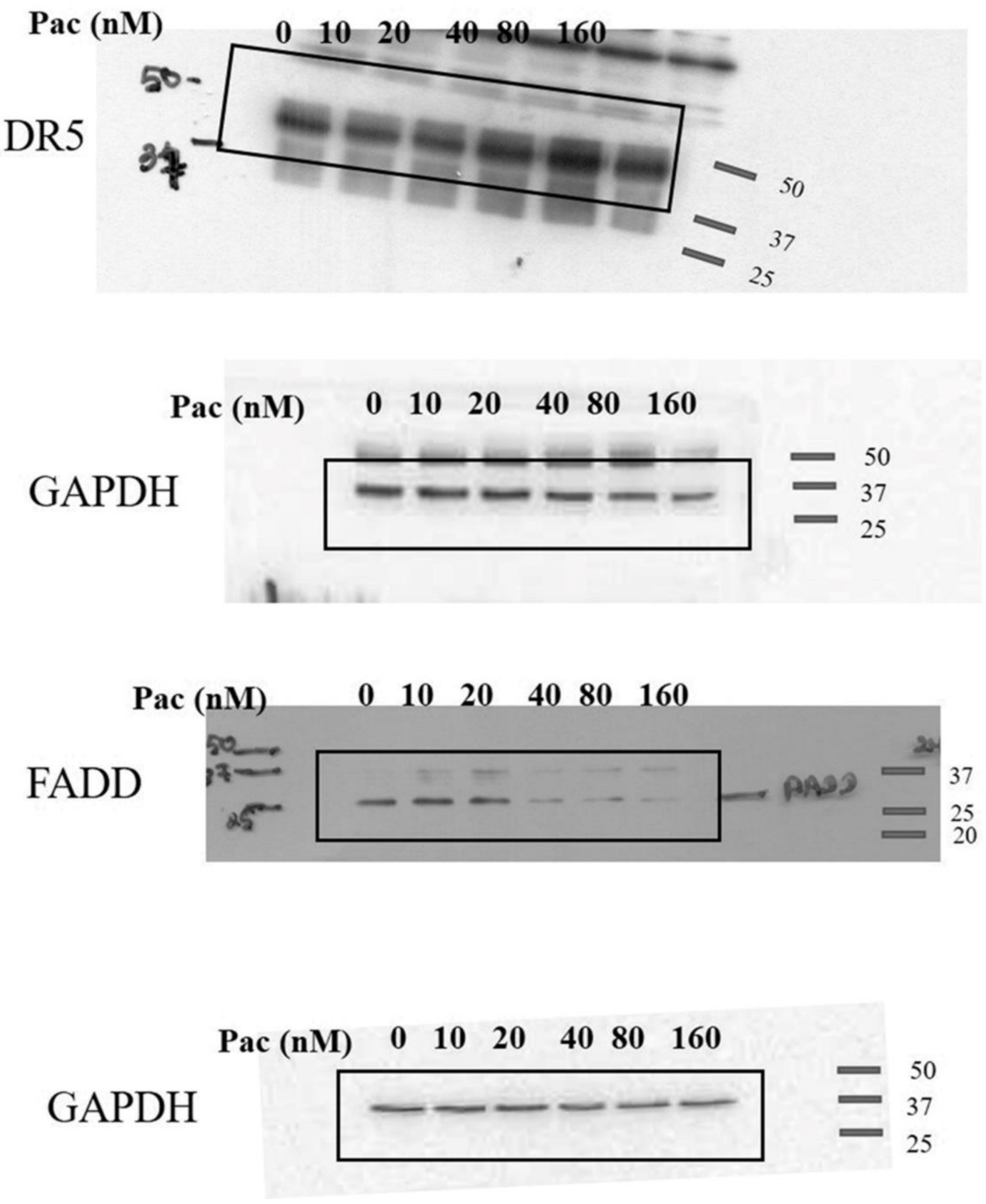

DR5

DR5

24 h

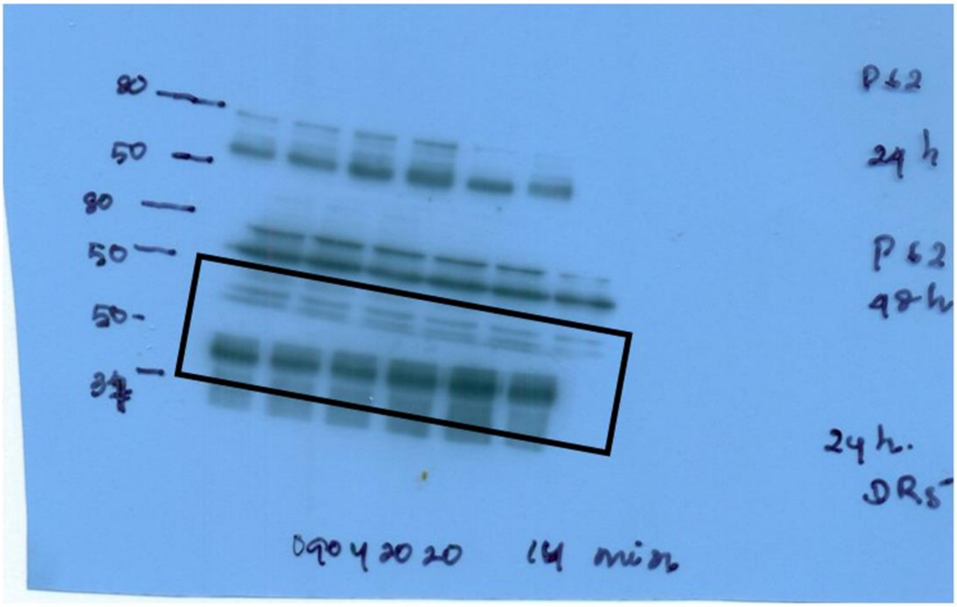

DR5

24 h

48h

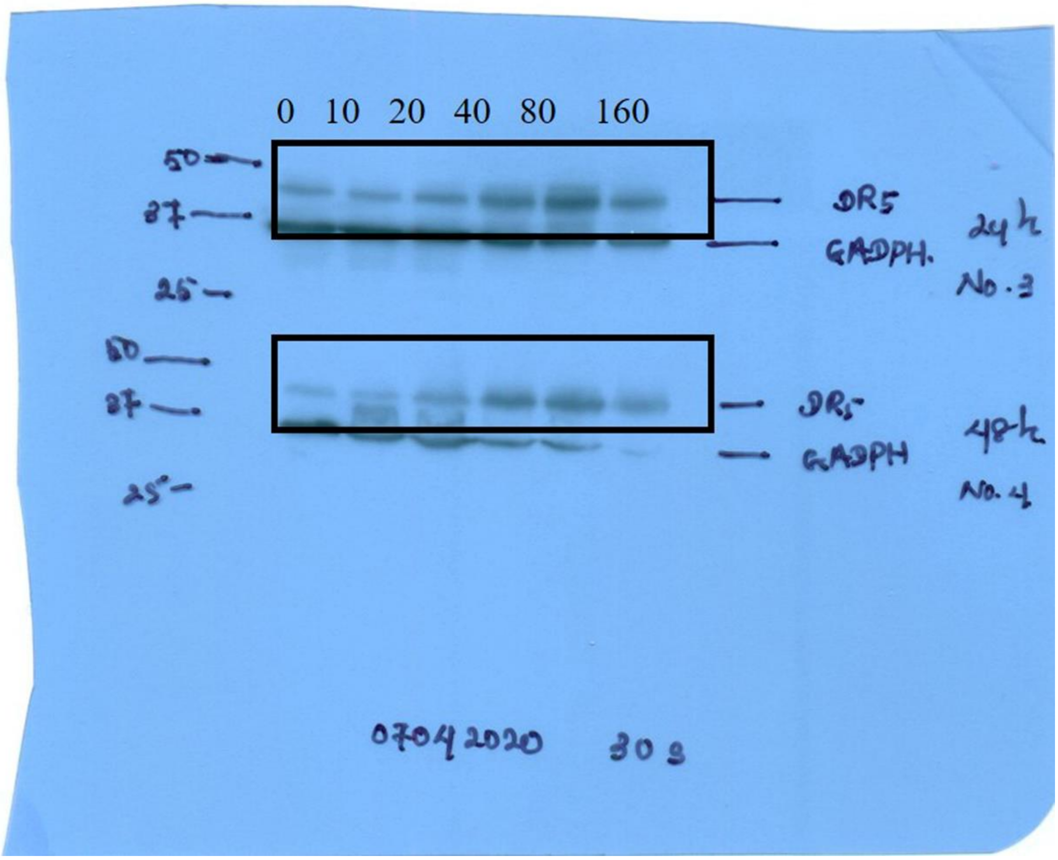

DR5

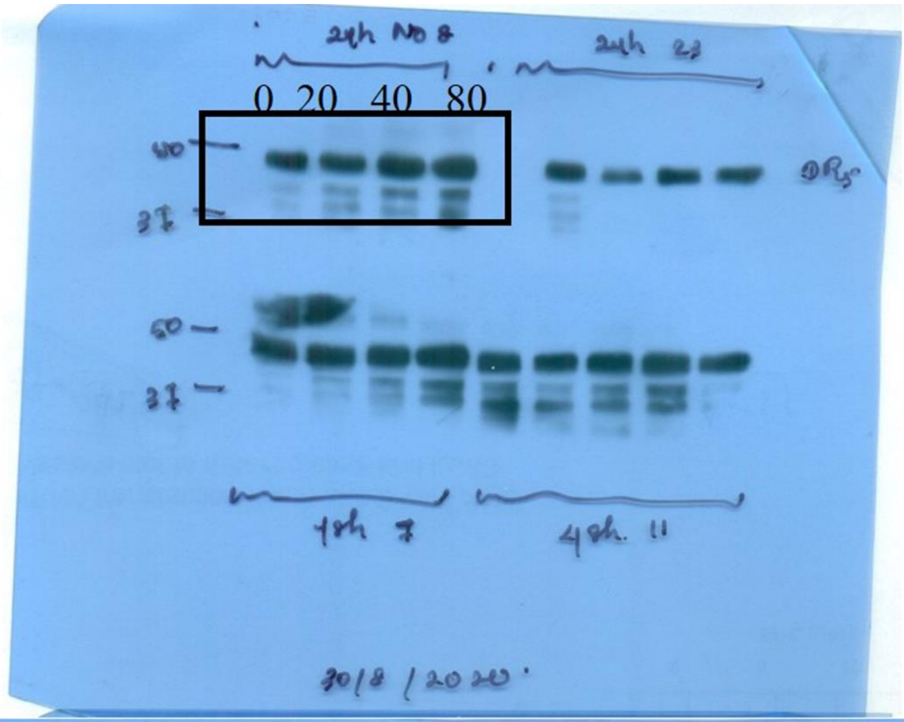

24h

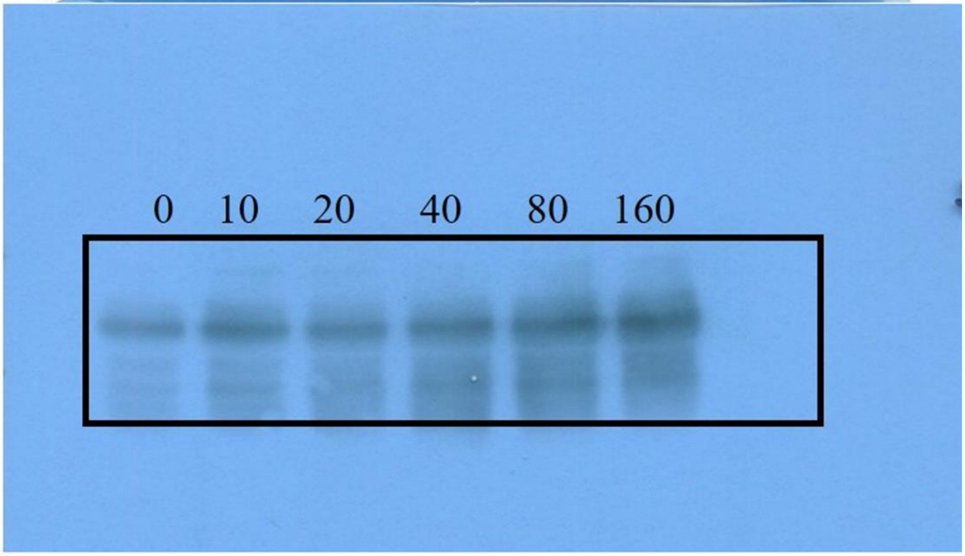

DR5

24 h

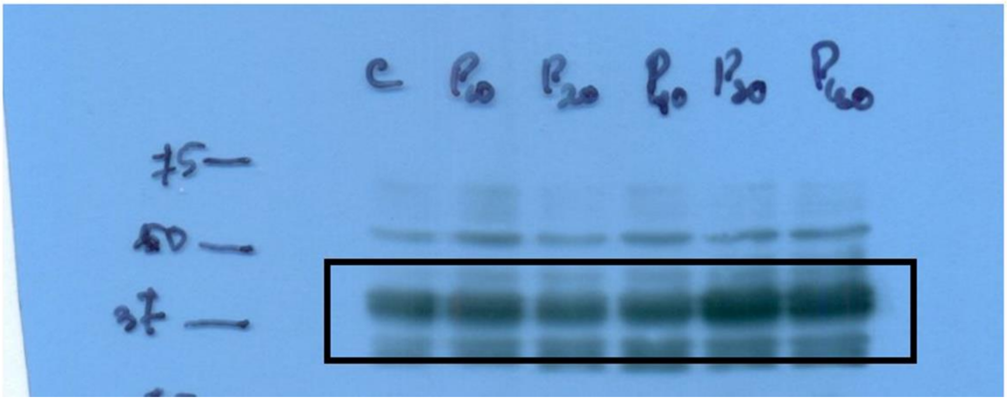

FADD

24 h

FADD

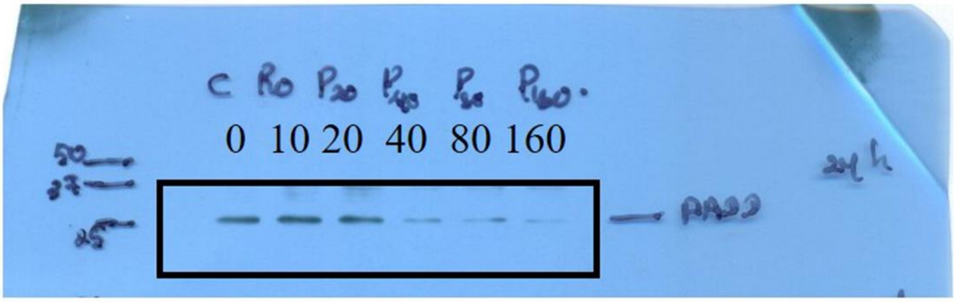

FADD

24 h

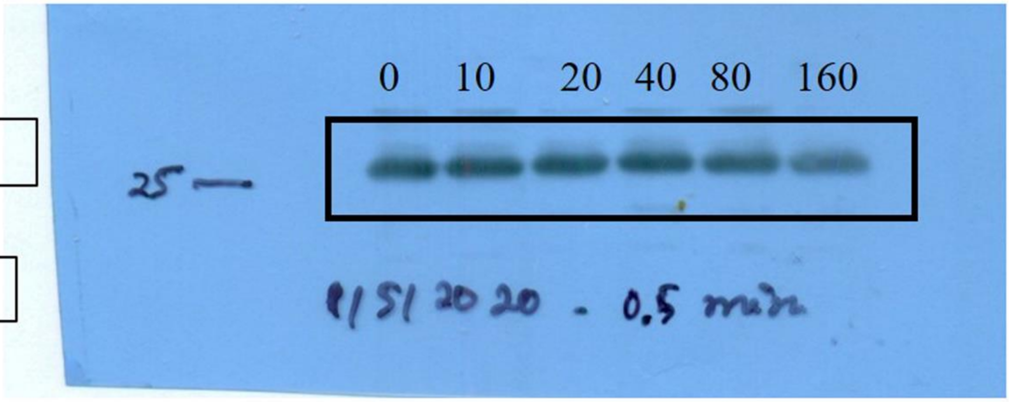

FADD

24 h

48 h

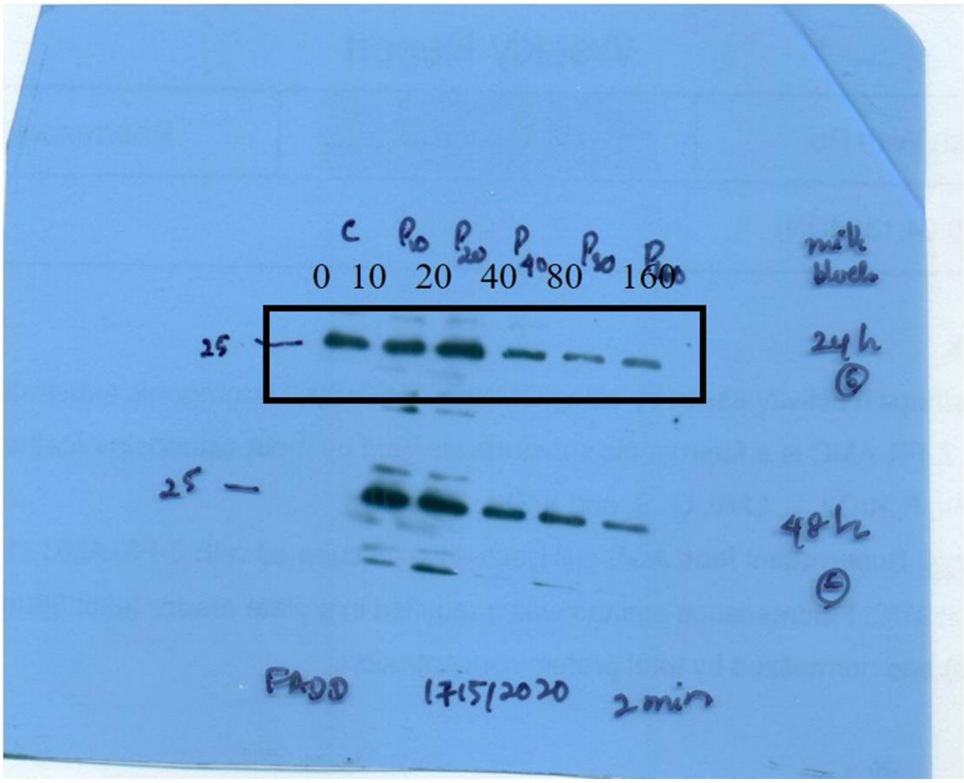

Fig.4b

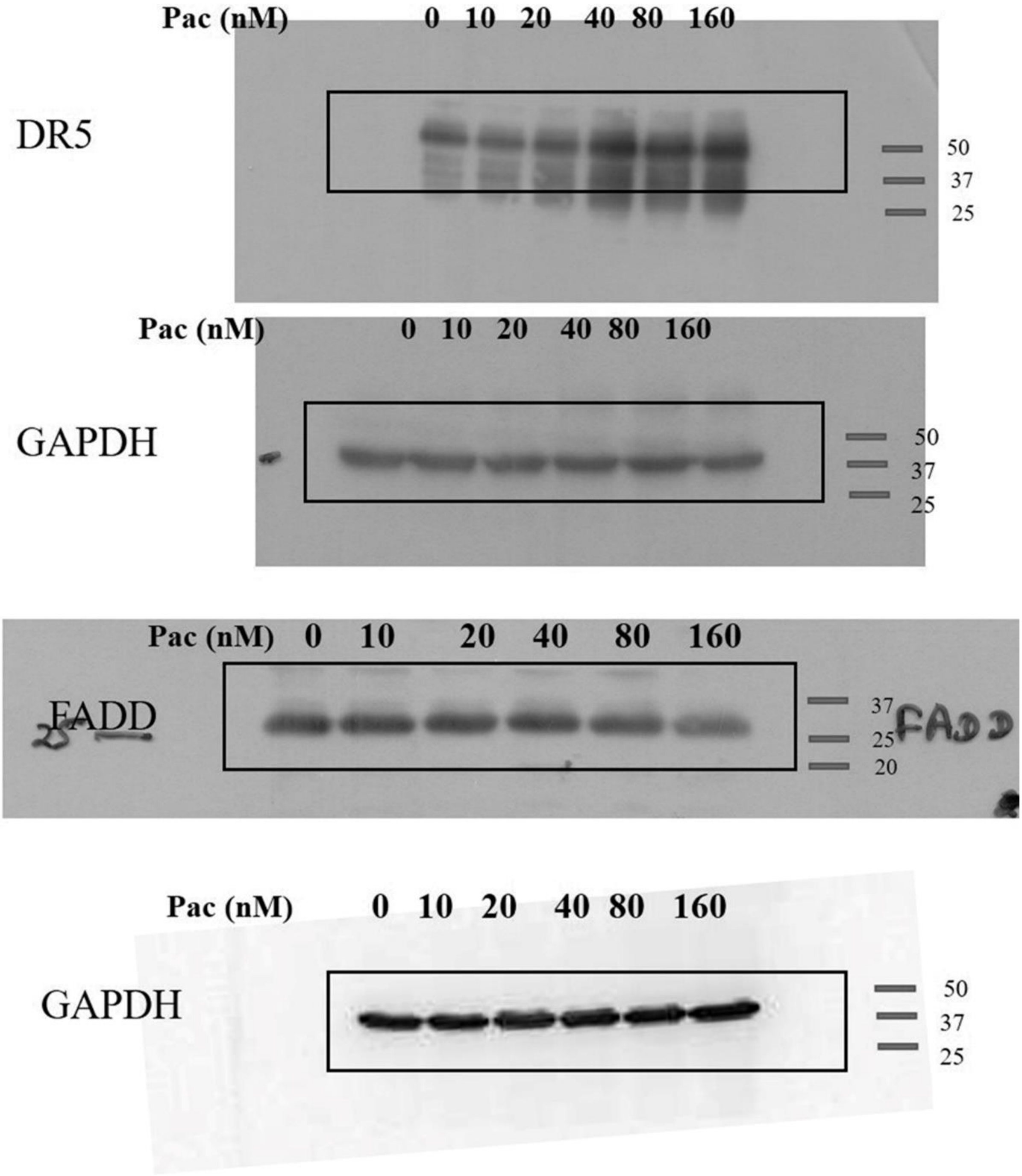
Pac (nM) 0 10 20 40 80 160
DR5
50
37
25
Pac (nM) 0 10 20 40 80 160
GAPDH
50
37
25
Pac (nM) 0 10 20 40 80 160
FADD
37
25
20
FADD
Pac (nM) 0 10 20 40 80 160
GAPDH
50
37
25

DR5

DR5

48h

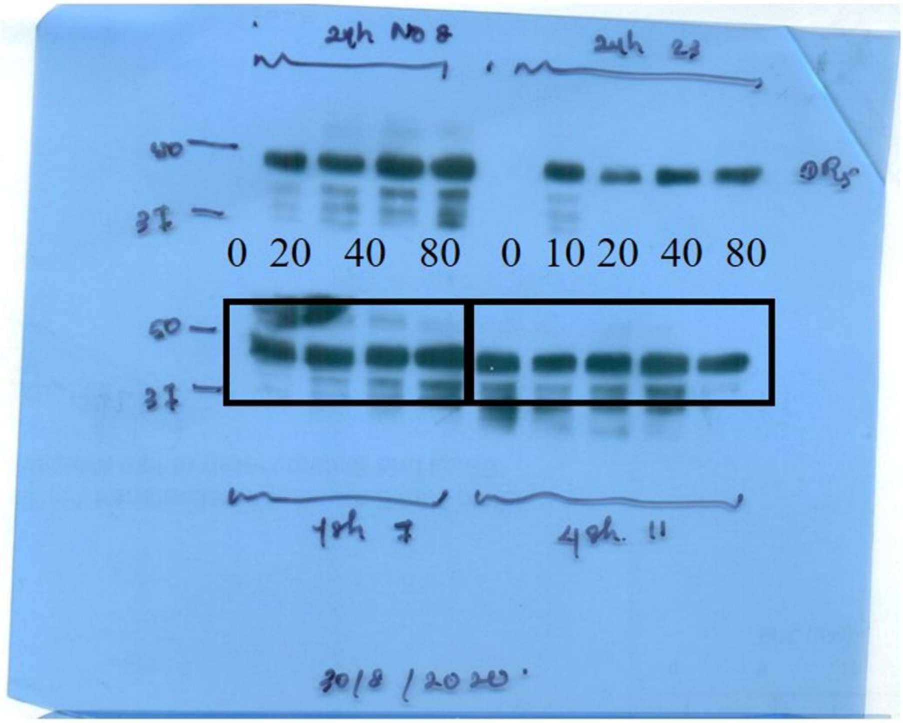

DR5

48h

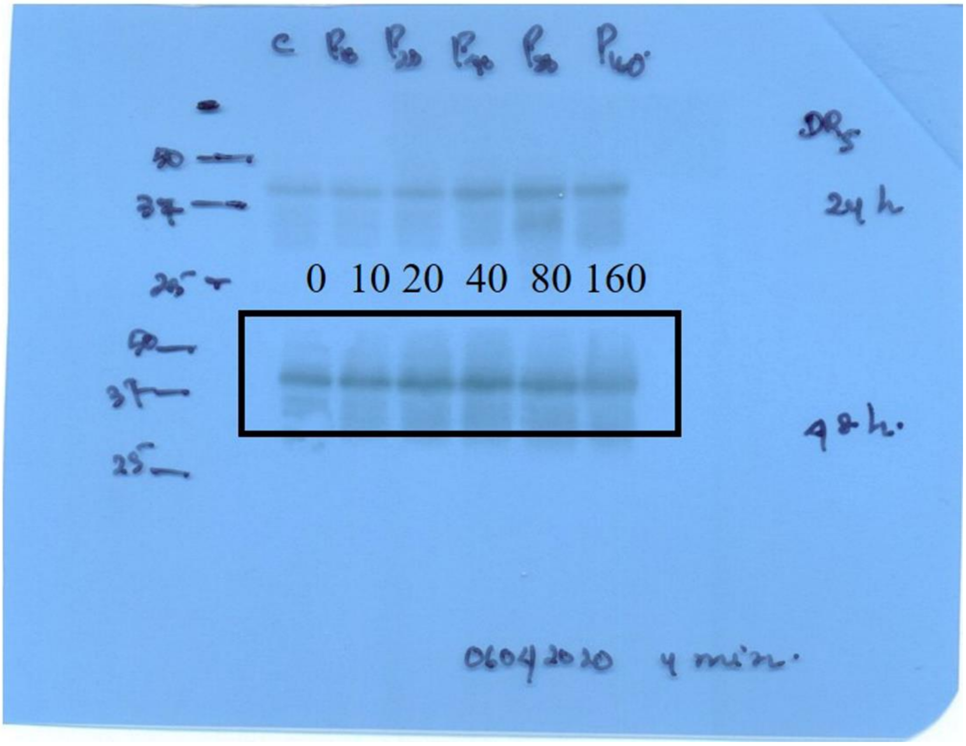

FADD

FADD

24 h

48 h

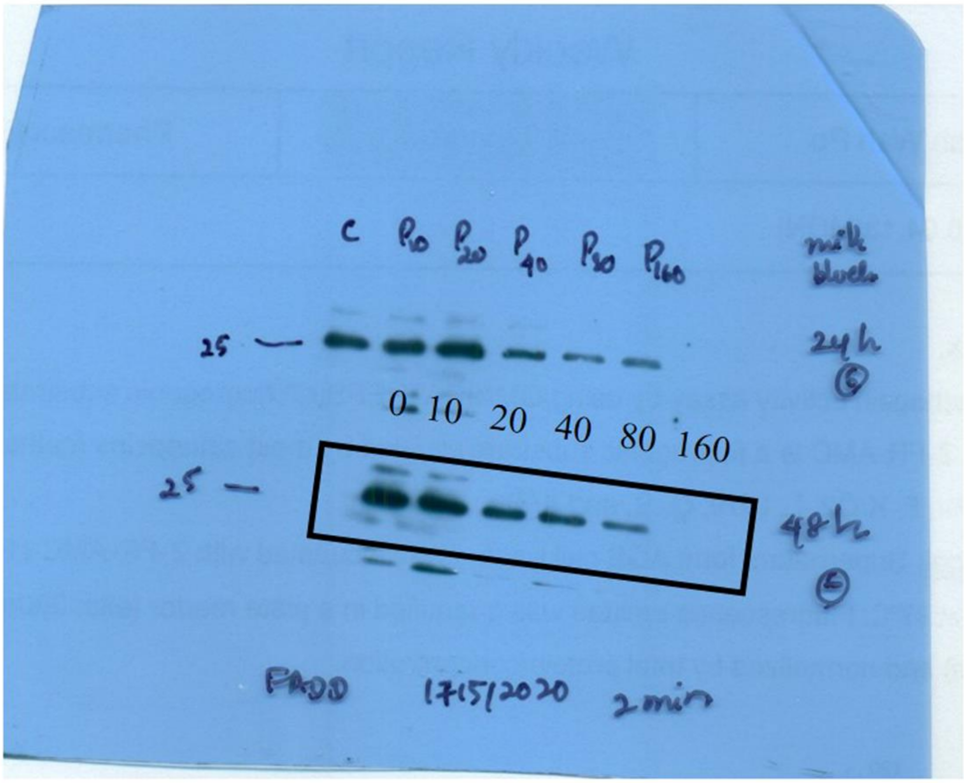

FADD

48 h

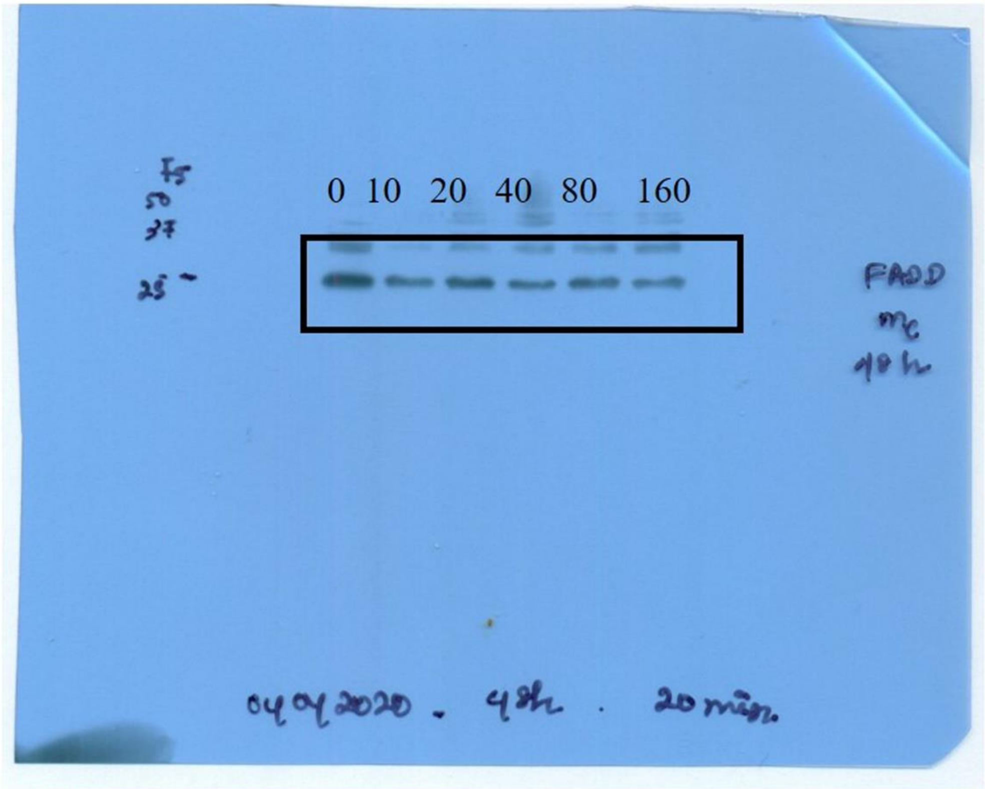

48 h

FADD

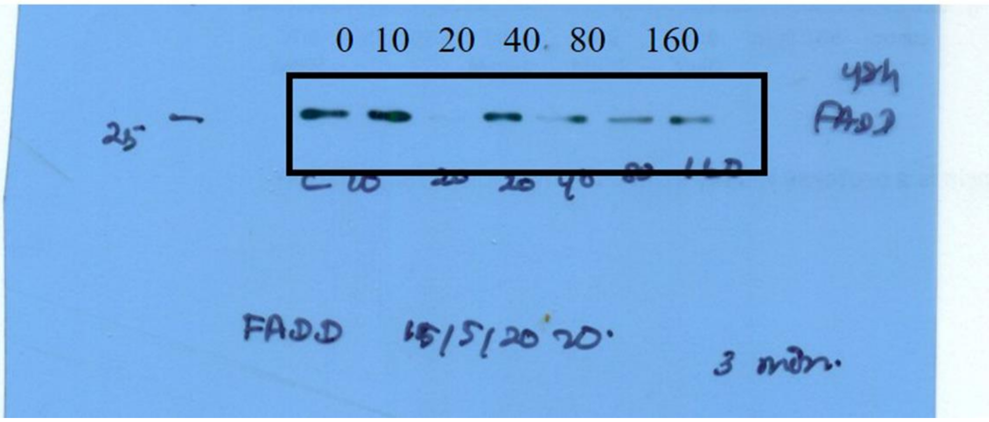

Fig. 4c

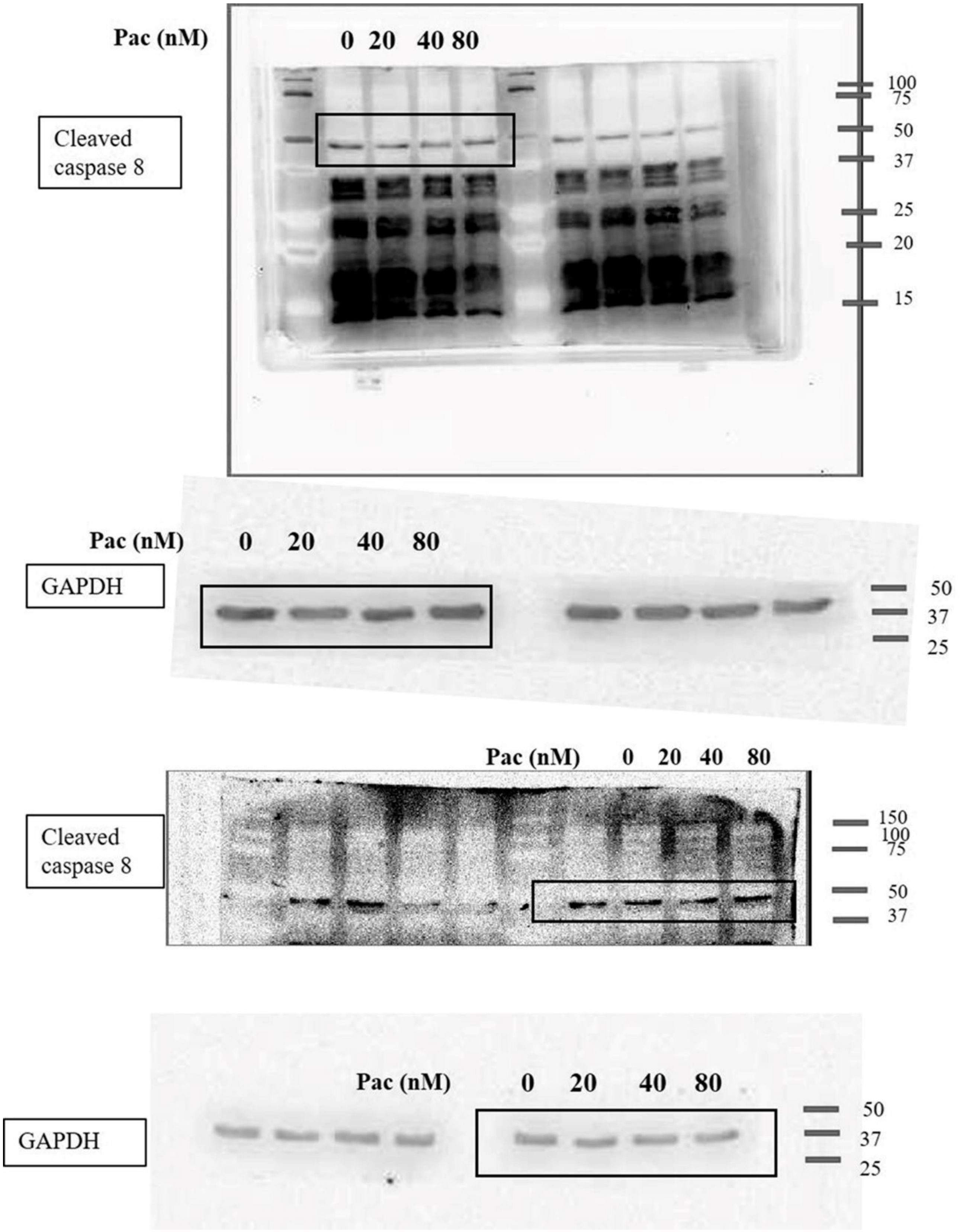

Pac (nM)
0 20 40 80
Cleaved caspase 8
100
75
50
37
25
20
15
Pac (nM)
0 20 40 80
GAPDH
50
37
25
Pac (nM)
0 20 40 80
Cleaved caspase 8
150
100
75
50
37
Pac (nM)
0 20 40 80
GAPDH
50
37
25

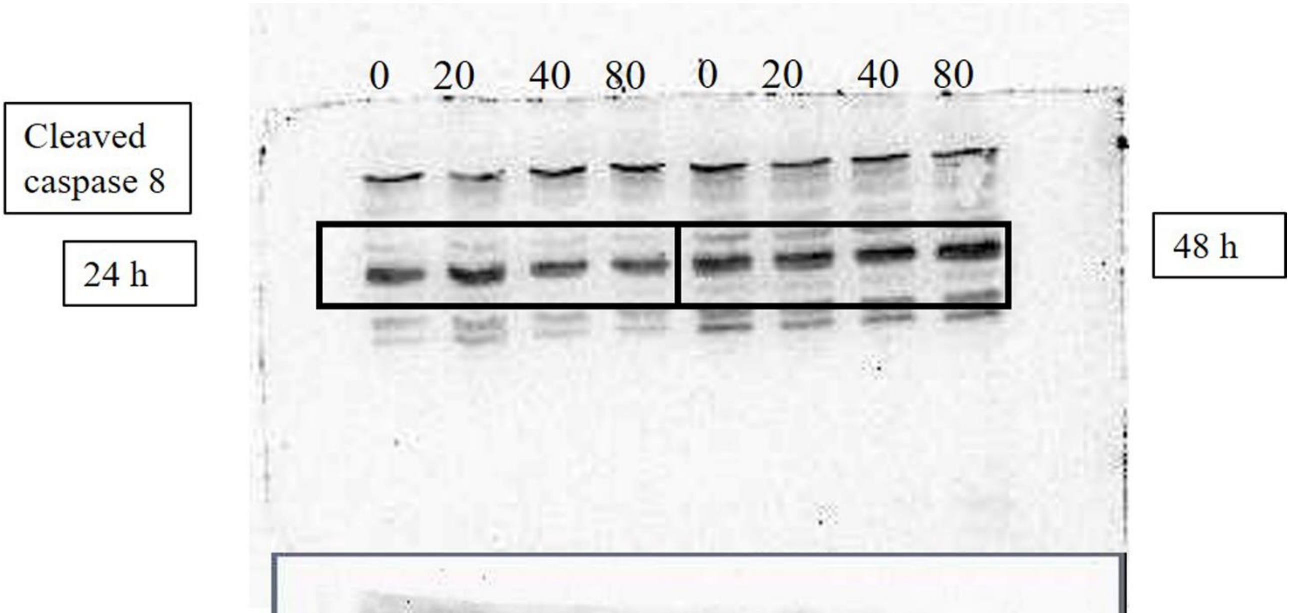
Cleaved caspase 8
24 h
48 h
0 20 40 80 0 20 40 80

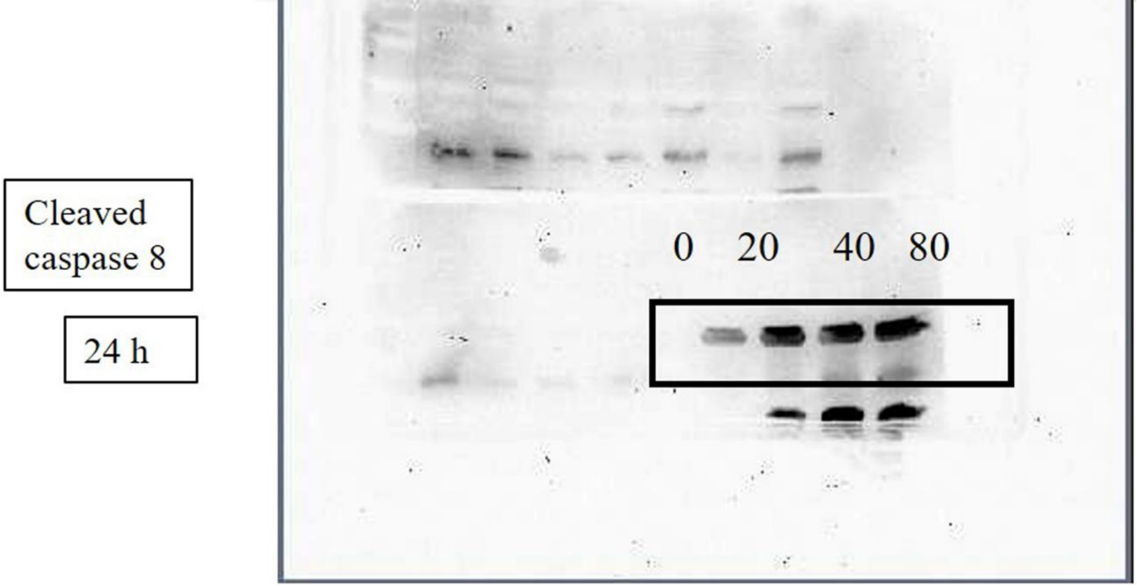
Cleaved caspase 8
24 h
0 20 40 80

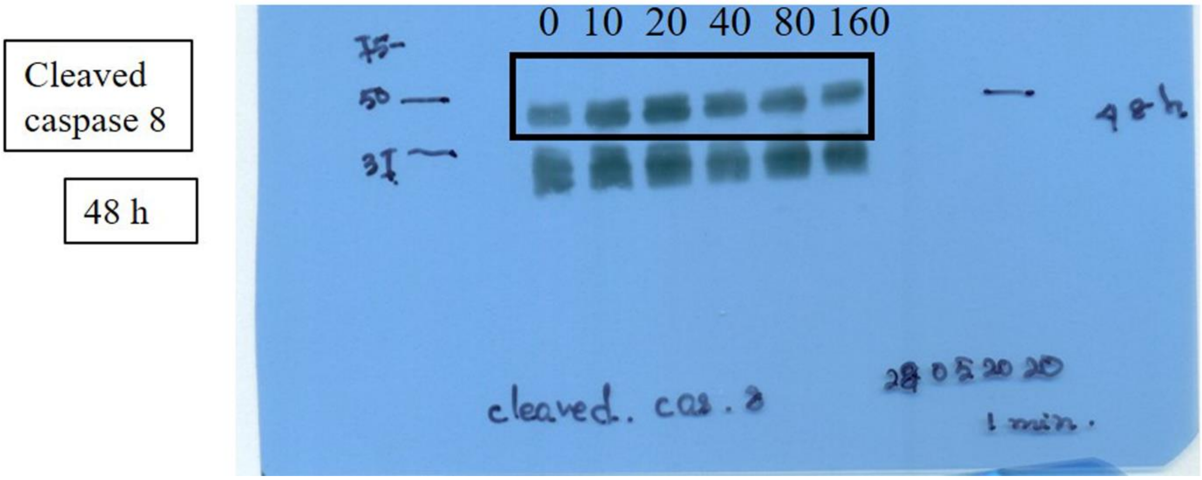
Cleaved caspase 8
48 h
0 10 20 40 80 160
75-
50
37
48 h
cleaved. cas. 8
28 05 2020
1 min.

Cleaved caspase 8

24 h

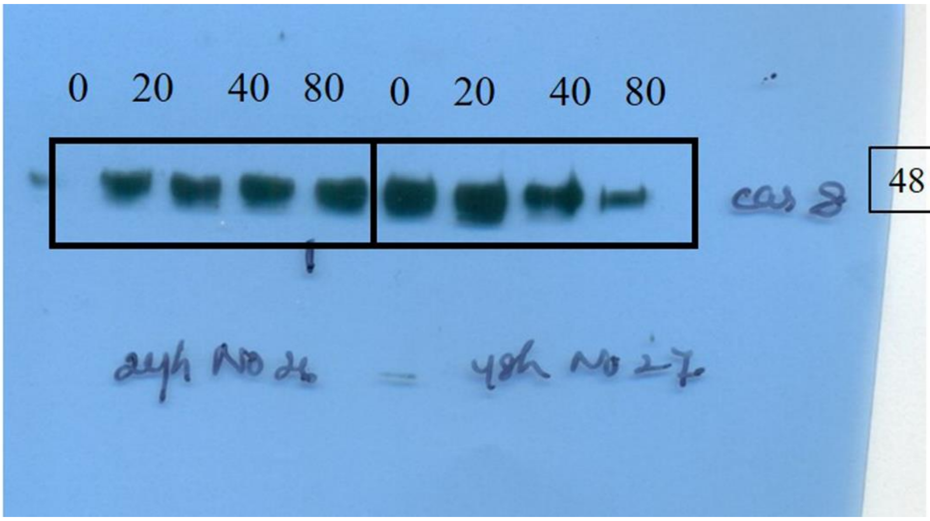

48 h

Supporting data

Fig.EE Photographs of the replicated full-length blots described in Figure 5

Fig. 5c

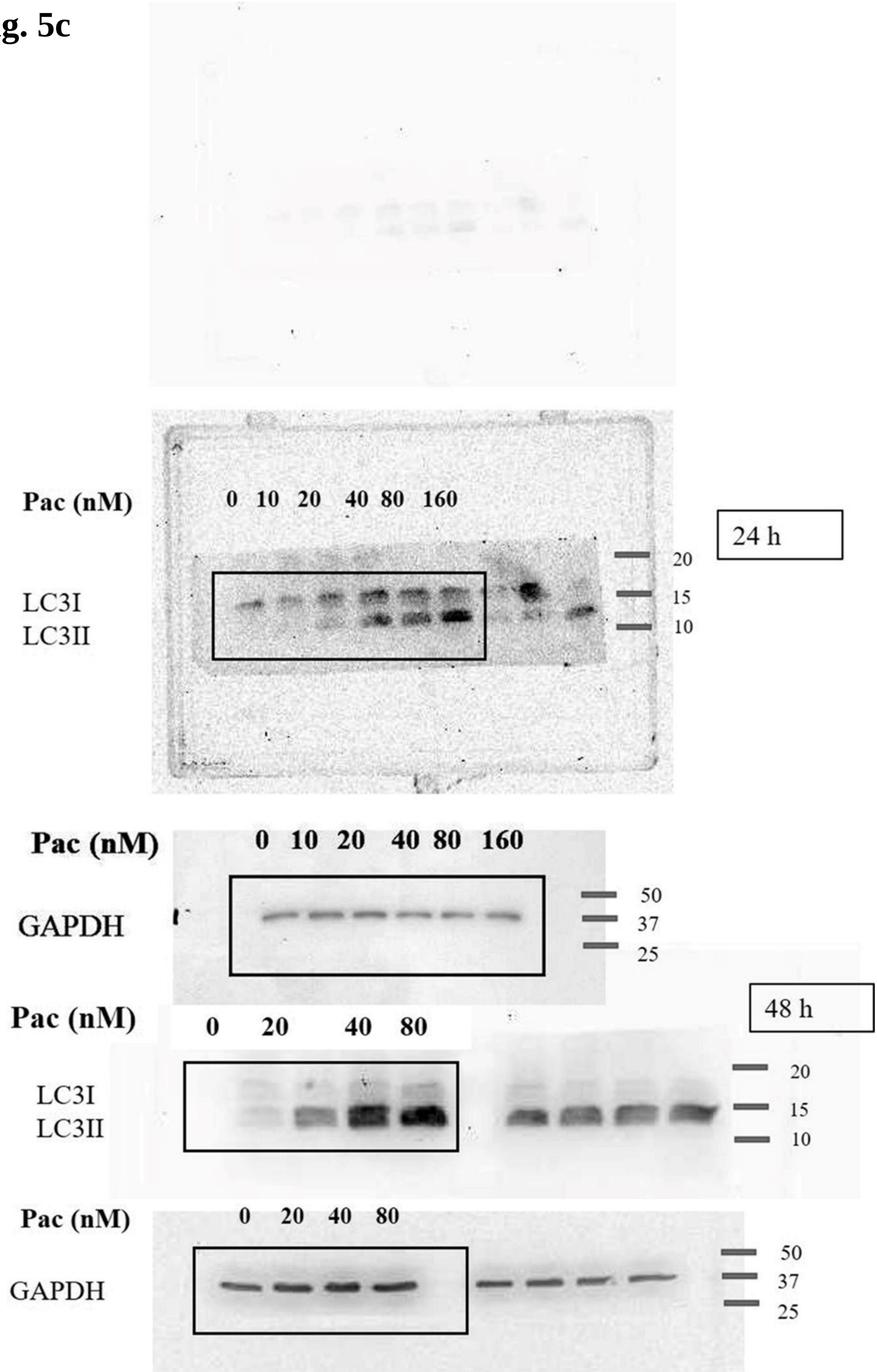

LC3B

24 h

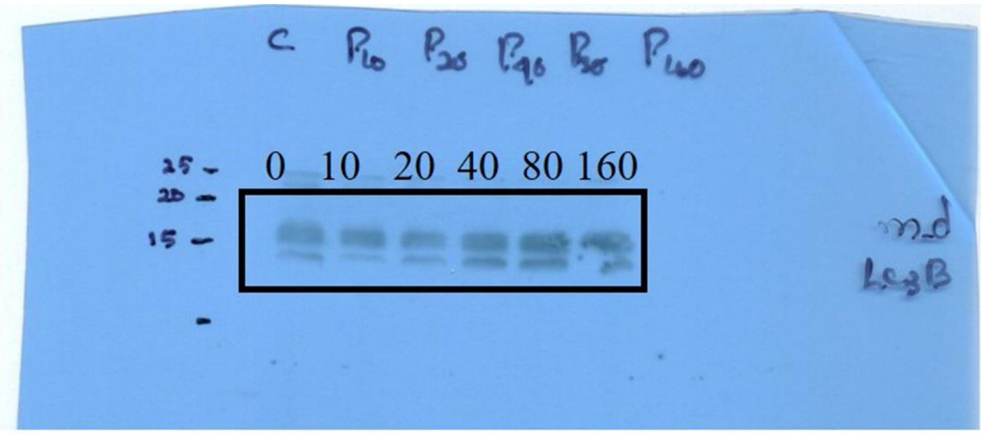

GAPDH

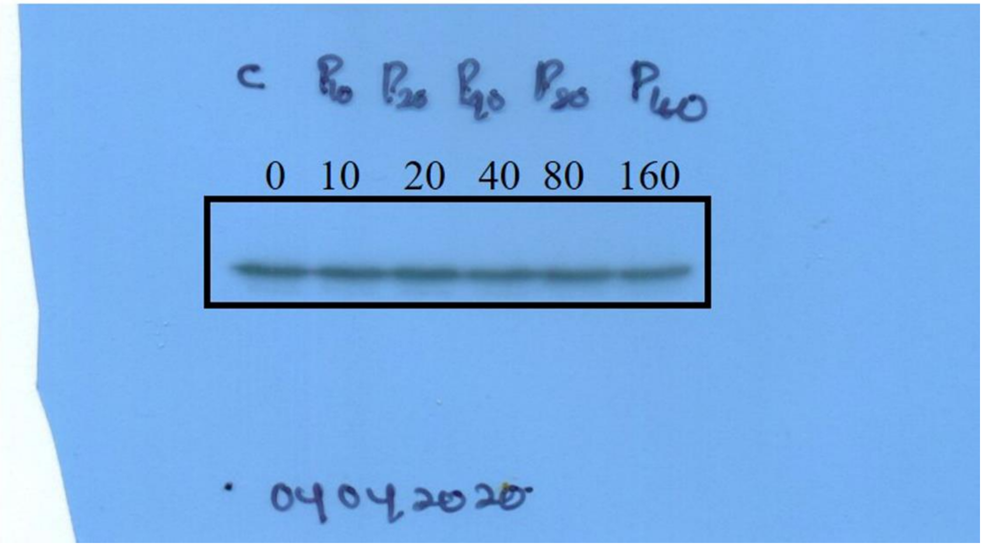

24 h

LC3B

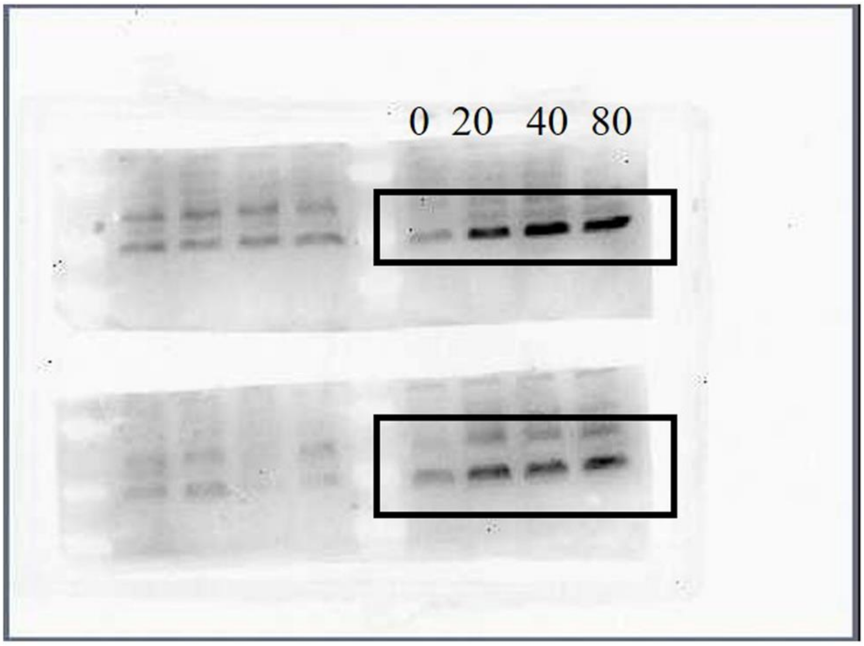

LC3B

24 h

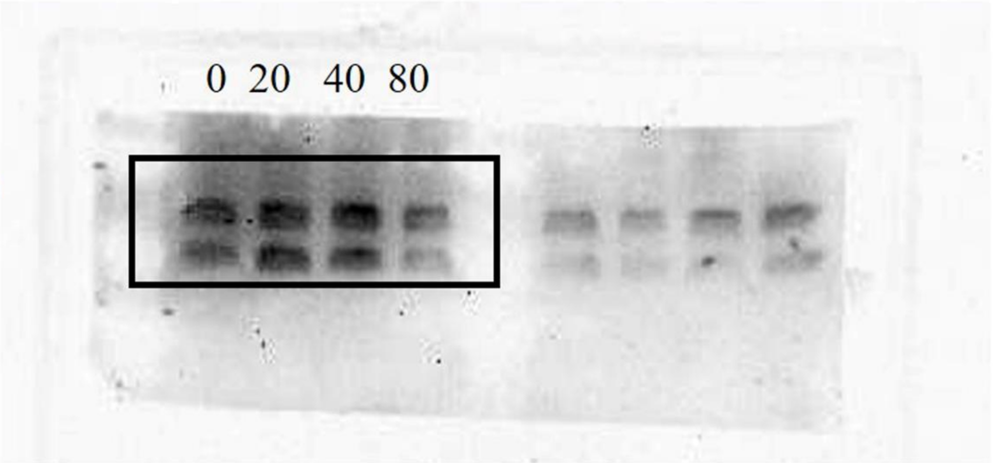

LC3B

24 h

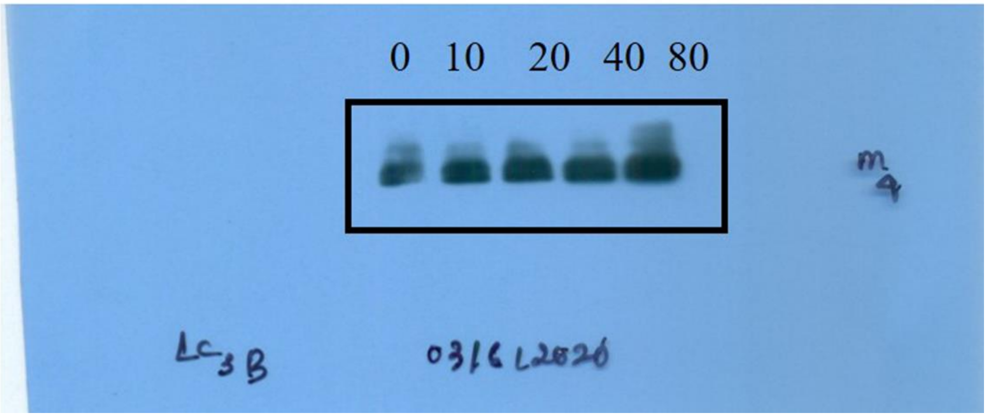

LC3B

0 20 40 80 0 20 40 80

48 h

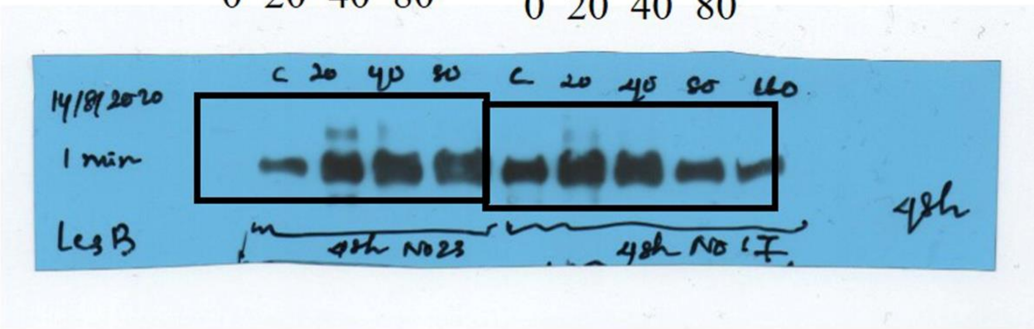

LC3B

48 h

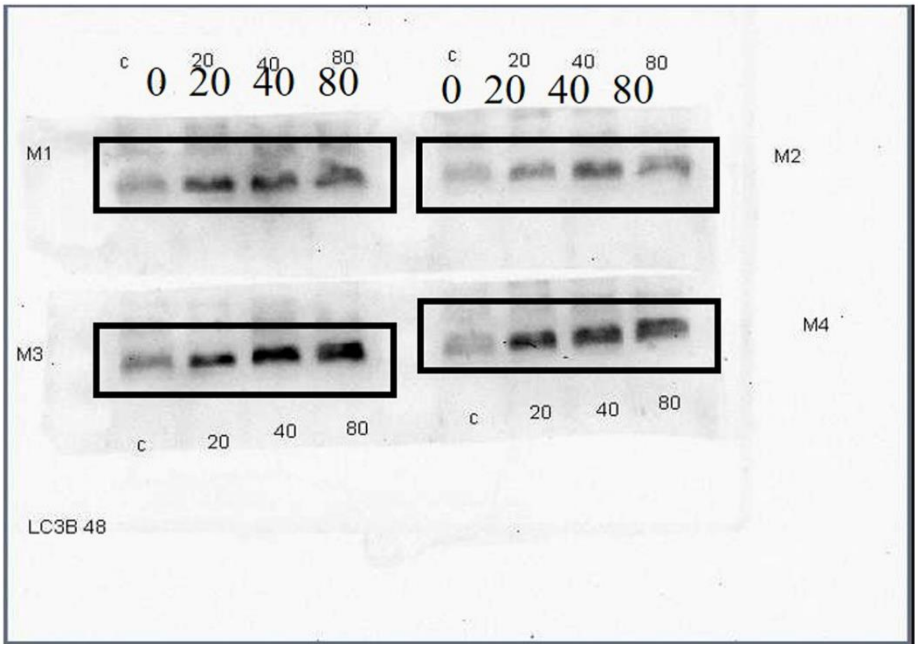

LC3B

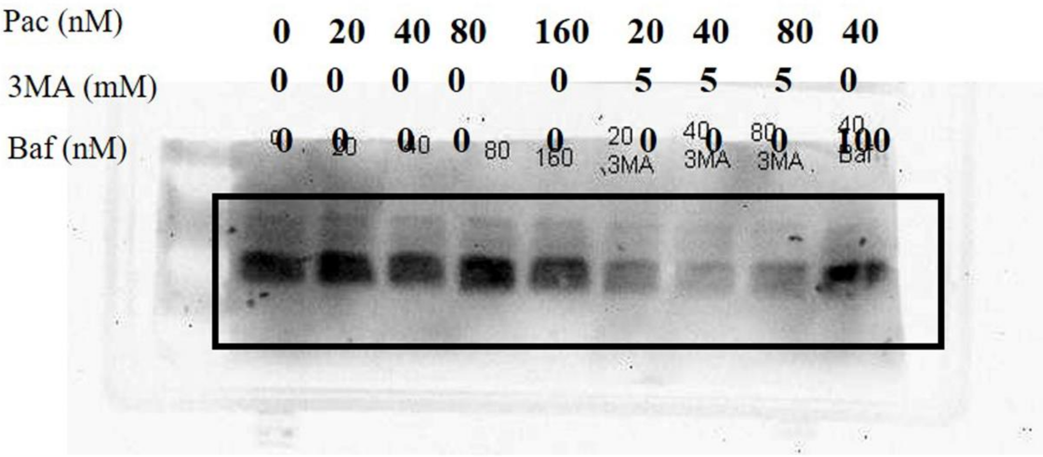
Pac (nM) 0 20 40 80 160 20 40 80 40
3MA (mM) 0 0 0 0 0 5 5 5 0
Baf (nM) 0 0 0 0 0 0 0 0 100

LC3B

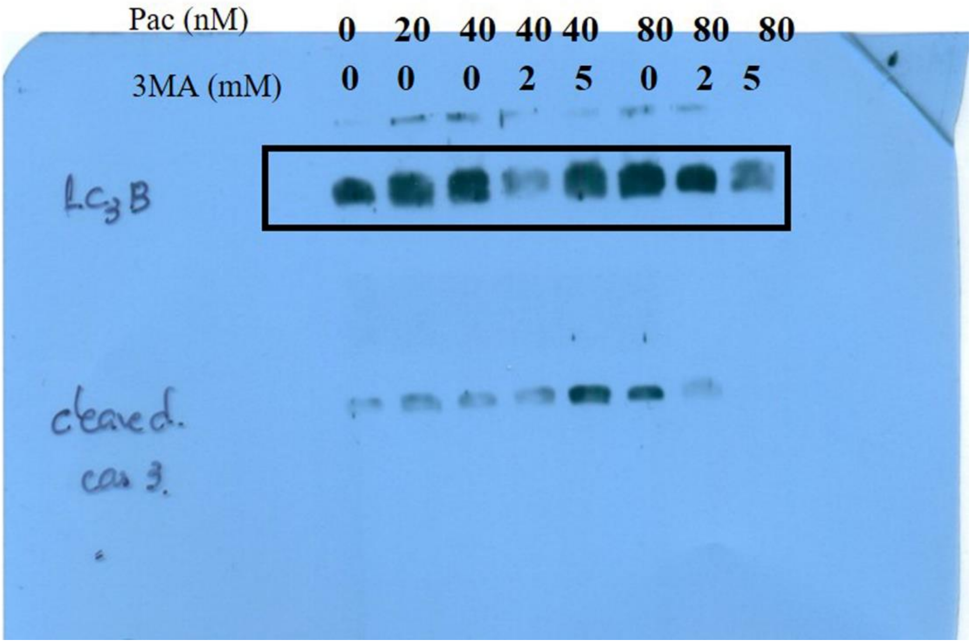
Pac (nM) 0 20 40 40 40 80 80 80
3MA (mM) 0 0 0 2 5 0 2 5
LC3B
cleaved. cas 3.

Fig. 5d

24 h

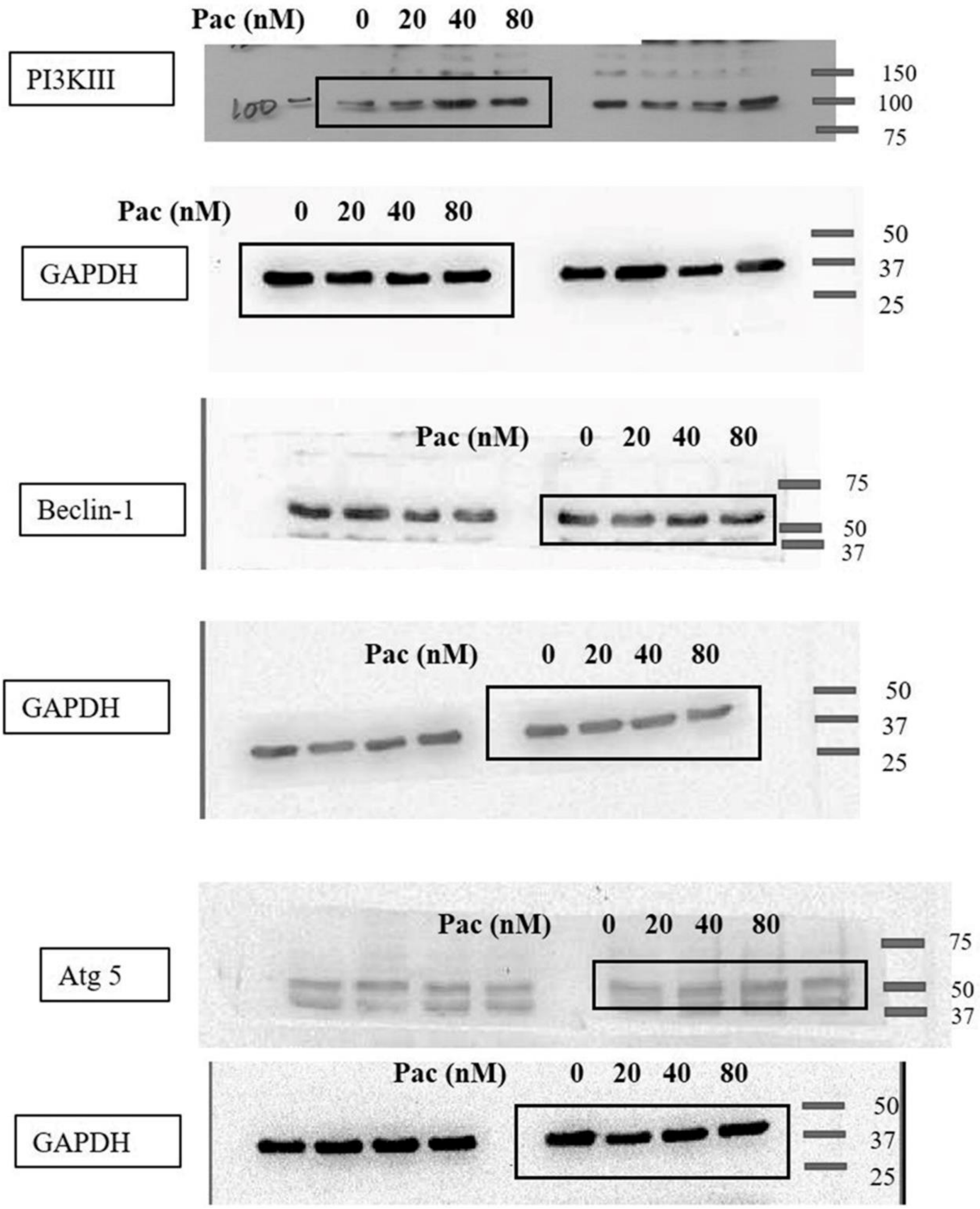

Pac (nM) 0 20 40 80
PI3KIII
150
100
75
Pac (nM) 0 20 40 80
GAPDH
50
37
25
Pac (nM) 0 20 40 80
Beclin-1
75
50
37
Pac (nM) 0 20 40 80
GAPDH
50
37
25
Pac (nM) 0 20 40 80
Atg 5
75
50
37
Pac (nM) 0 20 40 80
GAPDH
50
37
25

PI3K

24 h

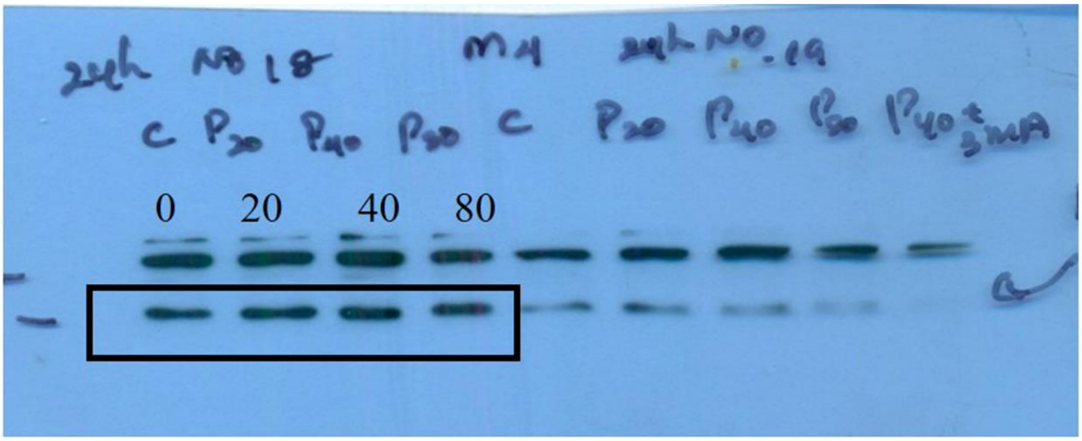

24 h

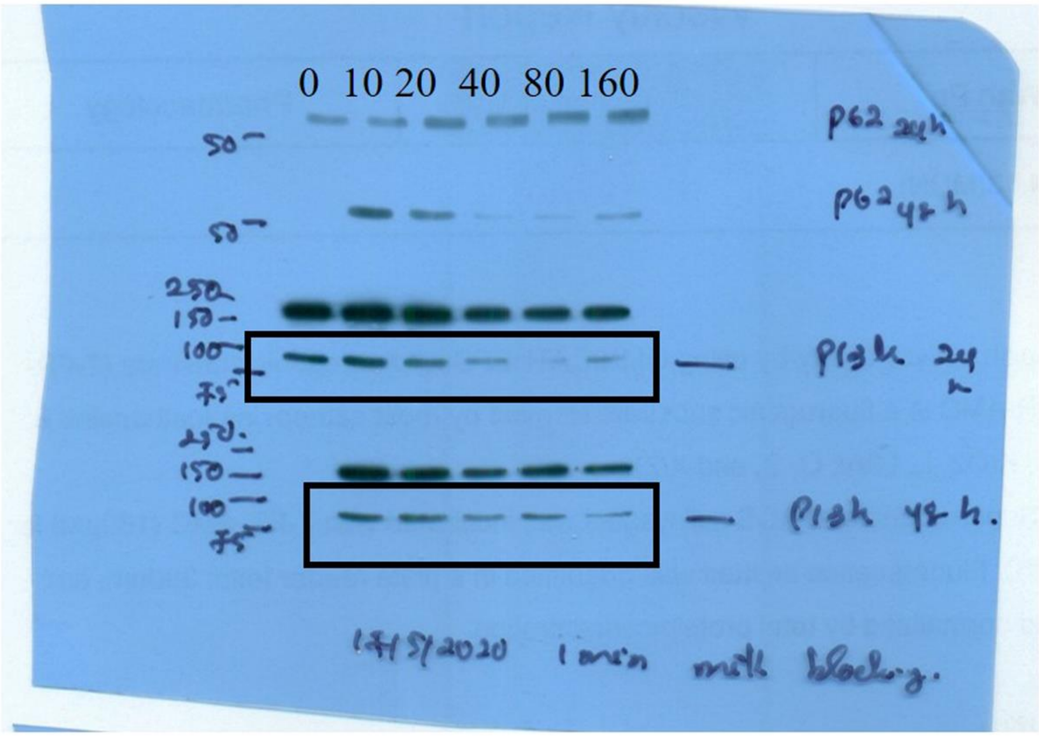

PI3K

24 h

48 h

48 h

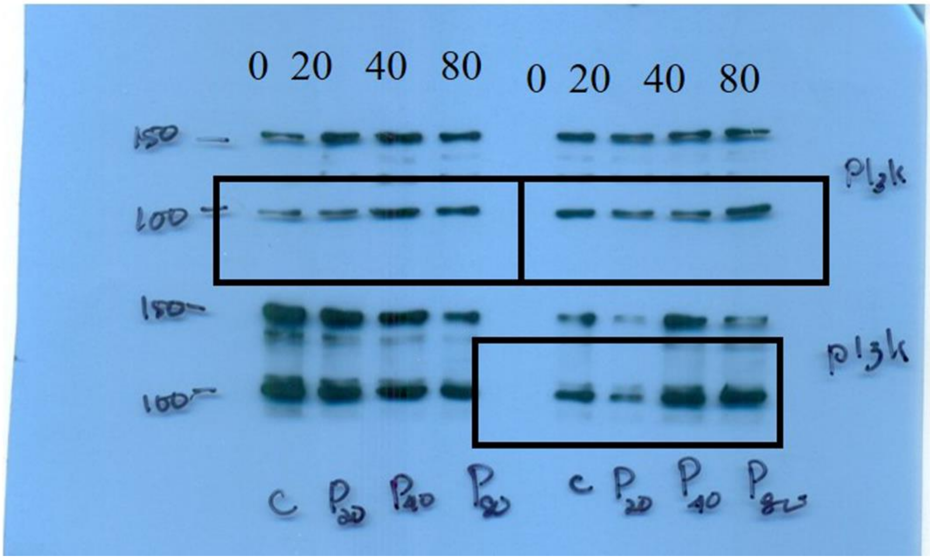

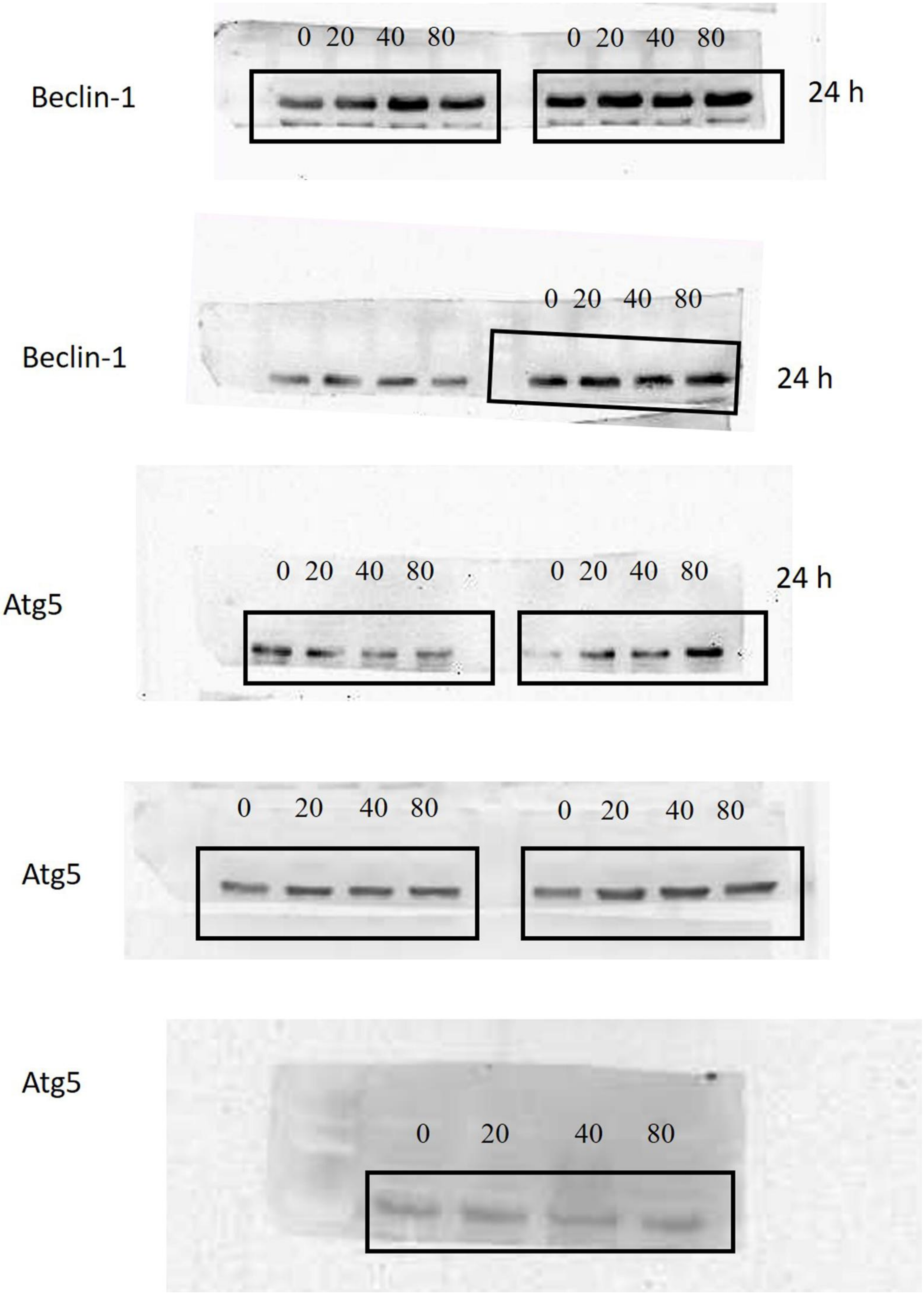
Beclin-1
0 20 40 80
0 20 40 80
24 h
Beclin-1
0 20 40 80
24 h
Atg5
0 20 40 80
0 20 40 80
24 h
Atg5
0 20 40 80
0 20 40 80
Atg5
0 20 40 80

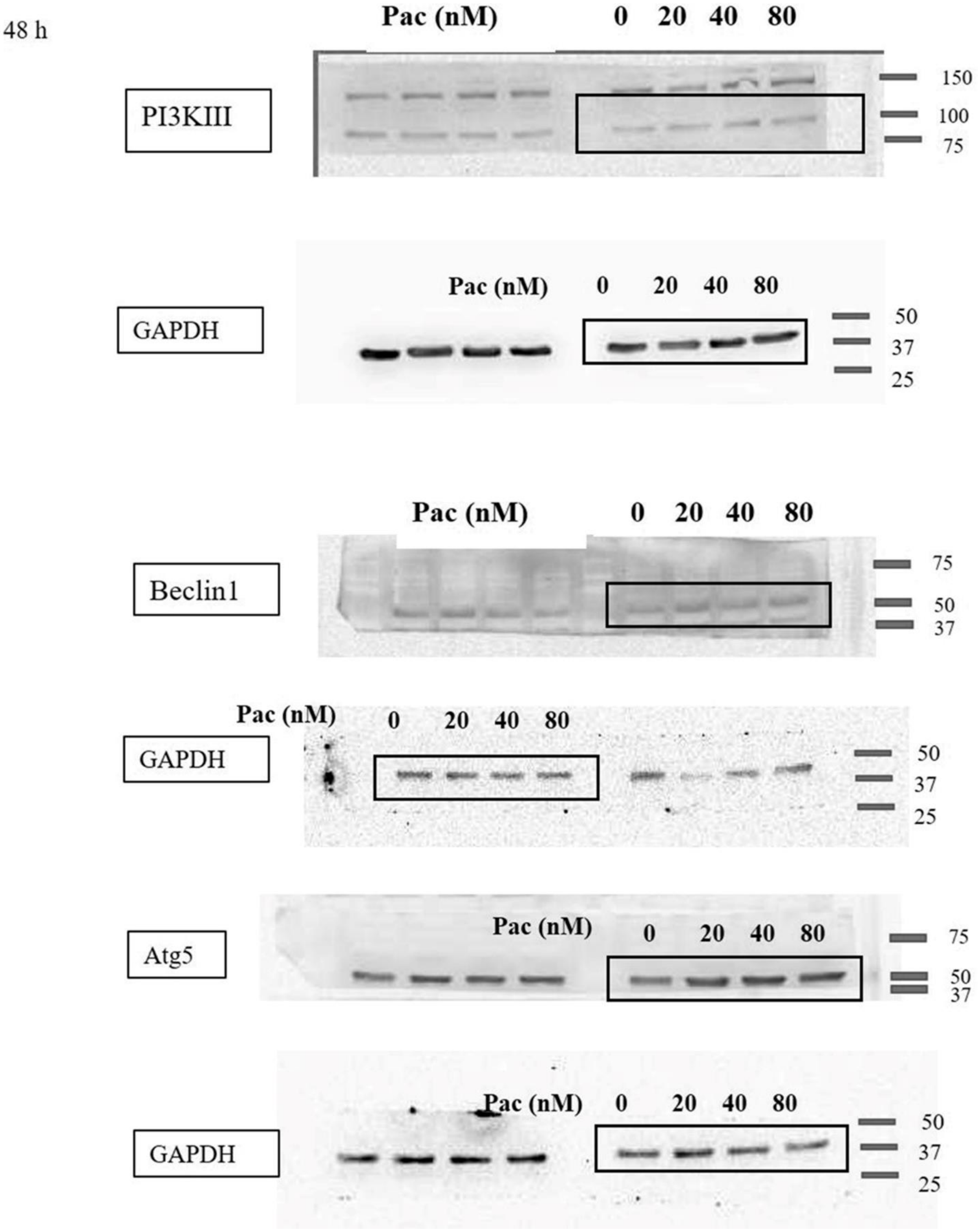
48 h
Pac (nM)
0
20
40
80
PI3KIII
150
100
75
Pac (nM)
0
20
40
80
GAPDH
50
37
25
Pac (nM)
0
20
40
80
Beclin1
75
50
37
Pac (nM)
0
20
40
80
GAPDH
50
37
25
Atg5
Pac (nM)
0
20
40
80
75
50
37
Pac (nM)
0
20
40
80
GAPDH
50
37
25

48 h

Atg5

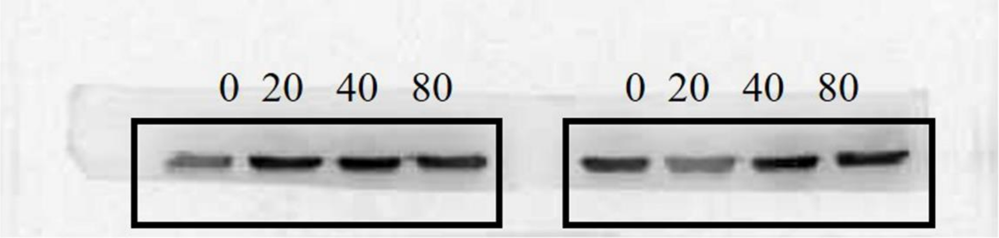

Atg5

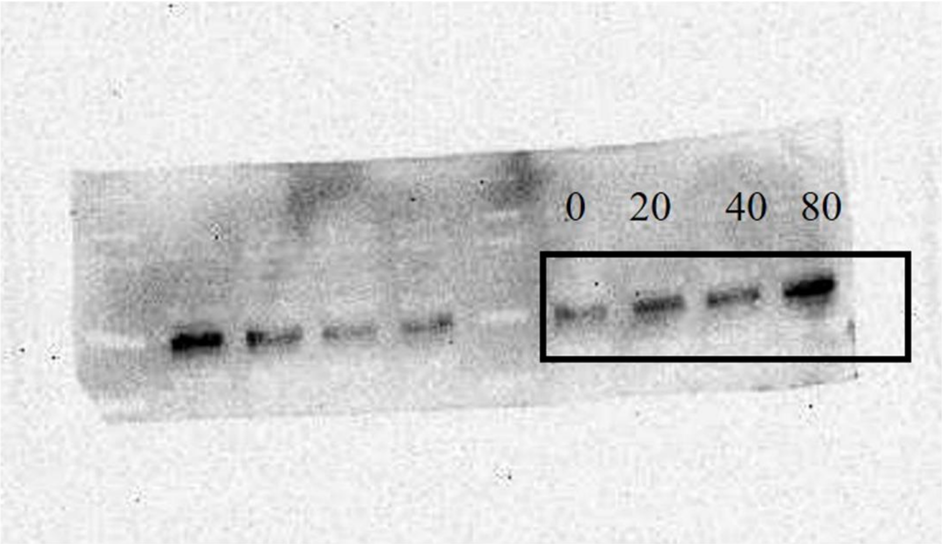

48 h

Atg5

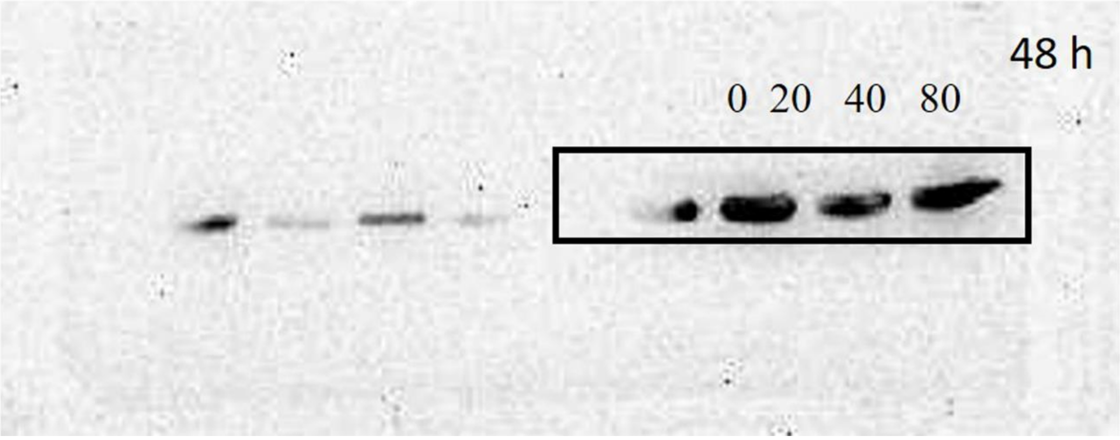

Beclin-1

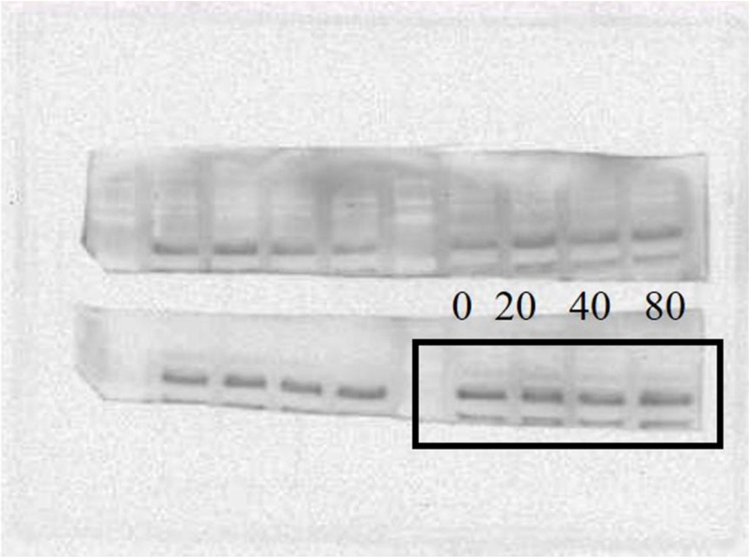

48 h

Beclin-1

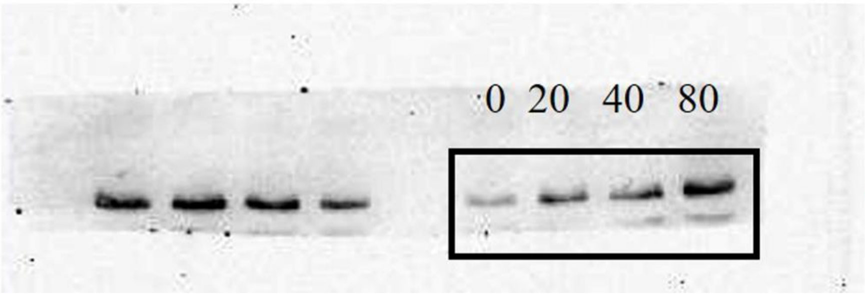

48 h

Fig. 5e

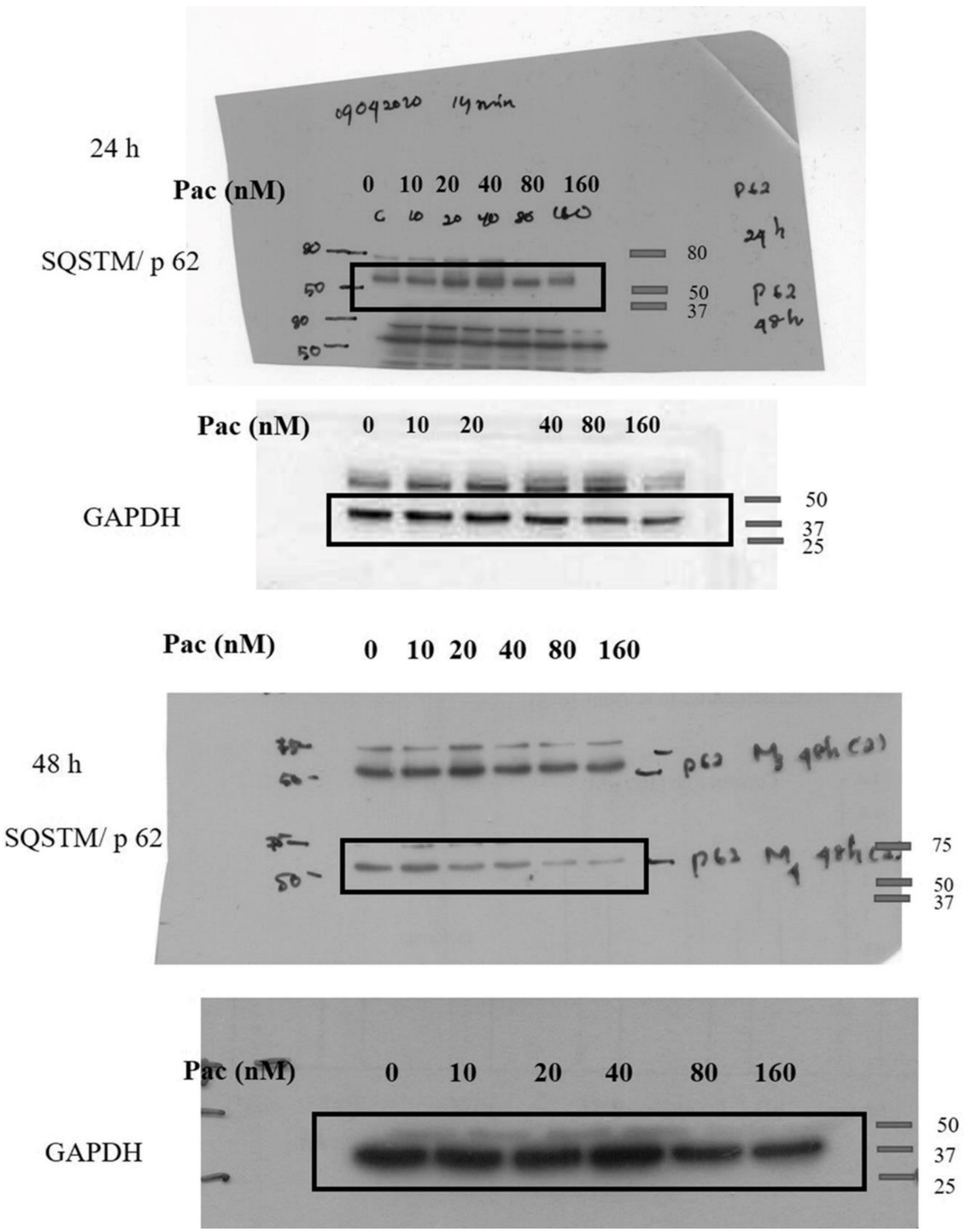
24 h
Pac (nM) 0 10 20 40 80 160
SQSTM/ p 62
80
50
37
Pac (nM) 0 10 20 40 80 160
GAPDH
50
37
25
Pac (nM) 0 10 20 40 80 160
48 h
SQSTM/ p 62
75
50
37
Pac (nM) 0 10 20 40 80 160
GAPDH
50
37
25

P 62

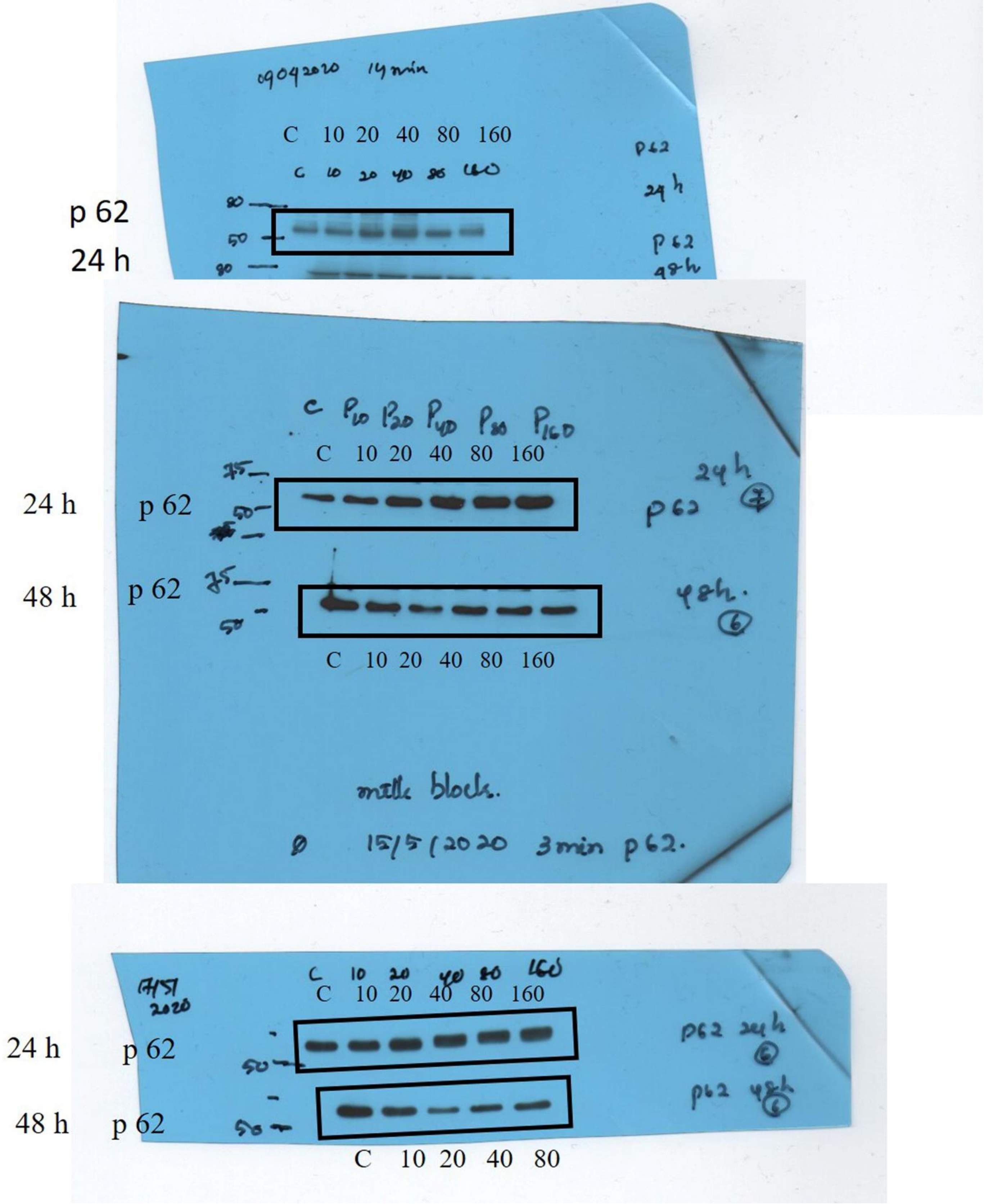
09042020 14 min
C 10 20 40 80 160
p 62
24 h
p62
24 h
p62
48h
C P10 P20 P40 P80 P160
C 10 20 40 80 160
24 h p 62
48 h p 62
C 10 20 40 80 160
24 h
p62
48h.
milk block.
15/5/2020 3 min p62.
C 10 20 40 80 160
C 10 20 40 80 160
24 h p 62
48 h p 62
p62 24h
p62 48h
C 10 20 40 80

P 62

p 62

24 h

24 h

48 h

48 h

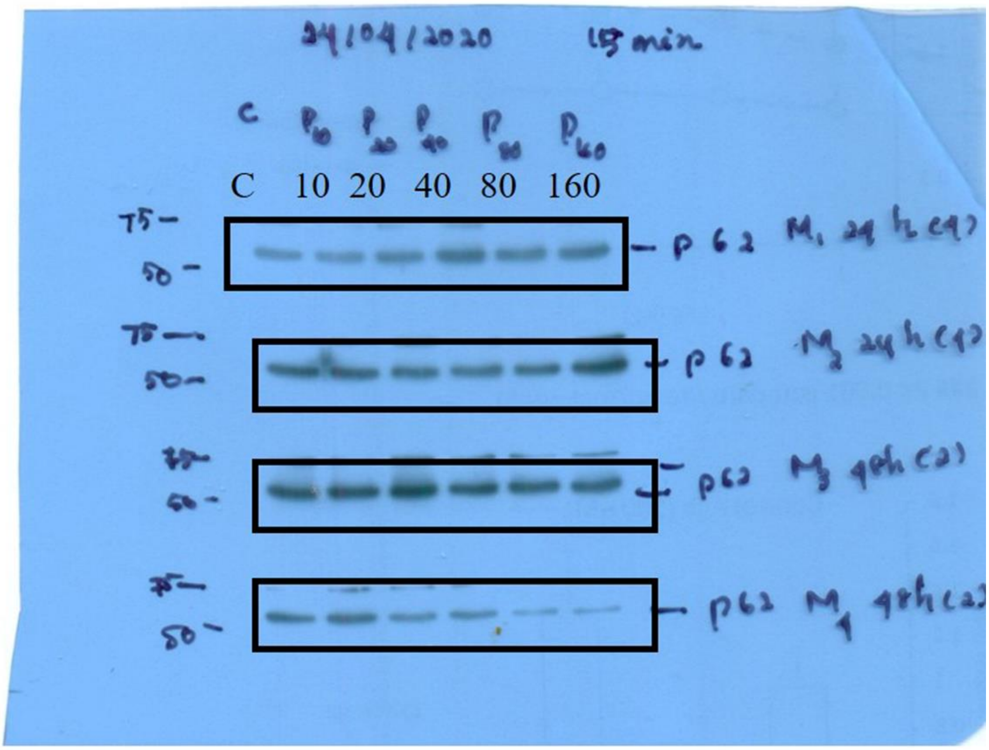

24 h p62

48 h p62

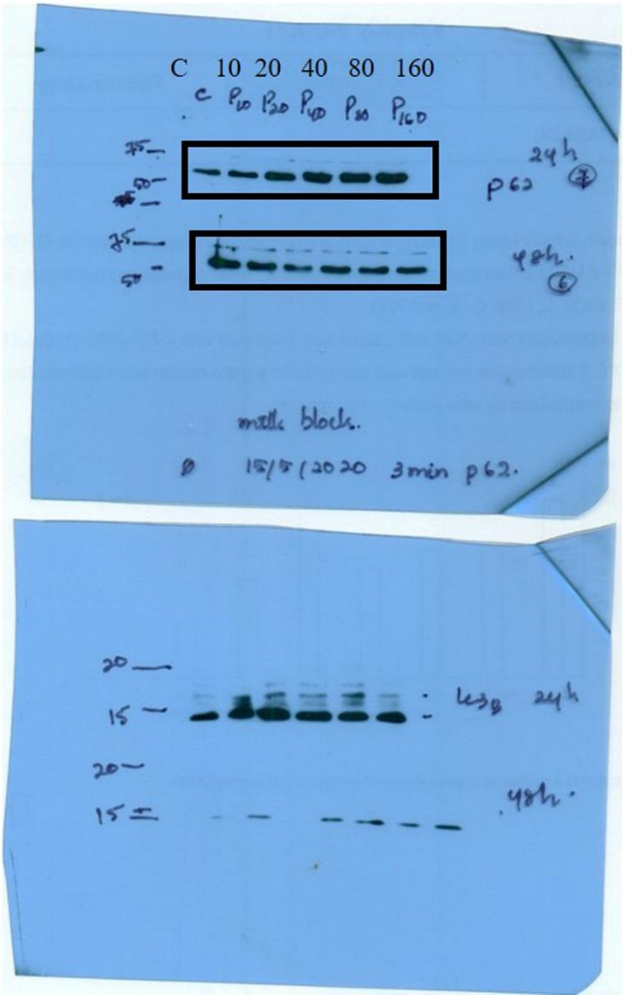

Fig. 5f

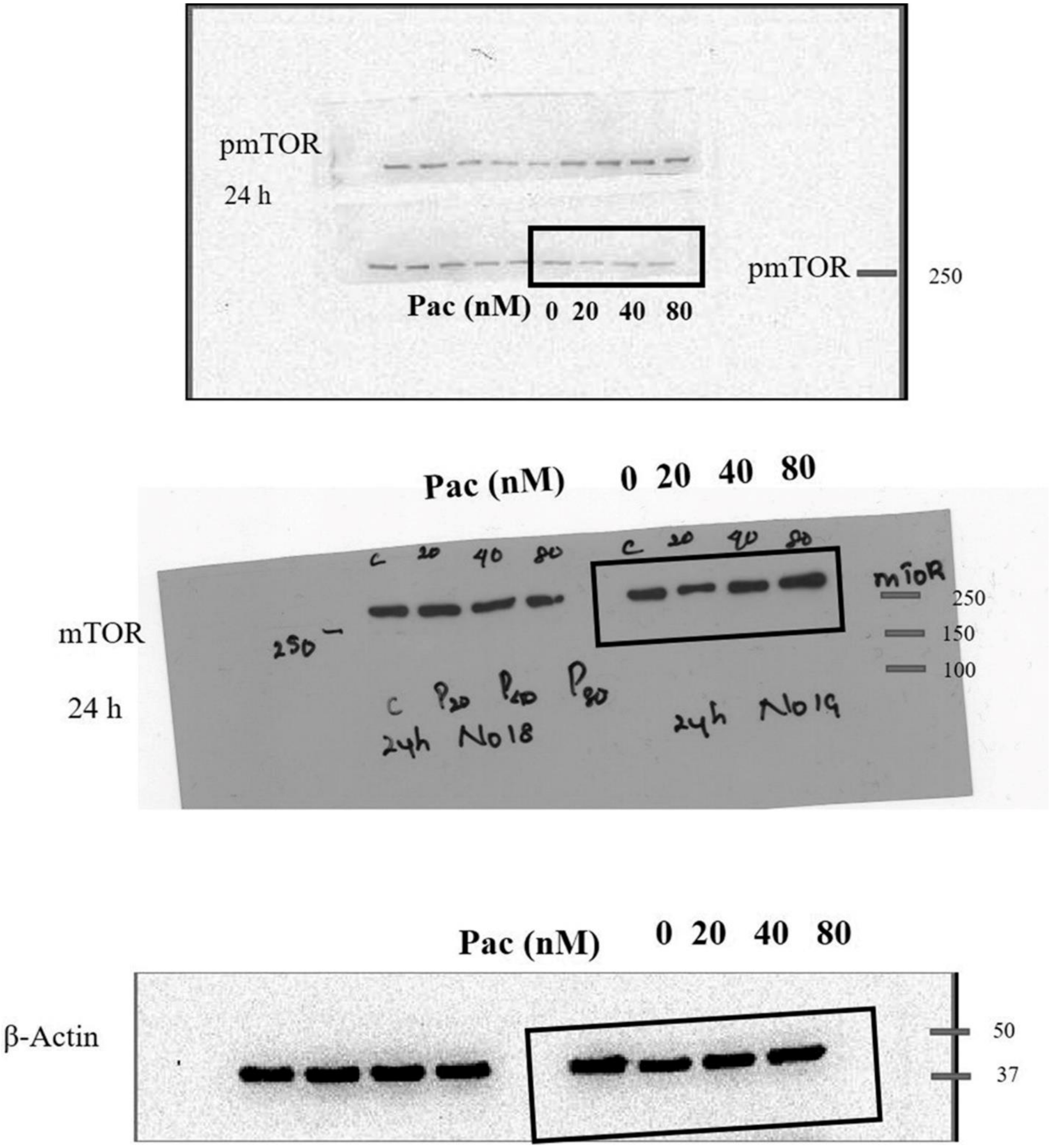
pmTOR
24 h
Pac (nM) 0 20 40 80
pmTOR
250
Pac (nM) 0 20 40 80
mTOR
24 h
250
150
100
β-Actin
Pac (nM) 0 20 40 80
50
37

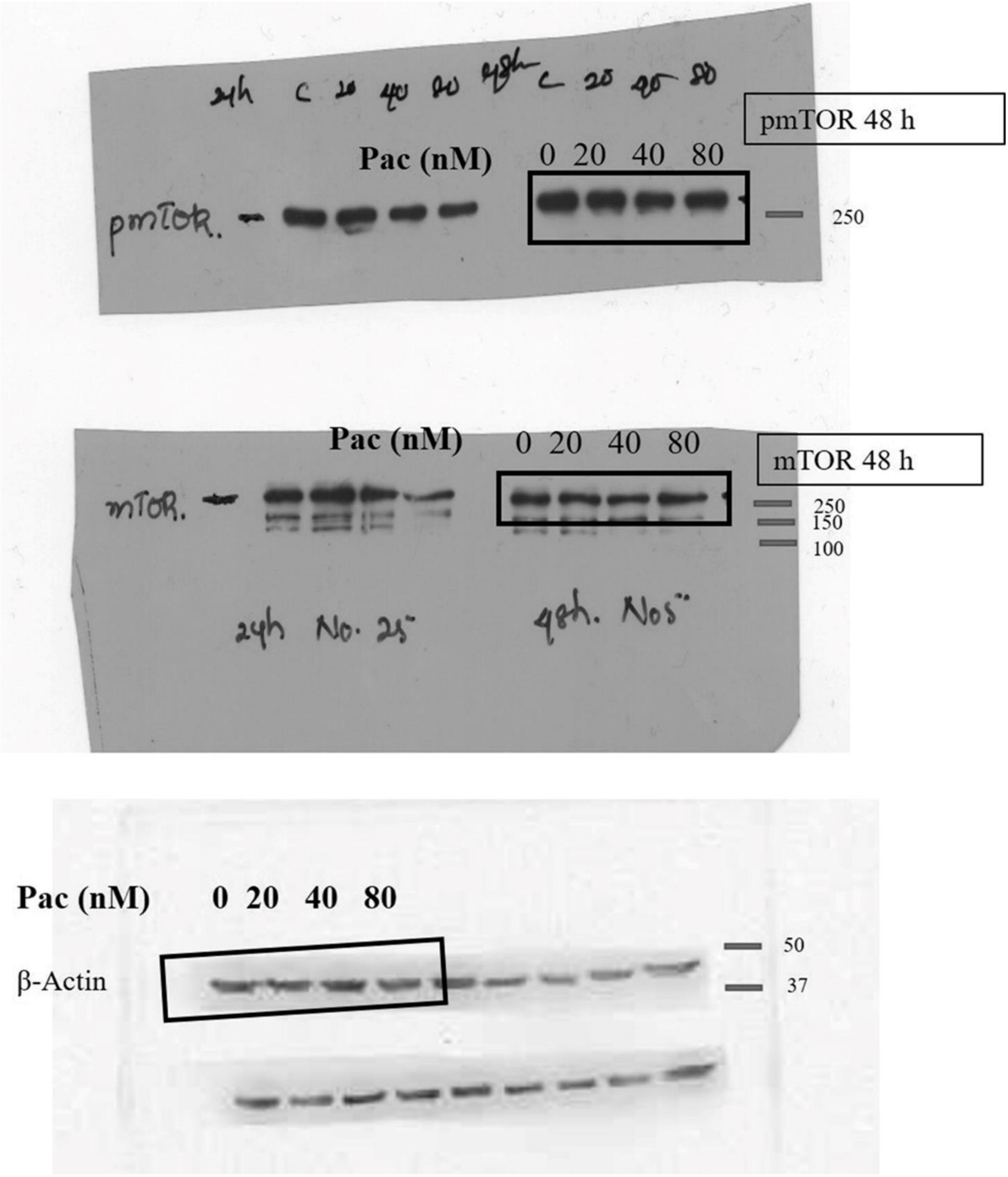

pmTOR 48 h
Pac (nM)
0 20 40 80
250
mTOR 48 h
Pac (nM)
0 20 40 80
250
150
100
Pac (nM)
0 20 40 80
β-Actin
50
37

p-m TOR

m TOR

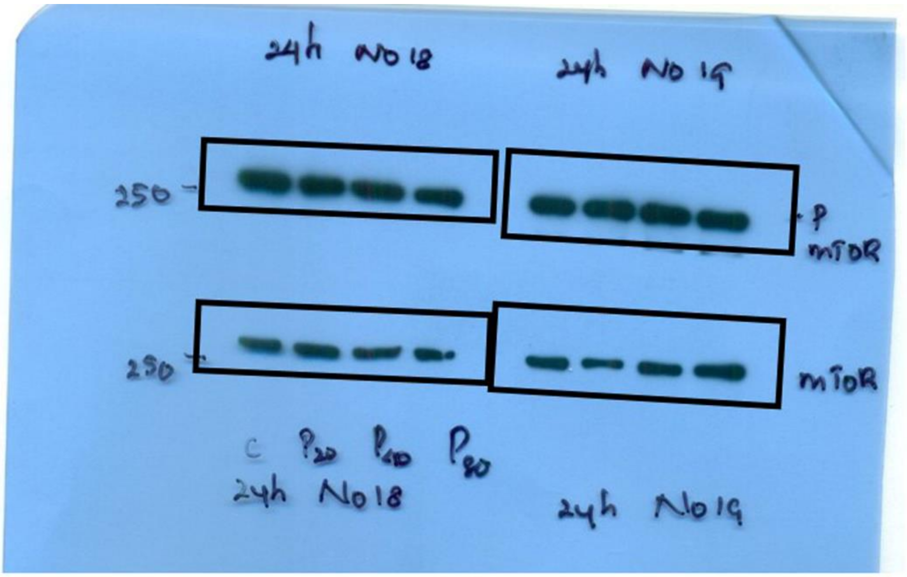

m TOR

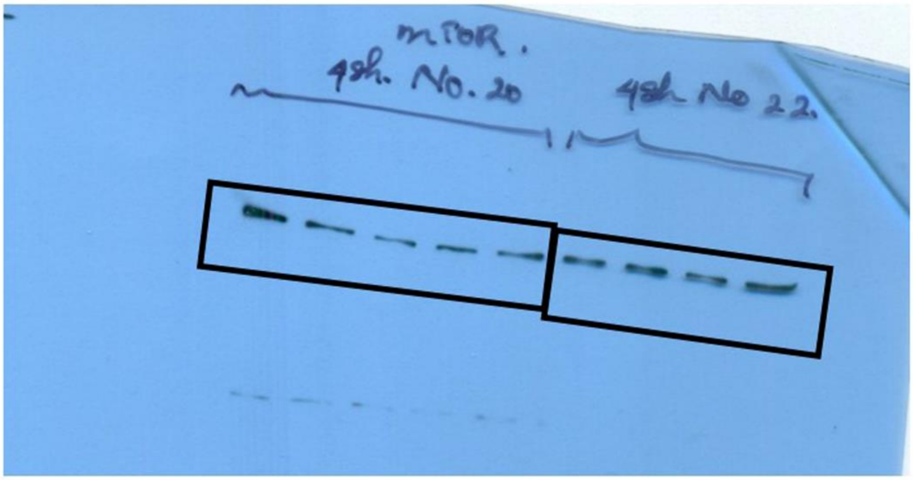

m TOR

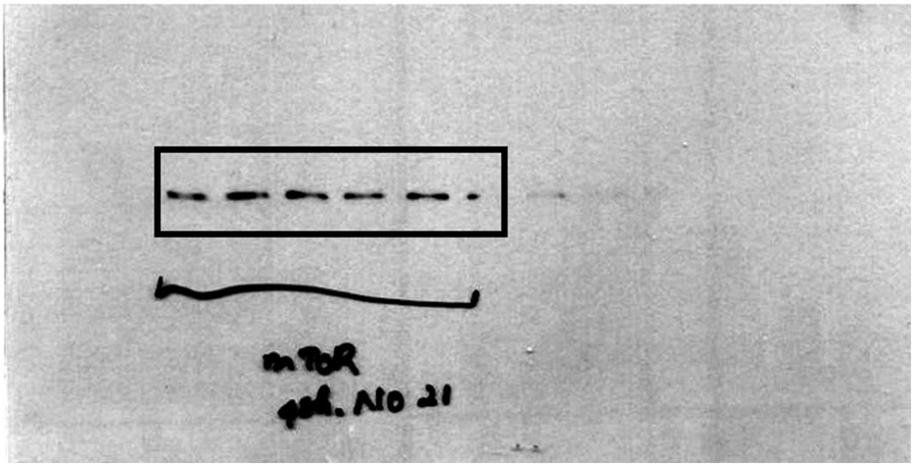

m TOR

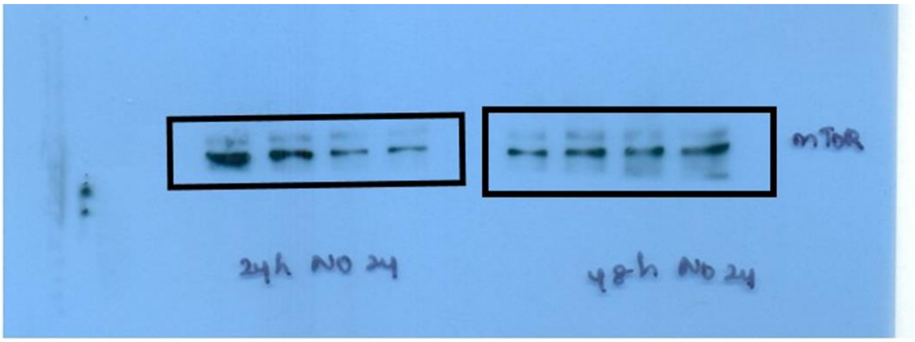

p-m TOR

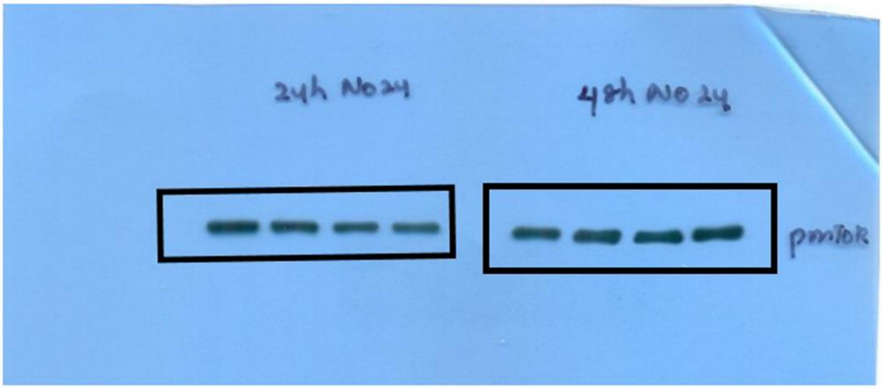

p-m TOR

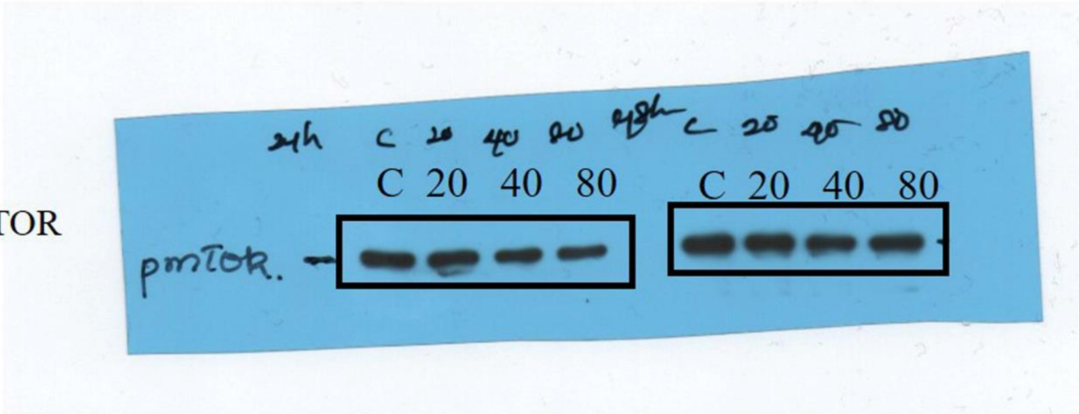

m TOR

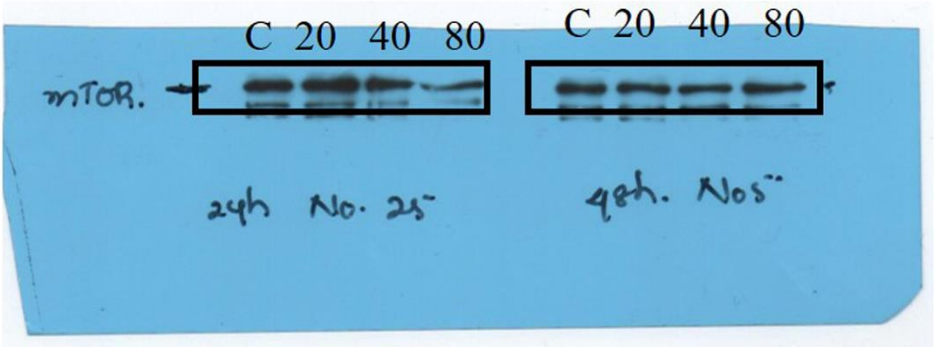

Fig. 5g

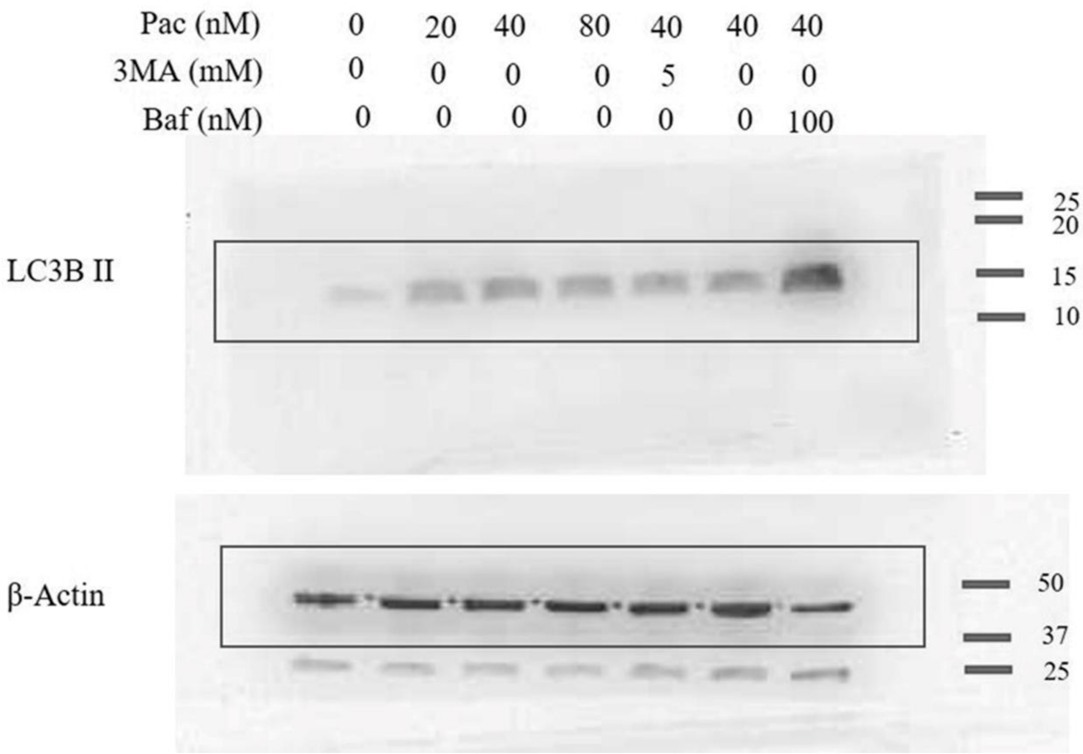

Pac (nM) 0 20 40 80 40 40 40
3MA (mM) 0 0 0 0 5 0 0
Baf (nM) 0 0 0 0 0 0 100
LC3B II
25
20
15
10
β-Actin
50
37
25

Supporting data

Fig. FF Photographs of the replicated full-length blots described in Figure 6

Fig. 6b

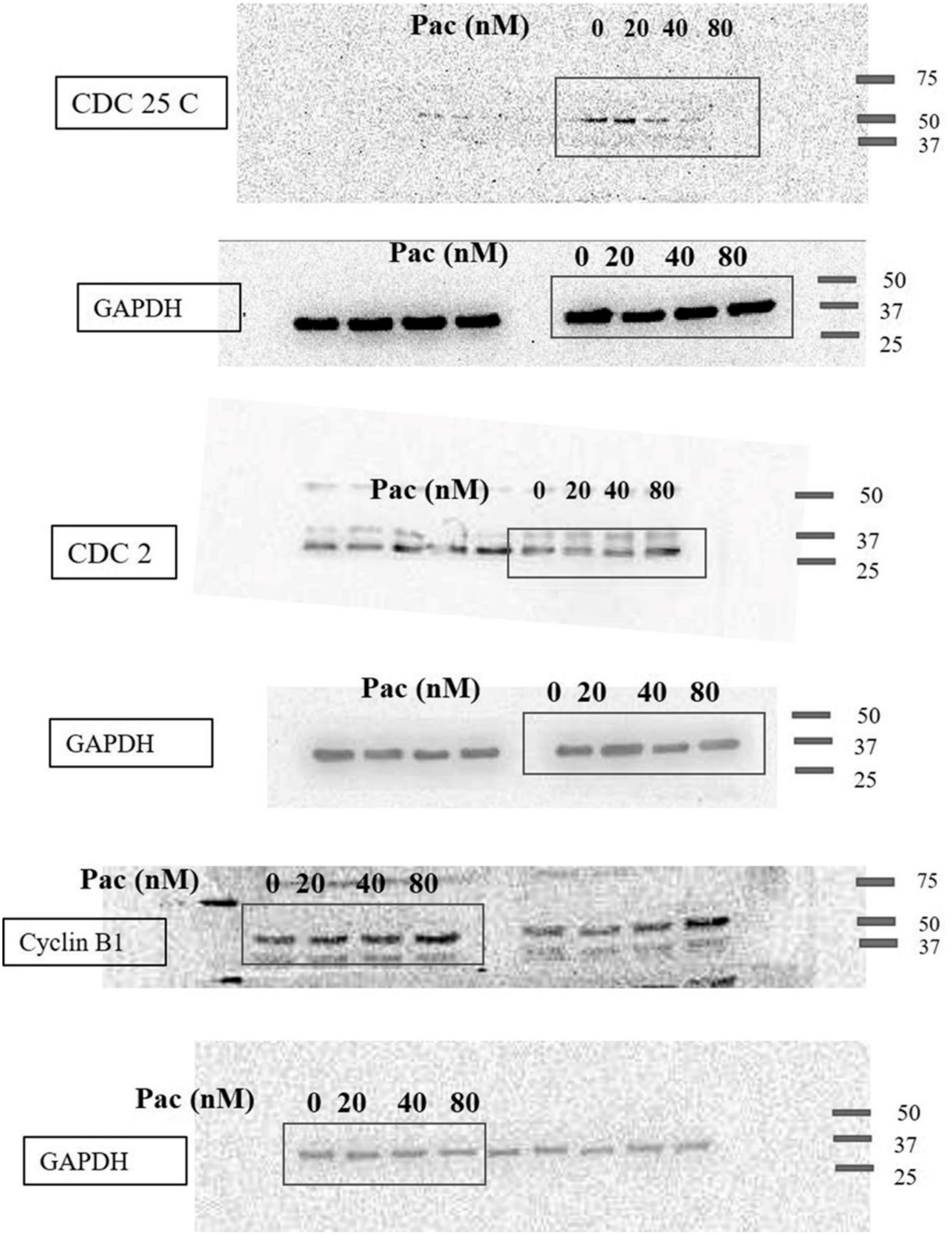

Fig. 6d

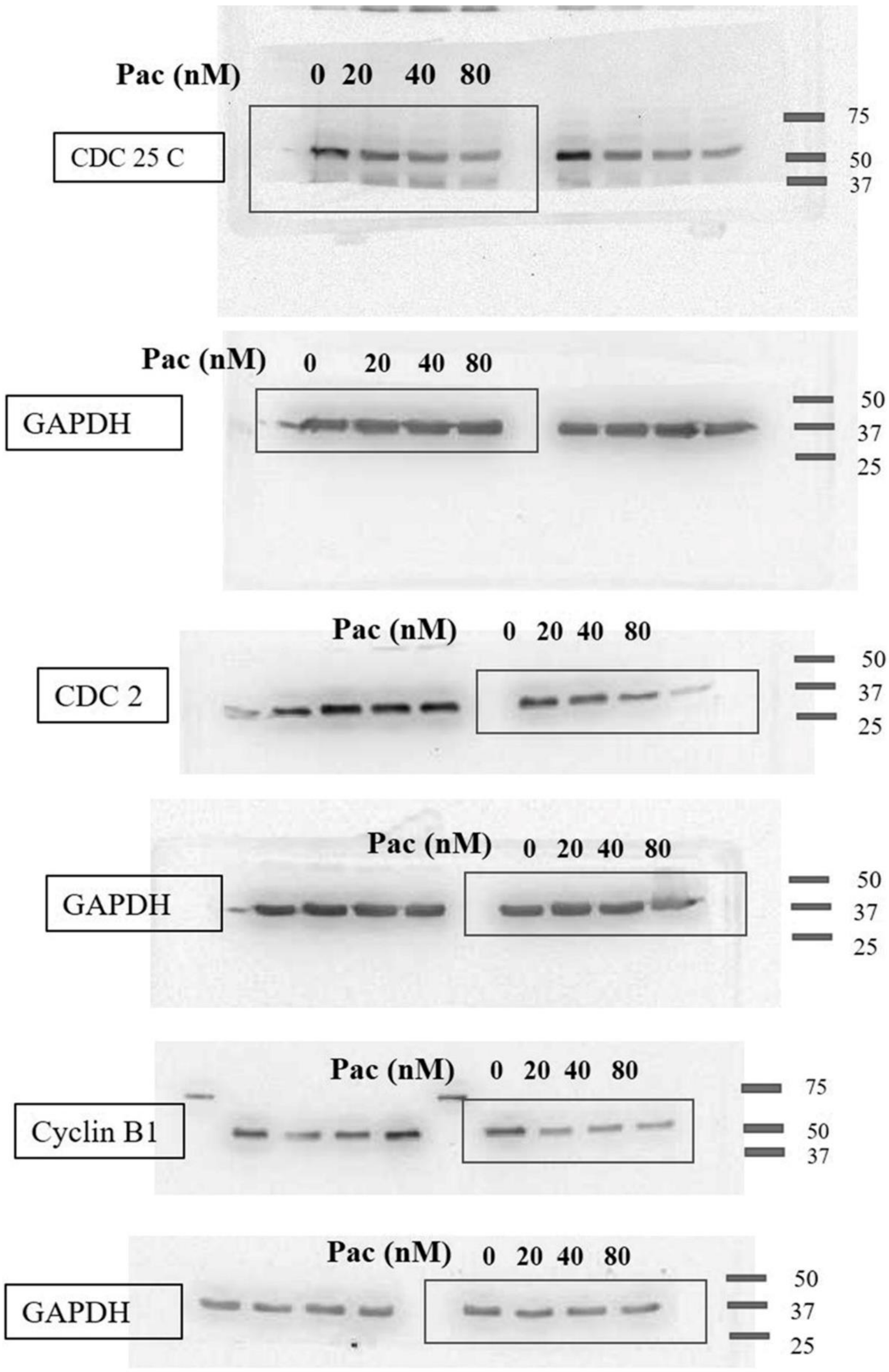
Pac (nM) 0 20 40 80
CDC 25 C
75
50
37
Pac (nM) 0 20 40 80
GAPDH
50
37
25
Pac (nM) 0 20 40 80
CDC 2
50
37
25
Pac (nM) 0 20 40 80
GAPDH
50
37
25
Pac (nM) 0 20 40 80
Cyclin B1
75
50
37
Pac (nM) 0 20 40 80
GAPDH
50
37
25

cdc25c

CDC 25c

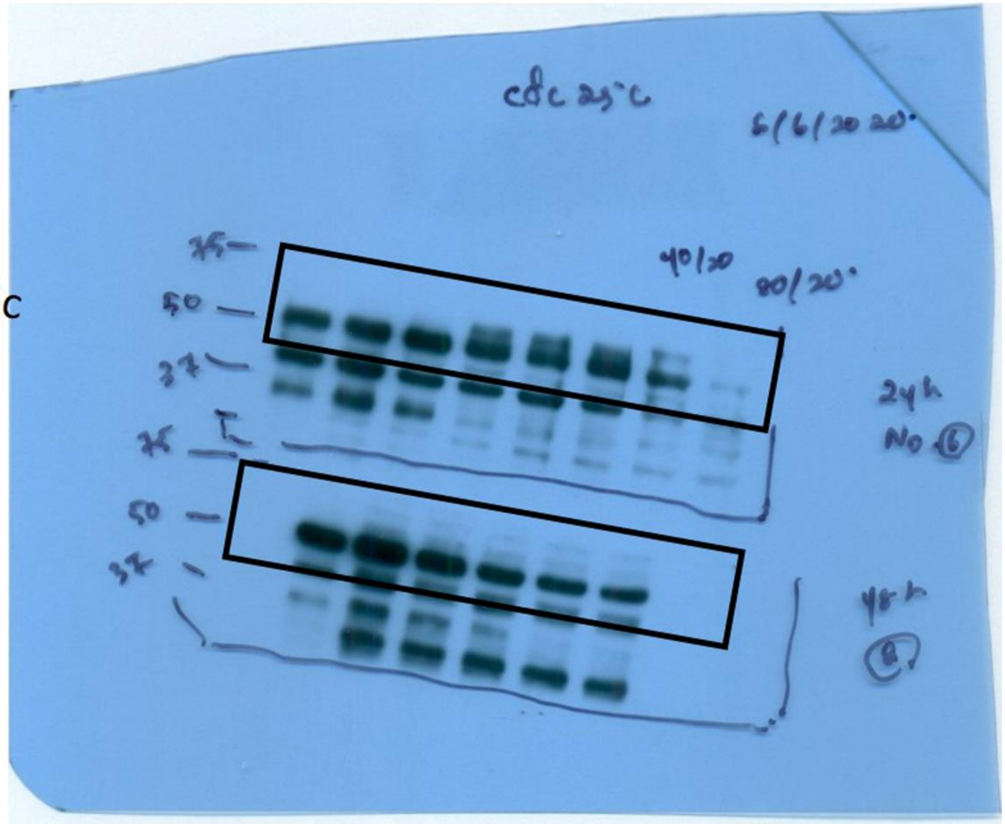

CDC 25c

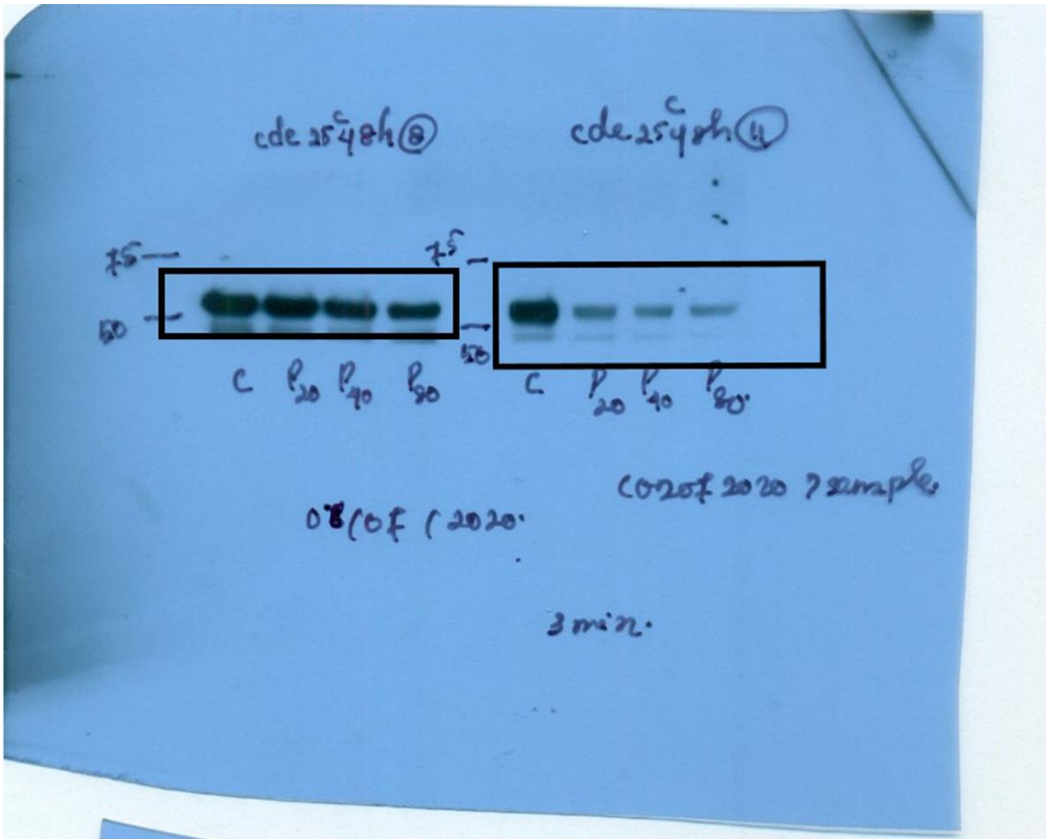

CDC 25c

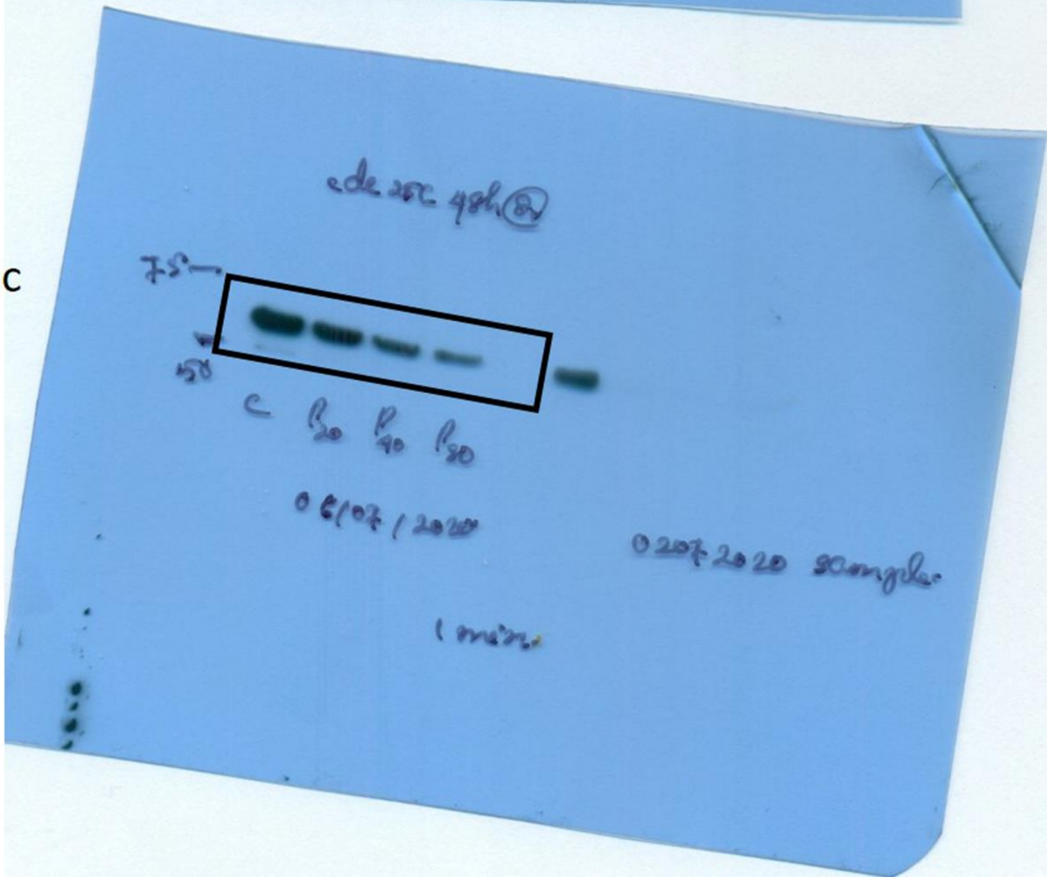

cdc25c

48 h

24 h

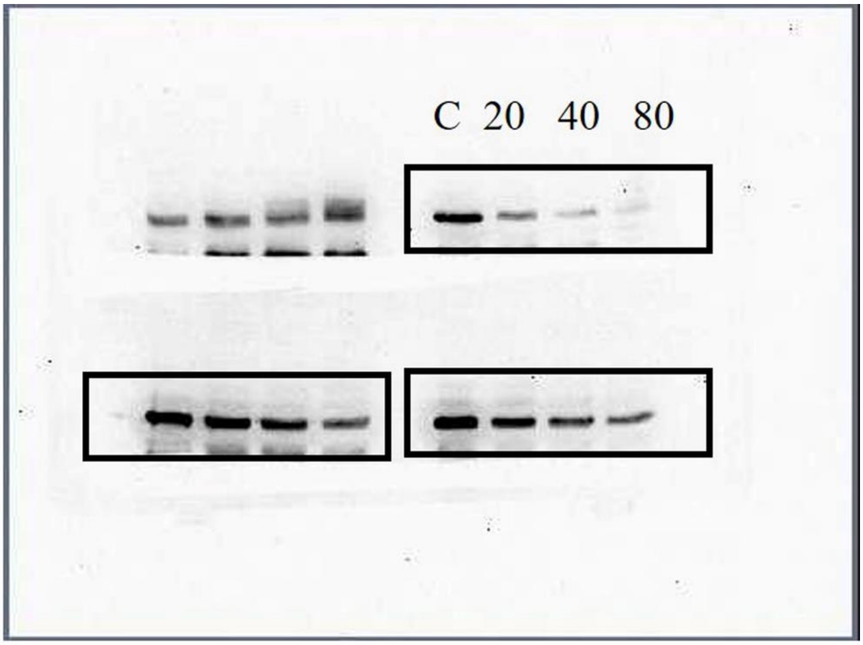

cdc25c

24 h

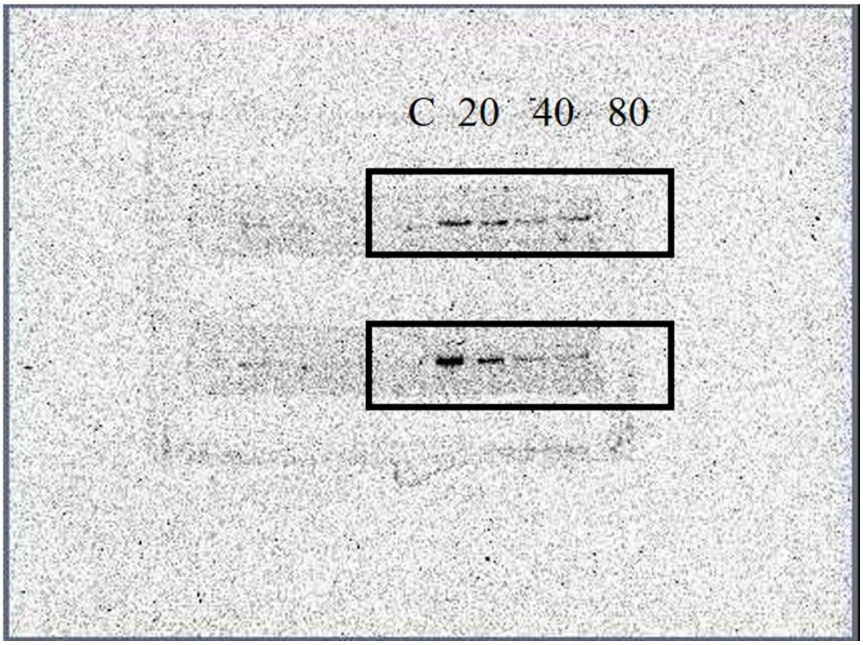

cdc-2

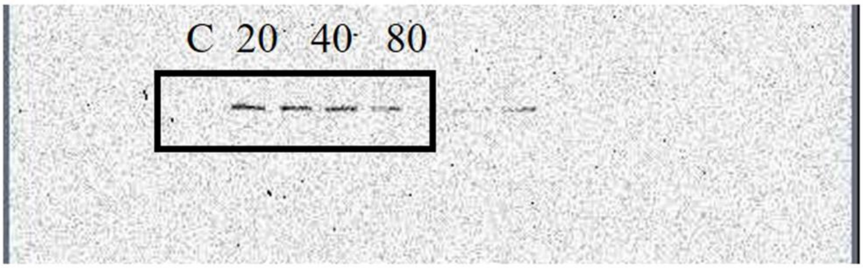

cdc-2

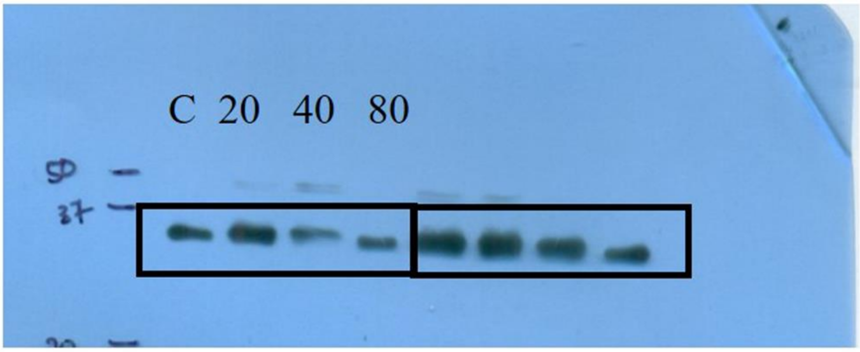

GAPDH

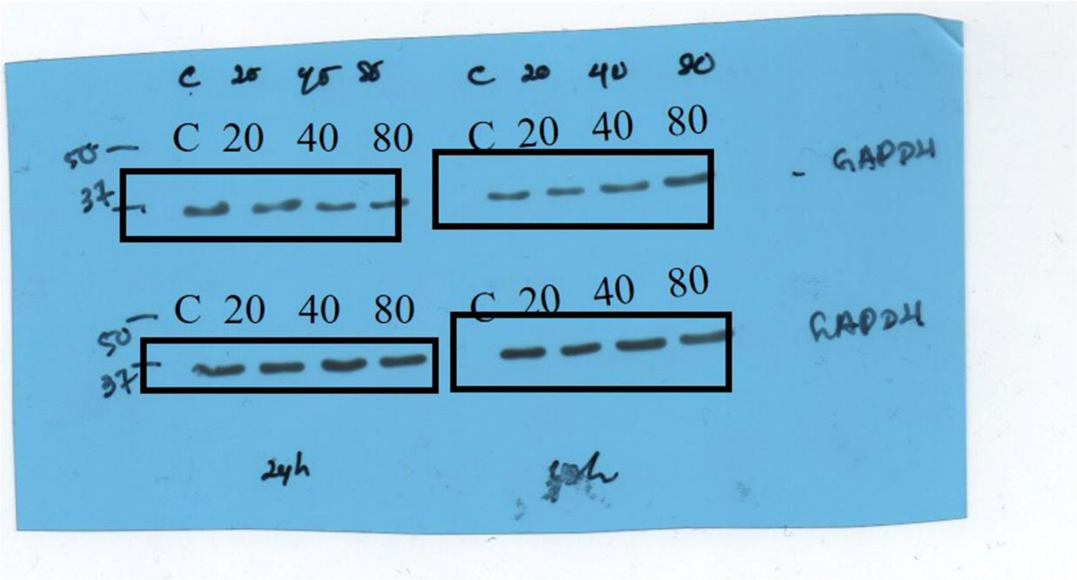

Cyclin b1

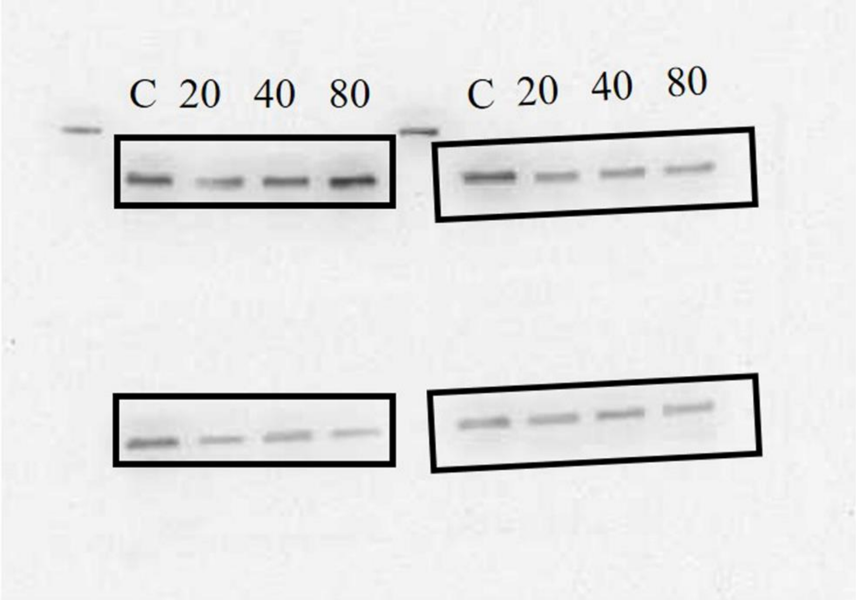

Cyclin b1

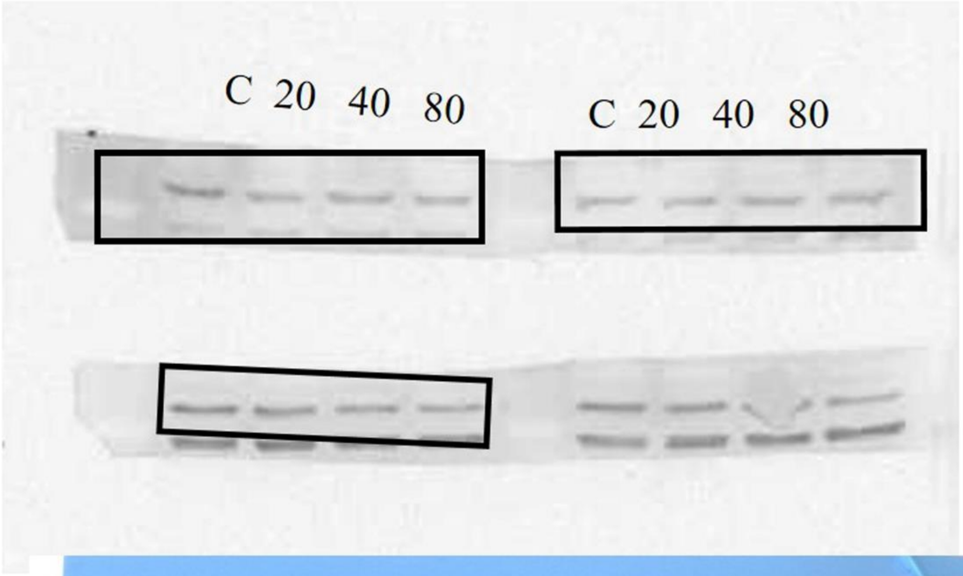

GAPDH

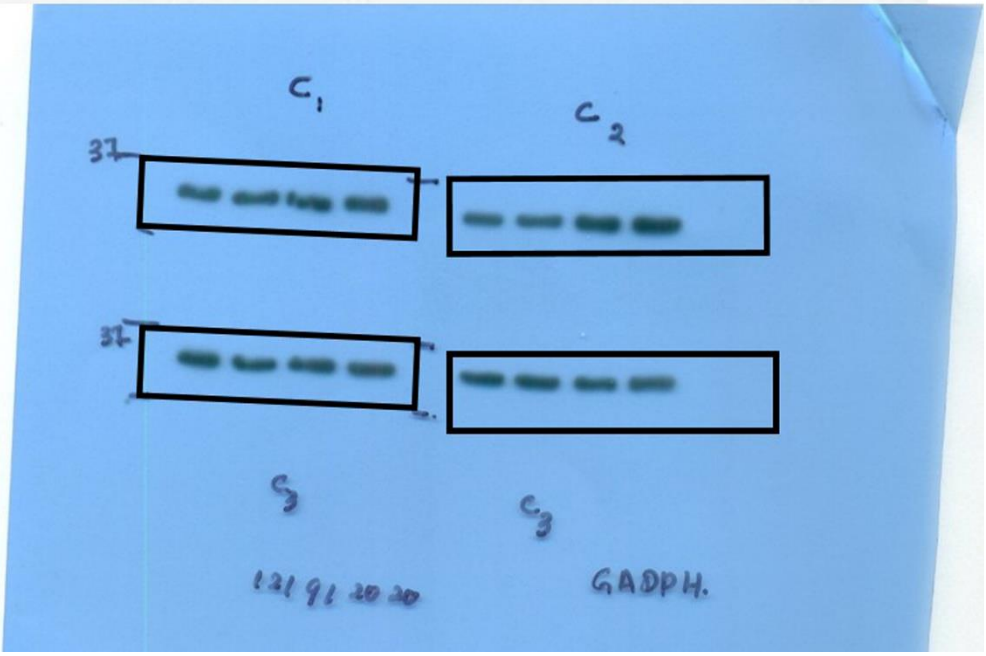

Fig. 7f

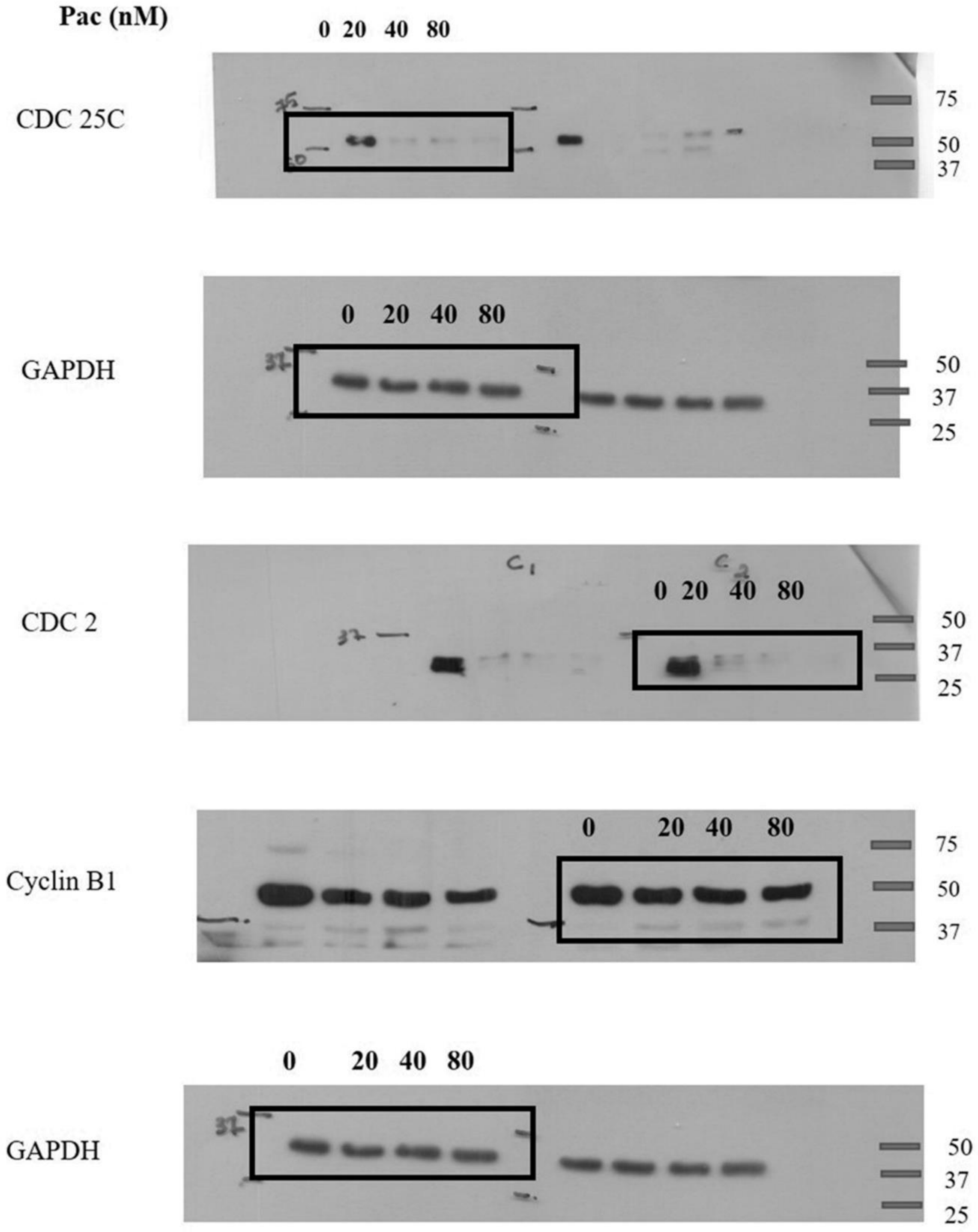
Pac (nM)
0 20 40 80
CDC 25C
75
50
37
0 20 40 80
GAPDH
50
37
25
0 20 40 80
CDC 2
50
37
25
0 20 40 80
Cyclin B1
75
50
37
0 20 40 80
GAPDH
50
37
25

CDC 25c

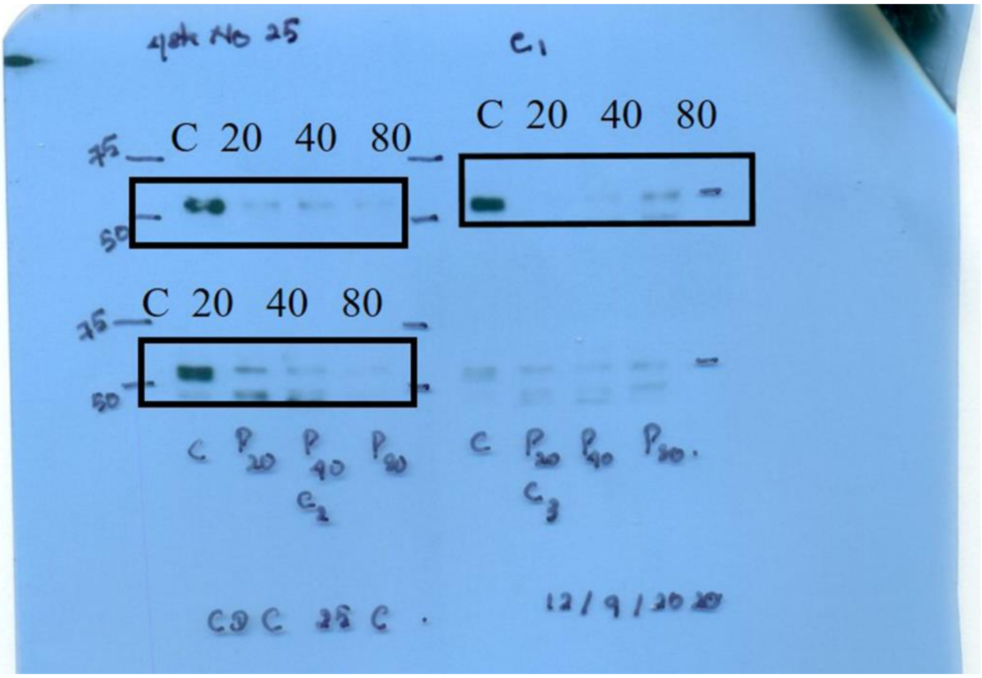

cdc 2

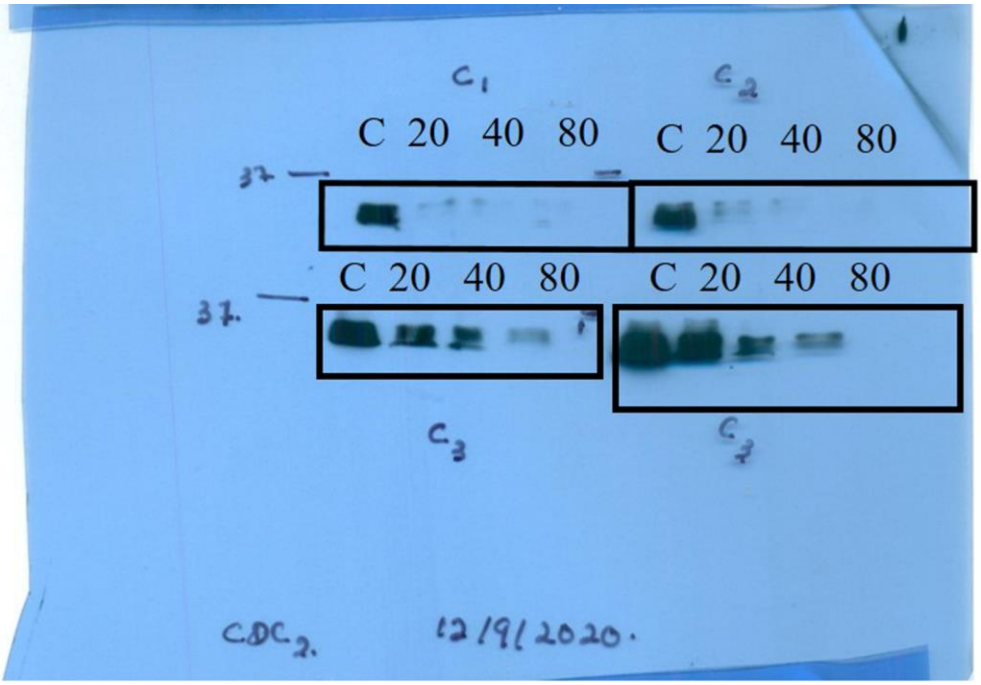

cdc 2

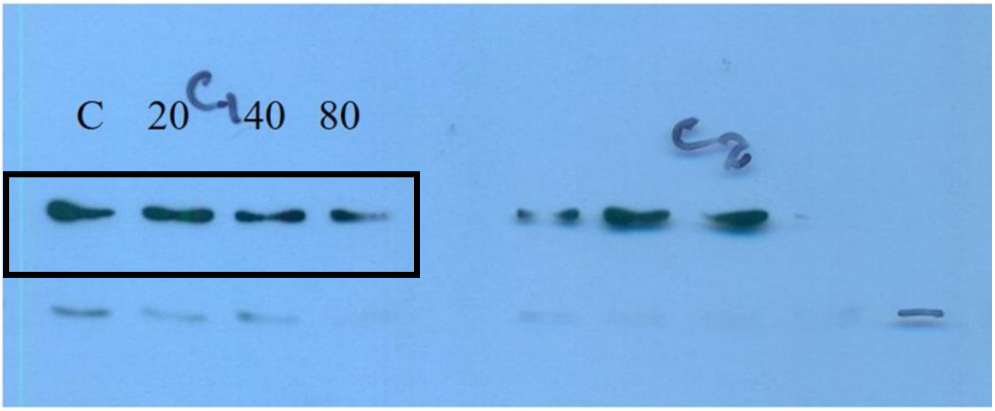

GAPDH

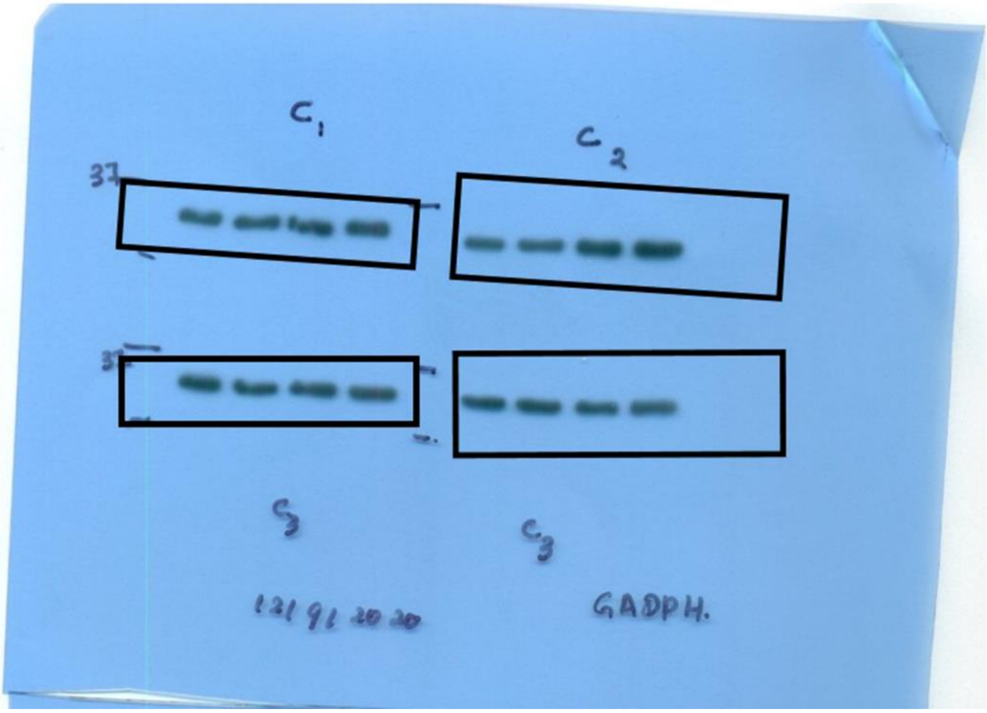

Cyclin b1

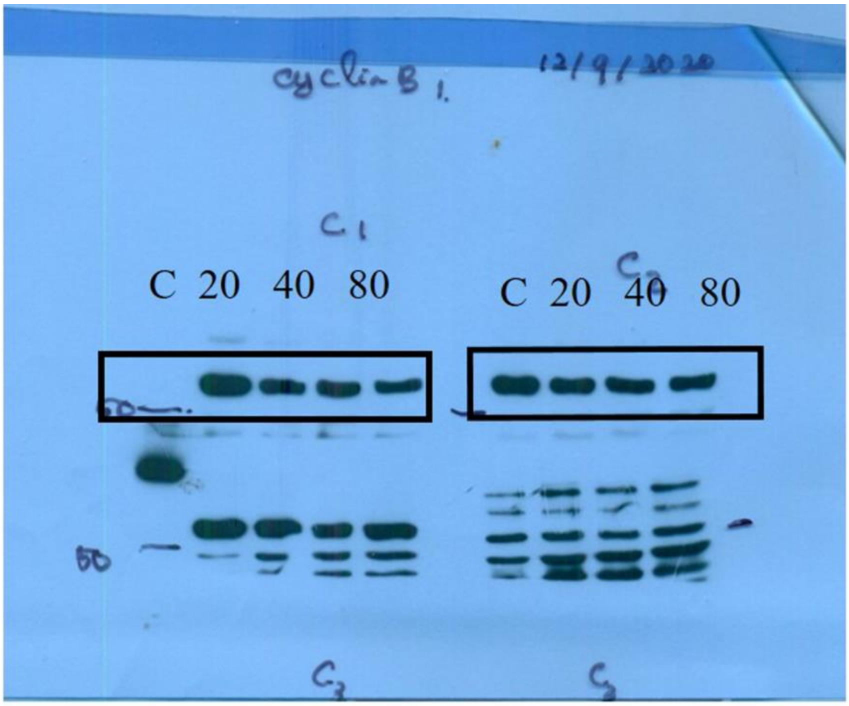

GAPDH

GAPDH

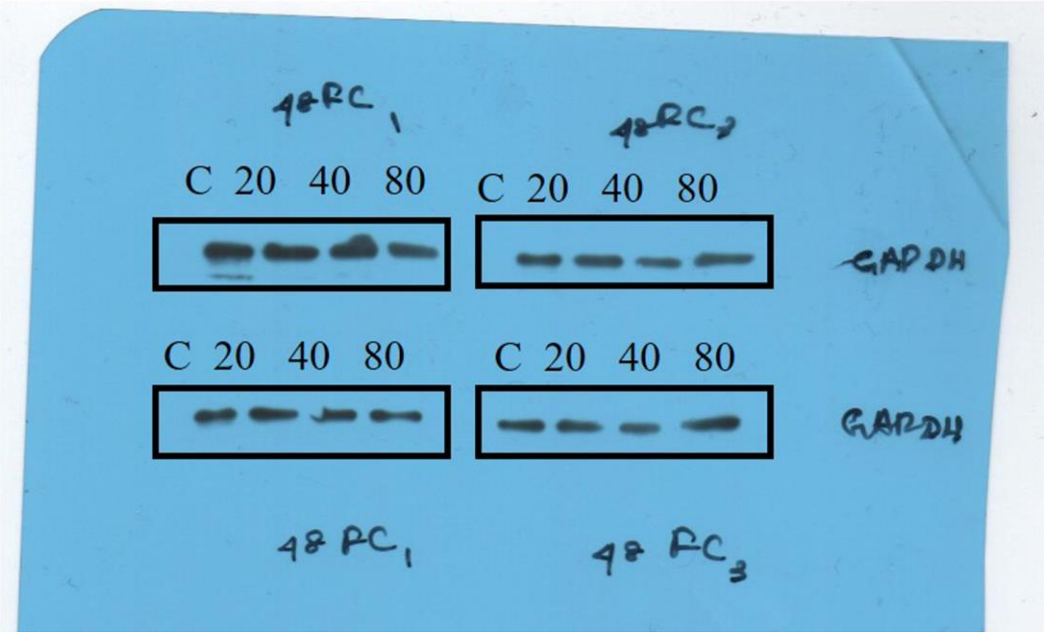

Supplementary Fig. G

Fig. G Photographs of the full-length blots utilized in Figure 7

Fig. 7g

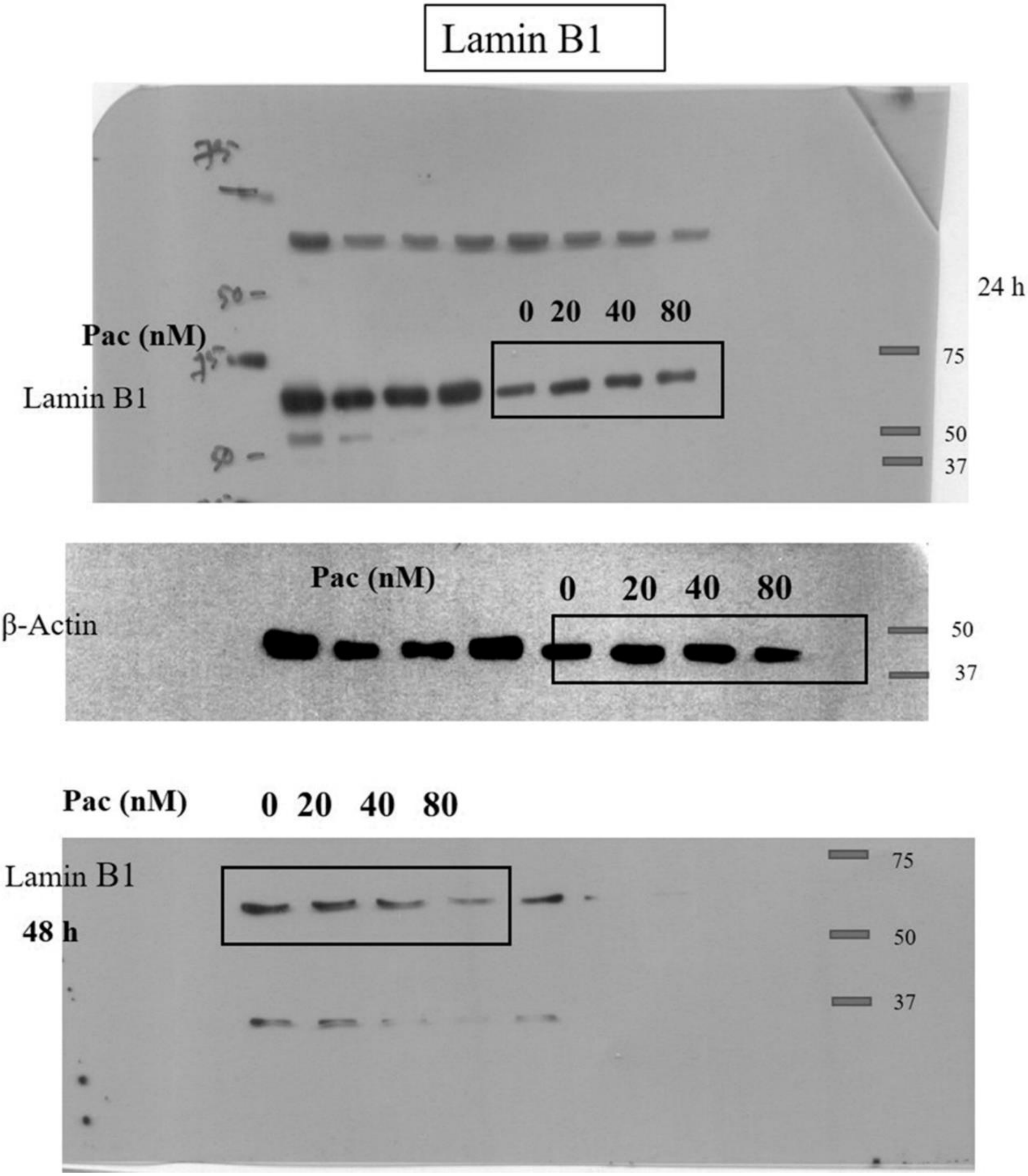

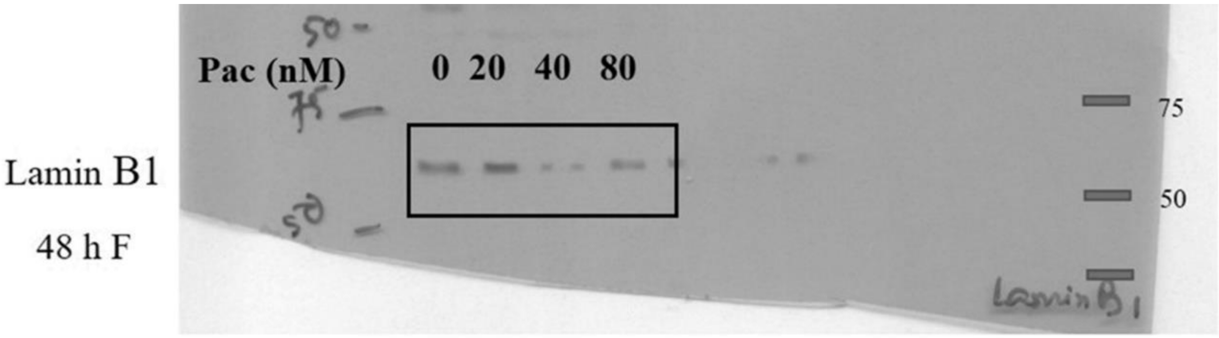
Pac (nM)
0 20 40 80
Lamin B1
48 h F
75
50

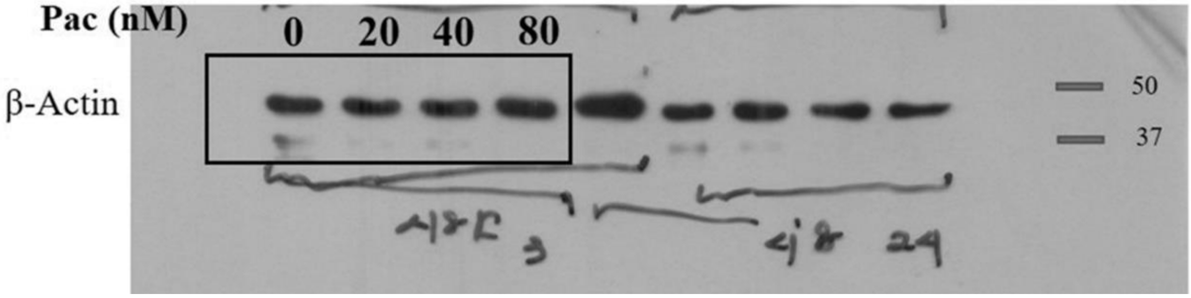
Pac (nM)
0 20 40 80
β-Actin
50
37

Lamin B1

24 h

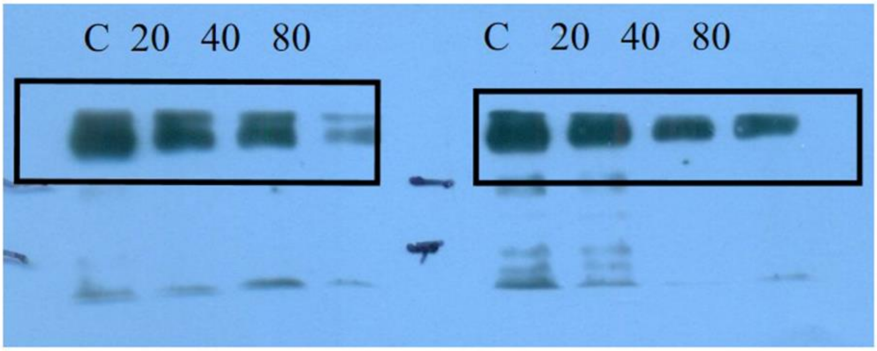

48 h

Lamin B1

24 h

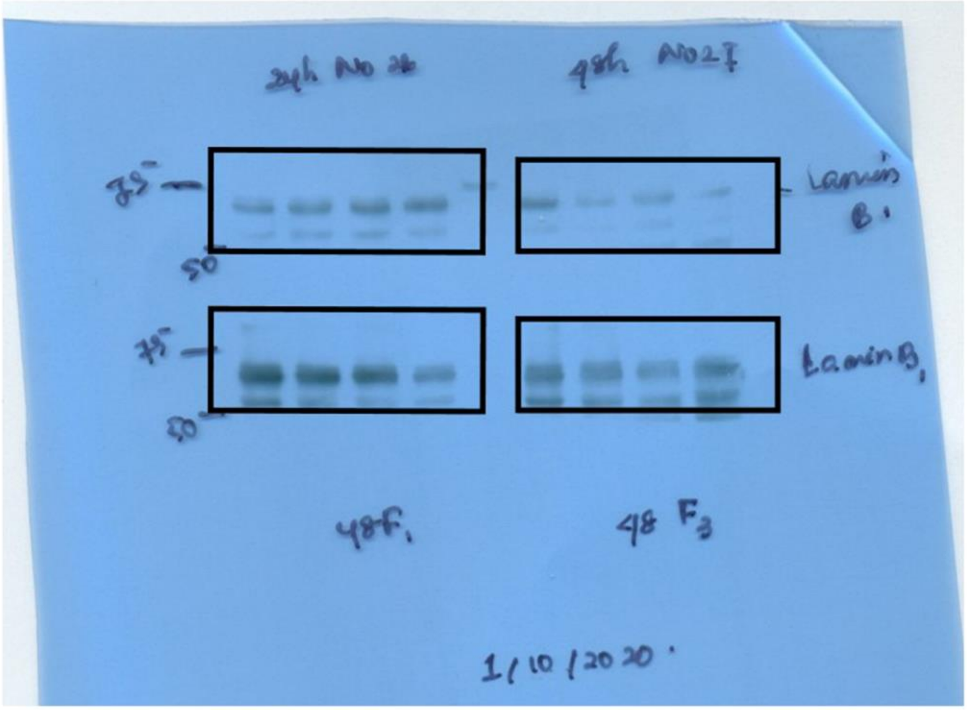

48 h

48 h F

Lamin B1

48 h

24 h

24 h

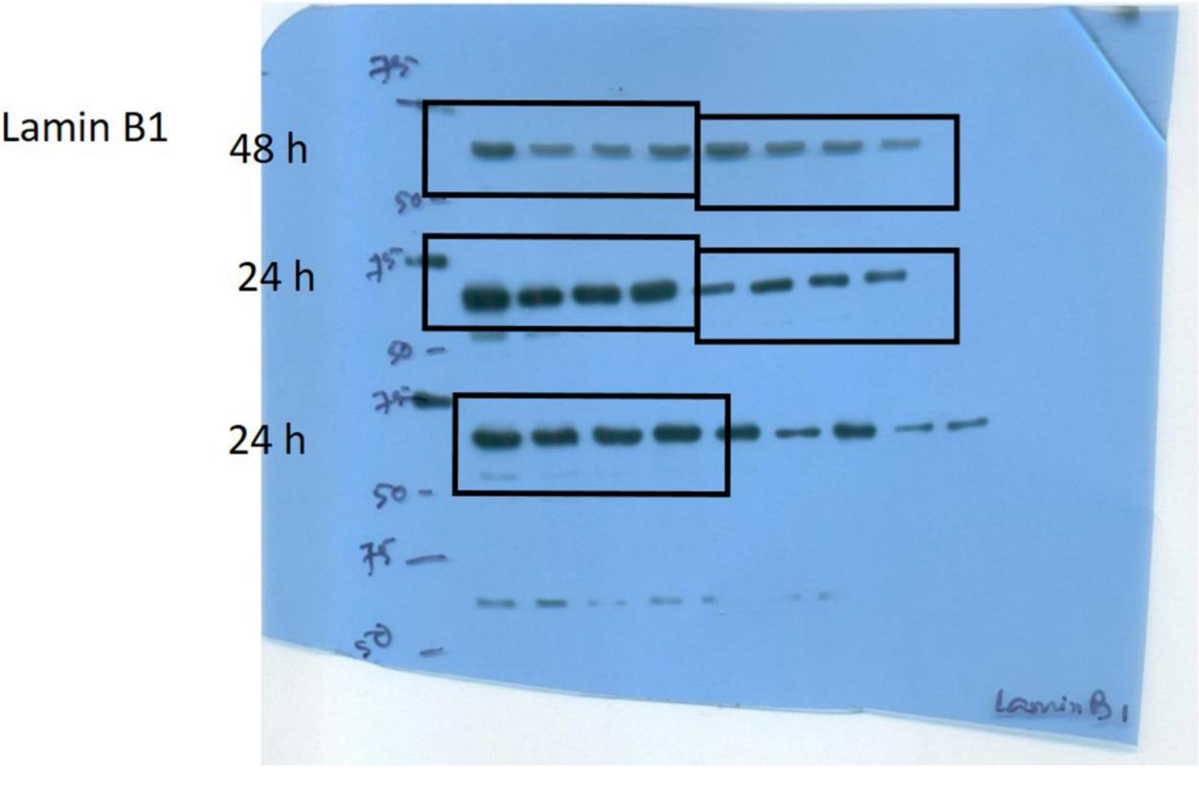

Fig. 7h

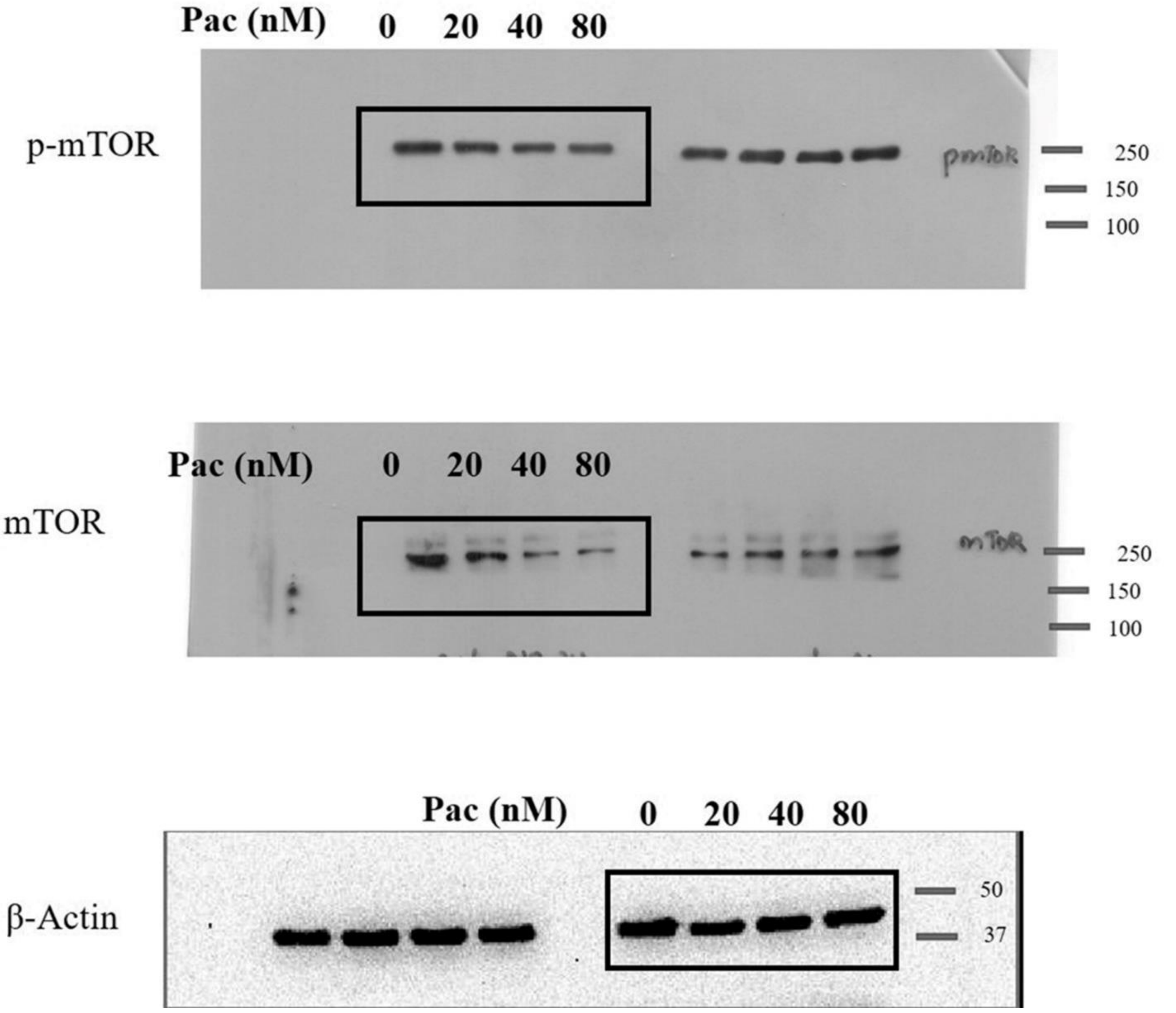

Pac (nM) 0 20 40 80
p-mTOR
pmTOR
250
150
100
Pac (nM) 0 20 40 80
mTOR
mTOR
250
150
100
Pac (nM) 0 20 40 80
β-Actin
50
37

mTOR/ pmTOR

Figure. 7h

mTOR

48 Followed by 24 h

P-mTOR

48 Followed by 24 h

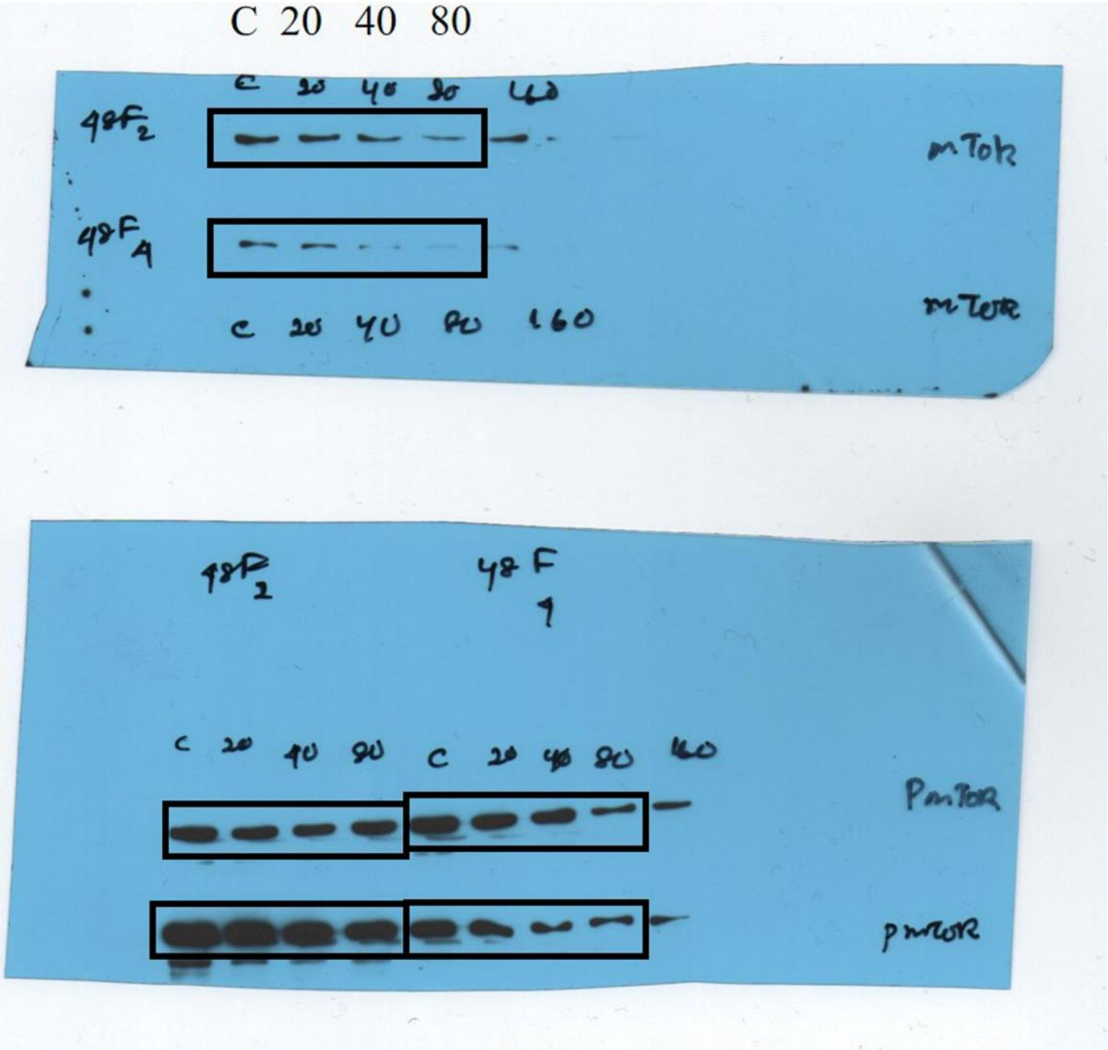

mTOR

48 Followed by 24 h

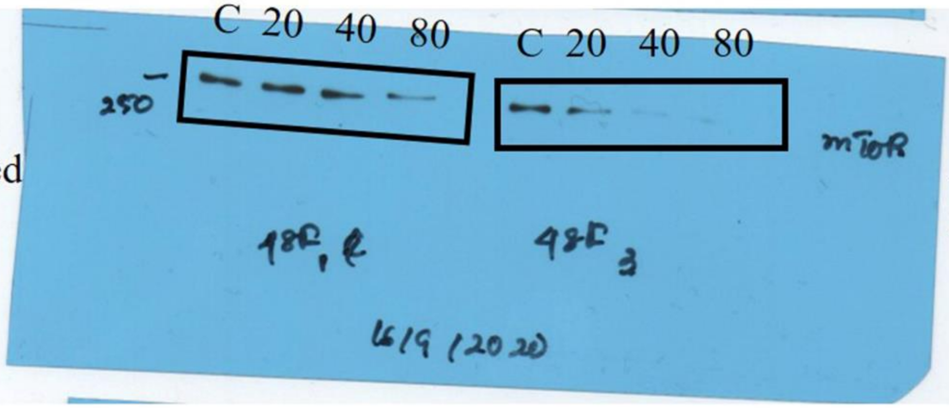

mTOR

48 Followed by 24 h

p-mTOR

48 Followed by 24 h

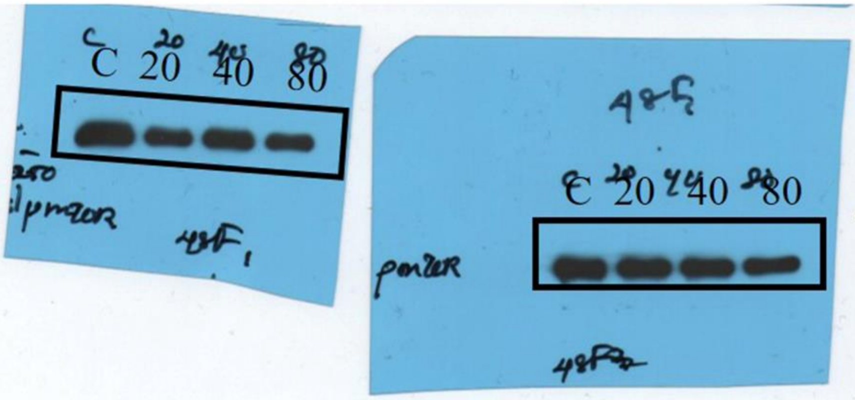

p-mTOR

48 Followed by 24 h

m TOR

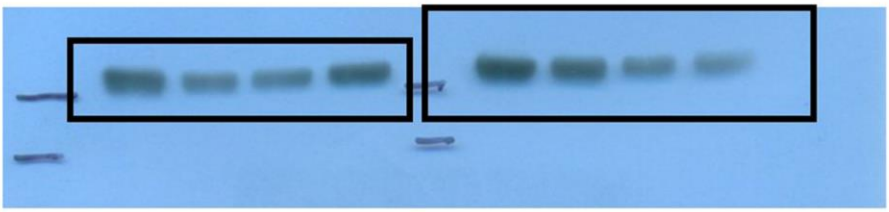

p-m TOR

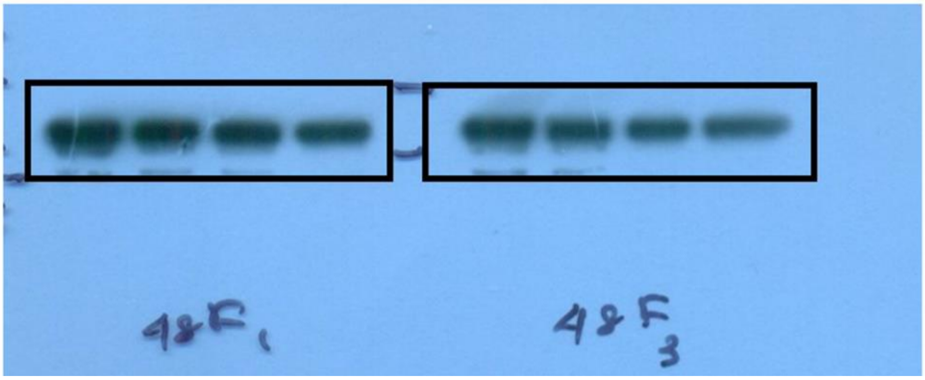

p-m TOR

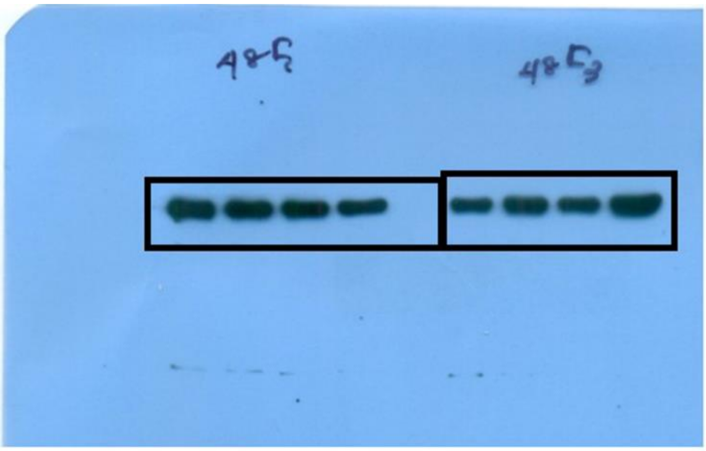

m TOR

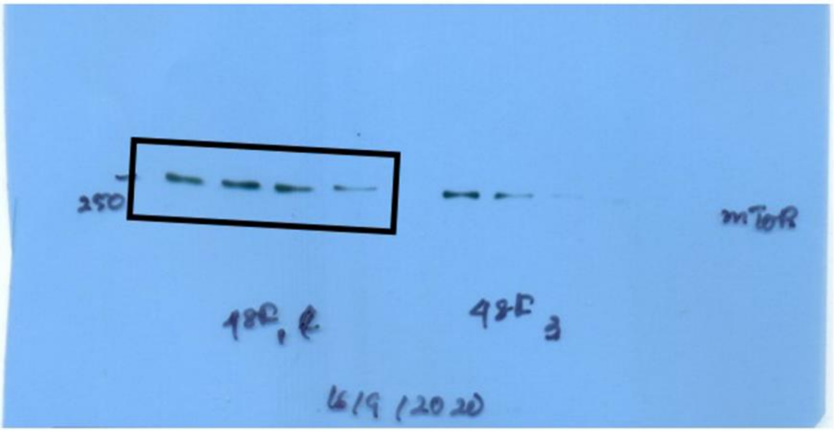

p-m TOR

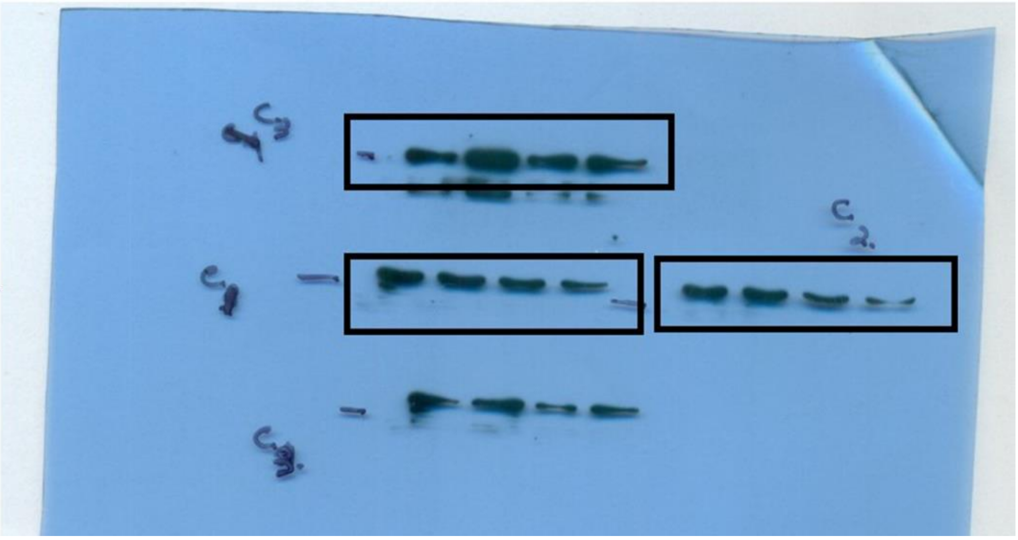

m TOR

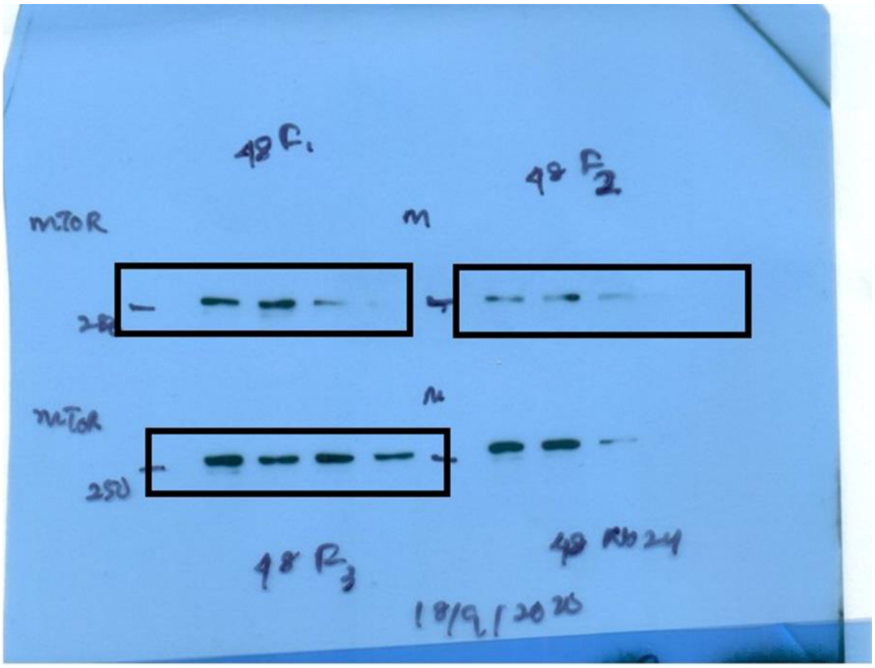

β-Actin

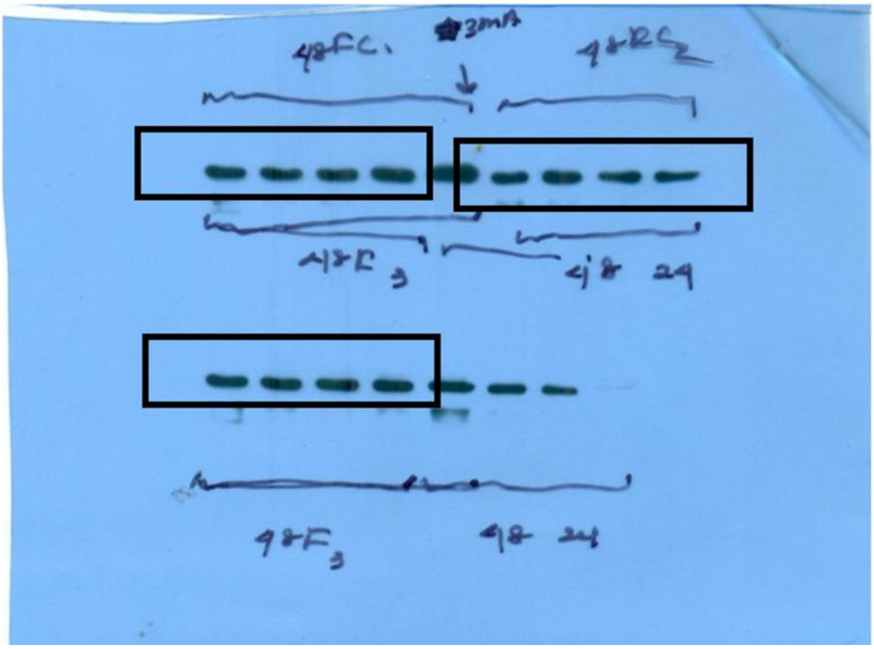

Supplement: Supplementary file 3 — Supplementary Information 3. [file 41598_2021_2503_MOESM3_ESM.pdf]
